# Supplementary material for: Modeling the disruption of respiratory disease clinical trials by non-pharmaceutical COVID-19 interventions
Source: Nat Commun. 2022 Apr 13;13:1980. doi: 10.1038/s41467-022-29534-8 (PMC9008035; doi:10.1038/s41467-022-29534-8)
Supplement: Supplementary file 1 — Supplementary Information [file 41467_2022_29534_MOESM1_ESM.pdf]

SUPPLEMENTARY INFORMATION FOR: Modeling the disruption of respiratory disease clinical trials by non-pharmaceutical COVID-19 interventions

Simon Arsène[1], Claire Couty[1, *], Igor Faddeenkov[1, *], Natacha Go[1, *], Solène Granjeon-Noriot[1, *], Daniel Šmít[1], Riad Kahoul[1], Ben Illigens[1, 3], Jean-Pierre Boissel[1], Aude Chevalier[2], Lorenz Lehr[2], Christian Pasquali[2], and Alexander Kulesza[1, †]

[1]Novadiscovery SA, Lyon, France
[2]OM Pharma, Meyrin, Switzerland
[3]Dresden International University, Dresden, Germany
[†]corresponding author(s): Alexander Kulesza (Alexander.Kulesza@novadiscovery.com)
[*]these authors contributed equally to this work

ABSTRACT

Respiratory disease trials are profoundly affected by non-pharmaceutical interventions (NPIs) against COVID-19 because they perturbed existing regular patterns of seasonal viral epidemics. To address trial design with such uncertainty, we developed an epidemiological model of respiratory tract infection (RTI) coupled to a mechanistic description of viral RTI episodes. We explore the impact of reduced viral transmission (mimicking NPIs) using a virtual population and in silico trials for OM-85 as prophylaxis for RTI. Ratio-based efficacy metrics are only impacted under strict lockdown whereas absolute benefit already is with intermediate NPI (eg. mask-wearing). Consequently, despite NPI, trials may meet their relative efficacy endpoints (provided recruitment hurdles can be overcome) but are difficult to assess with respect to clinical relevance. These results advocate to report a variety of metrics for benefit assessment, to use adaptive trial design and adapted statistical analyses. They also question eligibility criteria misaligned with the actual disease burden.

Supplementary Methods

Within-host viral infection disease model

To date, several modeling efforts have succeeded to model viral kinetics during a respiratory tract infection (RTI) mainly for influenza[1,2]. Building on this, we developed a disease model which attempts to capture the interplay between viral infections and immune dynamics in the respiratory tract tissues. The model accounts for the following mechanisms: 1) Exposure to the pathogen, onset and resolution of infection; 2) Target cell infection and pathogen replication; 3) Preemptive clearance by the innate immune system; 4) Innate inflammatory response; 5) Activation of the adaptive response; 6) Neutralization of the virus by antibodies; 7) Cytolysis of infected cells by cytotoxic lymphocytes; 8) Age-dependent maturation of the immune system; 9) General immunocompetence. The resulting ODE system is detailed as follows.

Variables

E_h Healthy epithelial cell
E_i Infected epithelial cell
Ig Virus-specific immunoglobulins produced by B cells during infection
Ig_A Non-specific immunoglobulins produced by OM-85 induced plasma cells
L Cytotoxic T lymphocytes
V Virions
M Pre-activated type 1 innate cells

ODEs

$$\dot{E}_h = p_h \cdot \bar{E}_h \left(1 - \frac{E_h + E_i}{\frac{\bar{E}_h \cdot p_h}{p_h - d_h}}\right) - d_h E_h - k_{inf} \cdot v_f E_h V \frac{1}{\left(1 + \frac{c}{V}\right)^2} \tag{1}$$

$$\dot{E}_i = k_{inf} \cdot v_f E_h V \frac{1}{\left(1+\frac{c}{V}\right)^2} - d_i E_i - n_i L E_i \tag{2}$$

$$\dot{Ig} = p_{ig} \cdot S \frac{V}{K_V + V}\left(1 + p_M \frac{M}{K_M + M}\right) - d_{ig} Ig \tag{3}$$

$$\dot{Ig}_A = -d_{IgA} \cdot Ig_A \tag{4}$$

$$\dot{L} = -d_L L + p_L \cdot S \frac{V}{K_V + V}\left(1 + p_M \frac{M}{K_M + M}\right) \tag{5}$$

$$\dot{V} = -k_{inf} \cdot v_f E_h V \frac{1}{\left(1+\frac{c}{V}\right)^2} + p_v \cdot v_f E_i \frac{1}{\left(1+\frac{c}{E_i}\right)^2} - d_v V - n_v \left(Ig + Ig_A\right) V \tag{6}$$

Parameters

In the context of a single RTI, we calibrated the set of unknown parameters of the model against mean viral load of individual profiles obtained during an experimental human RSV (HRSV) infection[3] (Figure 2). The complete set of parameters is described in Table 1. Calibration was carried out using the covariance matrix adaptation evolution strategy (CMA-ES) algorithm[4] with constraints on mean viral load during a single infection events.

Between-host SIRS model

The between-host SIRS model follows the classical formalism[5,6] with populations of size N, partitioned into susceptible S, infected I and recovered R populations. Healthy susceptible individuals S may be infected with an infection rate β, leading them to become infected individuals I. Those can then recover after a given time with a recovery rate γ, leading them to become recovered individuals R. Since we are interested in recurrent infections, recovered individuals R may lose their immunity after a given time, represented by an immunity loss rate ζ, and become as a consequence susceptible again (S) to an infection. The system of differential equations reads:

$$\dot{S} = -\beta \cdot S \cdot I + \zeta \cdot R \tag{7}$$

$$\dot{I} = \beta \cdot S \cdot I - \gamma \cdot I \tag{8}$$

$$\dot{R} = \gamma \cdot I - \zeta \cdot R \tag{9}$$

The most common respiratory viruses causing RTIs worldwide in children are respiratory syncytial viruses (RSV), influenza viruses and rhinoviruses[7,8]. Therefore, we used three parallel SIRS models for each virus class without discriminating between viral strain. To capture seasonal variation of temperature in temperate climates[9], we used the common approach[10] where a periodic time-varying function is considered instead of a constant taken for the transmission parameter β: $\beta(t) = b_0 \cdot (1 + b_1 \cdot cos(2\pi t + \phi))$. For rhinoviruses, which show approximately a biannual behaviour[11], we divided the period by two.

The virus-dependent sets of parameters were calibrated using virus-specific incidence data[12–14] (Figure 4) and are detailed in Table 2. Similarly to the within-host model, the calibration was carried out using the covariance matrix adaptation evolution strategy (CMA-ES) algorithm[4] with constraints on virus-specific incidence data. Furthermore, each of the respiratory viruses has a unique susceptibility to cause either an upper RTI (URTI) or lower RTI (LRTI)[15,16], which we use for probabilistic guidance on the determination of whether an RTI is a normally milder URTI or a usually more severe LRTI[17].

To include the impact of COVID-19 containment measures, we simply decrease the transmission parameter β similarly to what is often reported in the literature[18,19] (Figure 1). This allows to explore the impact of the strength of the containment measures.

PBPK/PD model of OM-85 effect

OM-85 is administered orally and is absorbed in the intestine, triggering gut-associated lymphoid tissue (GALT) stimulation and subsequently generating the immune response within mucosal tissue (MALT) in other organs such as the respiratory tract[20]. The key factors in this chain-like reaction are reactive Peyers Patches (PPs) of GALT, responsible for antigen identification and subsequent generation of the adequate response. To model this, we used a physiologically based pharmacokinetic (PBPK) model coupled with a pharmacodynamics (PD) model of immune response in the GALT PPs. PBPK models map the complex drug transport scheme onto a physiologically realistic compartmental structure and in this sense are predetermined and largely independent of the particular drug of interest[21]. We incorporated a gut absorption model (Advanced CAT model[22]) but disregarded disintegration and dissolution for simplicity since the bacterial lysates such as OM-85 are highly soluble in water. The PBPK structure of the model allows us to use systemic blood circulation and lymphatic circuits to describe migration of activated immune cells from Peyer's Patches to mesenteric lymph nodes into the bloodstream with consecutive homing to effector sites such as respiratory tract mucosal tissue (Figure 5).

Pharmacokinetics data are available for a radiolabeled derivative of a product (OM-89) similar to OM-85 but only in rodents[23,24]. To incorporate those and parameterize the PK drug-specific parameters, we transformed our PBPK model into an inter-species model by allometric scaling and species-specific parameters[25–30]. Based on these data (25% of radioactivity detected in exhaled air) we incorporated a non-specific non-renal clearance as well as a metabolic clearance in the liver. Result of this calibration are presented in Figure 6 for agreement between the data and the simulations and in Table 3 for the values of the calibrated parameters.

For the pharmacodynamics effect of OM-85 in the GALT: in the first stage, when OM-85 reaches PPs, interactions between pathogen-associated molecular pattern (PAMPs) and pattern recognition receptors (PRR) induce a nonspecific activation of innate immune cells, including macrophage, monocyte, dendritic cell, natural killer cells and granulocyte with cytokines and chemokines production, activation of phagocytosis and early pathogens destruction[31,32]. In the second stage, antigen-specific T and B cells are generated in the PPs as well as a considerable number of lymphoblasts, mostly immunoglobulin A (IgA) precursors of the IgA producing plasmocytes[32]. In a last stage, lymphocytes and lymphoblasts maturate in mesentertic lymph nodes (mLNs) and subsequently migrate into MALT of various organs[32], leading to an increased production of antibacterial antibodies in serum, saliva, PPs, mLNs, but also the respiratory tract[31]. In order to model these mechanisms, we built a model that focuses on the perturbation of GALT homeostasis resulting from OM-85 administration in the Peyer's patches (Figure 5). Because of the scarcity of pharmacodynamic data and markers in human, we did not attempt to represent the complex interactions between all immune cells in GALT at homeostasis and instead focus on a few key immune cells and interactions in the immunomodulatory response to OM-85 (dendritic cells, innate cells, IgA+ B and plasma cells, and regulatory T cells). The resulting ODE system (in two representation compartments: a Peyer's Patch and the respiratory tract tissue) is presented below.

Variables

D Dendritic cells
O OM-85 drug
M_p Reprogrammed type-1 innate progenitors
M Pre-activated type 1 innate cells
B_L IgA+ memory B cells
B_P IgA+ plasma cells
T_r Regulatory T cells
Ig_A Non-specific IgA
X_V Concentration of X in the vascular compartment of a given organ

ODEs

Peyer's Patch

$$\dot{D} = E_O \cdot \frac{O^h}{K_O^h + O^h} - d_D \cdot D \tag{10}$$

$$\dot{M_p} = E_{M_p} \frac{D}{K_{M_p} + D} - (\alpha + d_{M_p}) \cdot M_p \tag{11}$$

$$\dot{M} = \alpha \cdot M_p - L_{PP} \cdot (1 - \sigma_{PP}) \cdot M \tag{12}$$

$$\dot{B_L} = E_{B_L} \frac{D}{K_{B_L} + D} - (\beta + d_{B_L}) \cdot B_L \tag{13}$$

$$\dot{B_P} = \left(\beta + E_{B_P} \frac{D}{K_{B_P} + D} \right) B_L - (d_{B_P} + L_{PP} \cdot (1 - \sigma_{PP})) \cdot B_P \tag{14}$$

$$\dot{T_r} = E_{T_r} \frac{D}{K_{T_r} + D} - (d_{T_r} + L_{PP} \cdot (1 - \sigma_{PP})) \cdot T_r \tag{15}$$

Respiratory tract tissue

$$\dot{M} = L \cdot \sigma_V \cdot (1 - \sigma_V^S) \cdot M_V - d_M \cdot M \tag{16}$$

$$\dot{B_P} = L \cdot \sigma_V \cdot (1 - \sigma_V^S) \cdot B_{LV} - d_{B_P} \cdot B_P \tag{17}$$

$$\dot{T_r} = L \cdot \sigma_V \cdot (1 - \sigma_V^S) \cdot T_{rV} - d_{T_r} \cdot T_r \tag{18}$$

$$\dot{Ig_A} = p_{Ig_A} + \gamma \cdot B_P - d_{Ig_A} \cdot Ig_A \tag{19}$$

We calibrated the model using data reported by Lusuardi et al. (1993) who studied IgA levels in bronchoalveolar lavage (BAL) fluids after treatment with OM-85 (Figure 7, Table 4). Note that this represent very few data points compared to the

number of degrees of freedom of our model. This is why, we validated this calibration using data reported by Danek et al. (1996)[33] who used a treatment regimen different from the one in Lusuardi et al. (1993) (Figure 8).

Impact of age

The immune system evolves throughout life with major differences between infants, young children, adults and elderly and as such, age is a determinant factor for susceptibility to respiratory tract infections (and potentially response to OM-85 treatment). The increased susceptibility of infants and young children to respiratory infections is the result of the physiological immaturity of components of the systemic and local immune responses and, possibly, of suboptimal complex crosstalk between microbiota and immune system effectors[34]. For instance, neutrophils from neonates exhibit defective bactericidal activity and the lower DC efficiency contributes to sustain the early at birth Th2 bias, related to elevated intrauterine IL-4 and IL-10 production[34,35].

To account for this immune system maturation, we implemented an age-dependent modulation of a subset of immune-related parameters (e.g. E_O, p_L) of the following form:

$$p(x) = p^* \cdot \frac{1+\alpha \cdot e^{-k(x-a)}}{1+e^{-k(x-a)}} \tag{20}$$

$p(x)$ Parameter value as a function of age in years
x Age in years
p^* Reference parameter value for an adult
α Maximum (relative) decrease of parameter value due to immature immune system
k Immune maturation slope
a Immune maturation inflection point

The set of impacted parameters includes: E_O for activation of DCs by OM-85, p_L for recruitment of virus-specific T cells and p_{ig} for production of virus-specific immunoglobulins. We calibrated the parameters controlling age-dependency in order to reproduce the age-dependent distribution of number of RTIs observed in the COPSAC2000 birth cohort[36] (Figure 3) and obtained $k = 2$ and $a = 3$ years for the three impacted parameters and $\alpha = 0.5$ for DC activation and $\alpha = 0.85$ for the two other mechanisms.

Interface of between host and within host model of viral infection

The time-dependent solution of the SIRS model corresponds to the instantaneous prevalence of RTI caused by any of the described viruses. We utilize the time-modulation of the prevalence to model the probability density $p(t)$ of getting exposed to an RTI provoking virus at time t for a representative (mean) individual patient. We assume that any number of RTIs needs an equal or bigger number of exposures to viruses causing the infection and that the intrinsic properties of a patient (notably his immune system) will determine how many of these exposures will lead to a detectable RTI. For this, we set the threshold to declare an infection clinically detectable to 20% on the proportion of infected cells. First, we follow a Monte Carlo-like process to determine potential exposure time points during the simulation (24 bins per year, amounting to maximum of 48 potential exposures over 2 years simulation). We define the acceptance criterion $W(ex)$ as:

$$W(ex) = \begin{cases} True \text{ if } ex \leq p(t) \\ False \text{ if } ex > p(t) \end{cases} \tag{21}$$

We denote ex to be the potential exposure, which is randomly drawn from a (scalable) uniform distribution $U_{[0,\gamma]}$, and is compared to the instantaneous probability density of exposure in each bin. An individual is exposed to a respiratory virus if the acceptance criterion (21) is evaluated as True. Following this procedure, the distribution of the number of exposures in a given time period $[t_0, t_A]$ resembles a (truncated) Poisson distribution with parameter $\lambda = \gamma \frac{\int_{t_0}^{t_A} p(t)dt}{\int_0^{1year} p(t)dt}$.

The deterministic immunological model transforms exposures into RTIs and the value of γ can be adapted so that the combined deterministic-stochastic model can reproduce the expected mean number of RTIs in a specific period for a reference population. The number of RTIs for individual patients and subgroups will also depend on the state of their immune system. In the within-host model, the individual immune system states of patients are characterized by inter-patient and inter-observation variability, both following a random statistical model around an average value and consequently, there are additional parameters determining the fate of each individual exposure described above. In fact, the mean and variance of the distribution of the immune system's state related parameters determine the transformation function between the distribution of exposure

(Poisson-like) counts and the distribution of RTI counts, so that we conveniently fix γ to a number of 10 (large enough to encompass a wide range of behaviors in terms of number of RTIs per year), and calibrate only the random immune system mean state (immuno-competence) distribution so that the cumulative annual RTI count is matched to a reference situation (COPSAC2000 birth cohort[36]) (Figure 3). Note that, additionally, this procedure is done as a function of patient age with the immune effector function varying according to a sigmoid developmental dynamics model (Supplementary Methods: Impact of age).

Susceptibility of respiratory viruses to lower or upper tract

During the previously described Monte-Carlo-like process, the exposures were due to the main respiratory viruses - either RSV, rhinovirus or influenza. At each time point t_i, an exposure ex_i is triggered and the corresponding viral trigger is chosen between the three viruses according to its current prevalence w.r.t. the seasonality. This means that the viral pathogen is chosen stochastically via the contribution of the specific seasonality of the virus, $p_{virus}(t_i)$ to the total seasonality of the three respiratory viruses $p(t_i)$ at the time point of exposure t_i. In a second step, if this exposure is converted to an RTI according to the combined deterministic-stochastic model, then we choose, again stochastically, whether an LRTI or URTI is provoked according to the viral affinity. We consider for each virus its unique probability to provoke a URTI conditioned on an RTI development (with the equivalent for LRTI stimulation being the complementary probability), which is used for this stochastic approach.

Calibration of OM-85 clinical efficacy

We performed the calibration of OM-85 clinical efficacy on top of the calibrated multi-scale RTI disease model. The unknown parameters left calibrated are those controlling the up-regulation of lymphocytes activation and virus-specific immunoglobulins production due to innate memory-like cells migrating from activated Peyer's Patches. Our main data source regarding OM-85 efficacy is the meta-analysis by Yin et al. (2018)[37] who performed a systematic review of 53 RCTs of its effect in recurrent RTI involving 4851 pediatric patients and reported frequency of RTIs in OM-85 vs. the control group. Their analysis showed that OM-85 was positively correlated with a reduction in the frequency of respiratory infection compared to the control group.

We grouped the studies into either 12 months or 6 months of follow-up and considered only studies with a single course of treatment and excluded all others. A 2D analysis of the absolute benefit as a function of the RTI frequency in the control group, similar to the Effect Model law[38] (Figure 9) confirms that the effect of OM-85 is in fact not a constant, but depends non-linearly on the risk for RTI (which explains that studies preferably enroll patients at risk for recurrent RTI). Selection of the appropriate at-risk population is usually done by evaluating the frequency of RTI in a reference period, assuming that immunological characteristics drive the risk for RTI and thus their frequency is correlated in consecutive years. This situation was mimicked by in silico clinical trials comprising an observational period and a follow-up period. We matched the simulated treatment efficacy distribution by small in silico clinical trials of 25 patients per arm with a pediatric population of 1 to 6 years of age while varying patients' eligibility criteria regarding their number of RTI in the observational period. The results of efficacy as a function of eligibility criteria (Figure 11-10) indicate in fact that tuning the number of required RTI in the observational period can navigate through the entire meta-analyzed risk-stratified efficacy of the meta-analysis (parameters values controlling treatment efficacy were chosen from a series of simulations so that coverage the meta-analysis by the entire in silico procedure was maximal).

Mechanistic uncertainty management

To account for the substantial uncertainty regarding the mechanism determining the effect of OM-85 (through gut-reprogrammed innate cells that convey pro-type 1 immunomodulation in respiratory tract mucosal tissue), we simulated different mechanistic scenarios for the following key parameters in parallel as part of uncertainty management.

The first key but uncertain mechanism is the antigen presenting cell i.e. dendritic cell (DC) sensitivity to the concentration of the immunogenic compound, which depends on the administered dose. Indeed, human in vivo OM-85 dose-effect relationship data regarding the immune activation have not yet been reported. We used human in vitro data and apply an in vitro to in vivo translation factor. Since no data was available to set this factor, we included four different values (very low: 0.01, low: 0.05, medium: 0.1 and high: 0.2) for the DC sensitivity to OM-85 dose around a typical values of 0.1[39].

Trained innate immunity postulates that immune cells can alter their gene expression for a longer time period after encountering an inflammatory stimulus and that they can partially persist in the organism and confer protection against a secondary stimulus[40,41]. Various in vitro, rodent models and clinical data suggest that exposure to OM-85 may generate generate such trained innate immune cells[42] enhancing a pre-alert anti-infectious RT-tissue state. This mechanism represented in our model through long-lived innate memory-like progenitor cells that can differentiate into type-1 innate immune cells. The lifetime of such postulated OM-85 stimulus-reprogrammed cells is unknown. We included three lifetimes: short (30 days), medium (60 days) and long (90 days).

The final model ensemble is then constructed with the Cartesian product of the set of values for the two key mechanisms and sensitivity of main results are presented taking into account that uncertainty (Figure 12, Figure 14). Absolute benefit is

consistent per NPI scenario throughout the range of mechanistic hypotheses whereas ERR and consequently sample size and recruitment times are more impacted with the strong NPI scenario. Interestingly, lifetime of type-1 activated innate immune cells is the most determinant factor where short-lived cells reduce the impact of the strong NPI scenario compared to long-lived ones.

Sensitivity analysis of the within-host model

To assess the within-host model robustness and uncertainty, we performed a variance-based global sensitivity analysis[43] on the viremia peak (AUC) with respect to variation of the 13 calibrated parameters of the within-host viral infection disease model around their calibrated value ($\pm$25%). The resulting sensitivity indices provide information on the parameters' global influence with respect to their contribution to the variance of the model output, including the effect of interactions among parameters. The computation of the sensitivity indices relies on a variance analysis (ANOVA), which is based on the hypothesis that the response (viremia peak here) is explained by a linear model of the mechanistic model parameters. Hence, three hypotheses have to be met: independence of the observation, and the normality and homoscedasticity. As a consequence, the parameter combinations explored by the sensitivity analysis have to respect these hypotheses. A class of fractional factorial designs is particularly well adapted in this case: regression model-based optimal design, which optimizes the parameter combinations for a selected regression model (linear in our case). We thus used a fractional factorial design of $3^7 = 2187$ samples from a 3-level design and obtained by Monte Carlo simulations where 10,000 random partial designs are generated and reduced using the Federov algorithm. We repeated the procedure 5 times and the best design was kept. We ran the 2187 simulations with this design for a single viral exposition event at t=0 and analyzed the sensitivity of the viremia peak to the corresponding design. Global sensitivity indexes ranked by increasing impact are presented in Figure 13a and distribution of relative deviation from the mean of vireamia AUC in Figure 13b. Percentage of total variance is high (96%) and second order interactions only represent a negligible part. Four parameters come out as equally most influential: lysis rate of infected cells, immune-activation by the virus, lymphocyte development rate and virions production rate. This indicates that calibration should focused on these parameters if the model is to be adapted to new viremia data (including different viruses for example) and that experimental efforts could be directed at better informing those parameters to increase robustness in such viral infection models. These results where parameters related to how well the virus replicates and how efficient the immune system is at clearing infected cells are found the most influential are consistent with what was reported with a model of viral dynamics for influenza[44].

Supplementary Figures and Tables

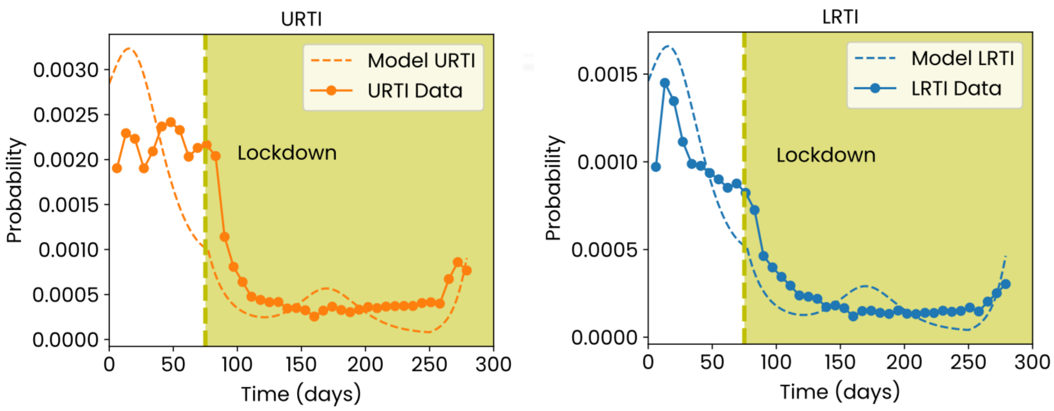

Supplementary Figure 1. Validation of modeling approach of RTI burden under NPI measures using RCGP data[45]. Comparison between simulations (dashed line) and RCGP data (solid line) on percentage of the population with either an upper (left panel) or a lower RTI (right panel). The simulation starts at 01/01/2020, the lockdown is introduced at 75 days (mid-March) and is modeled by a transmission decreased by 17.5%.

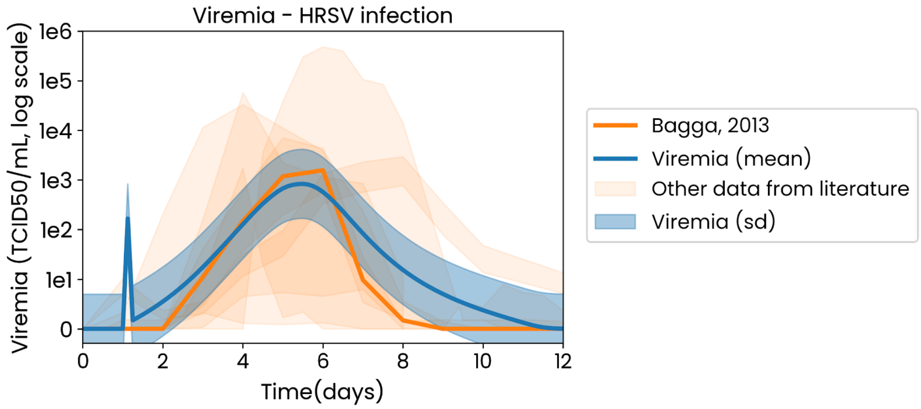

Supplementary Figure 2. Viral load kinetics following experimental HRSV infection in adults. Experimental data (orange) was extracted from Bagga et al.[3]. Simulations are indicated in blue (mean and standard deviation). Virtual patients were challenged on day 1 with a viral dose of 1000 TCID50/mL. Other dataset from literature of experimental HRSV infections are superimposed (orange, inter SD range), confirming the general form of the viremia dynamics.

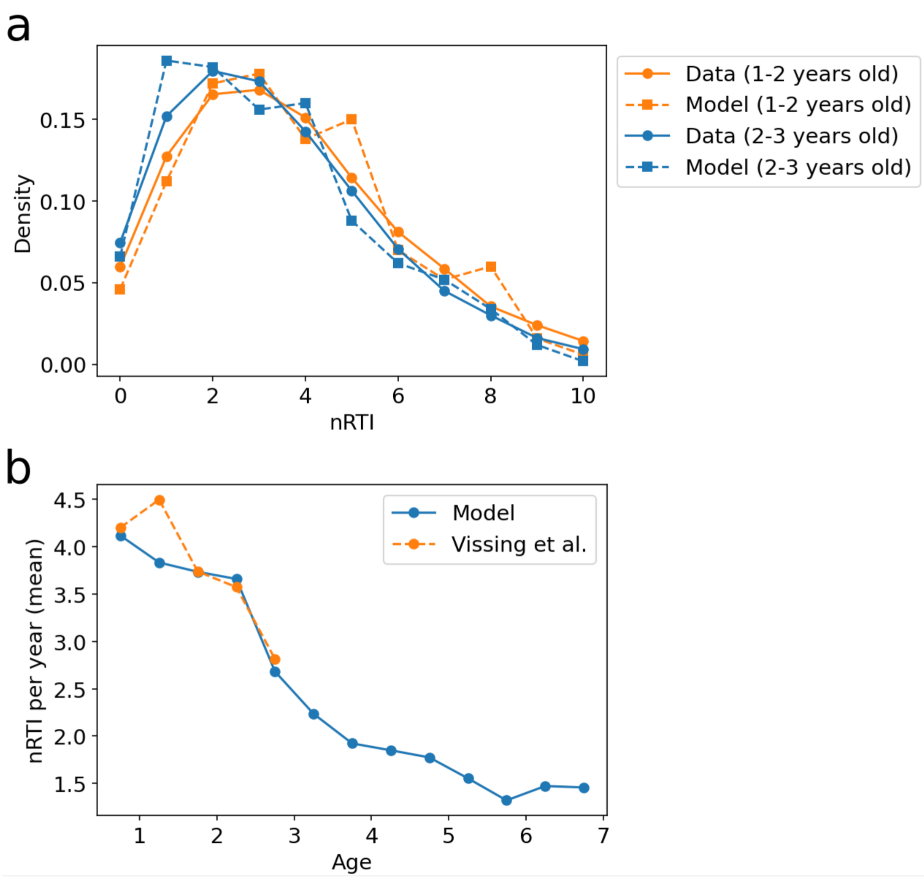

Supplementary Figure 3. Calibration of immuno-maturation in the within-host model. **a**, Negative binomial distributions were fitted (solid lines) to match the distribution of number of RTIs (nRTI) from the COPSAC2000 cohort extracted from Vissing et al.[36] (median, mean and IQR for age 1-2 and 2-3 years old). Model parameters controlling immuno-maturation in the within-host RTI model were calibrated to reproduce this age-dependent distribution. Here a representative virtual cohort of similar size as the COPSAC2000 cohort (334 children) was simulated for two age groups (1-2 and 2-3 year old in orange and blue resp.) over one year. Distribution of cumulative number of RTIs for each age group is plotted (dashed lines). **b**, Number of RTIs per year per age is plotted for the model (solid blue line) for children from 0.5 to 7 years old and compared with data from the COPSAC2000 cohort[36] (dashed orange line).

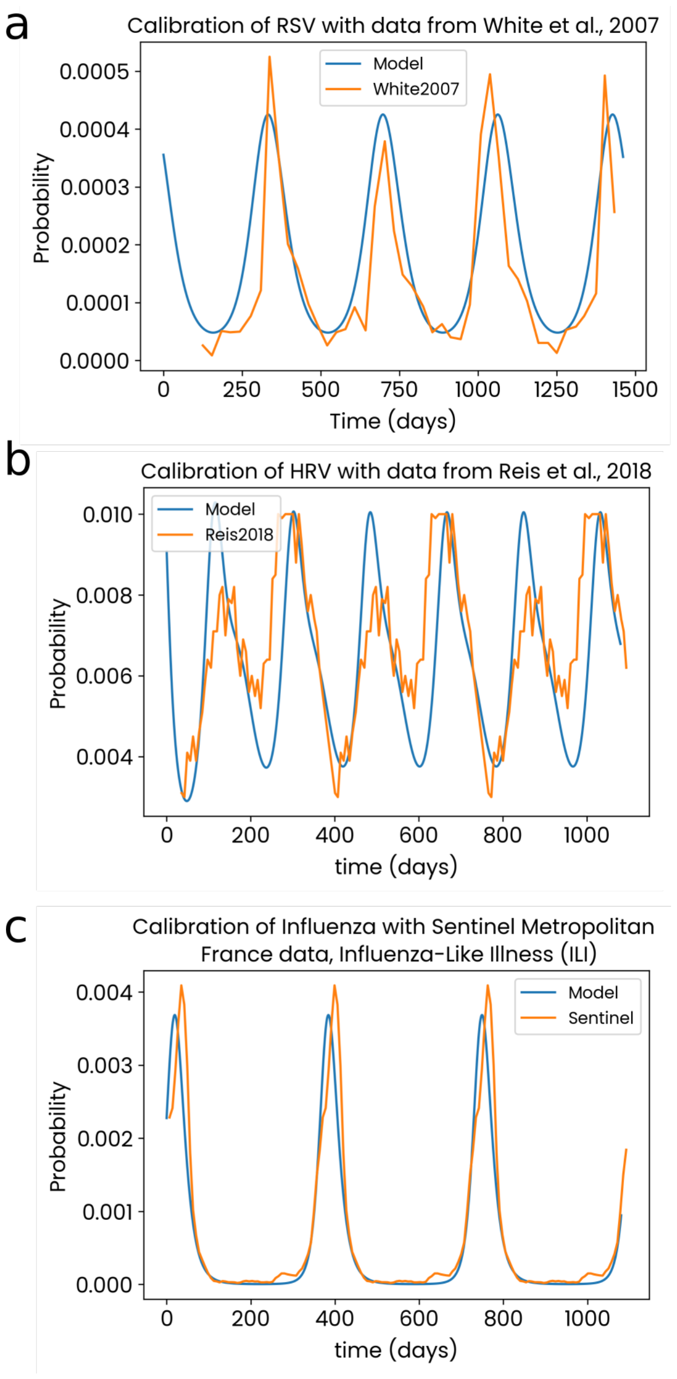

Supplementary Figure 4. Calibration of the three parallel between-host SIRS models for: **a**, respiratory syncytial viruses (RSV) with data from White et al. (2007)[12]; **b**, rhinoviruses (HRV) with data from Reis et al. (2018)[46] and **c**, influenza viruses with data from Sentinel network in France[14]. Data (orange) over one year was replicated to extend the range of the comparison between simulations (blue) and data to several years when needed. Time starts January, 1st.

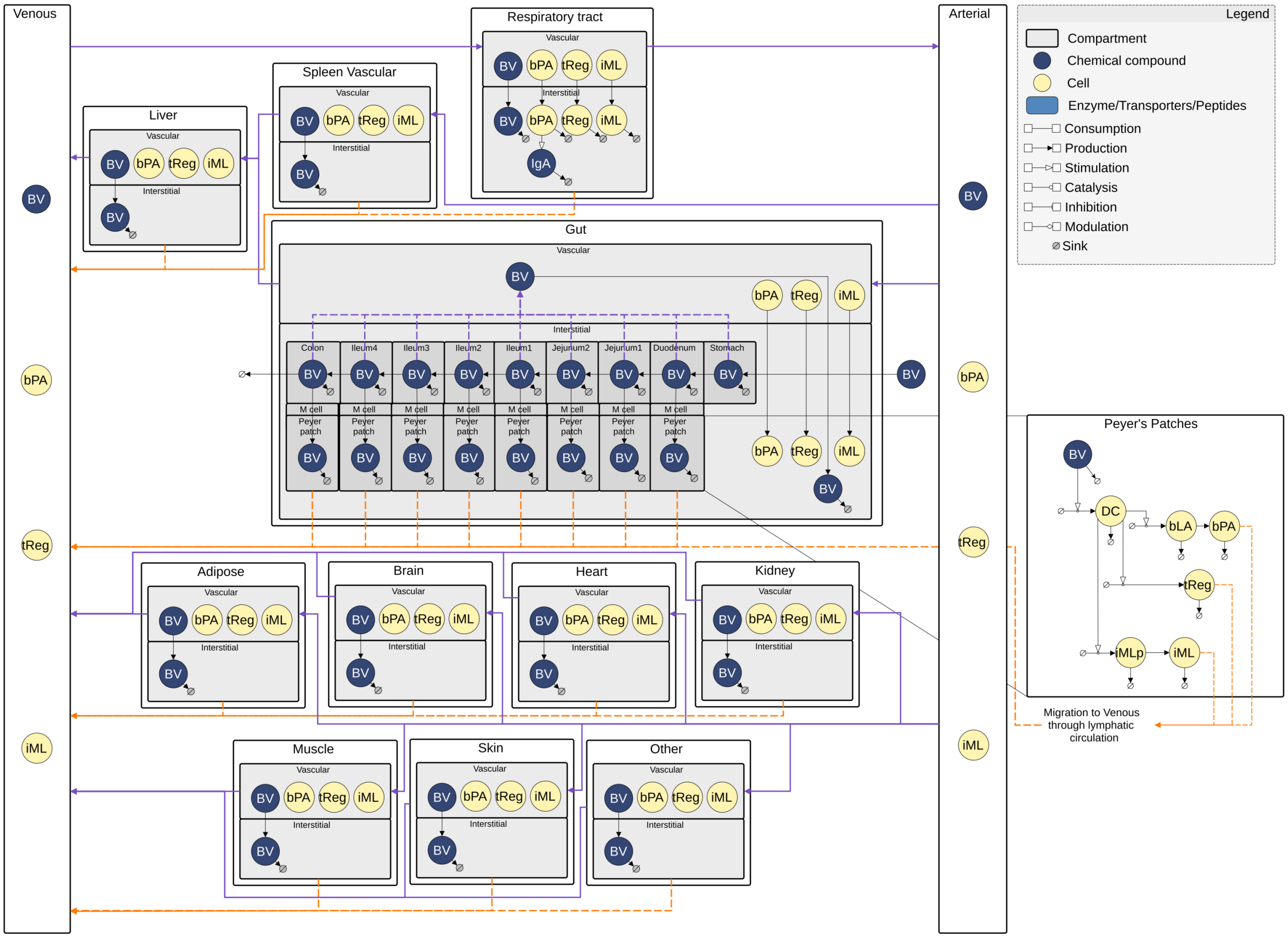

Supplementary Figure 5. Graphical representation of the PBPK/PD model used for the treatment model. A detailed absorption model is also represented notably for permeation via M cells to Peyer's Patches. BV = Broncho-Vaxom i.e. OM-85. The systemic circulation is indicated with purple arrows and the lymphatic circulation with orange arrows.

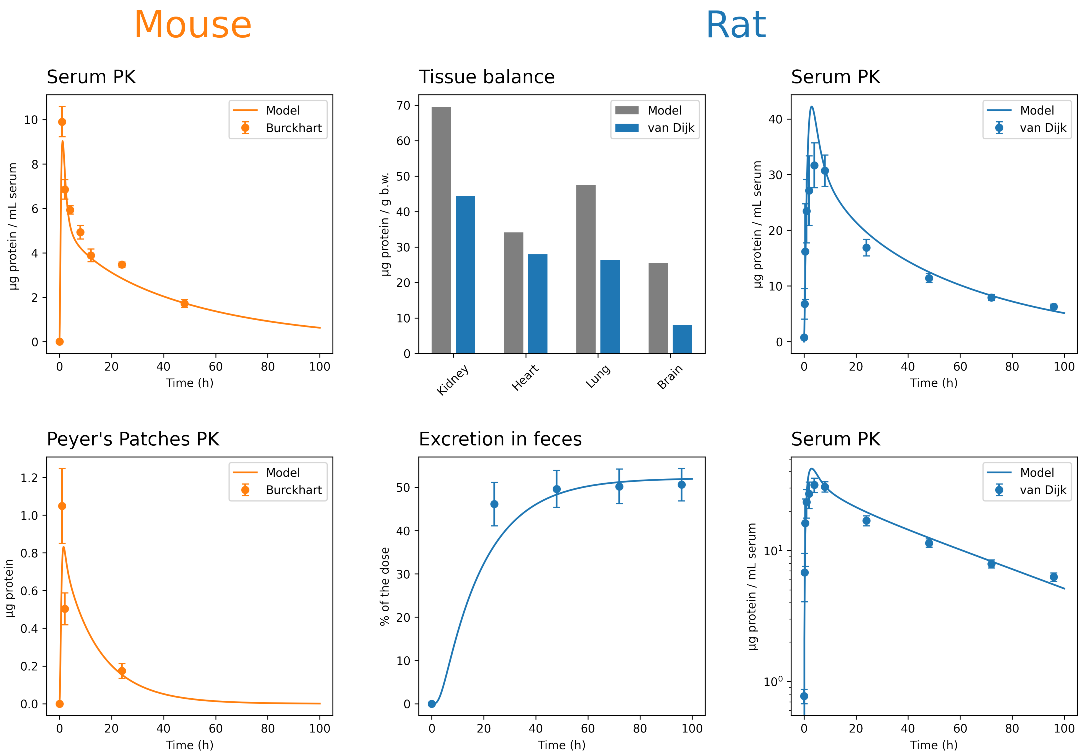

Supplementary Figure 6. Calibration of the PBPK model with PK data on a radiolabeled derivative of a product (OM-89) similar to OM-85 in rodents[23,24]. We built a inter-species PBPK model by allometric scaling and species-specific parameters to use these data for calibration. Simulations and experimental data in mice are indicated in orange (first column) whereas simulations and experimental data in rats are in blue (last two columns). Simulations are either plotted with a solid line or as blue bars in the bar plot. Experimental data[23,24] are indicated either as grey bars in the bar plot or as points with error bars (mice: mean ± SEM, n=5; rats: mean ± SD, n=5

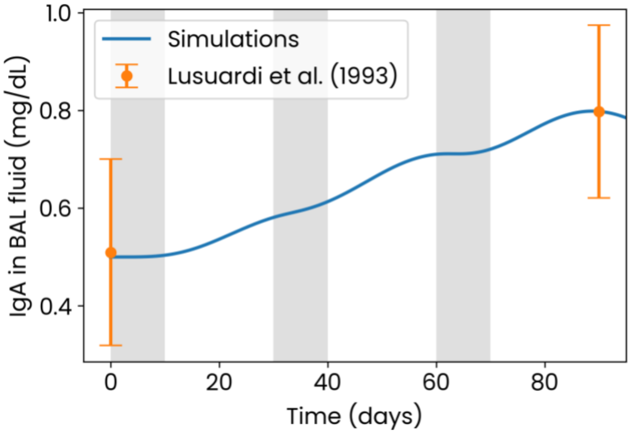

Supplementary Figure 7. Final parametrization selected to reproduce the IgA dynamics in BAL fluid as reported by Lusuardi et al. (1993). Virtual patient is treated with three 10-day courses of OM-85 (light grey vertical bars). Simulation is plotted with a blue solid line whereas data is indicated as orange points with error bars (mean ± SEM, n=10).

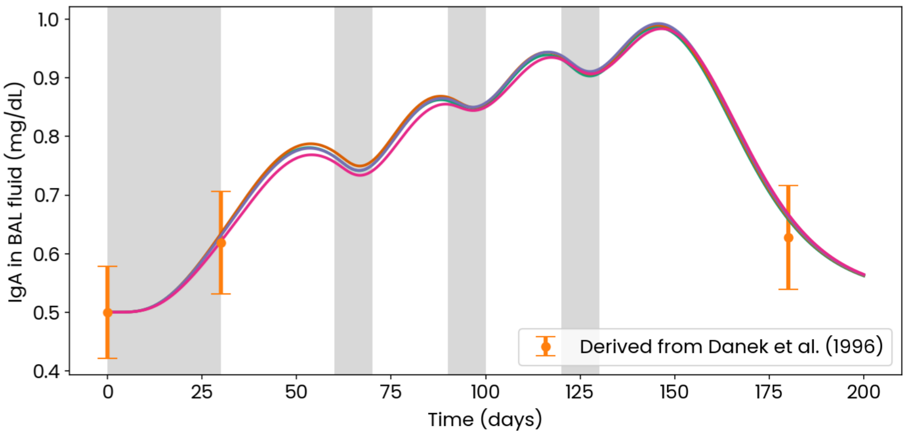

Supplementary Figure 8. Validation data on IgA concentration in BAL fluid (mg/dL) derived from Danek et al. (1996)[33] vs simulations. Regimen: One month of daily administration followed by one month without treatment followed by 10 days of daily administration at the beginning of the month for 3 months. Phases of daily administration are marked in shaded grey. Note that simulations are reported here for the four different hypotheses on sensitivity to the dose of GALT sensor cells (professional antigen-presenting-cells) as described in the Methods. Simulation is plotted with a solid line whereas data is indicated as orange points with error bars (mean $\pm$ SD, n=24).

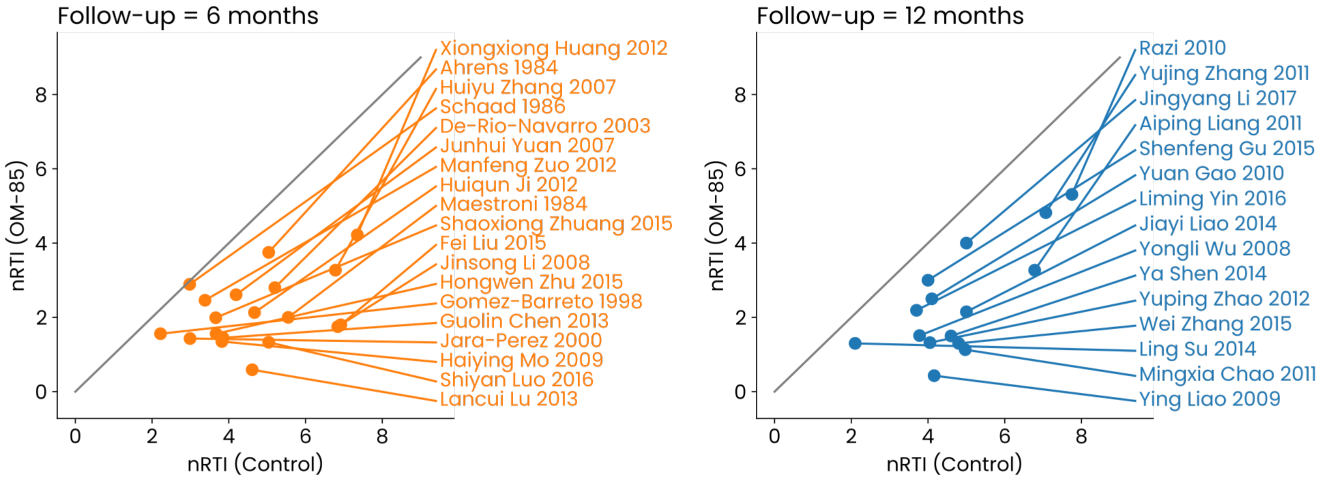

Supplementary Figure 9. 2D analysis of OM-85's absolute benefit (number of prevented RTIs) as a function of the RTI frequency in the control group (similar to the Effect Model law[38]) using data from the meta-analysis by Yin et al. (2018)[37]. The analysis is splitted according to follow-up duration: 6 months (left, orange) or 12 months (right, blue).

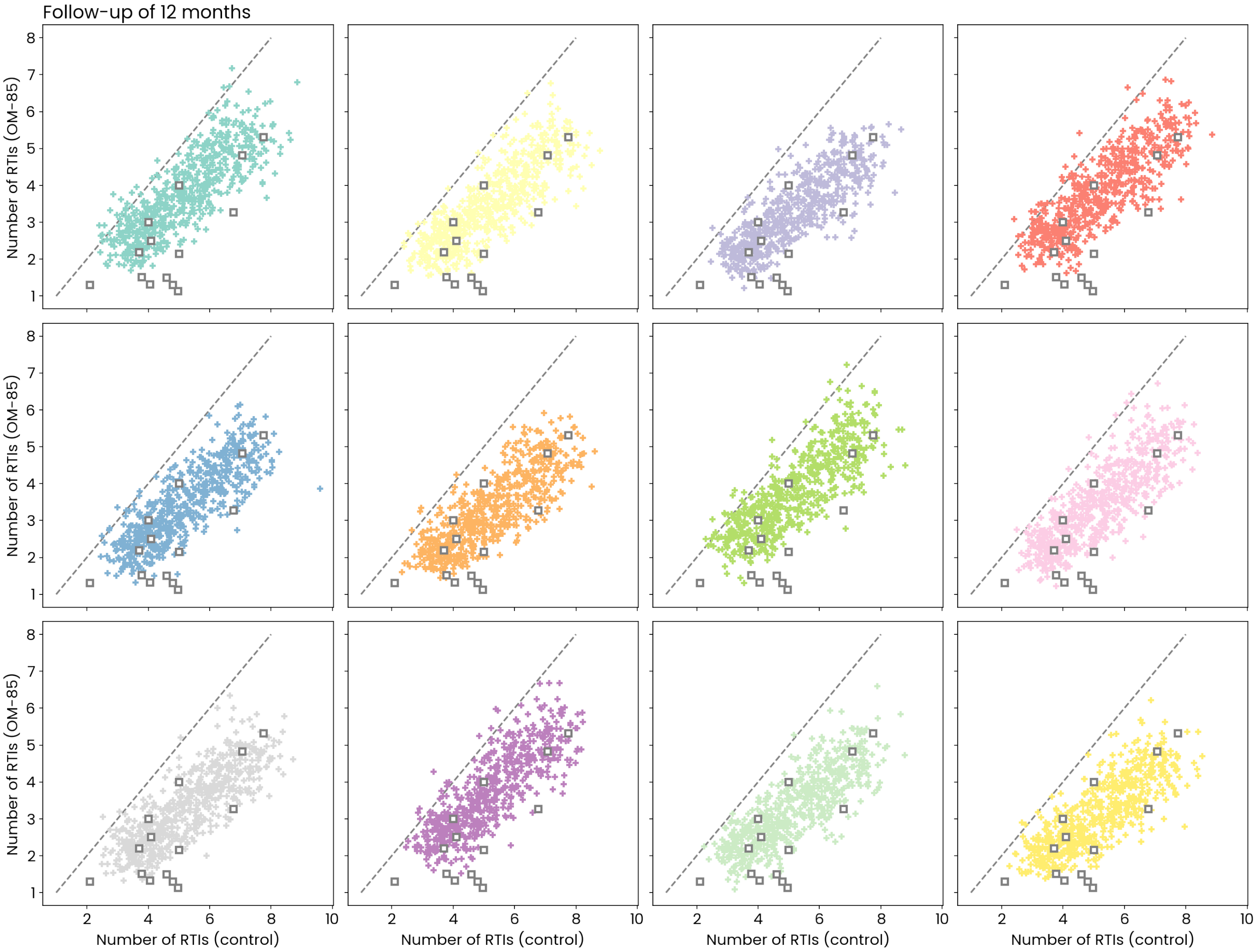

Supplementary Figure 10. Comparison of OM-85's simulated efficacy with the data from the meta-analysis by Yin et al. (2018)[37] for a population of pediatric patients (1-6 years old) for a regular treatment of 10 daily administrations (3.5 mg) per month for 3 months followed by 12 months of follow-up. Here, for each model variation (defined by the colors, see Methods and Figure 12), we conducted 50 in silico trials with 50 patients per arm for various inclusion criteria (defined as the number of RTIs in the selection year prior to treatment) going from 0 up to 12 RTIs per year. For each of these trials, we report the mean number of RTIs during the follow-up period (12 months) in the control group vs the treated group (crosses). Points (squares) corresponding to real clinical trials obtained from the meta-analysis are overlayed.

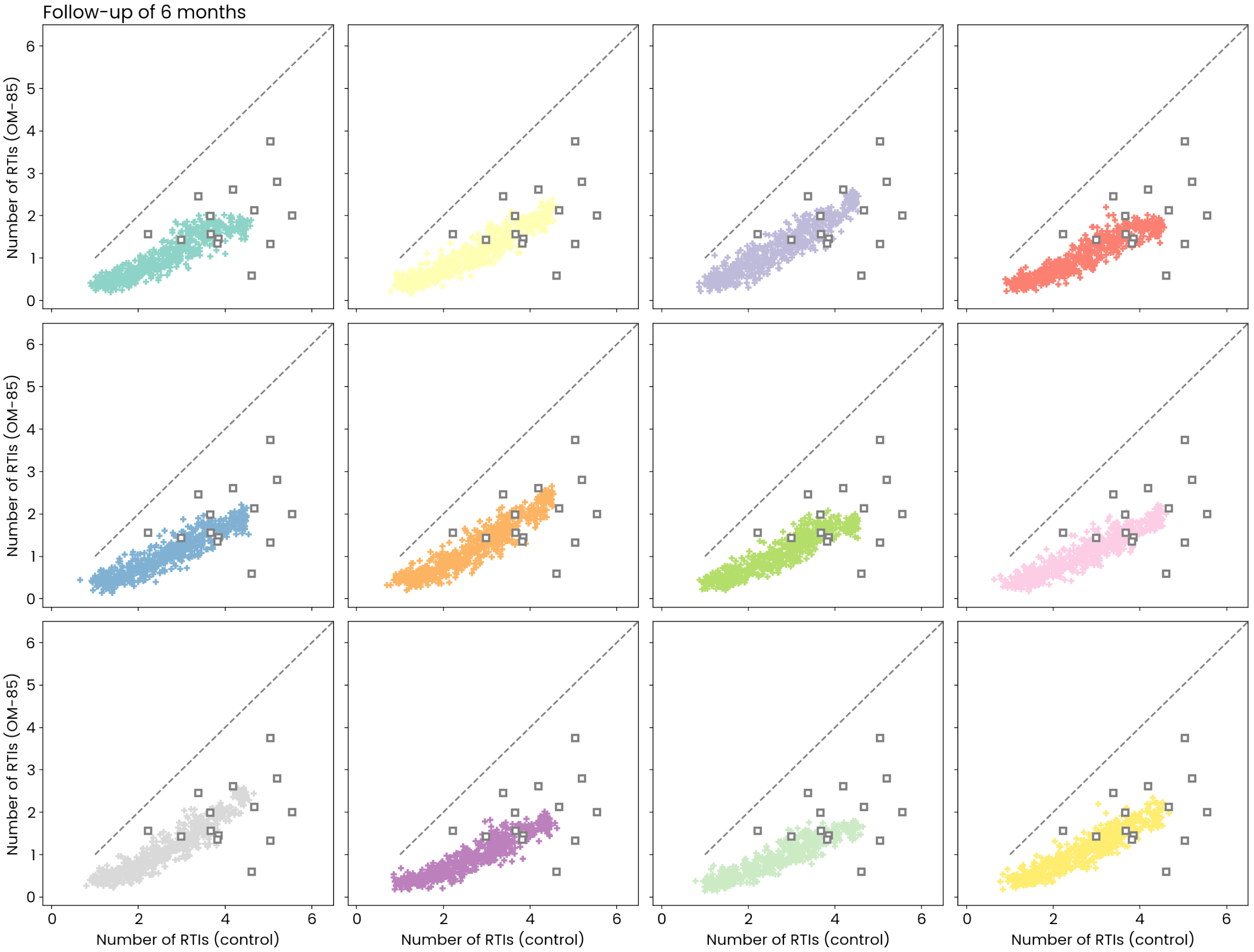

Supplementary Figure 11. Comparison of OM-85's simulated efficacy with the data from the meta-analysis by Yin et al. (2018)[37] for a population of pediatric patients (1-6 years old) for a regular treatment of 10 daily administrations (3.5 mg) per month for 3 months followed by 6 months of follow-up. Here, for each model variation (defined by the colors, see Methods and Figure 12), we conducted 50 in silico trials with 50 patients per arm for various inclusion criteria (defined as the number of RTIs in the selection year prior to treatment) going from 0 up to 12 RTIs per year. For each of these trials, we report the mean number of RTIs during the follow-up period (6 months) in the control group vs the treated group (crosses). Points (squares) corresponding to real clinical trials obtained from the meta-analysis are overlayed.

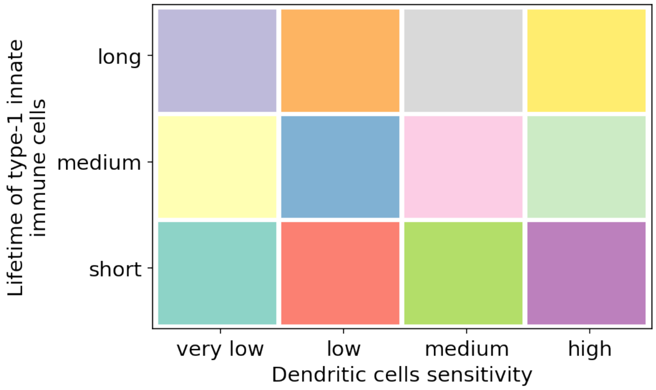

Supplementary Figure 12. Color code for the different combinations of the two hypotheses on dendritic cells sensitivity (x-axis; very low: 0.01, low: 0.05, medium: 0.1 and high: 0.2) and on lifetime of type-1 innate immune cells (y-axis; short: 30 days, medium: 60 days, long: 90 days

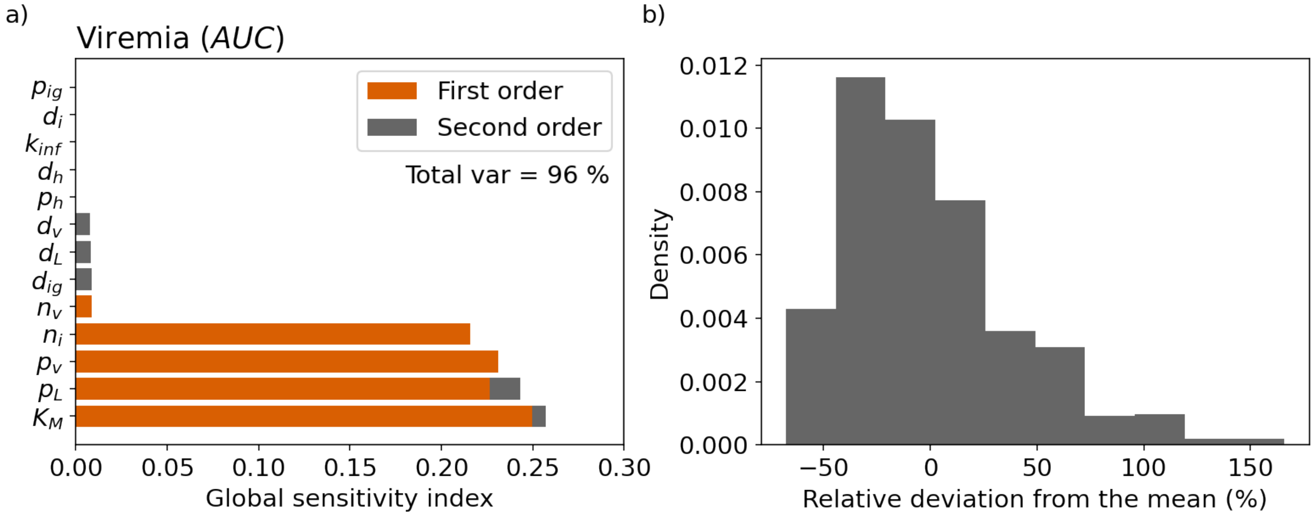

Supplementary Figure 13. **Sensitivity analysis (within-host model). a)** Global sensitivity indexes (first (orange) and second (grey) order interactions) for variance-based global sensitivity analysis on the viremia peak (AUC) with respect to variation of the 13 calibrated parameters of the within-host viral infection disease model around their calibrated value ($\pm 25\%$) using a fractional factorial design of $3^7 = 2187$ samples from a 3-level design and obtained by Monte Carlo simulations. **b)** Histogram (density) of relative deviation to the mean of viremia peak AUC in percentage.

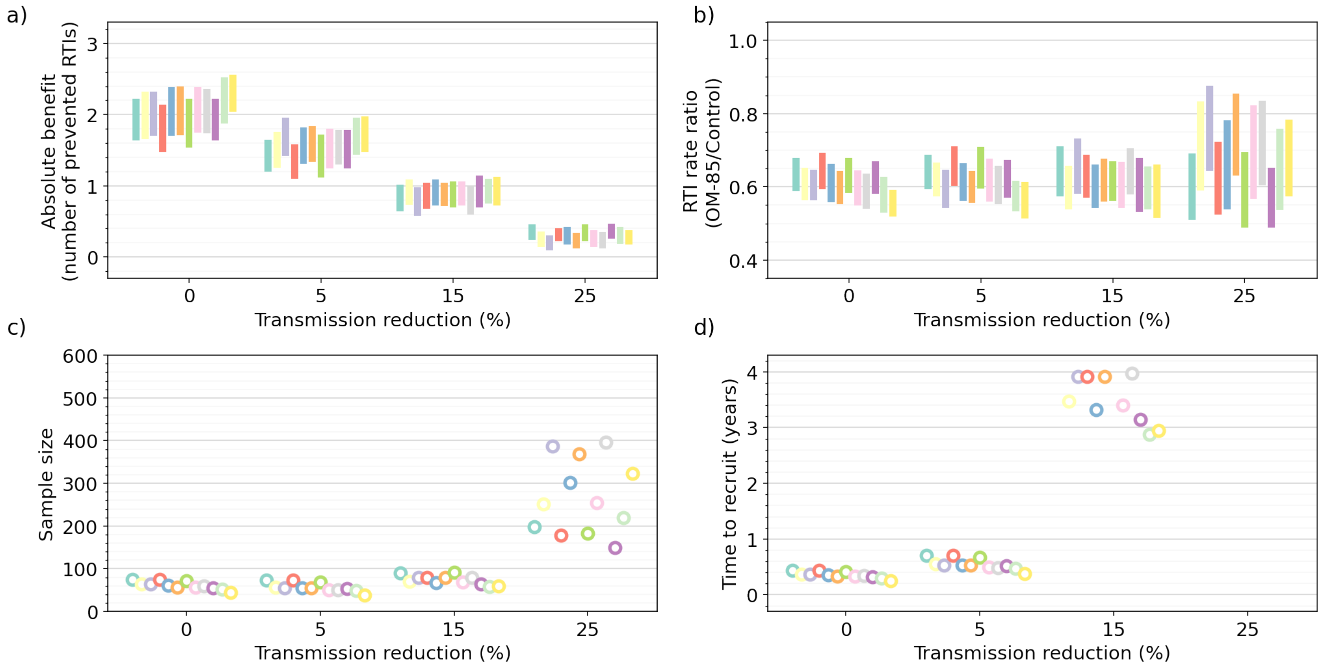

Supplementary Figure 14. Sensitivity of main results to mechanistic uncertainty. For this, we simulated different mechanistic scenarios in parallel. We used 12 different conditions (testing different immunogenic hypotheses on the effect of OM-85, Supplementary Methods: Mechanistic uncertainty management) for each of the 4 non-pharmaceutical interventions (NPIs) scenarios and pooled the results. Each mechanistic scenario is color coded (Figure 12). Similarly to Figure 3 of main text, we ran in silico clinical trials with 4 scenarios of NPIs with increasing strength (absent, mild, medium and strong) modeled by a decrease of the transmission rate parameter (no reduction, -5%, -15% and -25%, respectively). There is no NPI during year 1. The NPIs are started at the beginning of year 2 as well as the treatment (10 daily administrations of 3.5 mg of OM-85 from the beginning of the month for 3 consecutive months). Number of RTIs are counted for the complete duration of year 2. For the 12 mechanistic scenarios and for the 4 NPI scenarios, we report: **a)** the distribution (IQR) of absolute benefit; **b)** the distribution (IQR) of event RTI rate ratio (ERR, treated over control group); **c)** the distribution (IQR) of sample sizes per arm required to show efficacy of OM-85 treatment in reducing the number of RTIs; **d)** the distribution (IQR) of estimated patient screening times assuming an hypothetical screening rate of 1,000 patients per year and by taking year 2 as the selection year (without treatment).

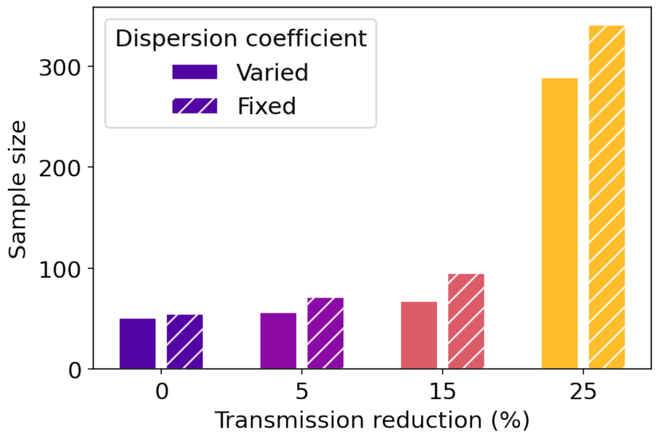

Supplementary Figure 15. Sensitivity of sample size (per arm) to using a dispersion parameter in the calculation either fixed or varied per NPI scenario. Sample size calculations was performed as in Zhu et al. (2013)[47] (see Methods). For this, we conducted generalized linear regression analysis on number of RTIs with negative binomial distributions to get the mean and dispersion parameter for each NPI scenario and for each arm (placebo and treated). We then either used the dispersion parameter of the placebo arm from the scenario without reduction of transmission (Fixed, hatched bars) or the average dispersion parameter between the two arms per NPI scenario (Varied, solid bars).

Supplementary Table 1. Parameters (description and value) of the within-host RTI disease model

Parameter	Value	Unit	Description	Reference(s)
n_i	2.7	$\mathrm{mL\,d^{-1}\,kcell^{-1}}$	Lysis rate of infected cells by lymphocytes	Calibrated
d_i	0.26	$\mathrm{d^{-1}}$	Rate of infected cell death	Calibrated
d_h	0.01	$\mathrm{s^{-1}}$	Rate of healthy cell death	Calibrated
p_h	1	$\mathrm{d^{-1}}$	Epithelium renewal rate (percentage)	Calibrated
d_v	$2 \cdot 10^{-6}$	$\mathrm{d^{-1}}$	Rate of viral particles decay	Calibrated
k_{inf}	0.11	$\mathrm{mL\,d^{-1}\,kcell^{-1}}$	rate of viral infection of healthy cells	Calibrated
v_f	1	-	Virulence factor	Varied per virus type
c	10	$\mathrm{count\,mL^{-1}}$	Threshold under which the viral load will not affect the healthy cells (represents the preemptive clearance by the innate immune system)	Fixed
p_v	2.58	$\mathrm{d^{-1}}$	Production rate of viruses by the infected cells	Calibrated
p_{ig}	3177	$\mathrm{kcount\,mL^{-1}\,ms^{-1}}$	Virus-specific antibody production	Calibrated
d_{ig}	0.22	$\mathrm{d^{-1}}$	Decay of virus-specific antibodies	Calibrated
S	3.78	-	Immune state of the patient	Distributed in the population
K_V	608	$\mathrm{kcount\,mL^{-1}}$	Virus-mediated activation of the immune system	Calibrated
p_M	4	-	Parameter upregulating the activation of the lymphocytes based on the innate memory-like cells resulting from GALT	Calibrated
K_M	4.64	$\mathrm{cell\,mL^{-1}}$	Modulates the activation of lymphocytes and IgA based on the innate memory-like cells resulting from GALT	Calibrated
n_v	10.7	$\mathrm{\mu L\,d^{-1}\,Mcount^{-1}}$	Neutralization rate of viruses by the antibodies	Calibrated
p_L	36.5	$\mathrm{kcell\,mL^{-1}\,d^{-1}}$	development rate of lymphocytes in response to infected cells	Calibrated
d_L	0.11	$\mathrm{d^{-1}}$	Death rate of the lymphocytes	Calibrated
$\bar{E}_h$	11.63	$\mathrm{Mcell\,mL^{-1}}$	Equilibrium concentration of healthy cells in the epithelium	Fixed
d_{Ig_A}	0.12	$\mathrm{d^{-1}}$	IgA decay rate	Fixed[48]

Supplementary Table 2. Parameters (description and value) of the between-host infection transmission model

Parameter	Value	Unit	Description	References(s)
$\beta_{0,RSV}$	0.219	d^{-1}	mean value of infection rate β for RSV	Calibrated
$\beta_{1,RSV}$	0.116	-	relative magnitude of seasonal oscillations of β for RSV	Calibrated
γ_{RSV}	0.1	d^{-1}	recovery rate for RSV	Fixed[10]
ζ_{RSV}	0.005	d^{-1}	rate of loss of immunity for RSV	Fixed[10]
P_{RSV}	0.274	-	phase shift of the β seasonality as a fraction of a year for RSV	Calibrated
ω_{RSV}	2π	-	frequency for seasonality oscillation of β for RSV	Fixed
$\beta_{0,HRV}$	1.118	d^{-1}	mean value of infection rate β for (human) rhinovirus	Calibrated
$\beta_{1,HRV}$	0.072	-	relative magnitude of seasonal oscillations of β for (human) rhinovirus	Calibrated
γ_{HRV}	0.5	d^{-1}	recovery rate for (human) rhinovirus	Fixed[49]
ζ_{HRV}	0.0274	d^{-1}	rate of loss of immunity for (human) rhinovirus	Fixed[49]
P_{HRV}	0.596	-	phase shift of the β seasonality as a fraction of a year for (human) rhinovirus	Calibrated
ω_{HRV}	4π	-	frequency for seasonality oscillation of β for (human) rhinovirus	Fixed
$\beta_{0,IV}$	0.591	d^{-1}	mean value of infection rate β for influenza	Calibrated
$\beta_{1,IV}$	0.101	-	relative magnitude of seasonal oscillations of β for influenza	Calibrated
γ_{IV}	0.2	d^{-1}	recovery rate for influenza	Fixed[50]
ζ_{IV}	0.00274	d^{-1}	rate of loss of immunity for influenza	Fixed[50]
P_{IV}	0.036	-	phase shift of the β seasonality as a fraction of a year for influenza	Calibrated
ω_{IV}	2π	-	frequency for seasonality oscillation of β for influenza	Fixed
A	0.5992	-	childcare influence	Calibrated
L	0.85	-	effect of a lockdown scenario	Estimated via[45]
f_{URTI}	0.661	-	fraction of URTIs in total number of RTIs	Estimated via[45]
f_{LRTI}	0.339	-	fraction of LRTIs in total number of RTIs	Estimated via[45]
N	1	-	population normalization (arbitrary number)	Fixed
t_0	1	d	normalization time of the oscillations	Fixed

Supplementary Table 3. Drug-specific parameters (description and value) of the PBPK model. Parameters are given for mice and were allometrically scaled for rats and humans.

Parameter	Value	Unit	Description
σ_{PP}	0.95	-	Reflection coefficient in Peyer's Patches for immune cells
σ_V^S	0.15	-	Scalar factor for vascular reflection coefficients
p_{PP}^{Eff}	34.4	$\mathrm{nm\,s^{-1}}$	Effective permeation of OM-85 into the PPs
p^{Eff}	376.4	$\mathrm{nm\,s^{-1}}$	Effective gut permeation of OM-85
d_{Gut}	0.29	$\mathrm{h^{-1}}$	Degradation of OM-85 in the intestinal lumen
CL	8.4	nL/ min /mg	Liver metabolic clearance of OM-85 per mg of microsomal proteins
$NSCL$	0.19	μL/ min	Non-specific clearance of OM-85 per mg of microsomal proteins

Supplementary Table 4. Parameters of the pharmacodynamics model of OM-85 immune activation in Peyer's Patches.

Parameter	Value	Unit	Description	Reference(s)
E_O	170	cell d^{-1} μL^{-1}	Saturation factor for the activation rate of dendritic cell by OM-85	Calibrated
K_O	0.12	$\mu mol\,L^{-1}$	Half saturation constant for the activation rate of dendritic cell by OM-85	Estimated from in vitro data[51]
h	3	-	Hill coefficient for the activation rate of dendritic cell by OM-85	Estimated from in vitro data[51]
E_{M_p}	550	cell d^{-1} L^{-1}	Saturation factor for the activation rate of pre-activated type 1 innate cell by OM-85 activated DCs	Calibrated
K_{M_p}	200	cell μL^{-1}	Half saturation constant for the activation rate of pre-activated type 1 innate progenitor cells by OM-85 activated DCs	Fixed
E_{B_L}	14	cell d^{-1} μL^{-1}	Saturation factor for the activation rate of IgA+ B cells by OM-85 activated DCs	Calibrated
K_{B_L}	200	cell μL^{-1}	Half saturation constant for the activation rate of IgA+ plasma cells by OM-85 activated DCs	Fixed
E_{B_P}	0.1	d^{-1}	Differentiation rate of IgA+ B cells to plasma cells stimulated by OM-85 activated DCs	Calibrated
K_{B_P}	200	cell μL^{-1}	Half saturation constant for the DC stimulated expansion of memory IgA+ B cells into IgA+ plasma cells	Fixed
E_{T_r}	10	cell d^{-1} μL^{-1}	Saturation factor for the rate of activation of tRegs by OM-85 activated DCs	Calibrated
K_{T_r}	200	cell μL^{-1}	Half saturation constant for the rate of tRegs by OM-85 activated dCs	Fixed
α	0.5	d^{-1}	Differentiation rate of reprogrammed type-1 innate progenitors in innate memory-like cells	Fixed
β	0.01	d^{-1}	Basal differentiation rate of IgA+ B cells to plasma cells	Fixed
γ	200	pg $cell^{-1}$ d^{-1}	Non-specific IgA production rate by plasma cells	Fixed[52,53]
$p_{I_{gA}}$	0.06	mg d^{-1} dL^{-1}	IgA basal production rate	Fixed[54]
d_D	0.11	d^{-1}	Death rate of OM-85 activated dendritic cell	Fixed[55]
d_{M_p}	0.01	d^{-1}	Death rate of pre-activated type 1 innate cell	Calibrated
d_M	0.06	d^{-1}	Natural decay rate of pre-activated type 1 innate cell	Calibrated
d_{B_L}	0.005	d^{-1}	Death rate of IgA+ B cells	Fixed[56]
d_{B_P}	0.23	d^{-1}	Death rate of non-specific IgA+ plasma cells	Fixed[53]
d_{T_r}	0.12	d^{-1}	Death rate of regulatory T cells	Fixed[56]
$d_{I_{gA}}$	0.12	d^{-1}	IgA decay rate	Fixed[48]

References

1. Baccam, P., Beauchemin, C., Macken, C. A., Hayden, F. G. & Perelson, A. S. Kinetics of influenza a virus infection in humans. *J Virol* **80**, 7590–7599, DOI: 10.1128/jvi.01623-05 (2006).
2. Smith, A. M. Host-pathogen kinetics during influenza infection and coinfection: insights from predictive modeling. *Immunol Rev* **285**, 97–112, DOI: 10.1111/imr.12692 (2018).
3. Bagga, B. *et al.* Comparing influenza and rsv viral and disease dynamics in experimentally infected adults predicts clinical effectiveness of rsv antivirals. *Antivir. Ther.* **18**, 785–791, DOI: 10.3851/imp2629 (2013).
4. Hansen, N., Müller, S. D. & Koumoutsakos, P. Reducing the time complexity of the derandomized evolution strategy with covariance matrix adaptation (cma-es). *Evol. computation* **11**, 1–18 (2003).
5. M"uller, J. & Kuttler, C. Methods and models in mathematical biology. *Lect. Notes on Math. Model. Life Sci.* DOI: 10.1007/978-3-642-27251-6 (2015).
6. Eggo, R. M., Scott, J. G., Galvani, A. P. & Meyers, L. A. Respiratory virus transmission dynamics determine timing of asthma exacerbation peaks: Evidence from a population-level model. *Proc. Natl. Acad. Sci.* **113**, 2194–2199, DOI: 10.1073/pnas.1518677113 (2016).
7. Yu, J. *et al.* Comparison of the prevalence of respiratory viruses in patients with acute respiratory infections at different hospital settings in North China, 2012-2015. *BMC Infect. Dis.* **18**, DOI: 10.1186/s12879-018-2982-3 (2018).
8. Pattemore, P. K. & Jennings, L. C. Epidemiology of Respiratory Infections. *Pediatr. Respir. Medicine* 435–452, DOI: 10.1016/b978-032304048-8.50035-9 (2009).
9. Weber, A., Weber, M. & Milligan, P. Modeling epidemics caused by respiratory syncytial virus (RSV). *Math. Biosci.* **172**, 95–113, DOI: 10.1016/s0025-5564(01)00066-9 (2001).
10. Acedo, L., Díez-Domingo, J., Moraño, J.-A. & Villanueva, R.-J. Mathematical modelling of respiratory syncytial virus (rsv): vaccination strategies and budget applications. *Epidemiol. Infect.* **138**, 853–860, DOI: 10.1017/s0950268809991373 (2009).
11. Price, R. H. M., Graham, C. & Ramalingam, S. Association between viral seasonality and meteorological factors. *Sci. Reports* **9**, DOI: 10.1038/s41598-018-37481-y (2019).
12. White, L. *et al.* Understanding the transmission dynamics of respiratory syncytial virus using multiple time series and nested models. *Math. Biosci.* **209**, 222–239, DOI: 10.1016/j.mbs.2006.08.018 (2006).
13. Zhang, Y. *et al.* Burden of respiratory syncytial virus infections in China: Systematic review and meta-analysis. *J. Glob. Heal.* **5**, DOI: 10.7189/jogh.05.020417 (2015).
14. Flahault, A. *et al.* Virtual surveillance of communicable diseases: a 20-year experience in France. *Stat Methods Med Res* **15**, 413–421, DOI: 10.1177/0962280206071639 (2006).
15. Kusel, M. M. H. *et al.* Role of respiratory viruses in acute upper and lower respiratory tract illness in the first year of life. *The Pediatr. Infect. Dis. J.* **25**, 680–686, DOI: 10.1097/01.inf.0000226912.88900.a3 (2006).
16. Bogoch, I. I., Andrews, J. R., Zachary, K. C. & Hohmann, E. L. Diagnosis of influenza from lower respiratory tract sampling after negative upper respiratory tract sampling. *Virulence* **4**, 82–84, DOI: 10.4161/viru.22466 (2013).
17. Simoes, E. A. F. *et al.* Chapter 25. acute respiratory infections in children. In *Disease Control Priorities in Developing Countries. 2nd edition* (The World Bank/ Oxford University Press, 2006).
18. Matrajt, L. & Leung, T. Evaluating the effectiveness of social distancing interventions to delay or flatten the epidemic curve of coronavirus disease. *Emerg. Infect. Dis.* **26**, 1740–1748, DOI: 10.3201/eid2608.201093 (2020).
19. Chandra, S. K., Singh, A. & Bajpai, M. K. Mathematical model with social distancing parameter for early estimation of covid-19 spread. *medrxiv* DOI: 10.1101/2020.04.30.20086611 (2020).
20. Rozy, A. & Chorostowska-Wynimko, J. Bacterial immunostimulants–mechanism of action and clinical application in respiratory diseases. *Pneumonol Alergol Pol* **76**, 353–359 (2008).
21. Nestorov, I. Whole body pharmacokinetic models. *Clin. Pharmacokinet.* **42**, 883–908, DOI: 10.2165/00003088-200342100-00002 (2003).
22. Agoram, B., Woltosz, W. S. & Bolger, M. B. Predicting the impact of physiological and biochemical processes on oral drug bioavailability. *Adv. Drug Deliv. Rev.* **50**, S41–S67, DOI: 10.1016/s0169-409x(01)00179-x (2001).

23. Burckhart, M. F., Mimouni, J. & Fontanges, R. Absorption kinetics of a 14c-labelled escherichia coli extract after oral administration in mice. *Arzneimittel-Forschung* **47**, 325–328 (1997).

24. van Dijk, A., Bauer, J., Sedelmeier, E. A. & Bessler, W. G. Absorption, kinetics, antibody-bound and free serum determination of a 14C-labeled Escherichia coli extract after single oral administration in rats. *Arzneimittelforschung* **47**, 329–334 (1997).

25. Brown, R. P., Delp, M. D., Lindstedt, S. L., Rhomberg, L. R. & Beliles, R. P. Physiological parameter values for physiologically based pharmacokinetic models. *Toxicol Ind Heal.* **13**, 407–484, DOI: 10.1177/074823379701300401 (1997).

26. Kirman, C. *et al.* Physiologically based pharmacokinetic model for rats and mice orally exposed to chromium. *Chem. Interactions* **200**, 45–64, DOI: 10.1016/j.cbi.2012.08.016 (2012).

27. Bjorkman, S. Prediction of drug disposition in infants and children by means of physiologically based pharmacokinetic (PBPK) modelling: theophylline and midazolam as model drugs. *Br J Clin Pharmacol* **59**, 691–704, DOI: 10.1111/j.1365-2125.2004.02225.x (2005).

28. Schlender, J.-F. *et al.* Development of a whole-body physiologically based pharmacokinetic approach to assess the pharmacokinetics of drugs in elderly individuals. *Clin Pharmacokinet* **55**, 1573–1589, DOI: 10.1007/s40262-016-0422-3 (2016).

29. authors listed, N. Human alimentary tract model for radiological protection. ICRP Publication 100. A report of The International Commission on Radiological Protection. *Ann ICRP* **36**, 25–327 (2006).

30. Johnson, T., Bonner, J., Tucker, G., Turner, D. & Jamei, M. Development and applications of a physiologically-based model of paediatric oral drug absorption. *Eur. J. Pharm. Sci.* **115**, 57–67, DOI: 10.1016/j.ejps.2018.01.009 (2018).

31. Bessler, W. G., vor dem Esche, U. & Masihi, N. The bacterial extract OM-85 BV protects mice against influenza and salmonella infection. *Int. Immunopharmacol.* **10**, 1086–1090, DOI: 10.1016/j.intimp.2010.06.009 (2010).

32. Jesenak, M., Ciljakova, M., Rennerova, Z., Babusikova, E. & Banovci, P. Recurrent respiratory infections in children – definition, diagnostic approach, treatment and prevention. In *Bronchitis*, DOI: 10.5772/19422 (InTech, 2011).

33. Danek, K. & Felus, E. Influence of oral bacterial lysate stimulation on local humoral immunity on bronchial asthma patients. *Int. Rev. Allergol. Clin. Immunol.* **2** (1996).

34. Rossi, G. A., Pohunek, P., Feleszko, W., Ballarini, S. & Colin, A. A. Viral infections and wheezing–asthma inception in childhood: is there a role for immunomodulation by oral bacterial lysates? *Clin. Transl. Allergy* **10**, DOI: 10.1186/s13601-020-00322-1 (2020).

35. Lacoma, A. *et al.* Impact of host genetics and biological response modifiers on respiratory tract infections. *Front. Immunol.* **10**, DOI: 10.3389/fimmu.2019.01013 (2019).

36. Carlsson, C. J. *et al.* Duration of wheezy episodes in early childhood is independent of the microbial trigger. *J. Allergy Clin. Immunol.* **136**, 1208–1214.e5, DOI: 10.1016/j.jaci.2015.05.003 (2015).

37. Yin, J., Xu, B., Zeng, X. & Shen, K. Broncho-vaxom in pediatric recurrent respiratory tract infections: A systematic review and meta-analysis. *Int. Immunopharmacol.* **54**, 198–209, DOI: 10.1016/j.intimp.2017.10.032 (2018).

38. Boissel, J.-P., Kahoul, R., Marin, D. & Boissel, F.-H. Effect model law: An approach for the implementation of personalized medicine. *JPM* **3**, 177–190, DOI: 10.3390/jpm3030177 (2013).

39. Magee, M. H., Blum, R. A., Lates, C. D. & Jusko, W. J. Pharmacokinetic/pharmacodynamic model for prednisolone inhibition of whole blood lymphocyte proliferation. *Br. J. Clin. Pharmacol.* **53**, 474–484, DOI: 10.1046/j.1365-2125.2002.01567.x (2002).

40. Netea, M. G. *et al.* Defining trained immunity and its role in health and disease. *Nat. Rev. Immunol.* **20**, 375–388, DOI: 10.1038/s41577-020-0285-6 (2020).

41. Pérez-Vázquez, D., Contreras-Castillo, E. & Licona-Limón, P. Memoria inmunológica innata, la pieza faltante de la respuesta inmunológica. *TIP Revista Especializada en Ciencias Químico-Biológicas* **21**, DOI: 10.22201/fesz.23958723e.2018.0.151 (2018).

42. Sánchez-Ramón, S. *et al.* Trained immunity-based vaccines: A new paradigm for the development of broad-spectrum anti-infectious formulations. *Front. Immunol.* **9**, DOI: 10.3389/fimmu.2018.02936 (2018).

43. Saltelli, A., Chan, K. & Scott, M. Sensitivity analysis (2000).

44. Pawelek, K. A. *et al.* Modeling within-host dynamics of influenza virus infection including immune responses. *PLoS Comput. Biol.* **8**, e1002588, DOI: 10.1371/journal.pcbi.1002588 (2012).

45. Royal College of General Practitioners (RCGP) Research & Surveillance Centre. RSC communicable and respiratory disease report for england - week 31. Tech. Rep., University of Oxford, University of Surrey (2020).

46. Reis, J. & Shaman, J. Simulation of four respiratory viruses and inference of epidemiological parameters. *Infect. Dis. Model.* **3**, 23–34, DOI: 10.1016/j.idm.2018.03.006 (2018).

47. Zhu, H. & Lakkis, H. Sample size calculation for comparing two negative binomial rates. *Stat. Med.* **33**, 376–387, DOI: 10.1002/sim.5947 (2013).

48. Davis, C. L. *et al.* Applying mathematical tools to accelerate vaccine development: Modeling shigella immune dynamics. *PLoS ONE* **8**, e59465, DOI: 10.1371/journal.pone.0059465 (2013).

49. Nickbakhsh, S. *et al.* Virus-virus interactions impact the population dynamics of influenza and the common cold. *Proc. Natl. Acad. Sci.* **116**, 27142–27150, DOI: 10.1073/pnas.1911083116 (2019).

50. Samsuzzoha, M., Singh, M. & Lucy, D. Numerical study of a diffusive epidemic model of influenza with variable transmission coefficient. *Appl. Math. Model.* **35**, 5507–5523, DOI: 10.1016/j.apm.2011.04.029 (2011).

51. Byl, B. *et al.* Bacterial Extract OM85-BV Induces Interleukin-12-Dependent IFN-γ Production by Human CD4+ T Cells. *J. Interf. & Cytokine Res.* **18**, 817–821, DOI: 10.1089/jir.1998.18.817 (1998).

52. Bromage, E., Stephens, R. & Hassoun, L. The third dimension of ELISPOTs: Quantifying antibody secretion from individual plasma cells. *J. Immunol. Methods* **346**, 75–79, DOI: 10.1016/j.jim.2009.05.005 (2009).

53. Mesin, L., Niro, R. D., Thompson, K. M., Lundin, K. E. A. & Sollid, L. M. Long-lived plasma cells from human small intestine biopsies secrete immunoglobulins for many weeks in vitro. *The J. Immunol.* **187**, 2867–2874, DOI: 10.4049/jimmunol.1003181 (2011).

54. Burnett, D. Immunoglobulins in the lung. *Thorax* **41**, 337–344, DOI: 10.1136/thx.41.5.337 (1986).

55. Eisenbarth, S. C. Dendritic cell subsets in t cell programming: location dictates function. *Nat. Rev. Immunol.* **19**, 89–103, DOI: 10.1038/s41577-018-0088-1 (2018).

56. Borghans, J. A. M., Tesselaar, K. & de Boer, R. J. Current best estimates for the average lifespans of mouse and human leukocytes: reviewing two decades of deuterium-labeling experiments. *Immunol. Rev.* **285**, 233–248, DOI: 10.1111/imr.12693 (2018).
