# Supplementary material for: Modeling the disruption of respiratory disease clinical trials by non-pharmaceutical COVID-19 interventions
Source: Nat Commun. 2022 Apr 13;13:1980. doi: 10.1038/s41467-022-29534-8 (PMC9008035; doi:10.1038/s41467-022-29534-8)
Supplement: Supplementary file 2 — Reporting Summary [file 41467_2022_29534_MOESM2_ESM.pdf]

nature portfolio

Corresponding author(s): Alexander Kulesza

Last updated by author(s): Mar 8, 2022

Reporting Summary

Nature Portfolio wishes to improve the reproducibility of the work that we publish. This form provides structure for consistency and transparency in reporting. For further information on Nature Portfolio policies, see our Editorial Policies and the Editorial Policy Checklist.

Statistics

For all statistical analyses, confirm that the following items are present in the figure legend, table legend, main text, or Methods section.

n/a	Confirmed	
☐	☒	The exact sample size (n) for each experimental group/condition, given as a discrete number and unit of measurement
☐	☒	A statement on whether measurements were taken from distinct samples or whether the same sample was measured repeatedly
☐	☒	The statistical test(s) used AND whether they are one- or two-sided<br>*Only common tests should be described solely by name; describe more complex techniques in the Methods section.*
☒	☐	A description of all covariates tested
☐	☒	A description of any assumptions or corrections, such as tests of normality and adjustment for multiple comparisons
☐	☒	A full description of the statistical parameters including central tendency (e.g. means) or other basic estimates (e.g. regression coefficient) AND variation (e.g. standard deviation) or associated estimates of uncertainty (e.g. confidence intervals)
☐	☒	For null hypothesis testing, the test statistic (e.g. F, t, r) with confidence intervals, effect sizes, degrees of freedom and P value noted<br>*Give P values as exact values whenever suitable.*
☒	☐	For Bayesian analysis, information on the choice of priors and Markov chain Monte Carlo settings
☒	☐	For hierarchical and complex designs, identification of the appropriate level for tests and full reporting of outcomes
☒	☐	Estimates of effect sizes (e.g. Cohen's d, Pearson's r), indicating how they were calculated

Our web collection on statistics for biologists contains articles on many of the points above.

Software and code

Policy information about availability of computer code

Data collection	JINKO Simwork v22.39.0 (Novadiscovery) was used for the implementation of the ODE system with reactions, species, parameters, rules and assignments as well as events.<br>Additionally, the model source is provided in the Supplementary File as a SBML file (Level 3 Version 2) along with a Python (3.7) script to run the model for a reference patient in a reference scenario using libroadrunner (http://libroadrunner.org/) a C/C++ library that supports simulation of SBML based models (version 2.2.0), reference model outputs for all variables (.csv, .pdf), summary model implementation table (.xls) as well as mapping between the source and the human readable description (pdf, xls).

Data analysis	Analysis to produce the results from the raw data was performed with custom Python / R scripts and jupyter notebooks. They are provided as Supplementary File along with the necessary raw data.<br><br>Packages version:<br>- Python 3.9.7<br>- Numpy 1.19.5<br>- scipy 1.8.0<br>- rpy2 3.4.5<br>- pandas 1.4.1<br>- matplotlib 3.5.1<br>- jupyterlab 3.2.9<br>- R 4.0.4<br>- MKmisc 1.8<br>- MASS 7.3-55

For manuscripts utilizing custom algorithms or software that are central to the research but not yet described in published literature, software must be made available to editors and reviewers. We strongly encourage code deposition in a community repository (e.g. GitHub). See the Nature Portfolio guidelines for submitting code & software for further information.

Data

Policy information about availability of data

All manuscripts must include a data availability statement. This statement should provide the following information, where applicable:

- Accession codes, unique identifiers, or web links for publicly available datasets
- A description of any restrictions on data availability
- For clinical datasets or third party data, please ensure that the statement adheres to our policy

The data (simulation outputs, in silico clinical trials and analyses) generated in this study and needed to reproduce the results presented in the figures are provided as comma-separated-value files (csv) compressed into one file (zip) as Supplementary File. 5-year average and the 2019-2020 upper and lower RTI (URTI and LRTI) incidence from the communicable and respiratory disease report 2019 to 2020 published in the UK by the Royal College of General Practitioners (RCGP) can be accessed at the following address: https://www.rcgp.org.uk/-/media/Files/CIRC/WeeklyReport_Summer_wk31_2020.ashx. All other datasets used (viral load evolution after experimental viral challenge, PK/PD data, age-dependent RTI distribution , meta-analyzed clinical efficacy) were obtained from published reports whose references are cited.

Field-specific reporting

Please select the one below that is the best fit for your research. If you are not sure, read the appropriate sections before making your selection.

☒ Life sciences ☐ Behavioural & social sciences ☐ Ecological, evolutionary & environmental sciences

For a reference copy of the document with all sections, see nature.com/documents/nr-reporting-summary-flat.pdf

Life sciences study design

All studies must disclose on these points even when the disclosure is negative.

Sample size	Virtual populations have no limit in sample size. We mimicked realistic limitations in sample size by selecting a finite sample per in silico arm (e.g. 50, see main text).
Data exclusions	No data was excluded from the analyses.
Replication	Seeds were used to enforce the replicability of the random sampling process to generate in silico trials from the database of simulated virtual patients (see analysis code in Supplementary File).
Randomization	In silico, virtual patients are fully randomized by principle. We mimicked randomization issues by selecting a finite sample per in silico arm (e.g. 50, see main text).
Blinding	The model is calibrated based on a meta-analysis of clinical trials with various blinding schemes (Yin et al. 2018). The details of blinding schemes are not reported in the meta-analysis.

Reporting for specific materials, systems and methods

We require information from authors about some types of materials, experimental systems and methods used in many studies. Here, indicate whether each material, system or method listed is relevant to your study. If you are not sure if a list item applies to your research, read the appropriate section before selecting a response.

Materials & experimental systems

n/a	Involved in the study
[x]	[ ] Antibodies
[x]	[ ] Eukaryotic cell lines
[x]	[ ] Palaeontology and archaeology
[x]	[ ] Animals and other organisms
[x]	[ ] Human research participants
[x]	[ ] Clinical data
[x]	[ ] Dual use research of concern

Methods

n/a	Involved in the study
[x]	[ ] ChIP-seq
[x]	[ ] Flow cytometry
[x]	[ ] MRI-based neuroimaging
