# Supplementary material for: Modeling the disruption of respiratory disease clinical trials by non-pharmaceutical COVID-19 interventions
Source: Nat Commun. 2022 Apr 13;13:1980. doi: 10.1038/s41467-022-29534-8 (PMC9008035; doi:10.1038/s41467-022-29534-8)
Supplement: Supplementary file 4 — Source Data [file 41467_2022_29534_MOESM4_ESM.zip › SupplementaryFile/source/mappingSource_-_Text.pdf]

**Within-host RTI disease model**	
Variables	Model implementation
E_h	`RTT.hEC`
V	`RTT.virus`
E_i	`RTT.iEC`
L	`RTT.tCL`
Ig	`RTT.ig`
Parameters	Model implementation
n_i	`kLystCL`
d_h	`dHEC`
d_i	`dIEC`
p_h	`rRegen`
d_v	`dVirus`
k_{Inf}	`kInfect`
v_f	`virulenceFactorEff`
c	`thresholdNaturalClearance`
p_c	`vMaxProInf`
K_c	`ec50ProInf`
d_c	`dProInfCyt`
p_v	`rVirus`
p_ig	`pIgRef`
d_ig	`deathIg`
S	`imStatus`
K_v	`kMVirus`
p_M	`vTrt`
K_M	`kMTrt`
n_v	`nIgVirus`
p_L	`atCLRef`
d_L	`dtCL`
$\bar{E}_h$	`concEC`
d_{Ig_A}	`digAns`

**Between-host SIRS model**	
Variables	Model implementation
S	`popdensComp.sPop`
I	`popdensComp.iPop`
R	`popdensComp.rPop`
S_{RSV}	`popdensComp.sPopRSV`
I_{RSV}	`popdensComp.iPopRSV`
R_{RSV}	`popdensComp.rPopRSV`
S_{HRV}	`popdensComp.sPopRV`
I_{HRV}	`popdensComp.iPopRV`
R_{HRV}	`popdensComp.rPopRV`
S_{IV}	`popdensComp.sPopIV`
I_{IV}	`popdensComp.iPopIV`
R_{IV}	`popdensComp.rPopIV`
Parameters	Model implementation
N	`pop`
β_{RSV}	`betaRSV`
β_{HRV}	`betaRV`
β_{IV}	`betaIV`
$\beta_{0,RSV}$	`b0EUrsv`
$\beta_{0,HRV}$	`b0EUrhinovirus`
$\beta_{0,IV}$	`b0EUinfluenza`
$\beta_{1,RSV}$	`b1EUrsv`
$\beta_{1,HRV}$	`b01EUrhinovirus`
$\beta_{1,IV}$	`b1EUinfluenza`
ζ_{RSV}	`zetaRSV`
ζ_{HRV}	`zetaRV`
ζ_{IV}	`zetaIV`
γ_{RSV}	`gammaRSV`
γ_{HRV}	`gammaRV`
γ_{IV}	`gammaIV`
P_{RSV}	`phaseAngle`
P_{HRV}	`phaseAngle2`
P_{IV}	`phaseAngle3`
A	`ageTransParameter`
L	`covidInfluence`
f_{URTI}	`fractionURTI`
f_{LRTI}	`fractionLRTI`
$S_{RSV}(0)$	`sPopRSVInit`
$I_{RSV}(0)$	`iPopRSVInit`
$R_{RSV}(0)$	`rPopRSVInit`
$S_{HRV}(0)$	`sPopRVInit`
$I_{HRV}(0)$	`iPopRVInit`
$R_{HRV}(0)$	`rPopRVInit`
$S_{IV}(0)$	`sPopIVInit`
$I_{IV}(0)$	`iPopIVInit`
$R_{IV}(0)$	`rPopIVInit`

**Model of OM-85 immune activation in Peyer's Patches**	
Variables	Model implementation
D	`PP.dC`
O	`PP.OM85`
M_p	`PP.iMLp`
M	`PP.iML`
B_L	`PP.bLA`
B_P	`PP.bPAns`
T_r	`PP.tReg`
Parameters	Model implementation
E_O	`adC`
K_O	`kdC`
h	`hdC`
d_D	`ddC`
E_{M_p}	`aiMLp`
K_{M_p}	`kiMLp`
α	`piMLp`
d_{M_p}	`diMLp`
σ_{PP}	`sigmaPP`
E_{B_L}	`abLA`
K_{B_L}	`kbLA`
β	`bToP`
d_{B_L}	`dbLA`
E_{B_P}	`bToPdC`
K_{B_P}	`kbPAns`
d_{B_P}	`dbPAns`
E_{T_r}	`atReg`
K_{T_r}	`ktReg`
d_{T_r}	`dtReg`
