# Supplementary material for: Modeling the disruption of respiratory disease clinical trials by non-pharmaceutical COVID-19 interventions
Source: Nat Commun. 2022 Apr 13;13:1980. doi: 10.1038/s41467-022-29534-8 (PMC9008035; doi:10.1038/s41467-022-29534-8)
Supplement: Supplementary file 4 — Source Data [file 41467_2022_29534_MOESM4_ESM.zip › SupplementaryFile/source/results.pdf]

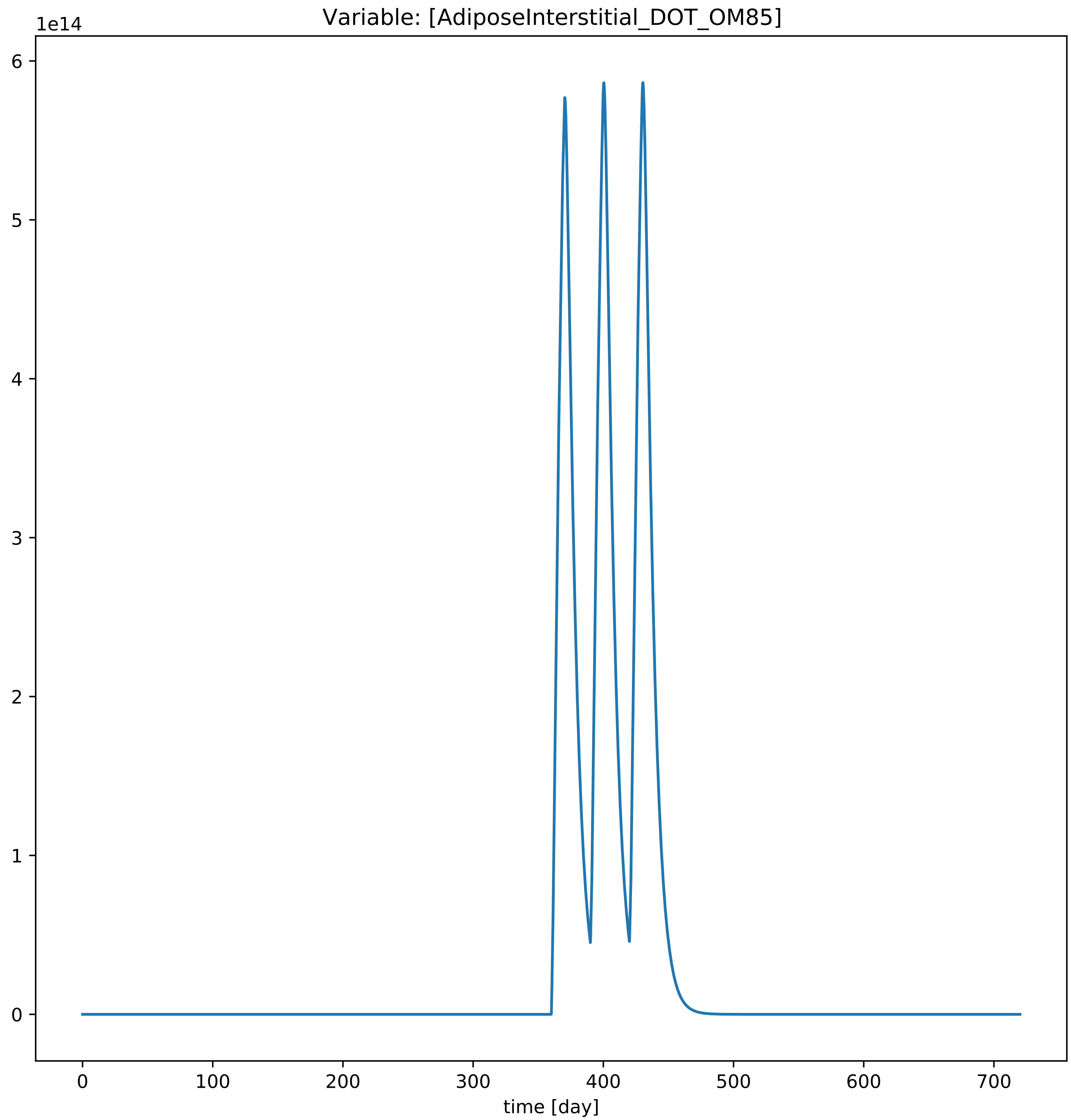

Variable: [AdiposeInterstitial_DOT_OM85]
1e14
6
5
4
3
2
1
0
0
100
200
300
400
500
600
700
time [day]

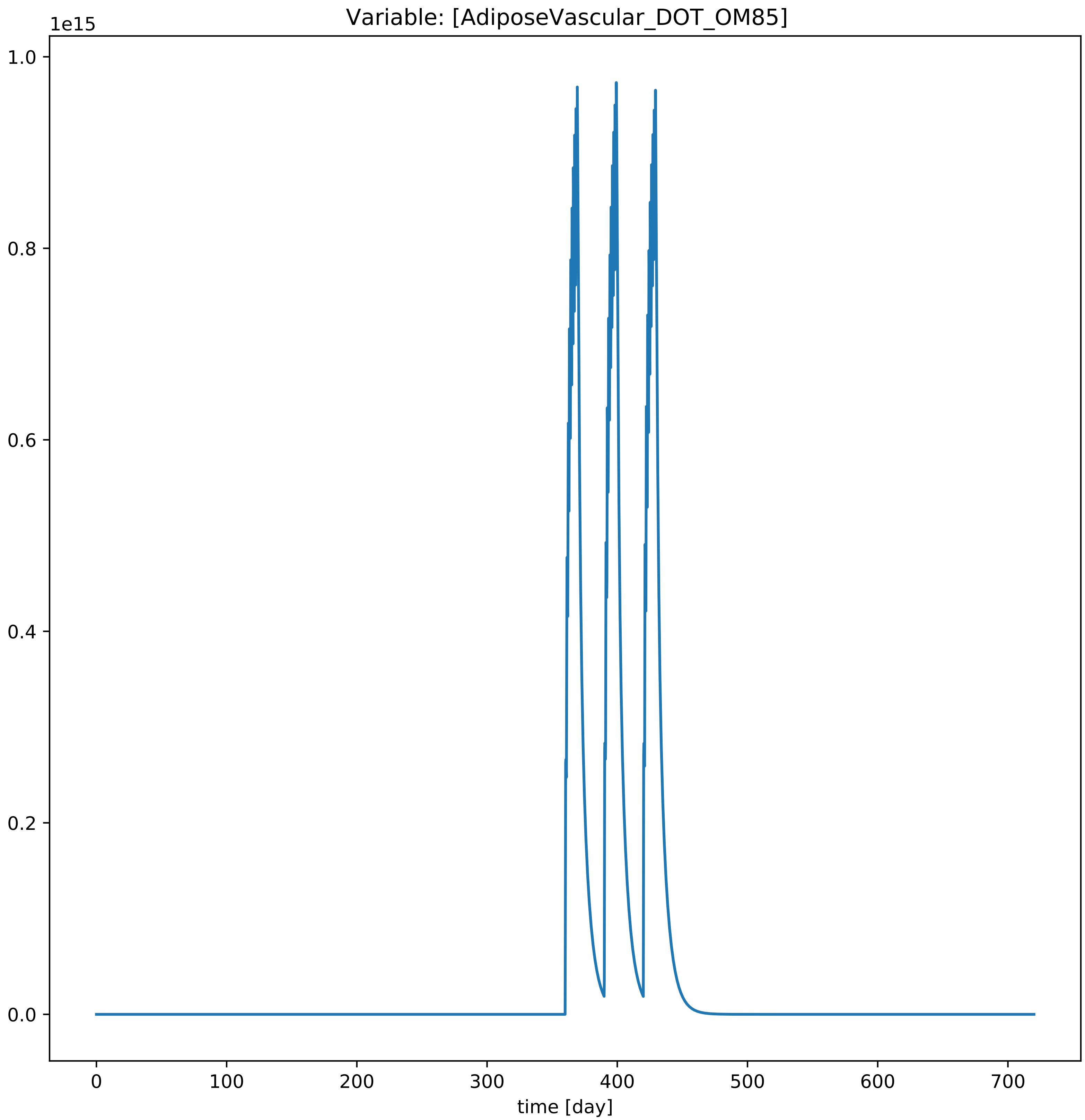

Variable: [AdiposeVascular_DOT_OM85]
1e15
1.0
0.8
0.6
0.4
0.2
0.0
0
100
200
300
400
500
600
700
time [day]

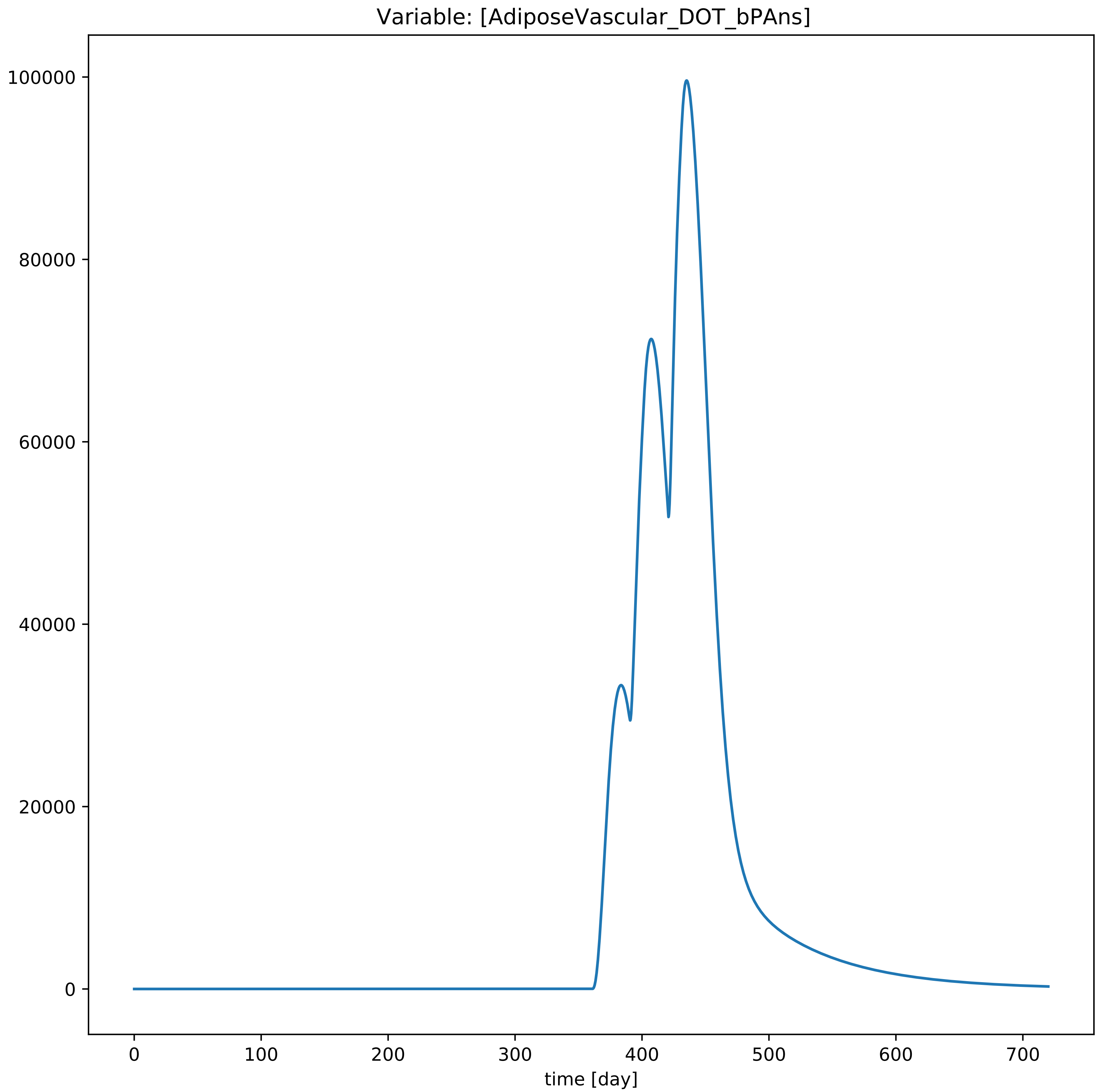
Variable: [AdiposeVascular_DOT_bPAns]
100000
80000
60000
40000
20000
0
0
100
200
300
400
500
600
700
time [day]

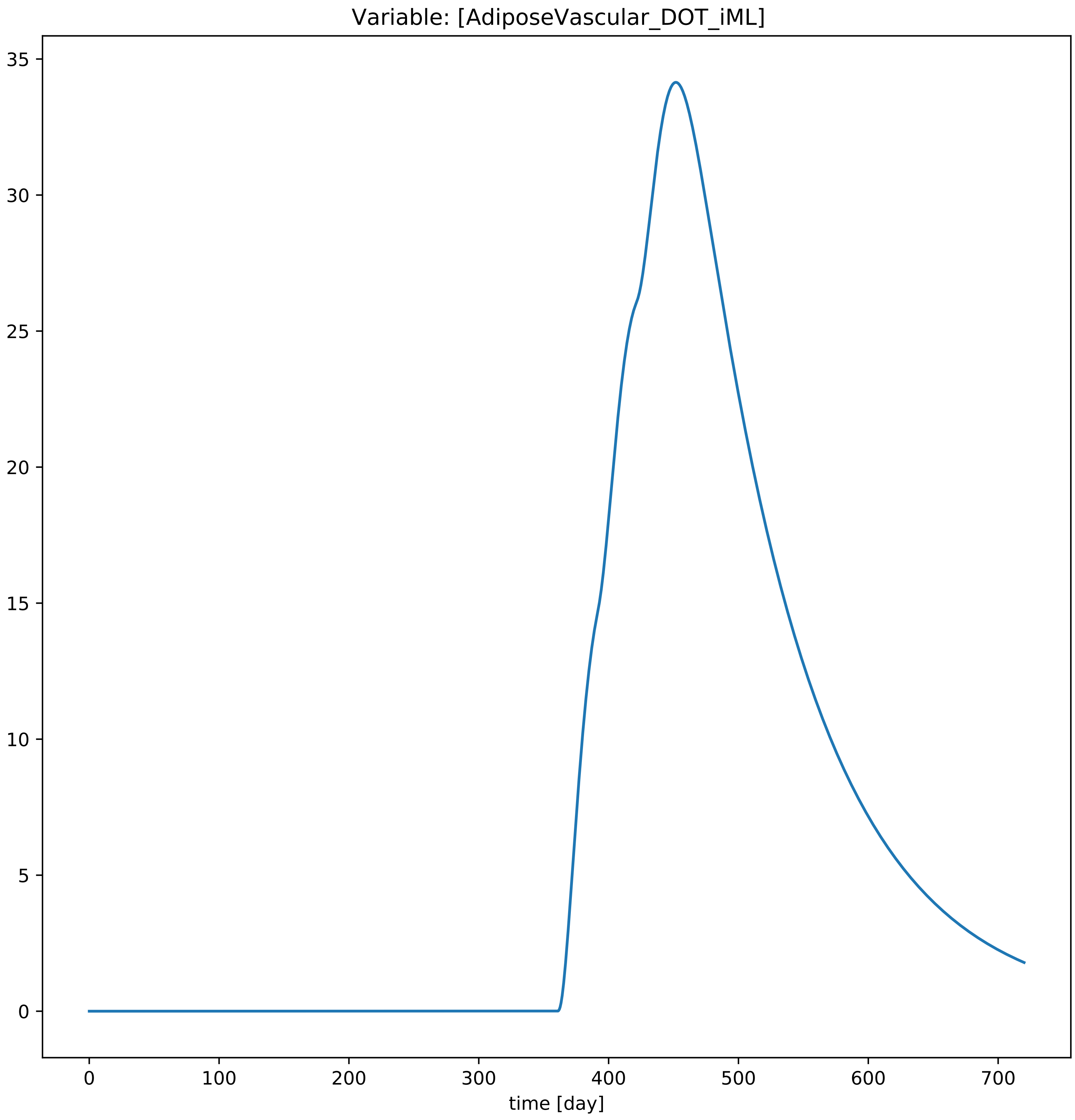
Variable: [AdiposeVascular_DOT_iML]
35
30
25
20
15
10
5
0
0
100
200
300
400
500
600
700
time [day]

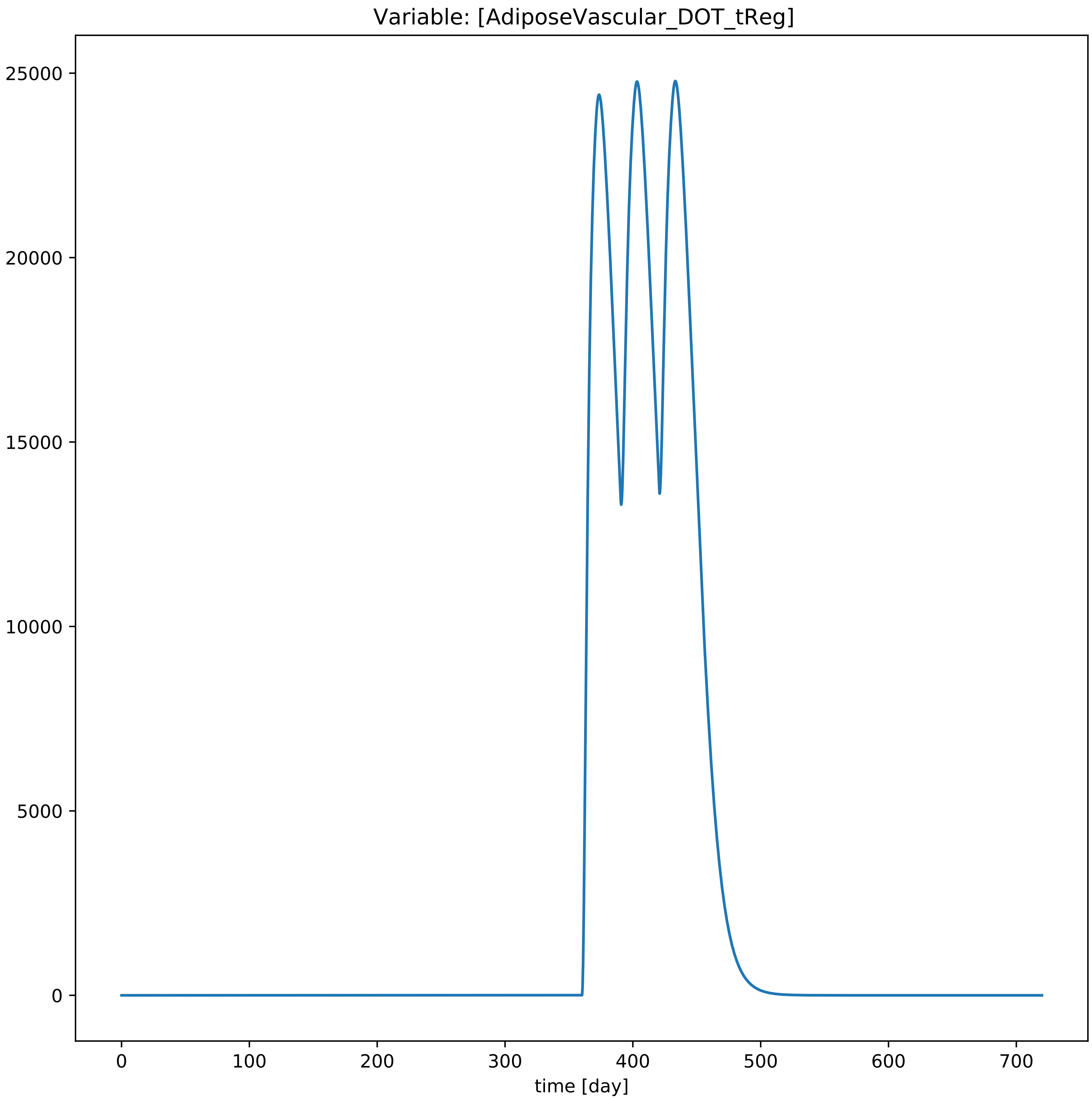

Variable: [AdiposeVascular_DOT_tReg]
25000
20000
15000
10000
5000
0
0
100
200
300
400
500
600
700
time [day]

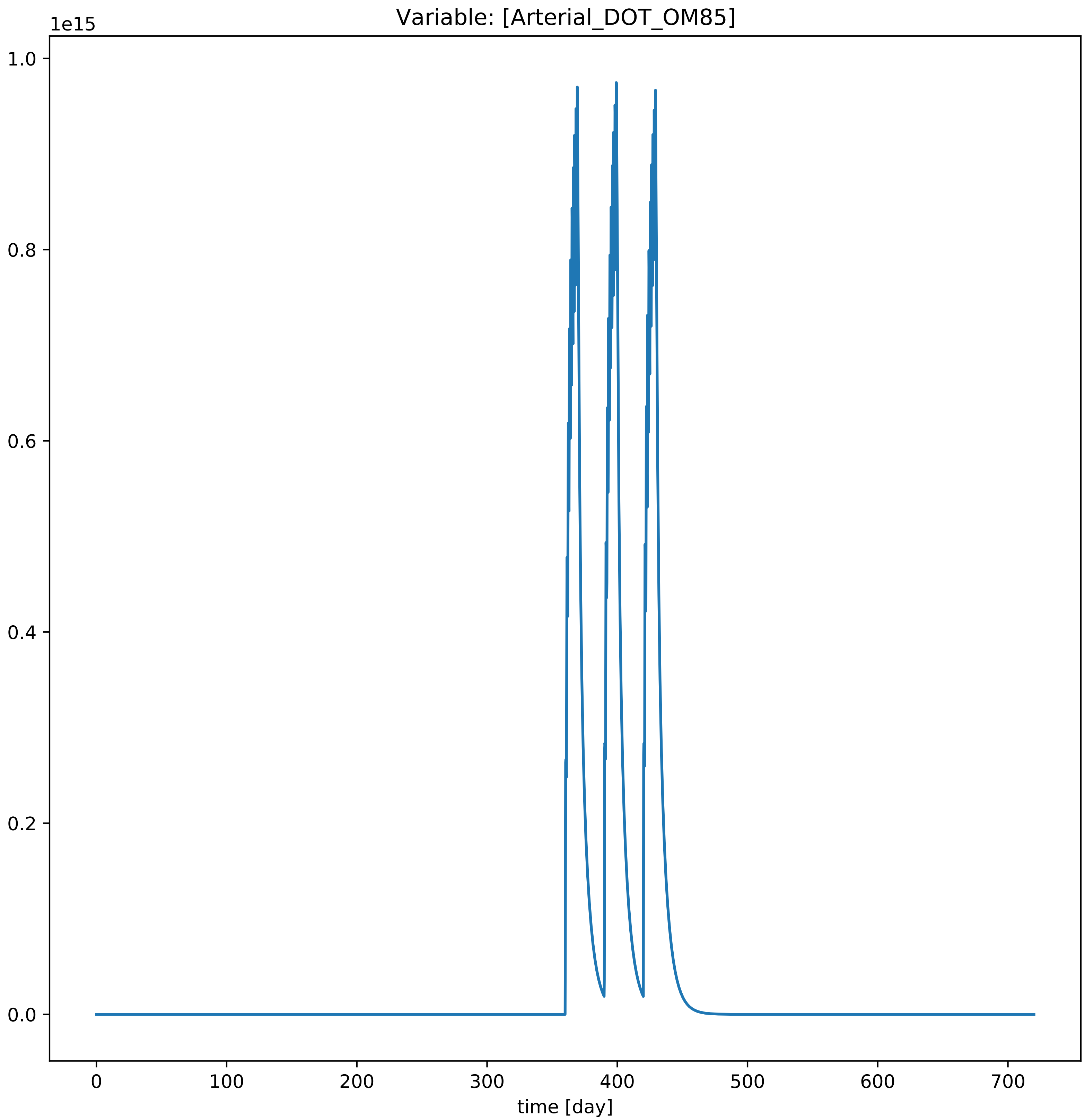

Variable: [Arterial_DOT_OM85]
1e15
1.0
0.8
0.6
0.4
0.2
0.0
0
100
200
300
400
500
600
700
time [day]

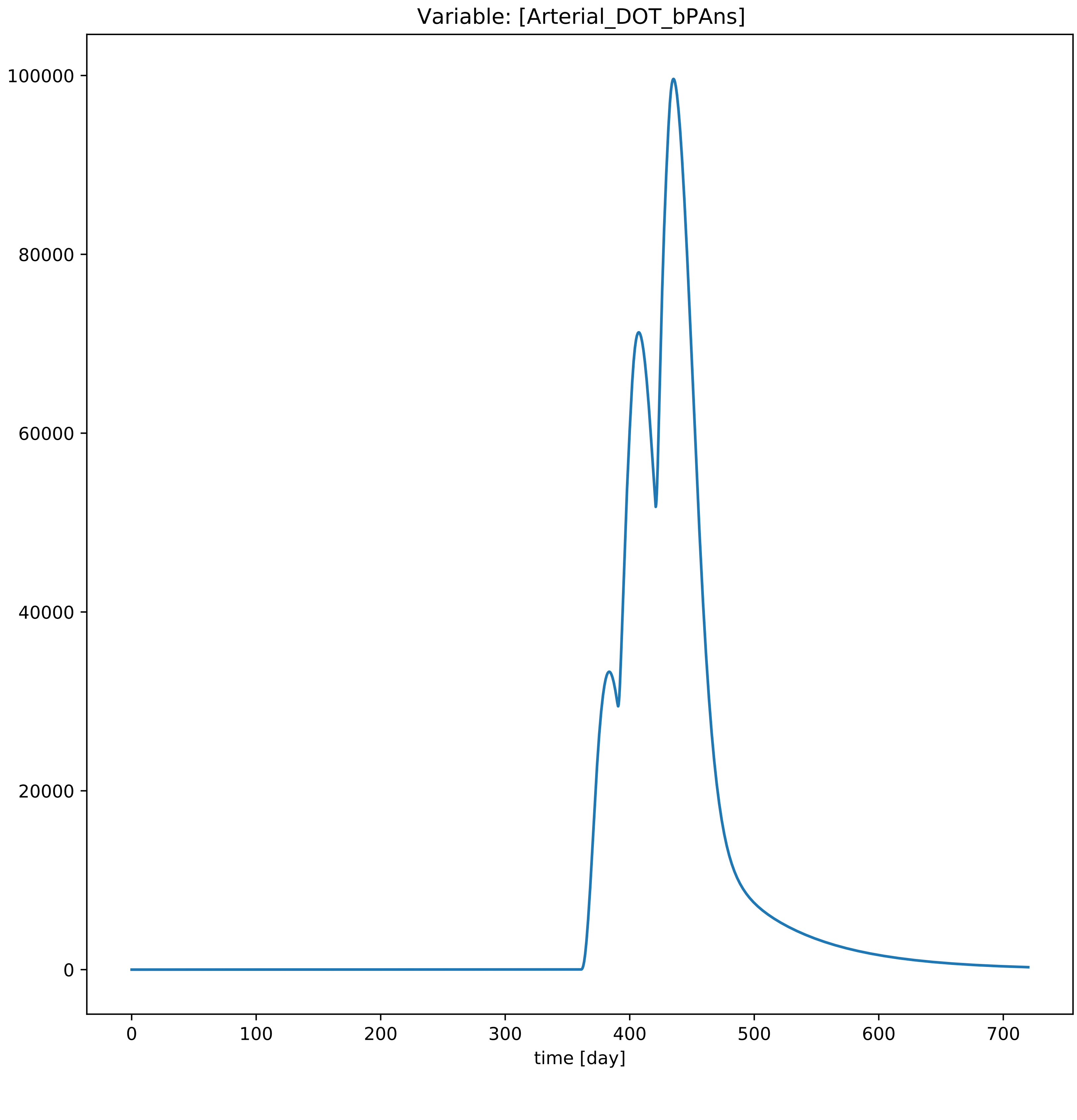

Variable: [Arterial_DOT_bPAns]
100000
80000
60000
40000
20000
0
0
100
200
300
400
500
600
700
time [day]

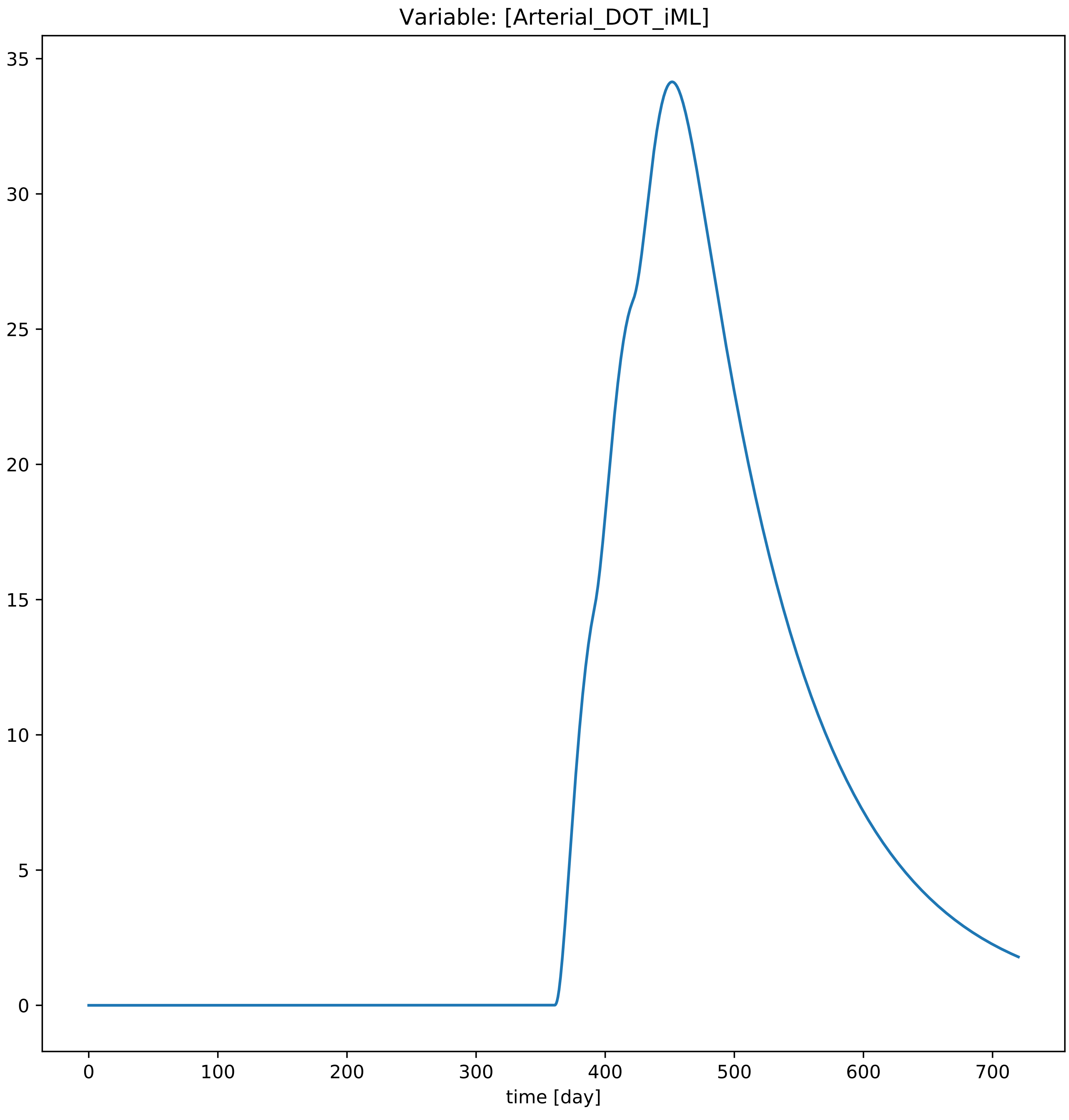

Variable: [Arterial_DOT_iML]
35
30
25
20
15
10
5
0
0
100
200
300
400
500
600
700
time [day]

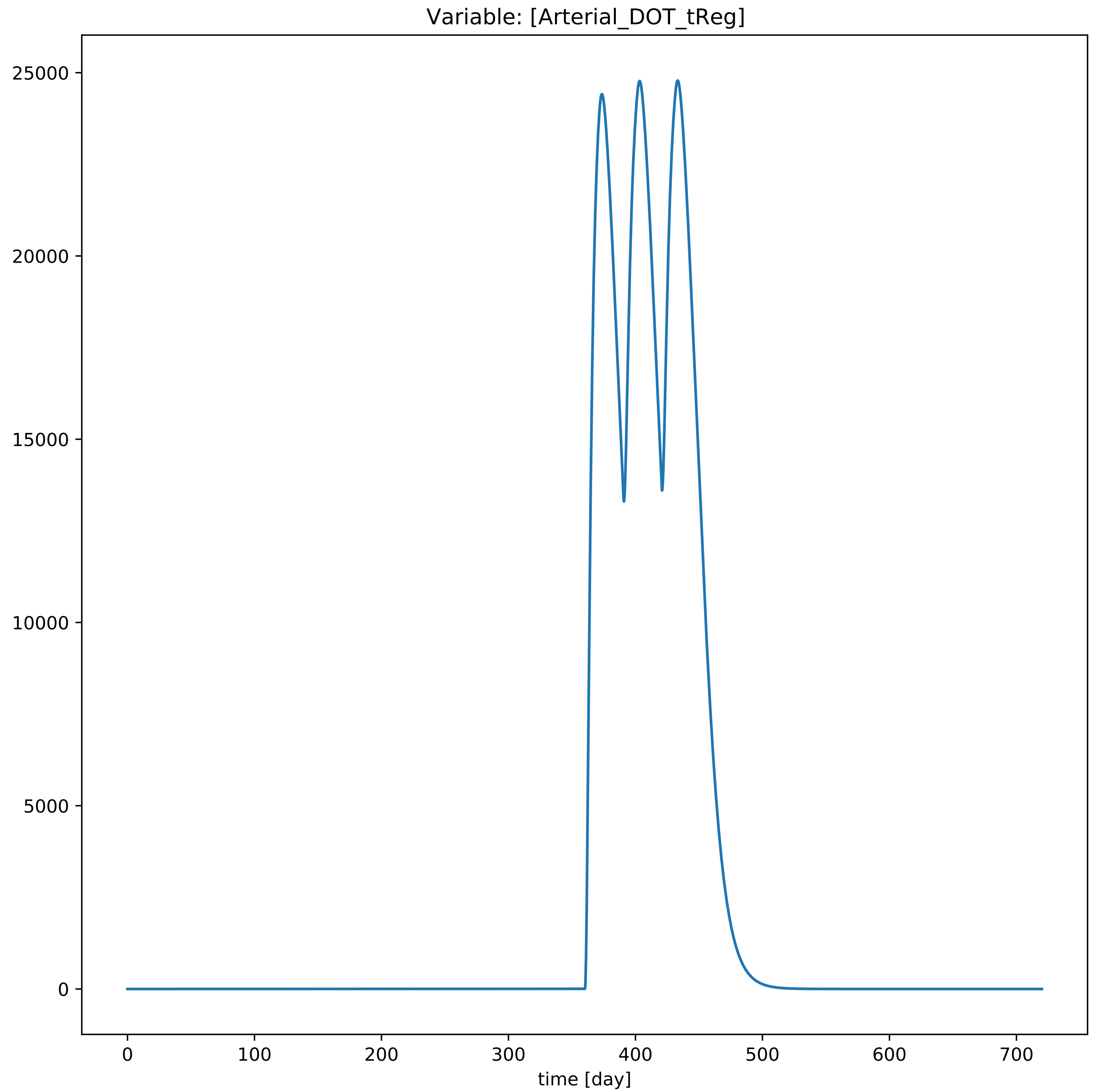

Variable: [Arterial_DOT_tReg]
25000
20000
15000
10000
5000
0
0
100
200
300
400
500
600
700
time [day]

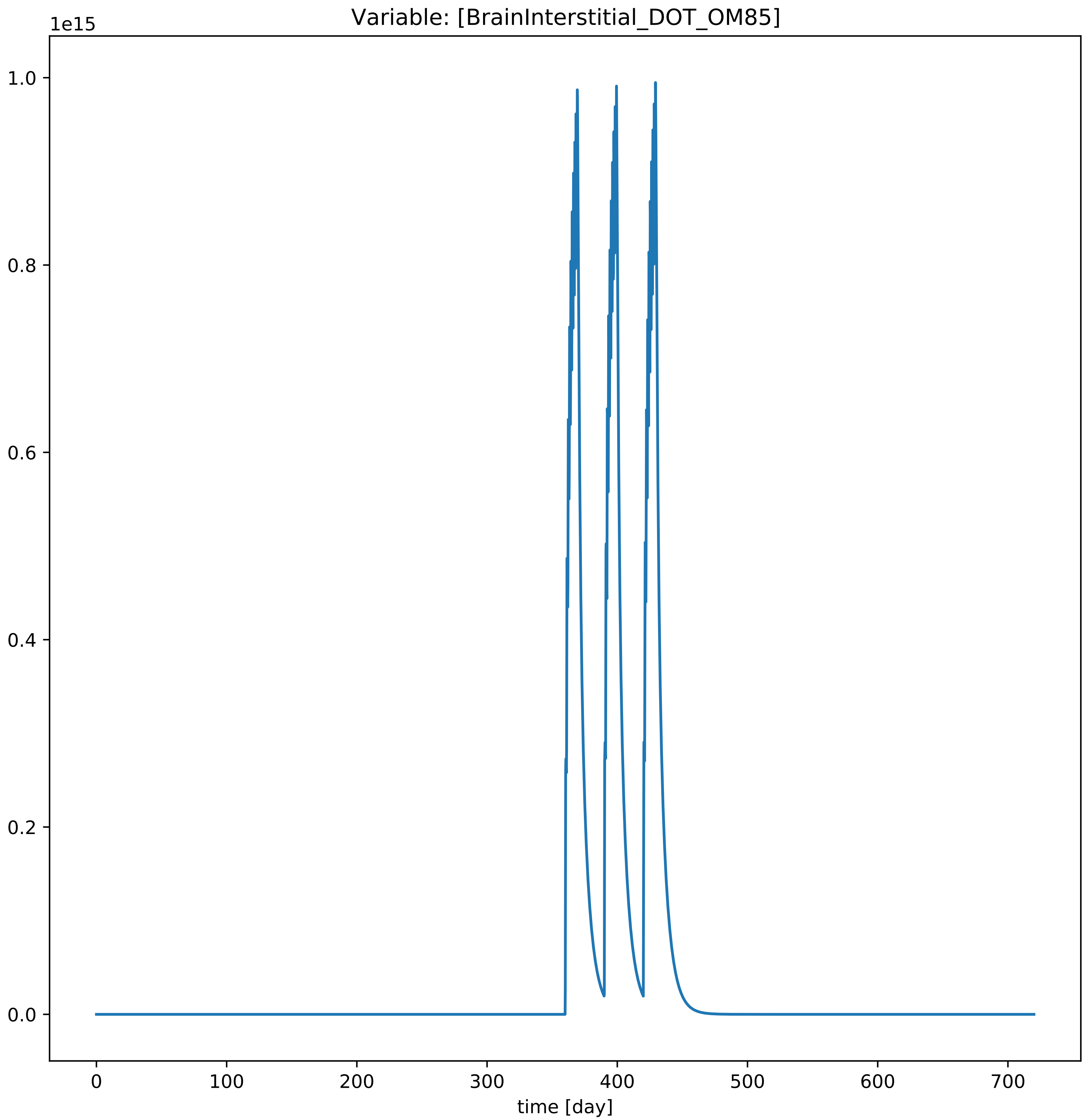

Variable: [BrainInterstitial_DOT_OM85]
1e15
1.0
0.8
0.6
0.4
0.2
0.0
0
100
200
300
400
500
600
700
time [day]

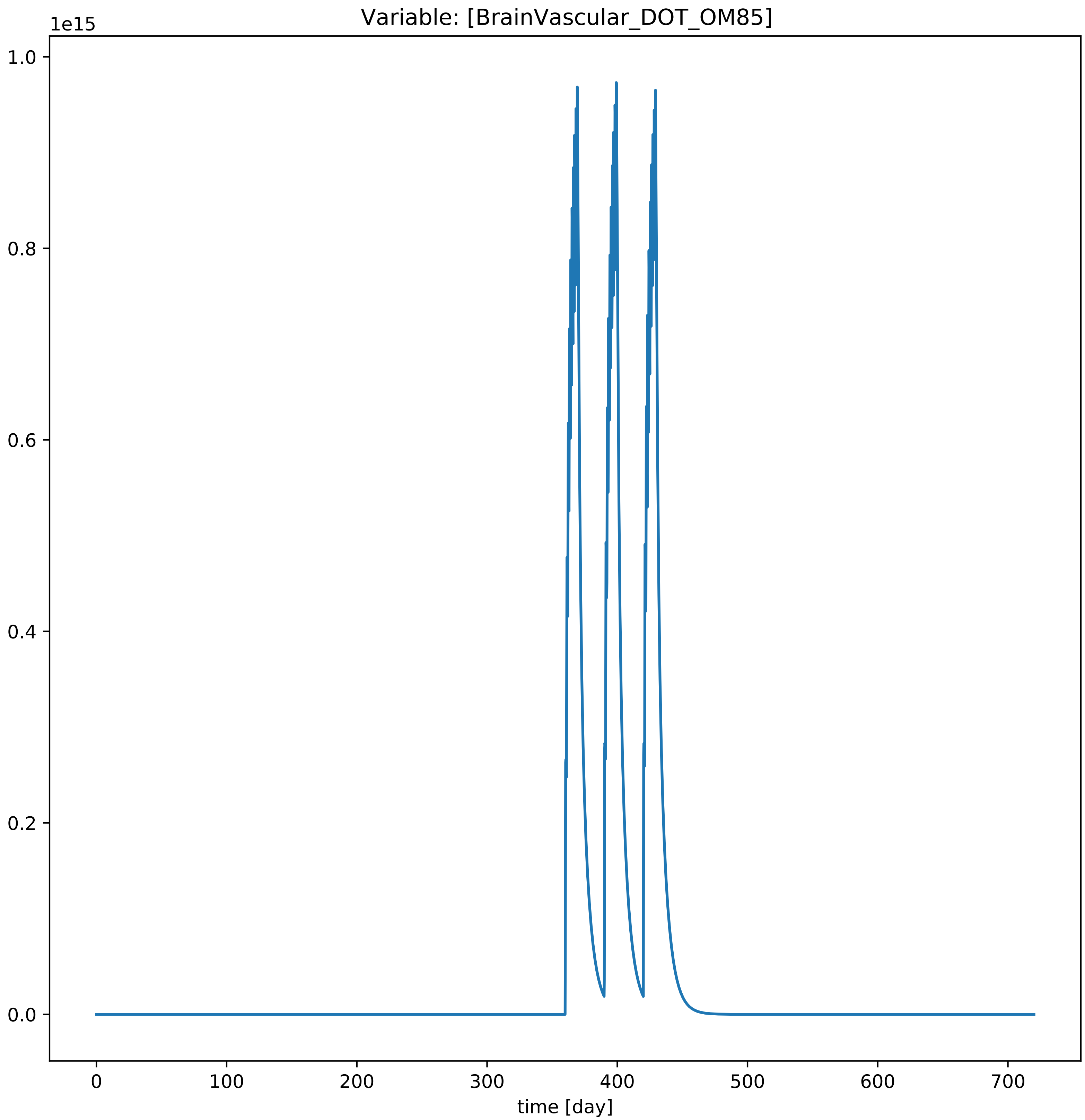

Variable: [BrainVascular_DOT_OM85]
1e15
1.0
0.8
0.6
0.4
0.2
0.0
0
100
200
300
400
500
600
700
time [day]

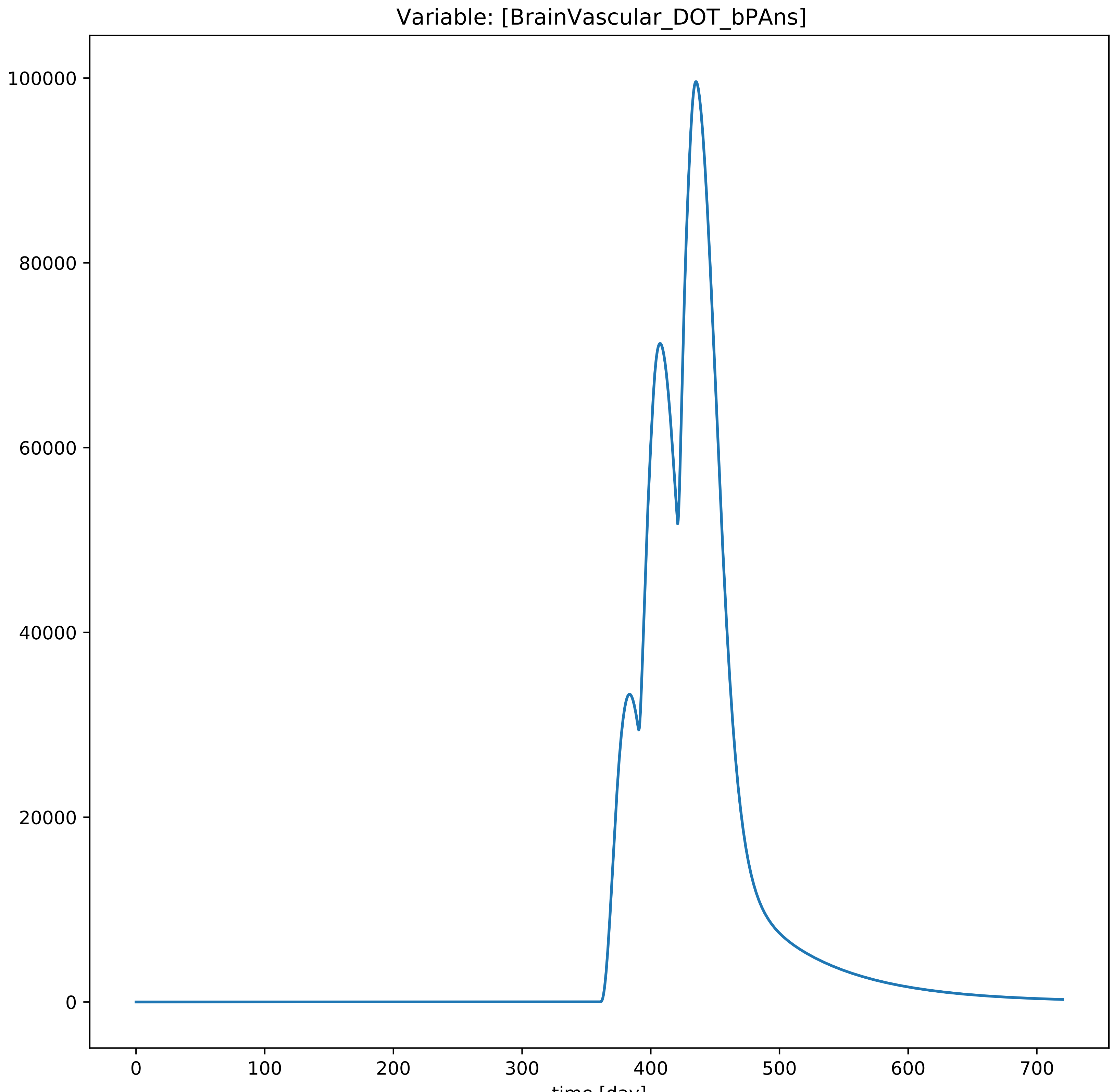

Variable: [BrainVascular_DOT_bPAns]
100000
80000
60000
40000
20000
0
0
100
200
300
400
500
600
700
time [day]

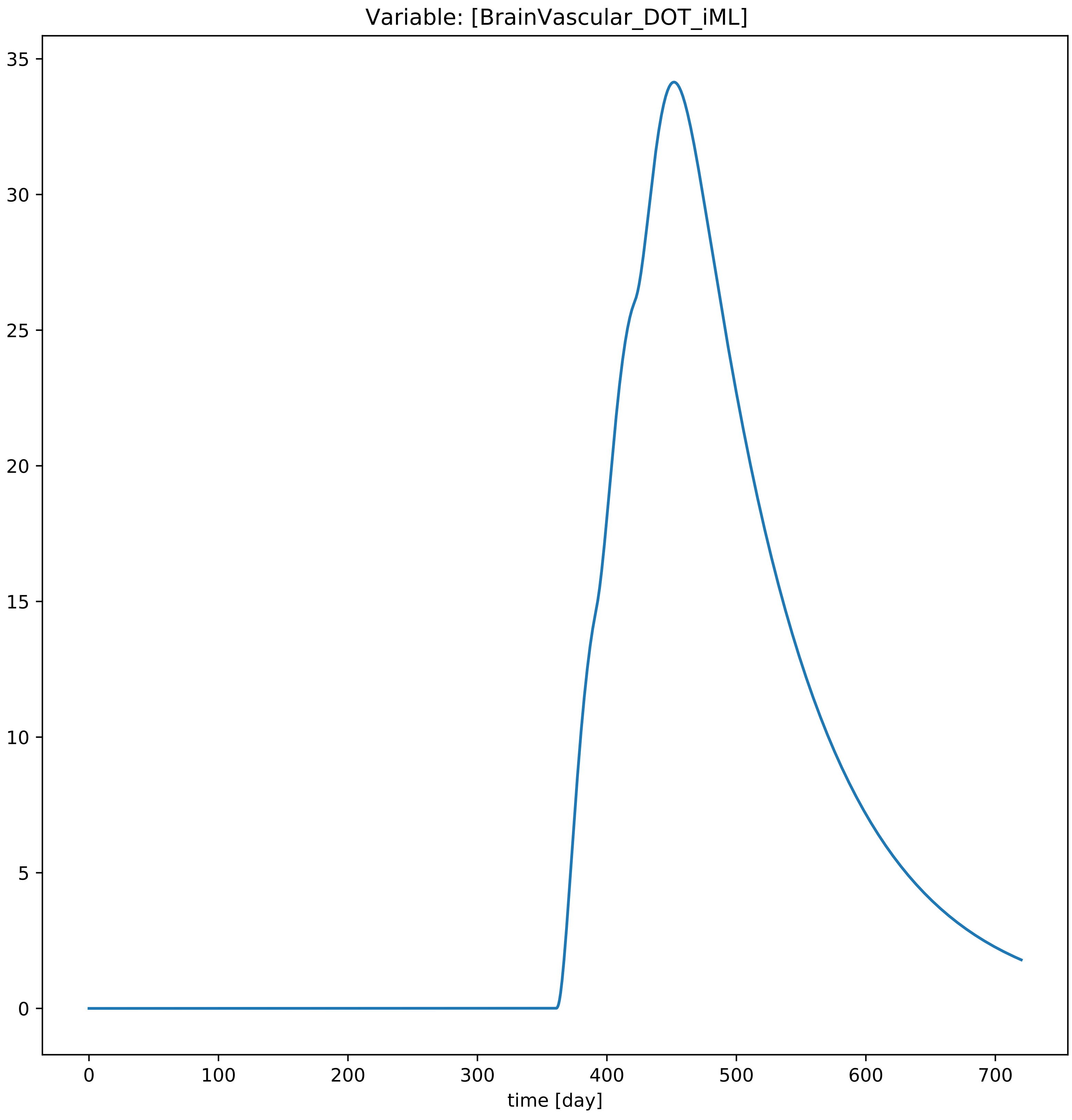

Variable: [BrainVascular_DOT_iML]
35
30
25
20
15
10
5
0
0
100
200
300
400
500
600
700
time [day]

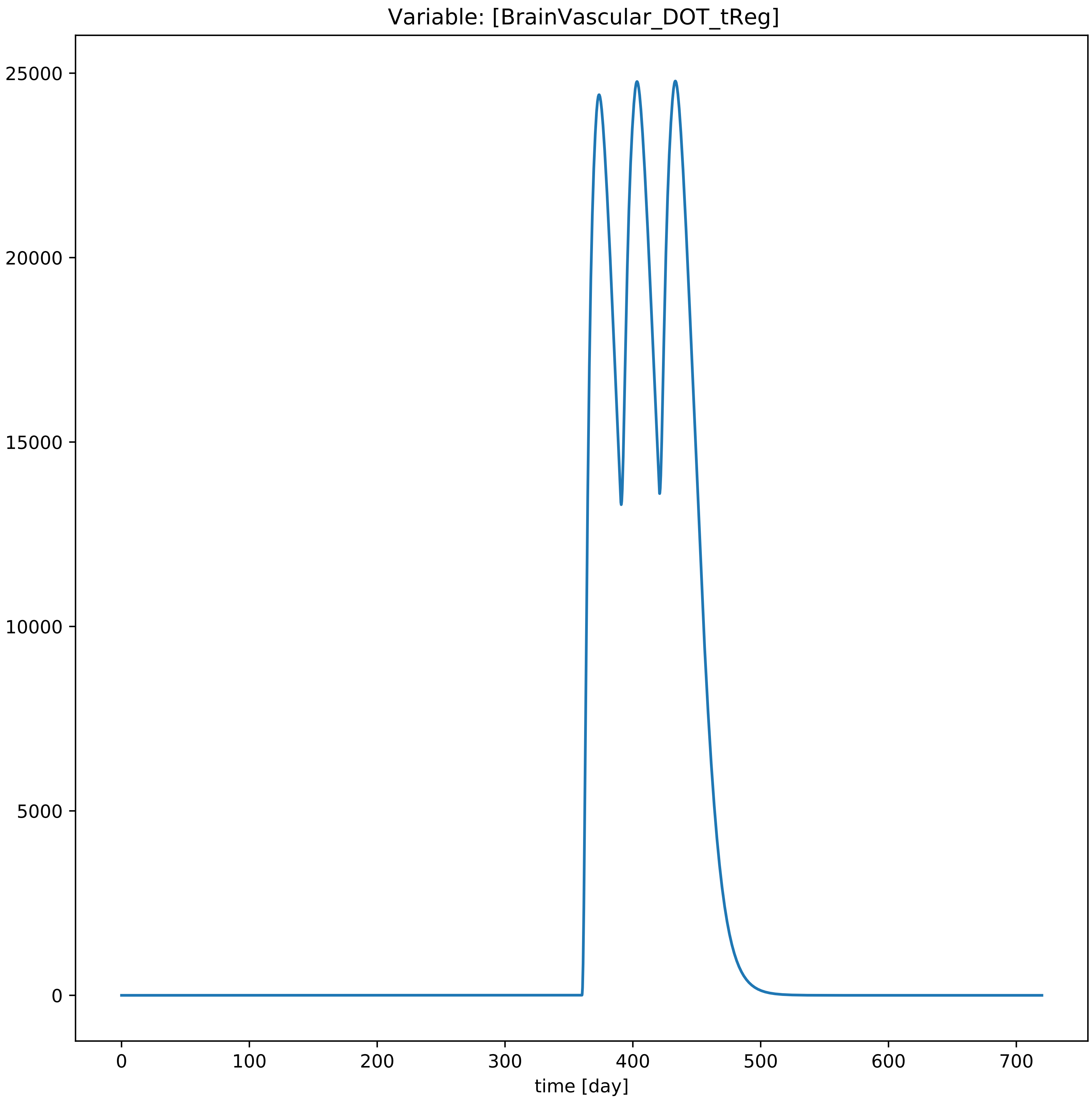

Variable: [BrainVascular_DOT_tReg]
25000
20000
15000
10000
5000
0
0
100
200
300
400
500
600
700
time [day]

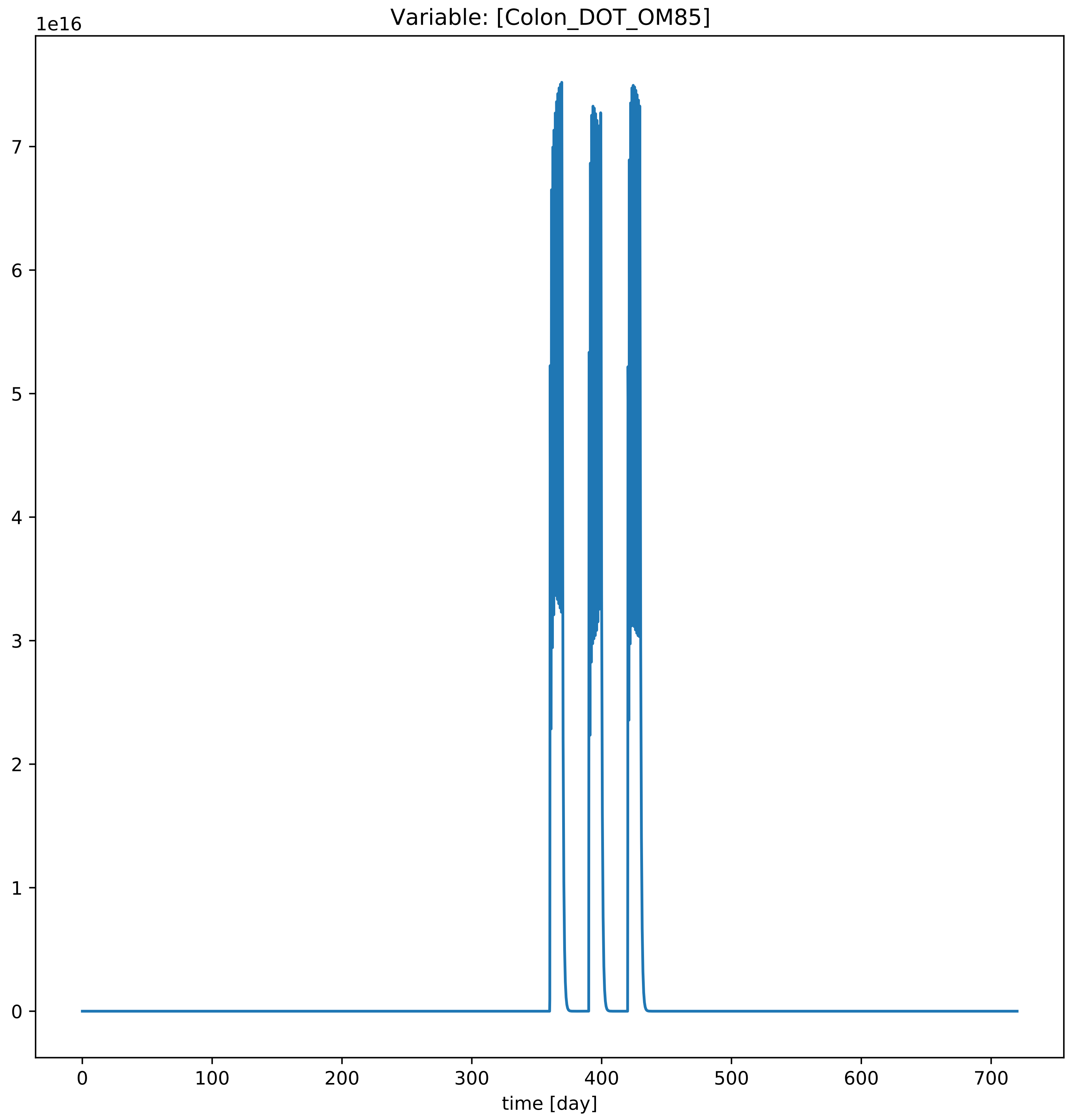
Variable: [Colon_DOT_OM85]
1e16
0
1
2
3
4
5
6
7
0
100
200
300
400
500
600
700
time [day]

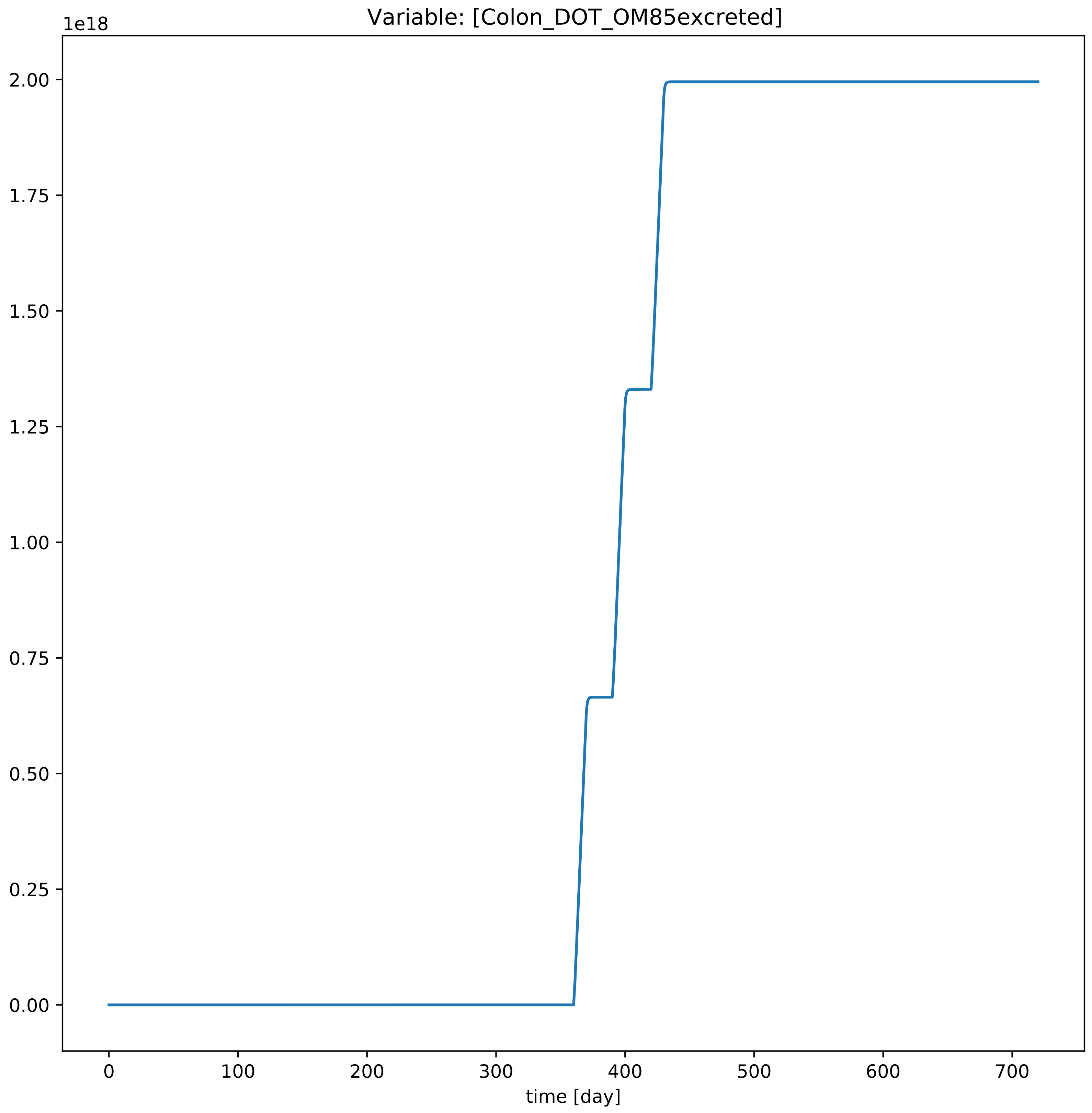

Variable: [Colon_DOT_OM85excreted]
1e18
2.00
1.75
1.50
1.25
1.00
0.75
0.50
0.25
0.00
0
100
200
300
400
500
600
700
time [day]

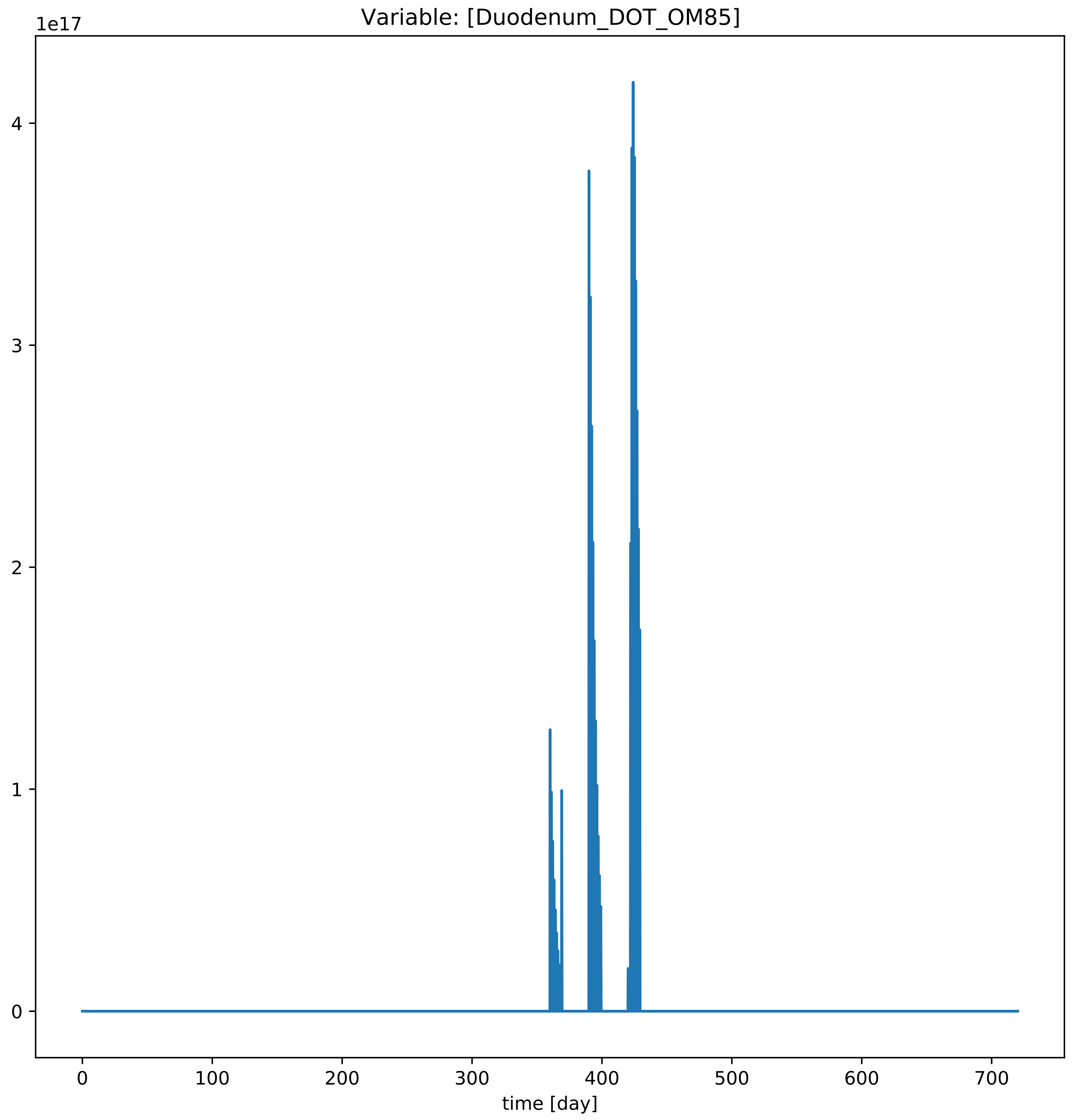

Variable: [Duodenum_DOT_OM85]
1e17
0
1
2
3
4
0
100
200
300
400
500
600
700
time [day]

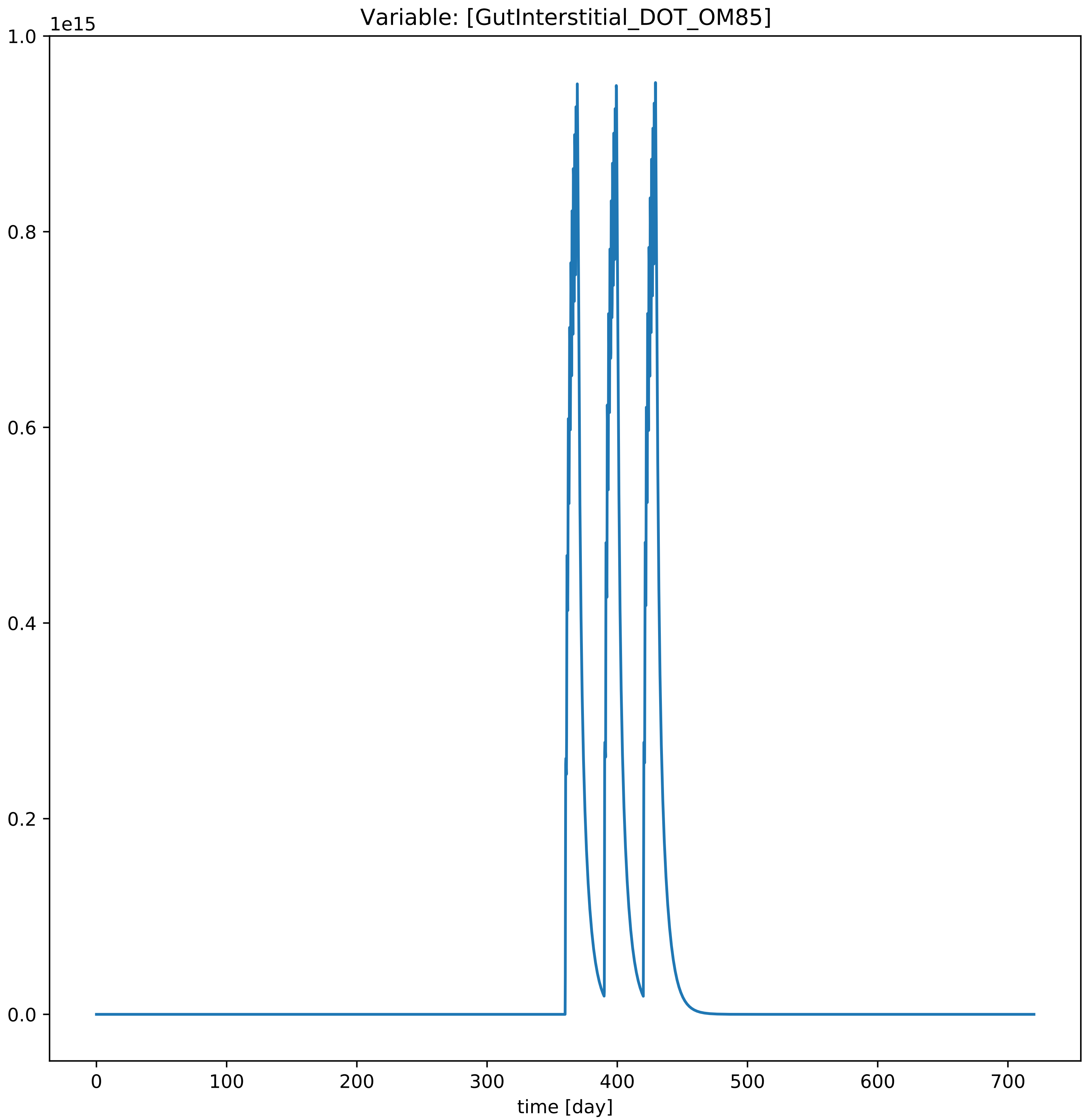

Variable: [GutInterstitial_DOT_OM85]
1e15
1.0
0.8
0.6
0.4
0.2
0.0
0
100
200
300
400
500
600
700
time [day]

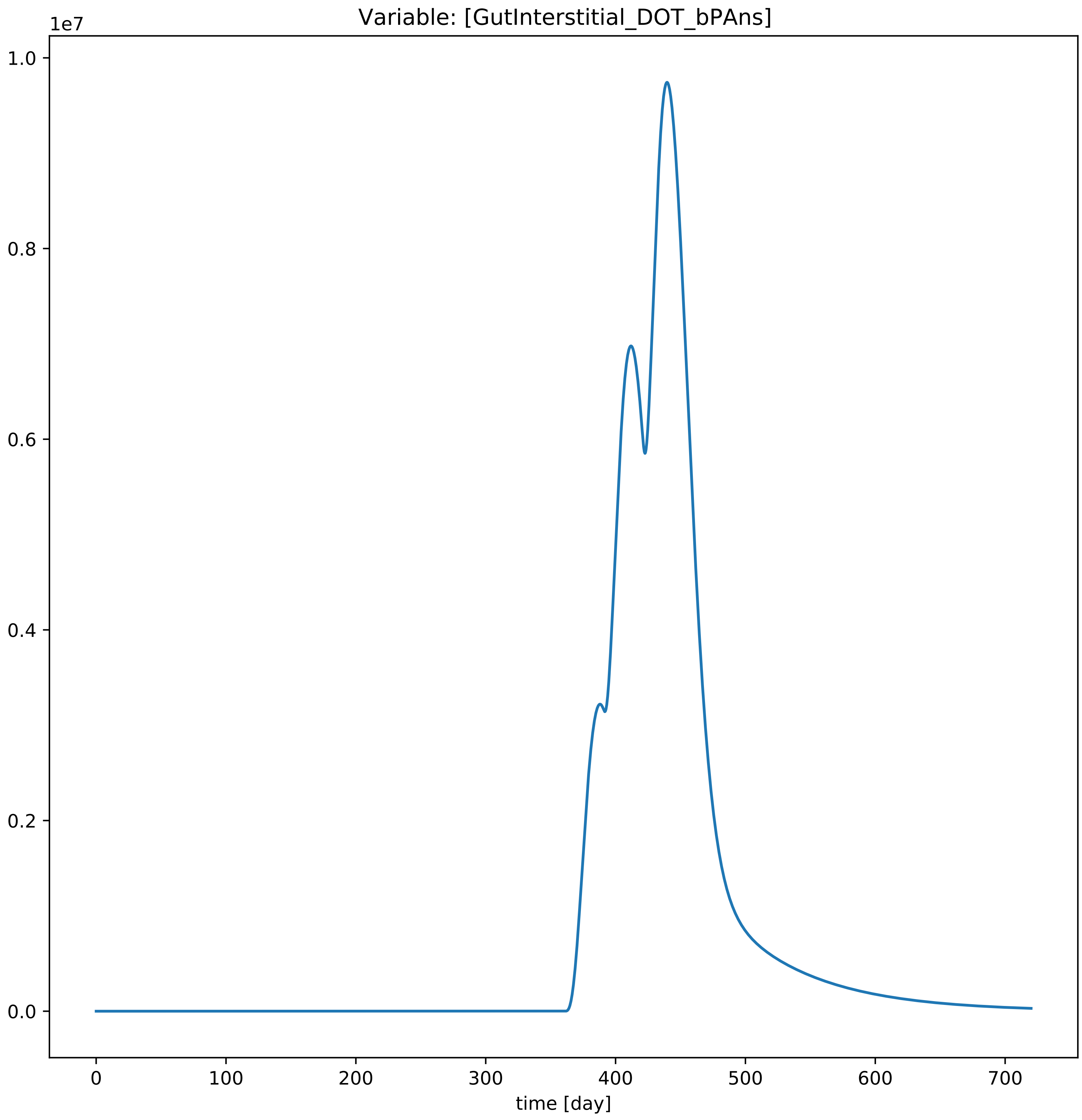

Variable: [GutInterstitial_DOT_bPAns]
1e7
1.0
0.8
0.6
0.4
0.2
0.0
0
100
200
300
400
500
600
700
time [day]

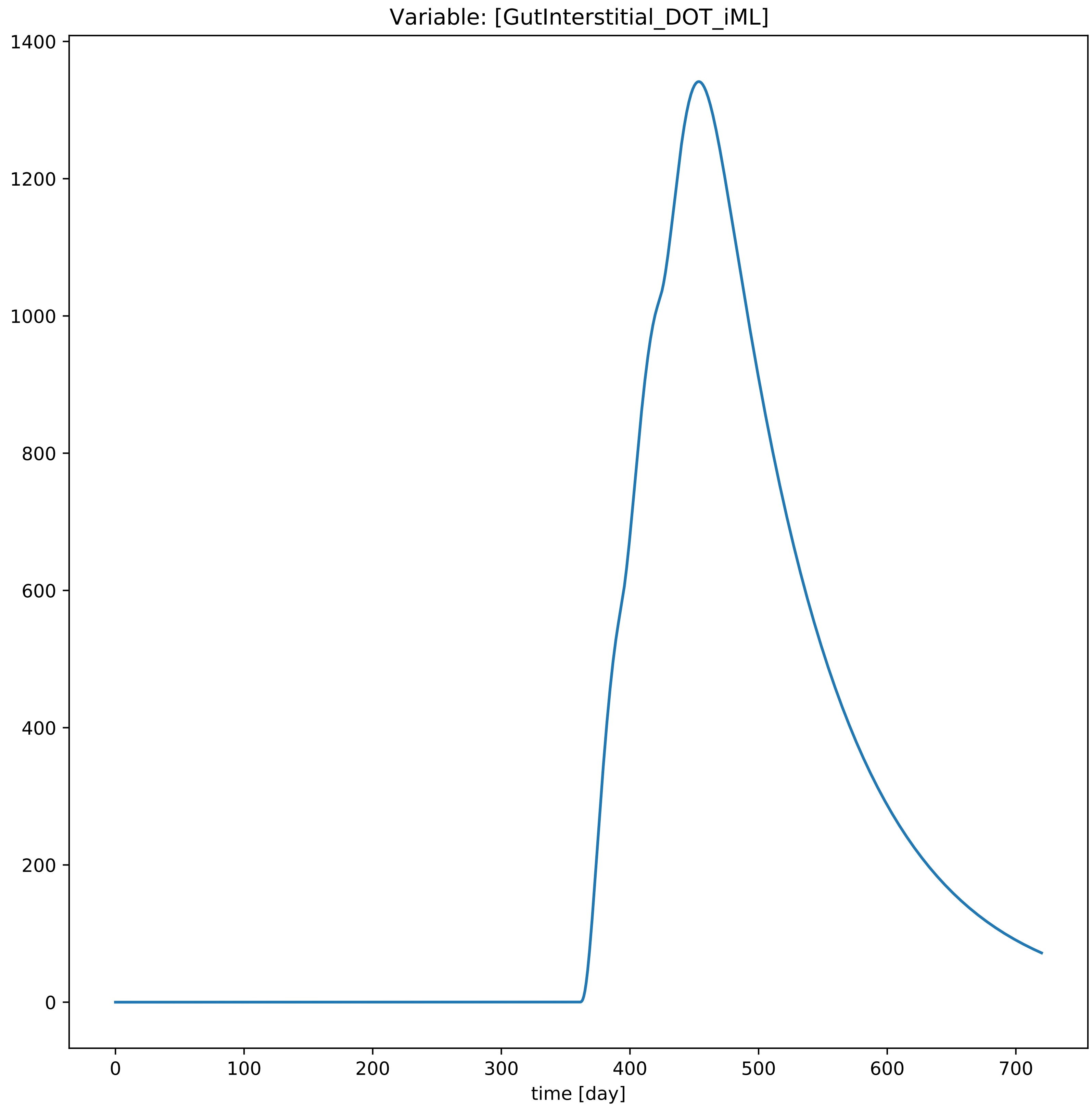
Variable: [GutInterstitial_DOT_iML]
1400
1200
1000
800
600
400
200
0
0
100
200
300
400
500
600
700
time [day]

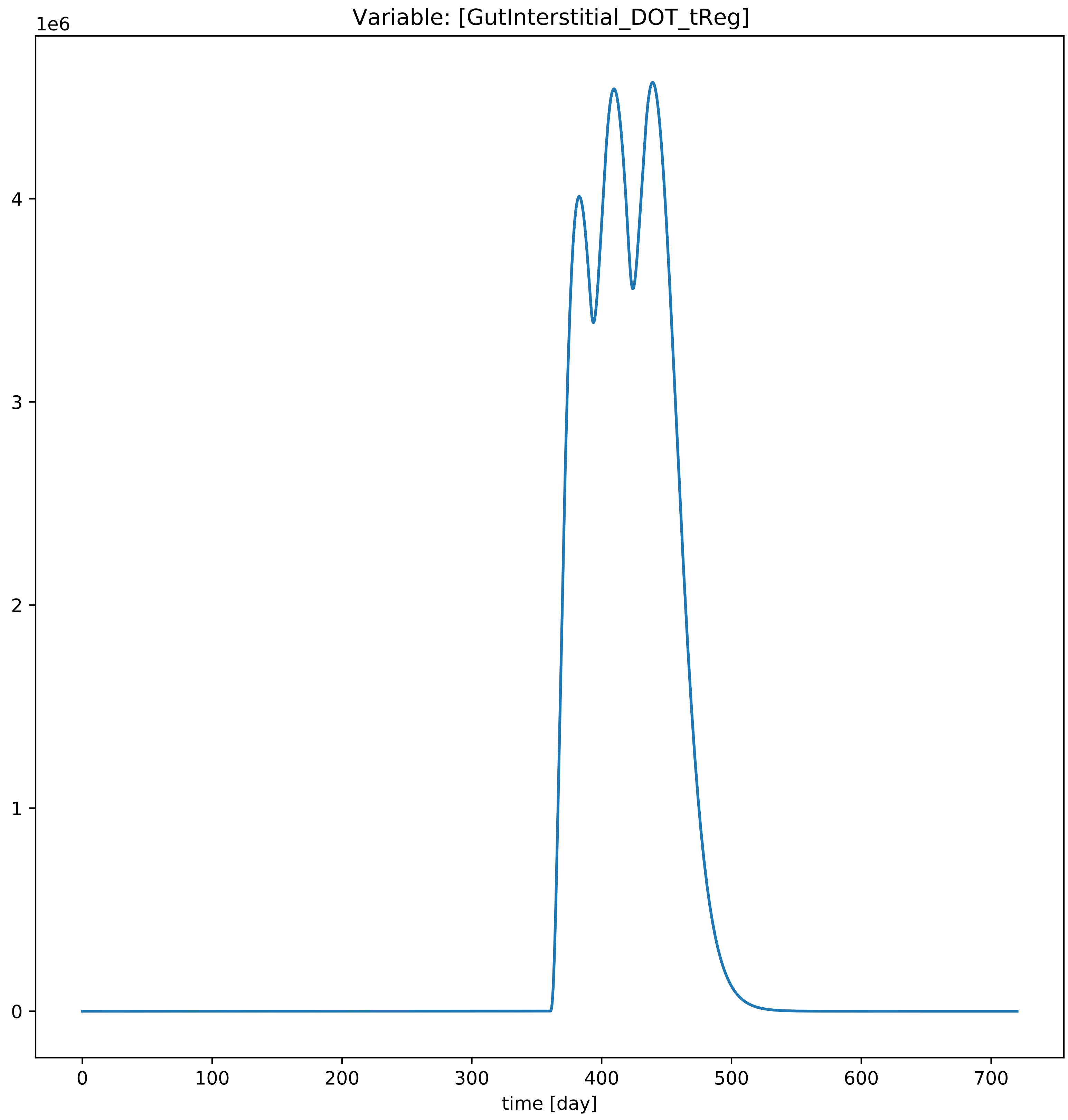

Variable: [GutInterstitial_DOT_tReg]
1e6
0
1
2
3
4
0
100
200
300
400
500
600
700
time [day]

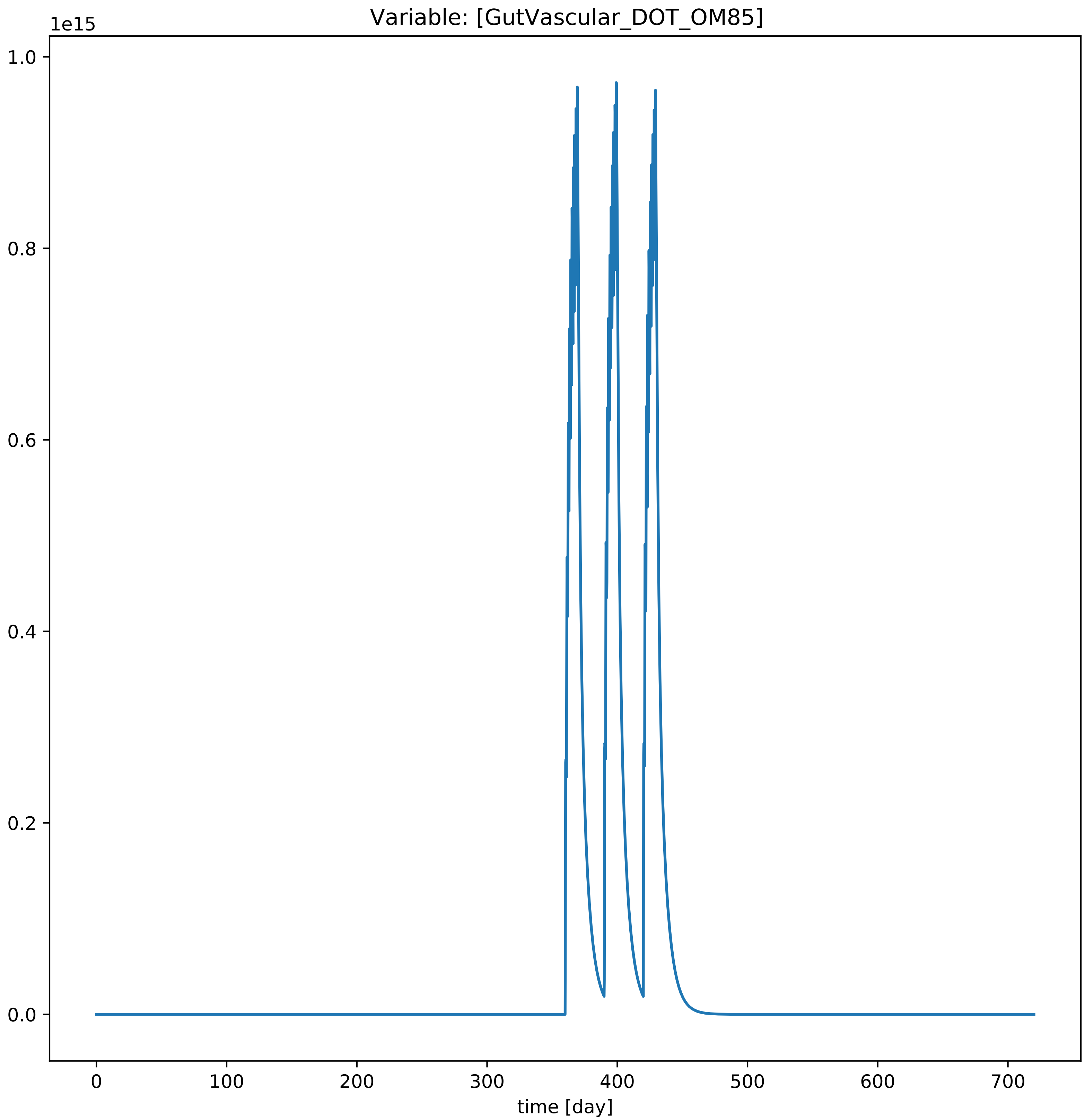

Variable: [GutVascular_DOT_OM85]
1e15
1.0
0.8
0.6
0.4
0.2
0.0
0
100
200
300
400
500
600
700
time [day]

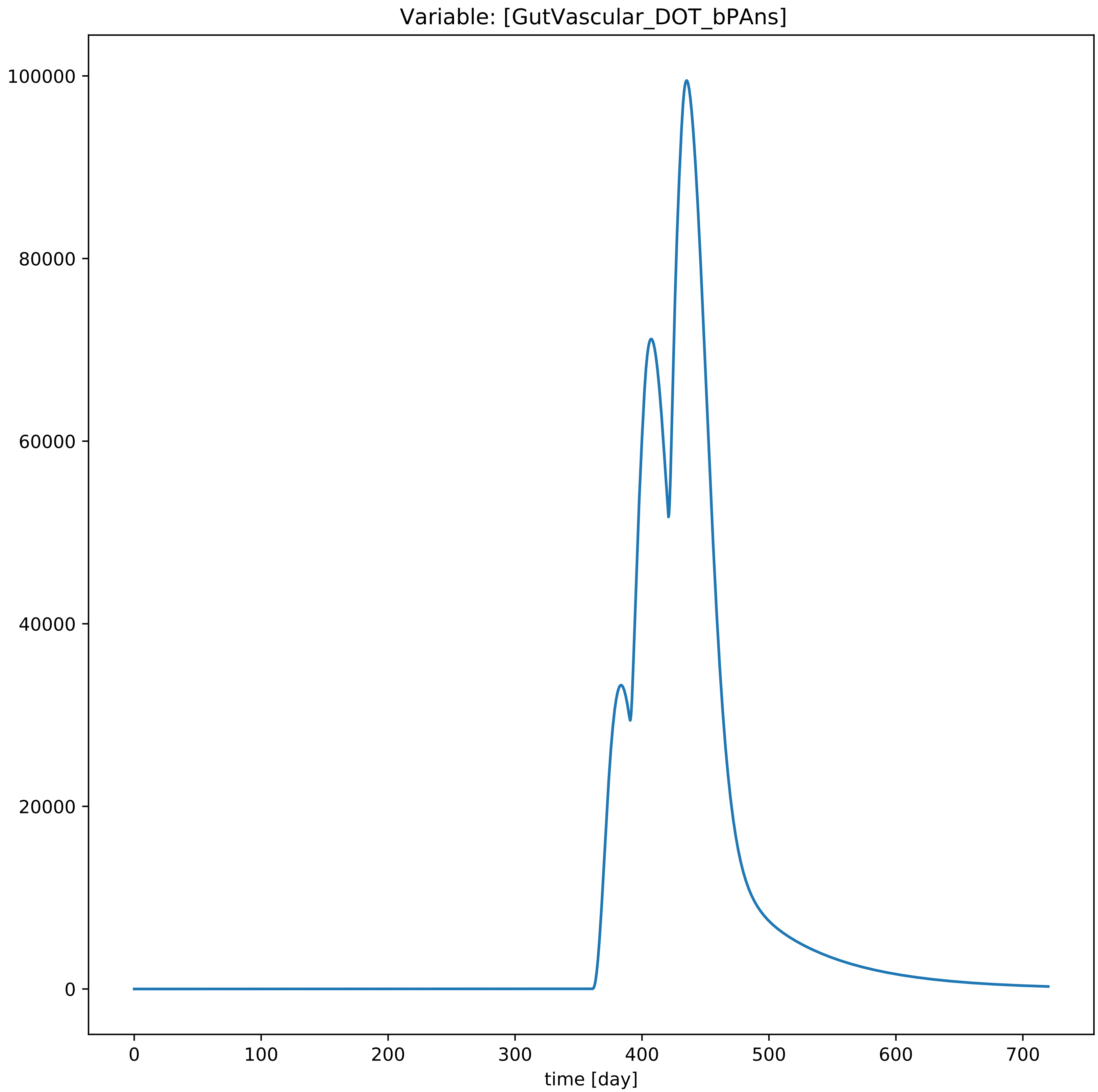
Variable: [GutVascular_DOT_bPAns]
100000
80000
60000
40000
20000
0
0
100
200
300
400
500
600
700
time [day]

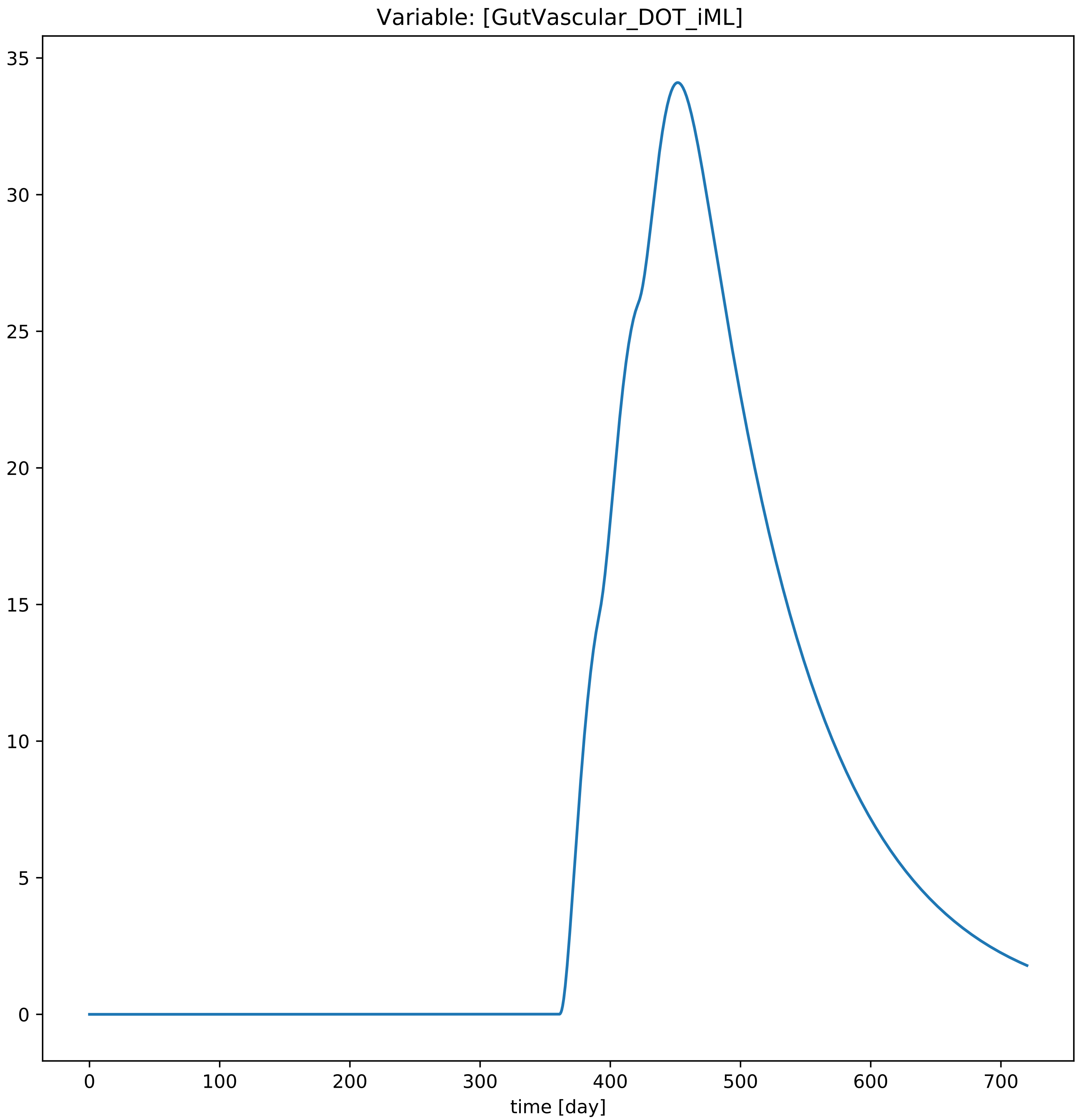

Variable: [GutVascular_DOT_iML]
35
30
25
20
15
10
5
0
0
100
200
300
400
500
600
700
time [day]

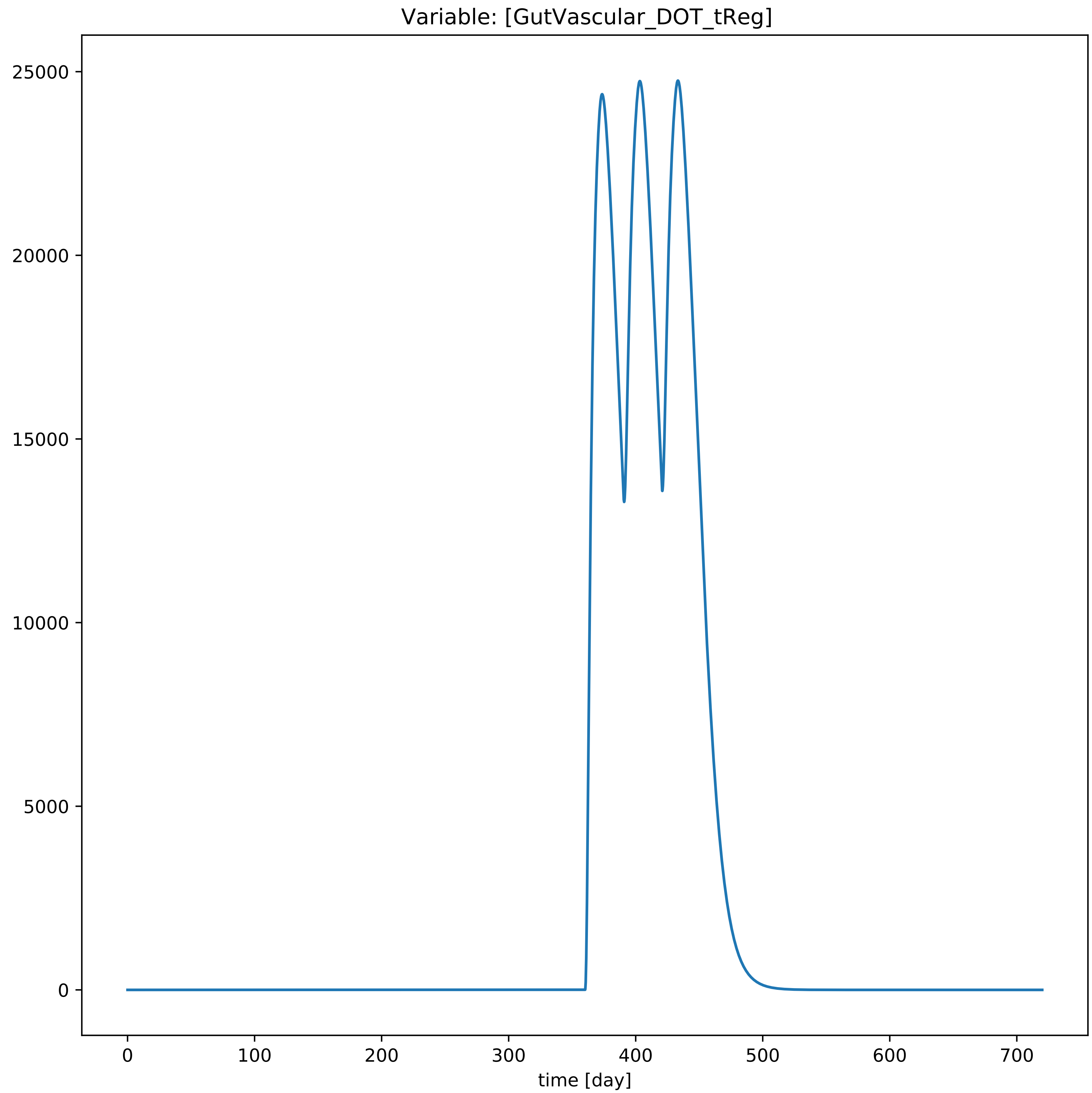

Variable: [GutVascular_DOT_tReg]
25000
20000
15000
10000
5000
0
0
100
200
300
400
500
600
700
time [day]

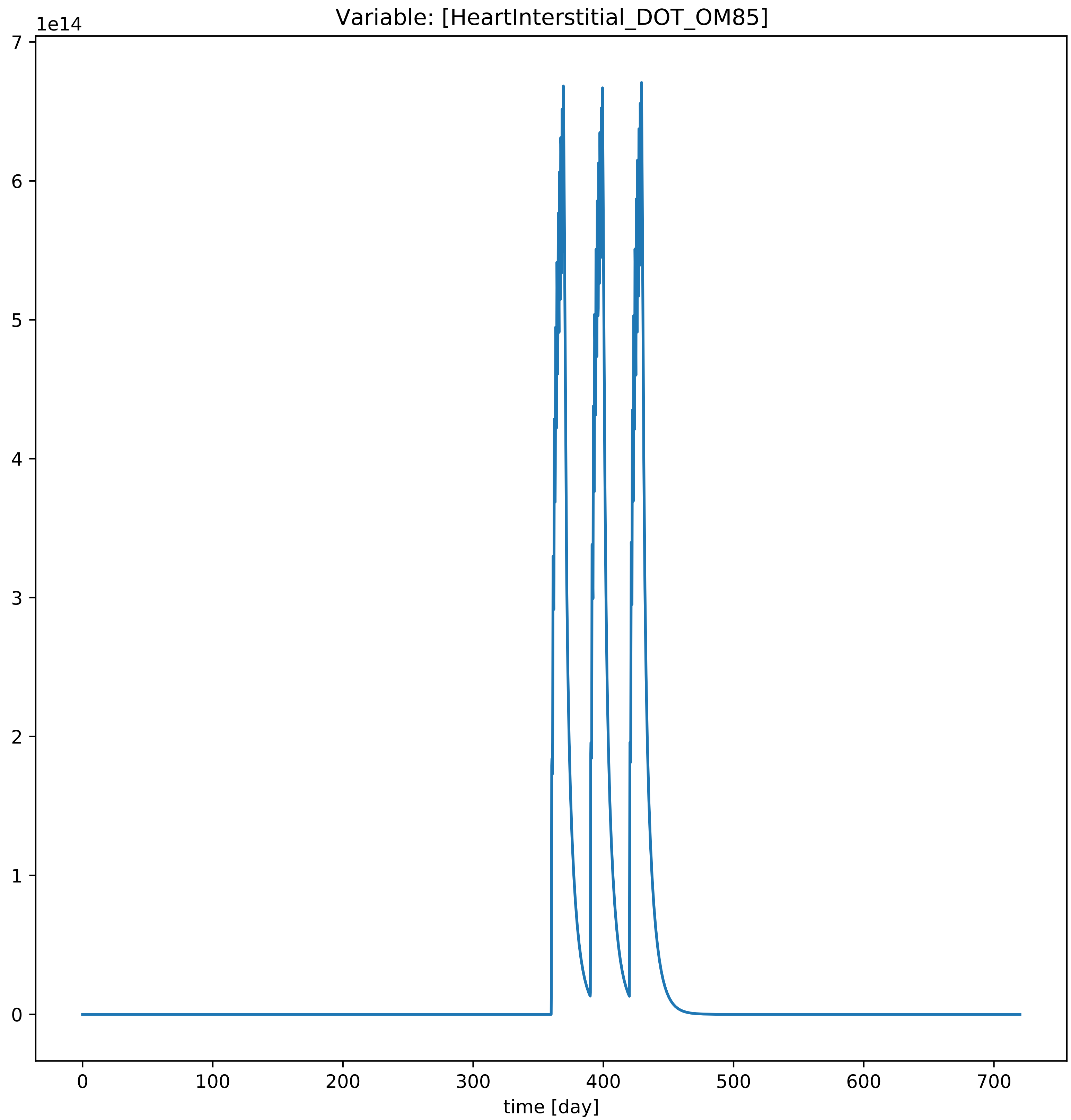

Variable: [HeartInterstitial_DOT_OM85]
1e14
7
6
5
4
3
2
1
0
0
100
200
300
400
500
600
700
time [day]

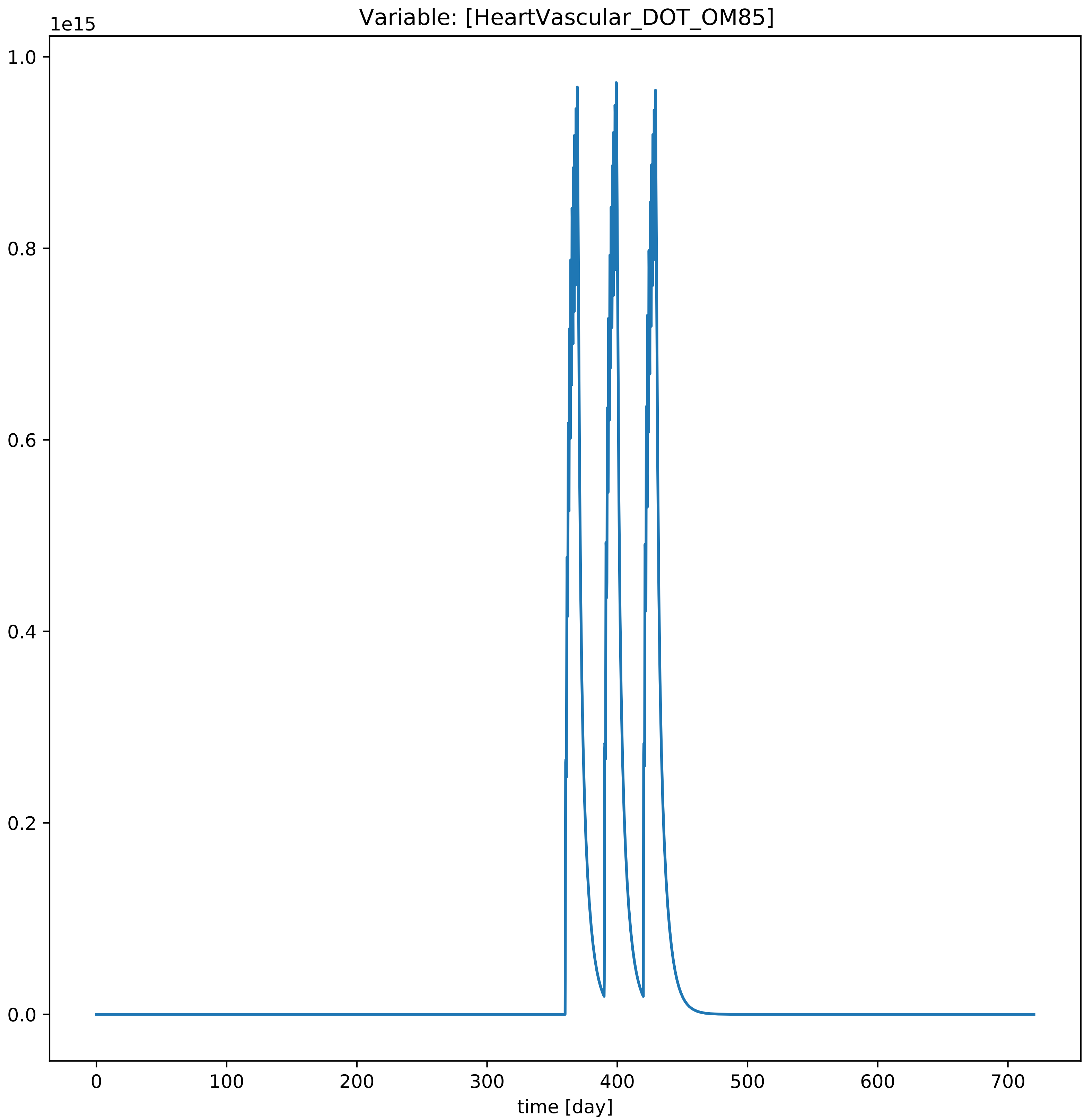

Variable: [HeartVascular_DOT_OM85]
1e15
1.0
0.8
0.6
0.4
0.2
0.0
0
100
200
300
400
500
600
700
time [day]

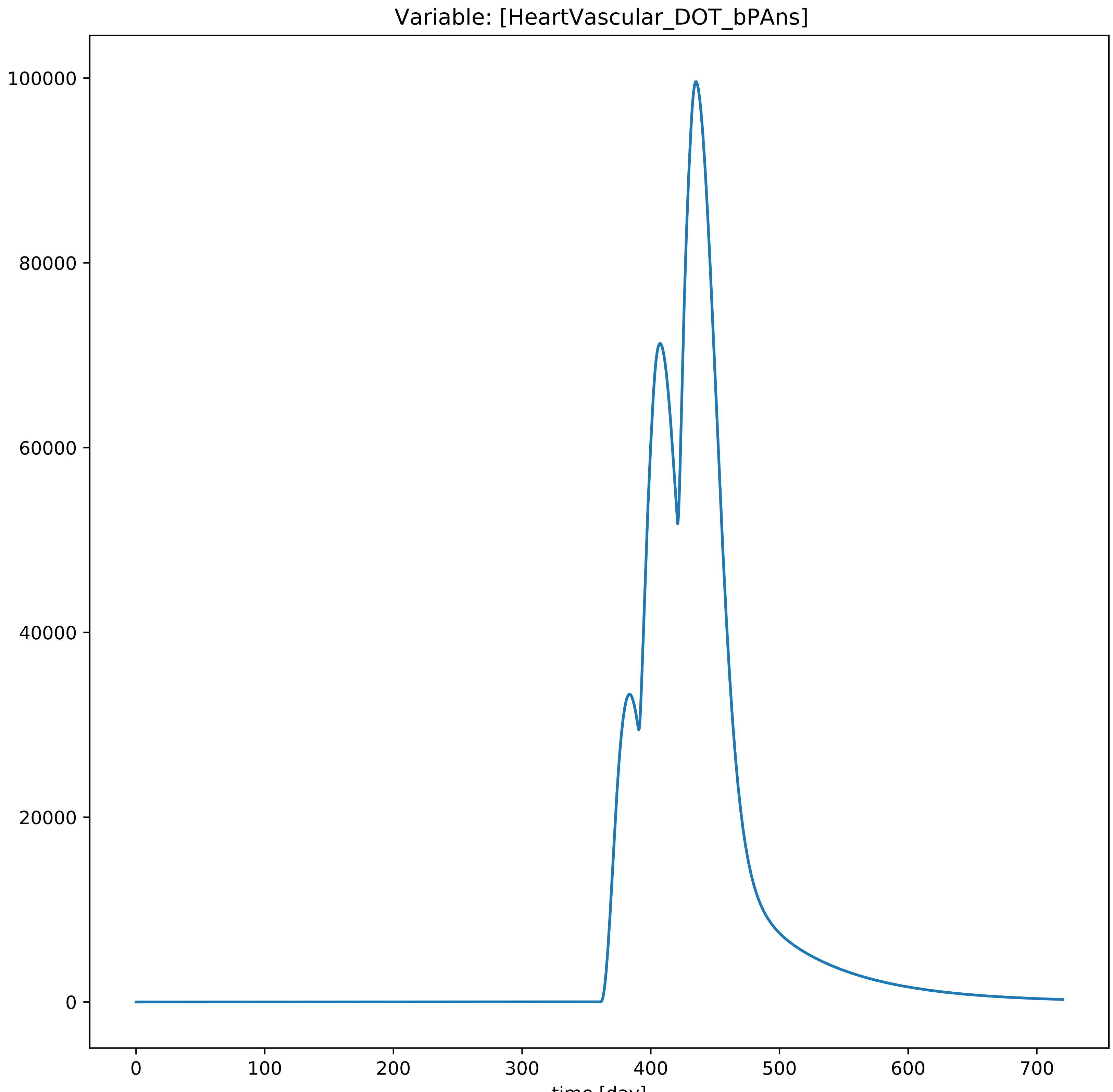

Variable: [HeartVascular_DOT_bPAns]
100000
80000
60000
40000
20000
0
0
100
200
300
400
500
600
700
time [day]

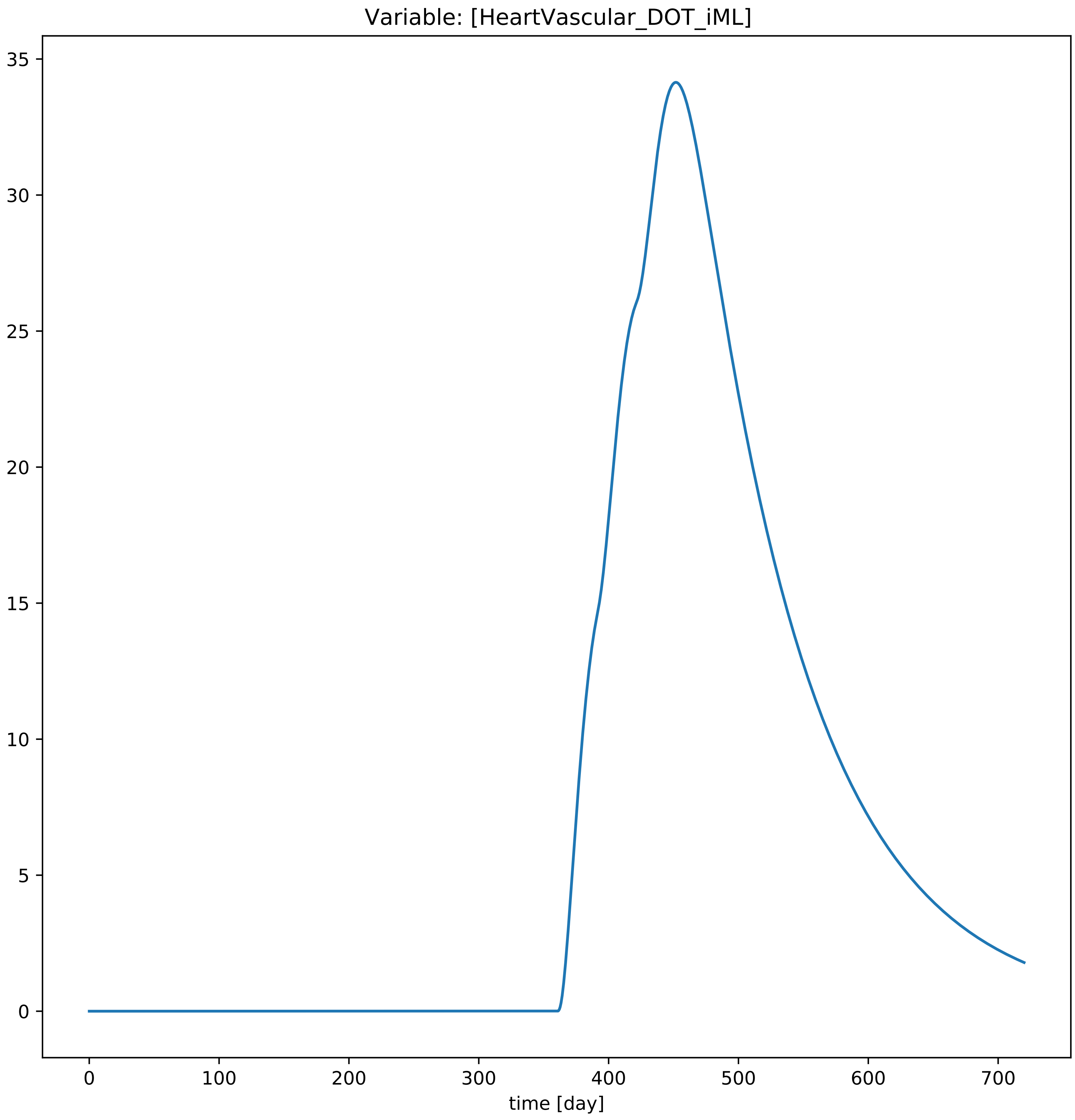

Variable: [HeartVascular_DOT_iML]
35
30
25
20
15
10
5
0
0
100
200
300
400
500
600
700
time [day]

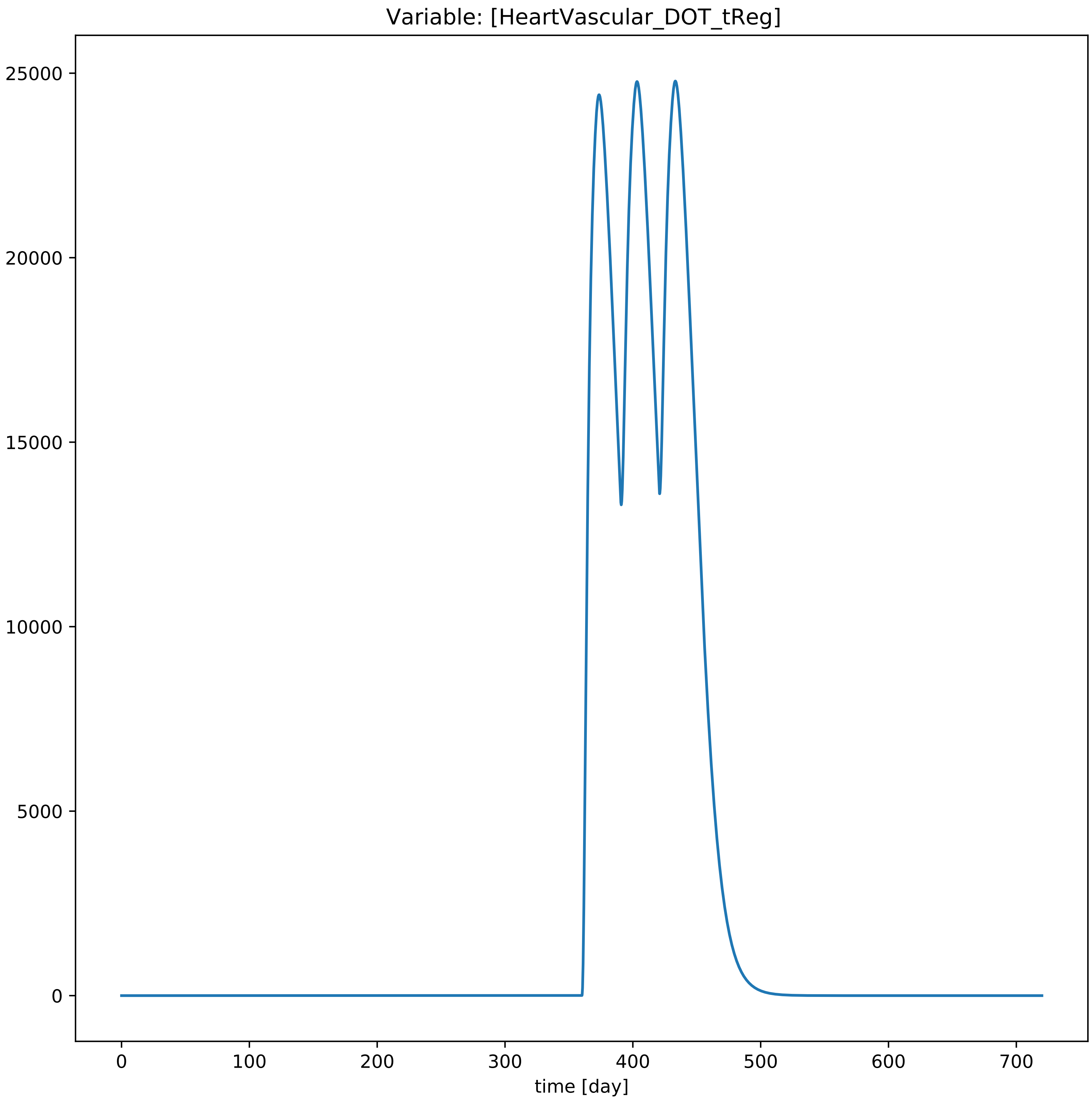
Variable: [HeartVascular_DOT_tReg]
25000
20000
15000
10000
5000
0
0
100
200
300
400
500
600
700
time [day]

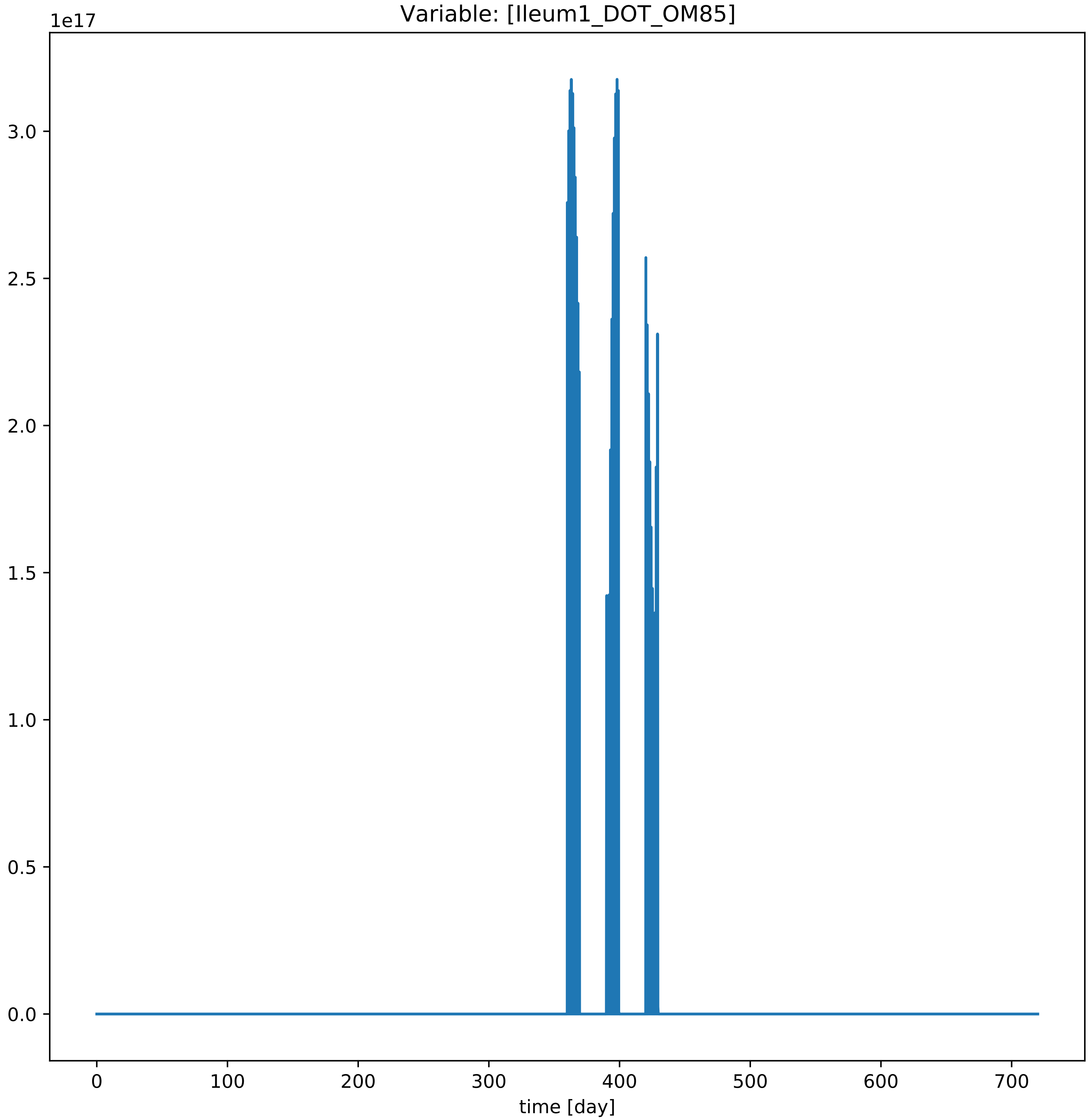

Variable: [Ileum1_DOT_OM85]
1e17
3.0
2.5
2.0
1.5
1.0
0.5
0.0
0
100
200
300
400
500
600
700
time [day]

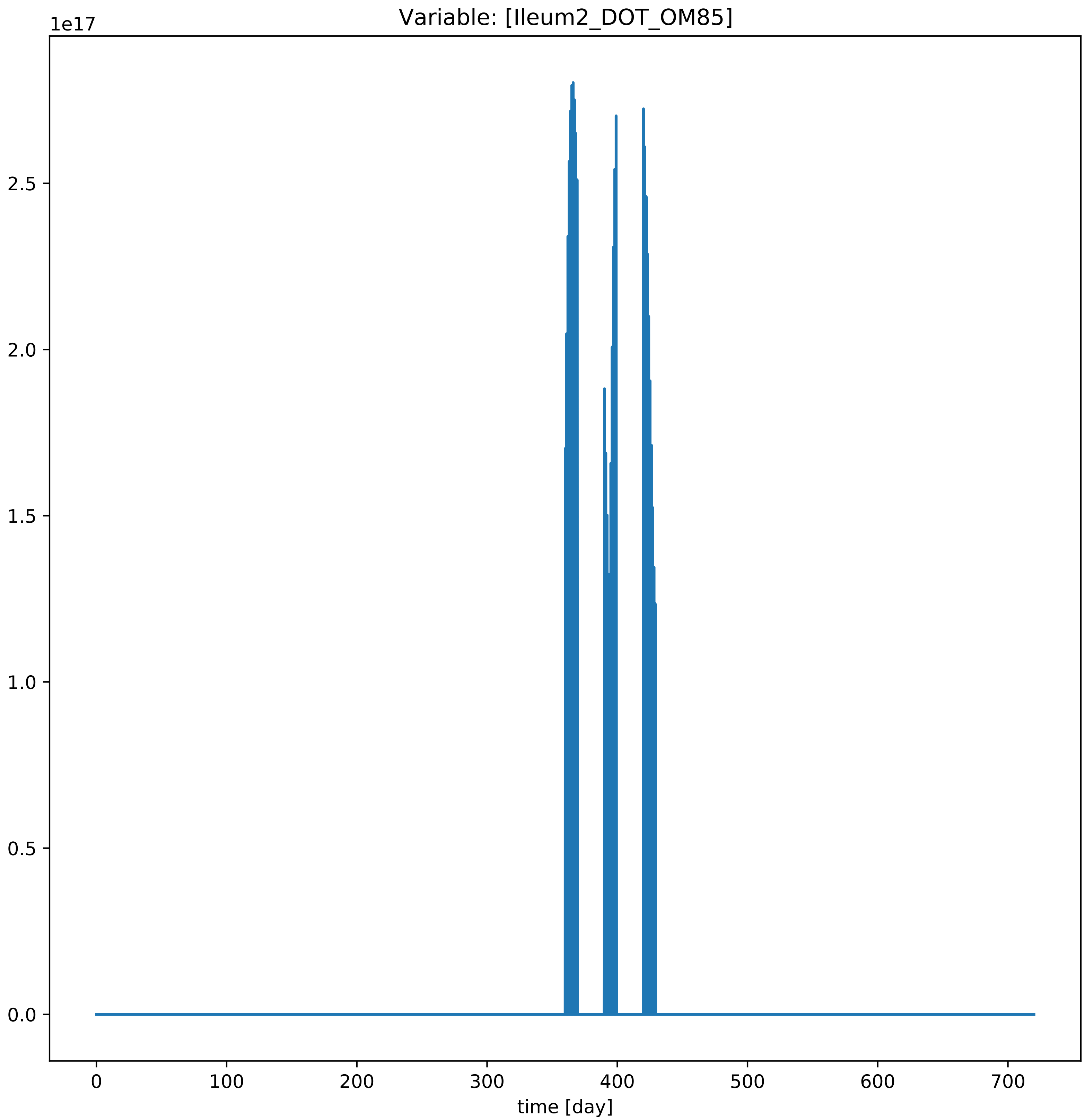
Variable: [Ileum2_DOT_OM85]
1e17
2.5
2.0
1.5
1.0
0.5
0.0
0
100
200
300
400
500
600
700
time [day]

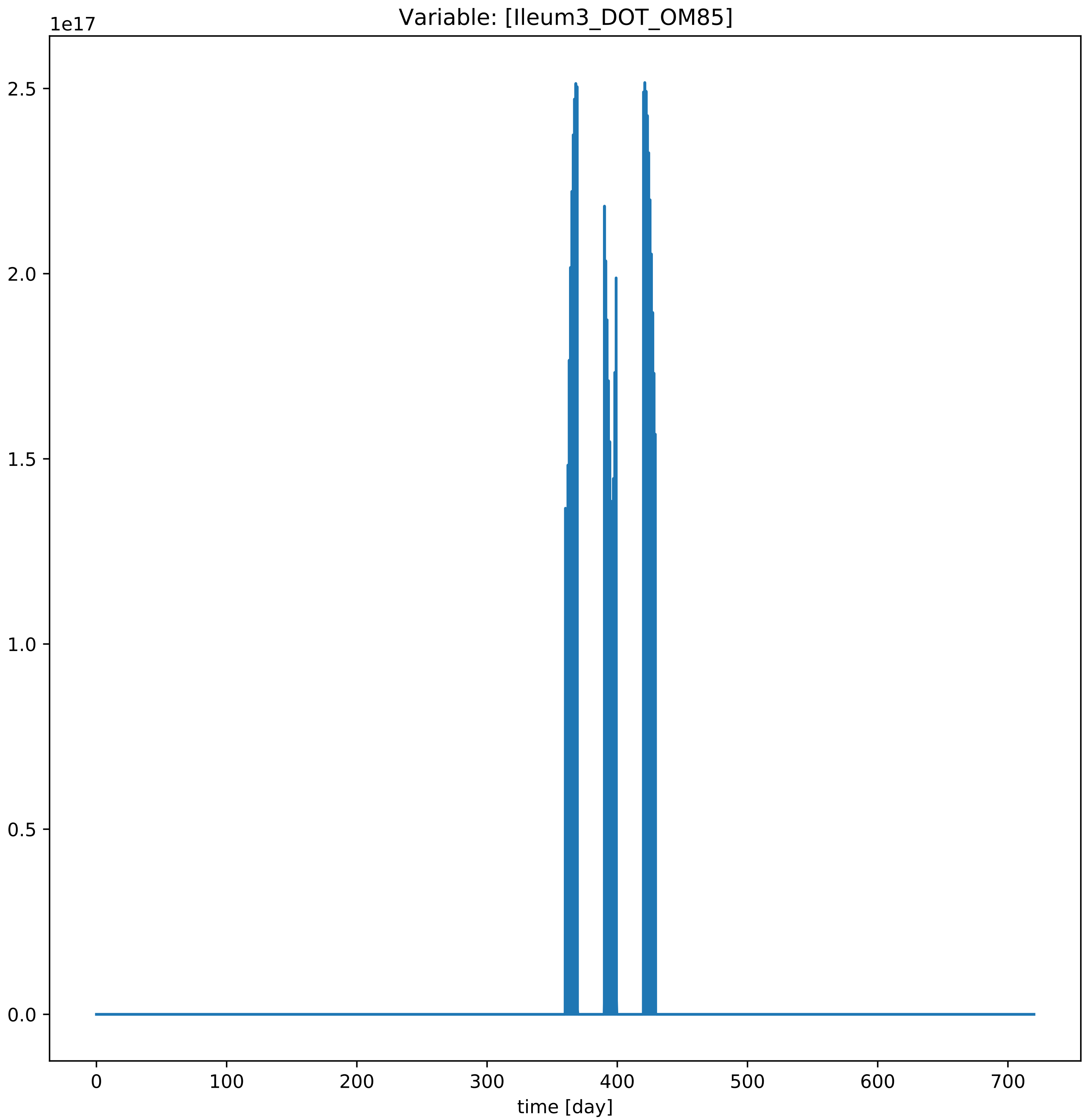

Variable: [Ileum3_DOT_OM85]
1e17
2.5
2.0
1.5
1.0
0.5
0.0
0
100
200
300
400
500
600
700
time [day]

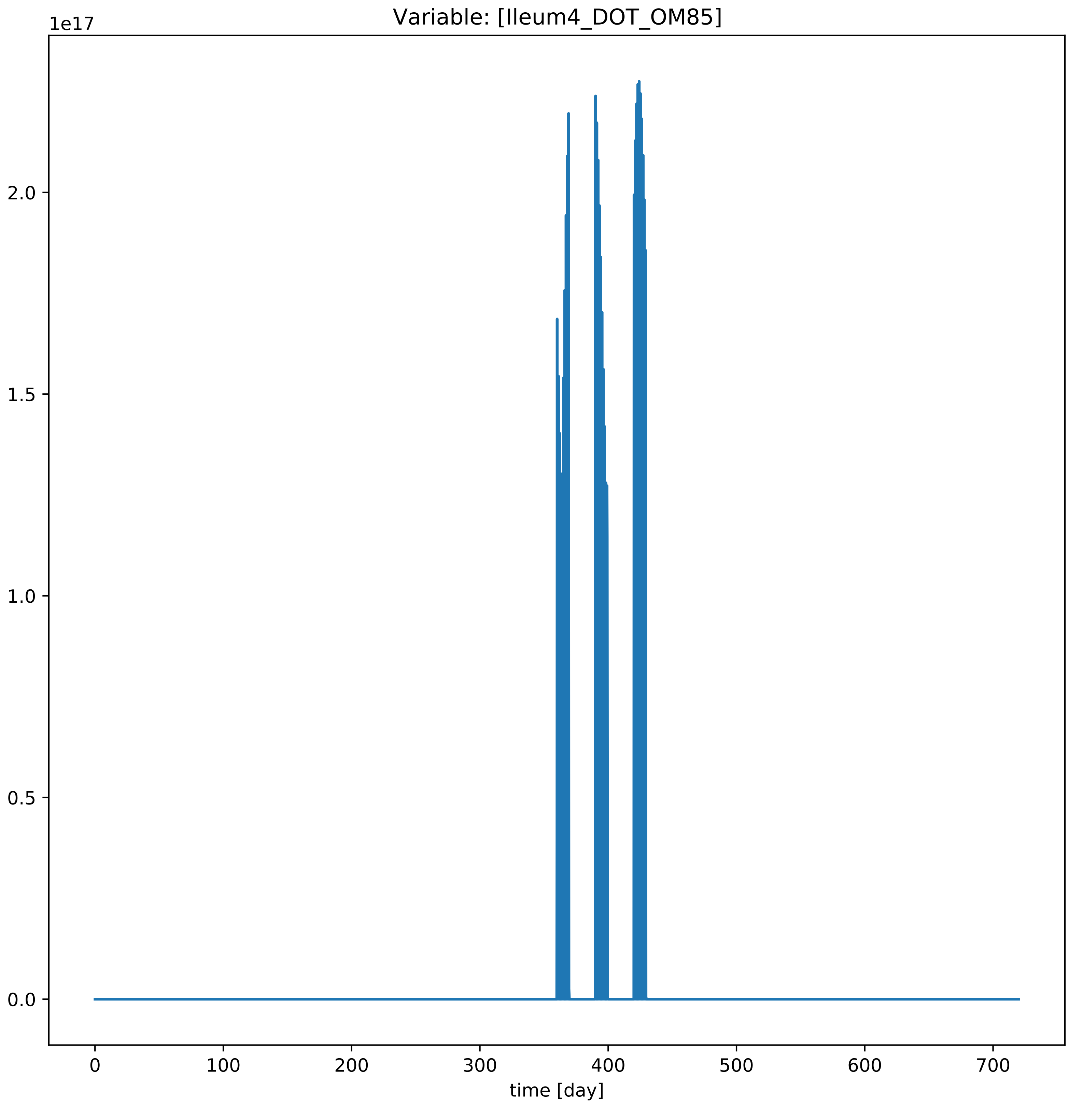

Variable: [Ileum4_DOT_OM85]
1e17
2.0
1.5
1.0
0.5
0.0
0
100
200
300
400
500
600
700
time [day]

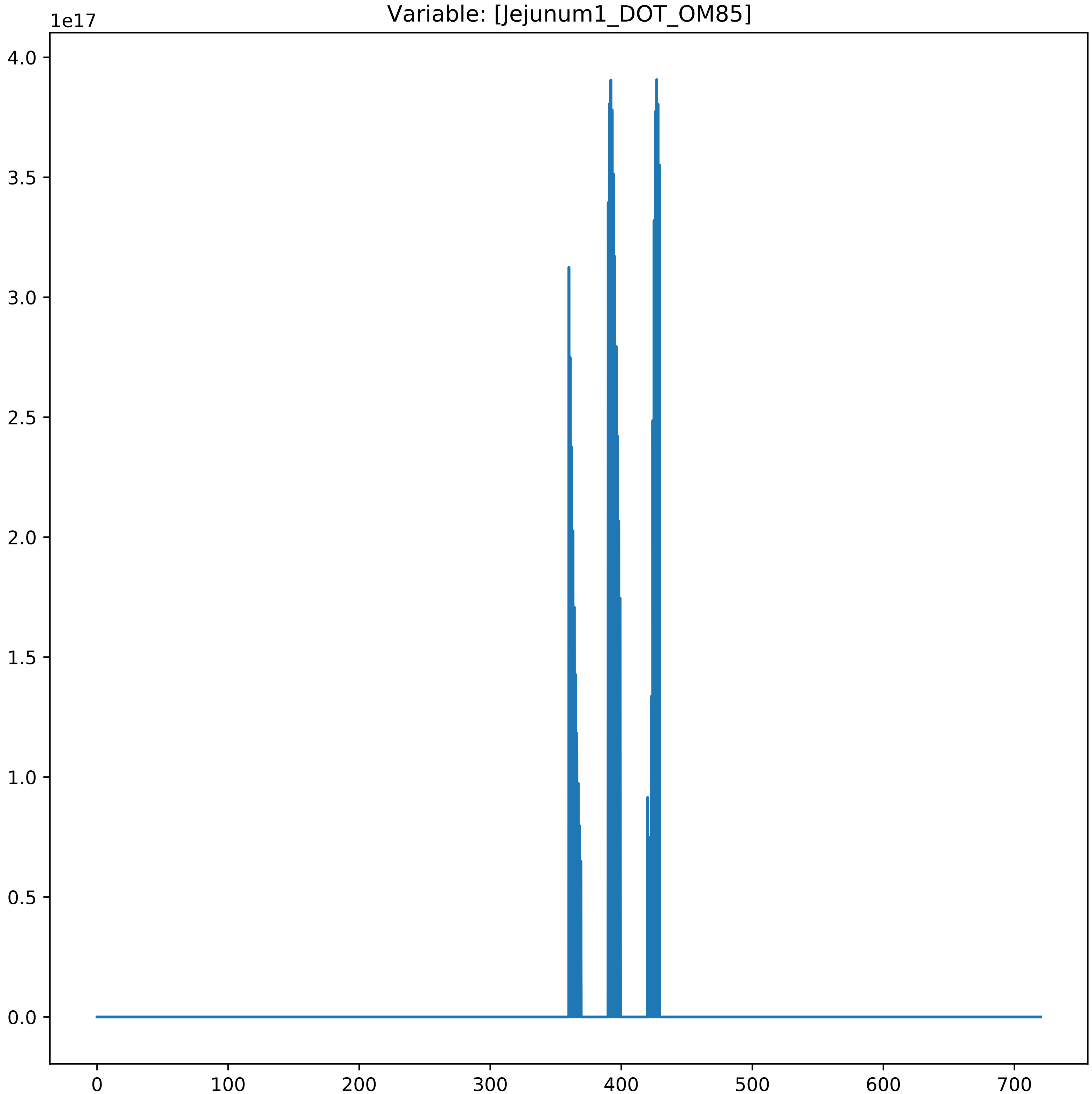

Variable: [Jejunum1_DOT_OM85]
1e17
4.0
3.5
3.0
2.5
2.0
1.5
1.0
0.5
0.0
0
100
200
300
400
500
600
700
time [day]

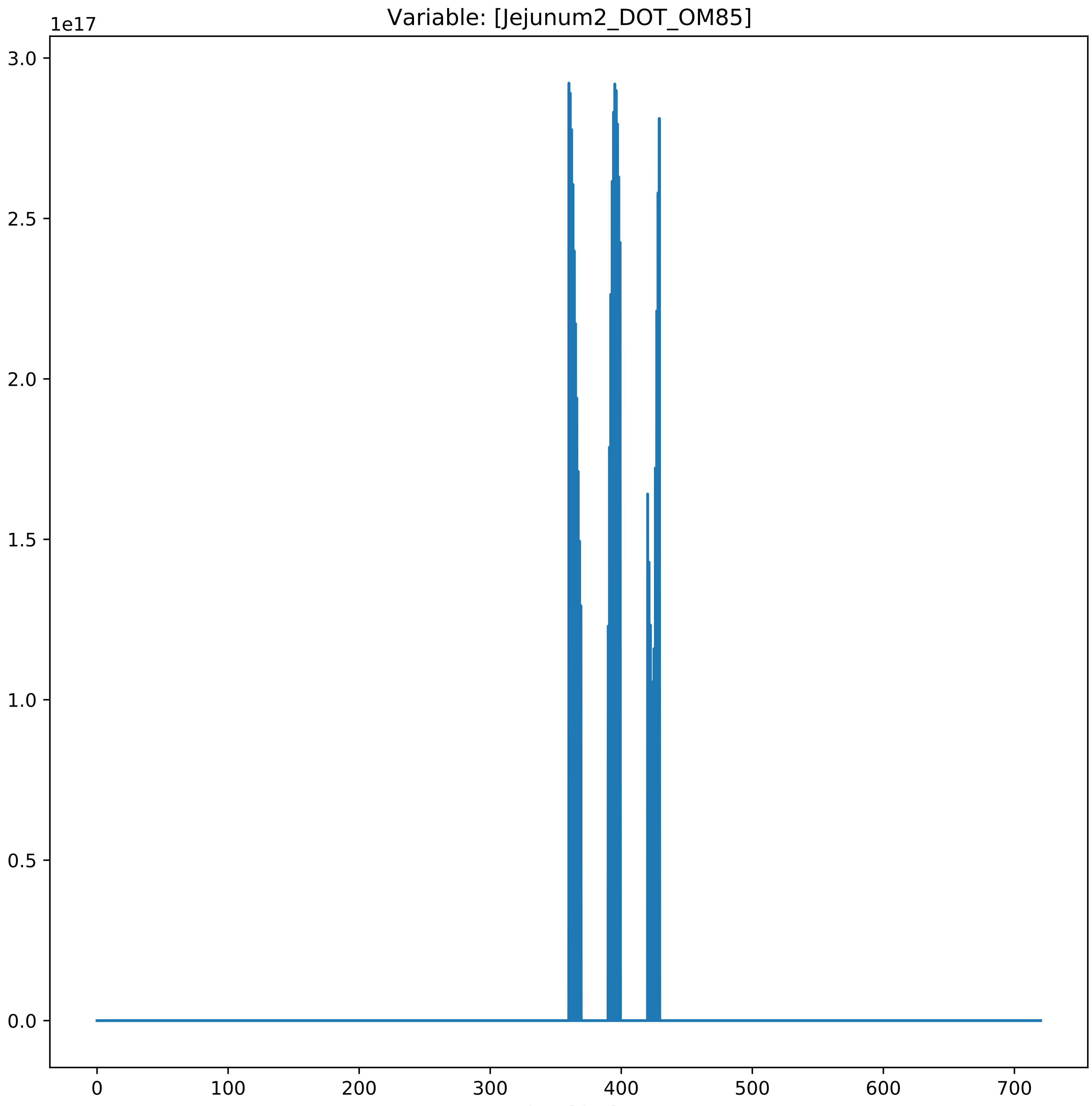
Variable: [Jejunum2_DOT_OM85]
1e17
3.0
2.5
2.0
1.5
1.0
0.5
0.0
0
100
200
300
400
500
600
700
time [day]

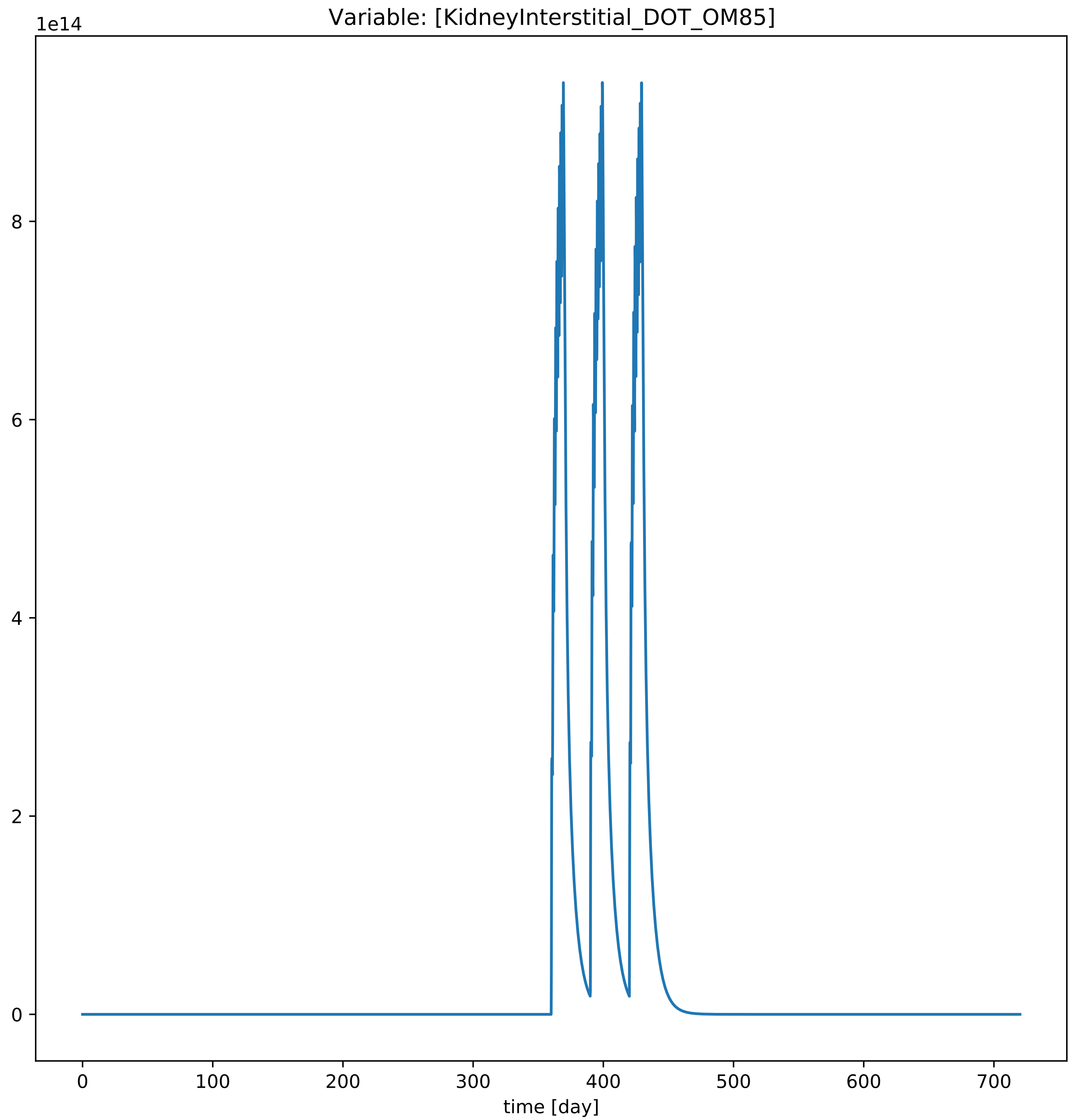

Variable: [KidneyInterstitial_DOT_OM85]
1e14
8
6
4
2
0
0
100
200
300
400
500
600
700
time [day]

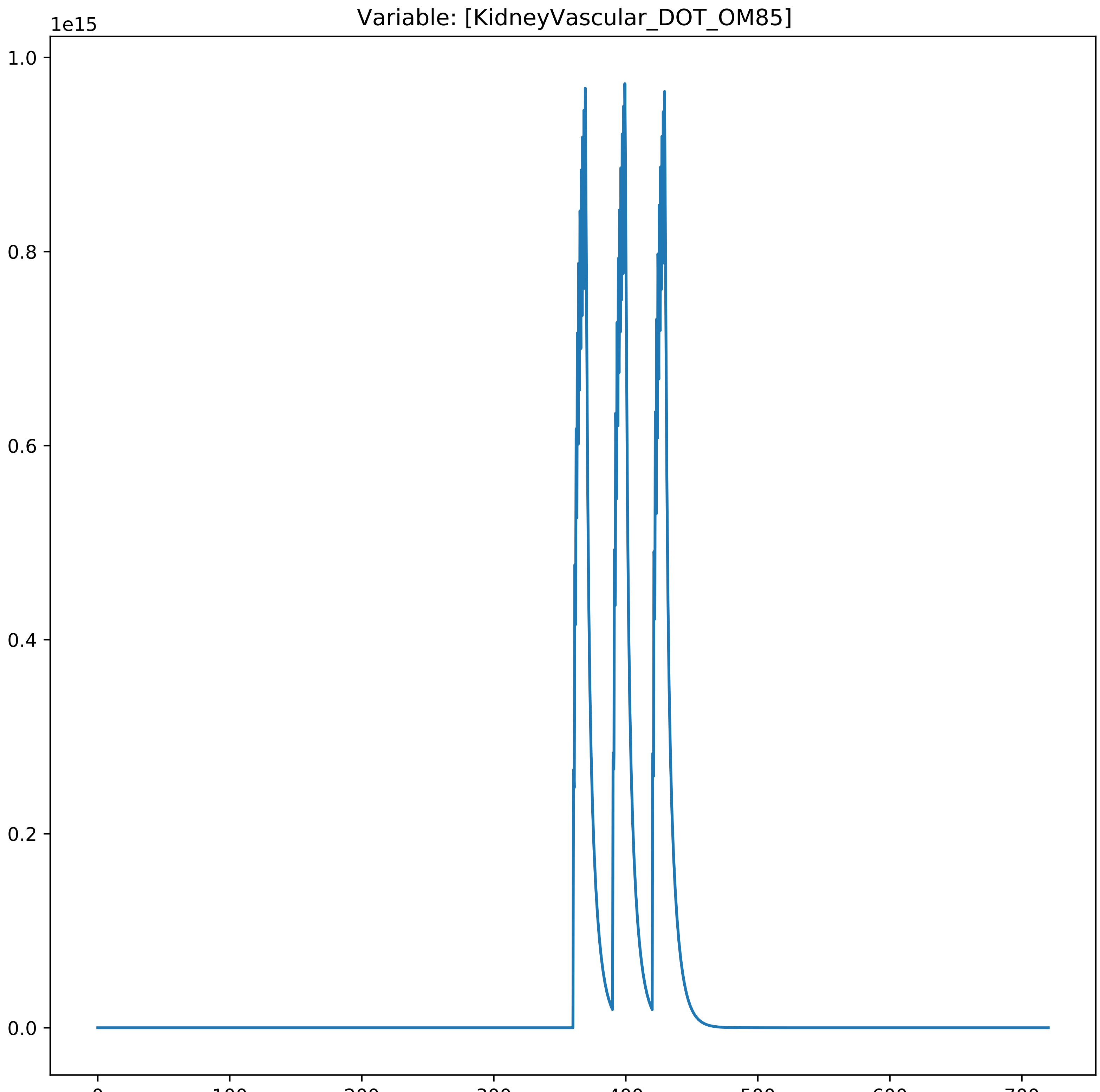

Variable: [KidneyVascular_DOT_OM85]
1e15
1.0
0.8
0.6
0.4
0.2
0.0
0
100
200
300
400
500
600
700
time [day]

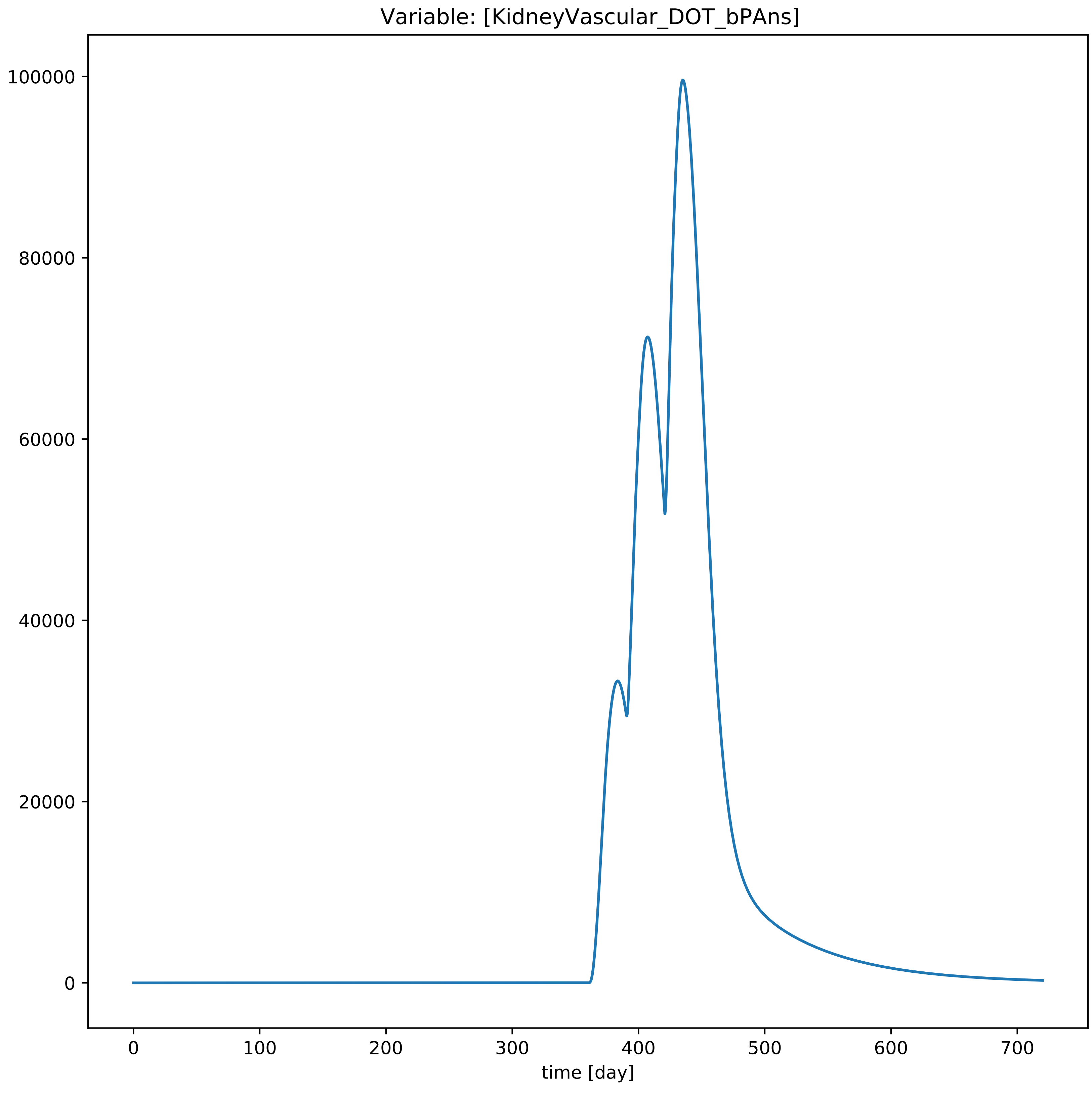
Variable: [KidneyVascular_DOT_bPAns]
100000
80000
60000
40000
20000
0
0
100
200
300
400
500
600
700
time [day]

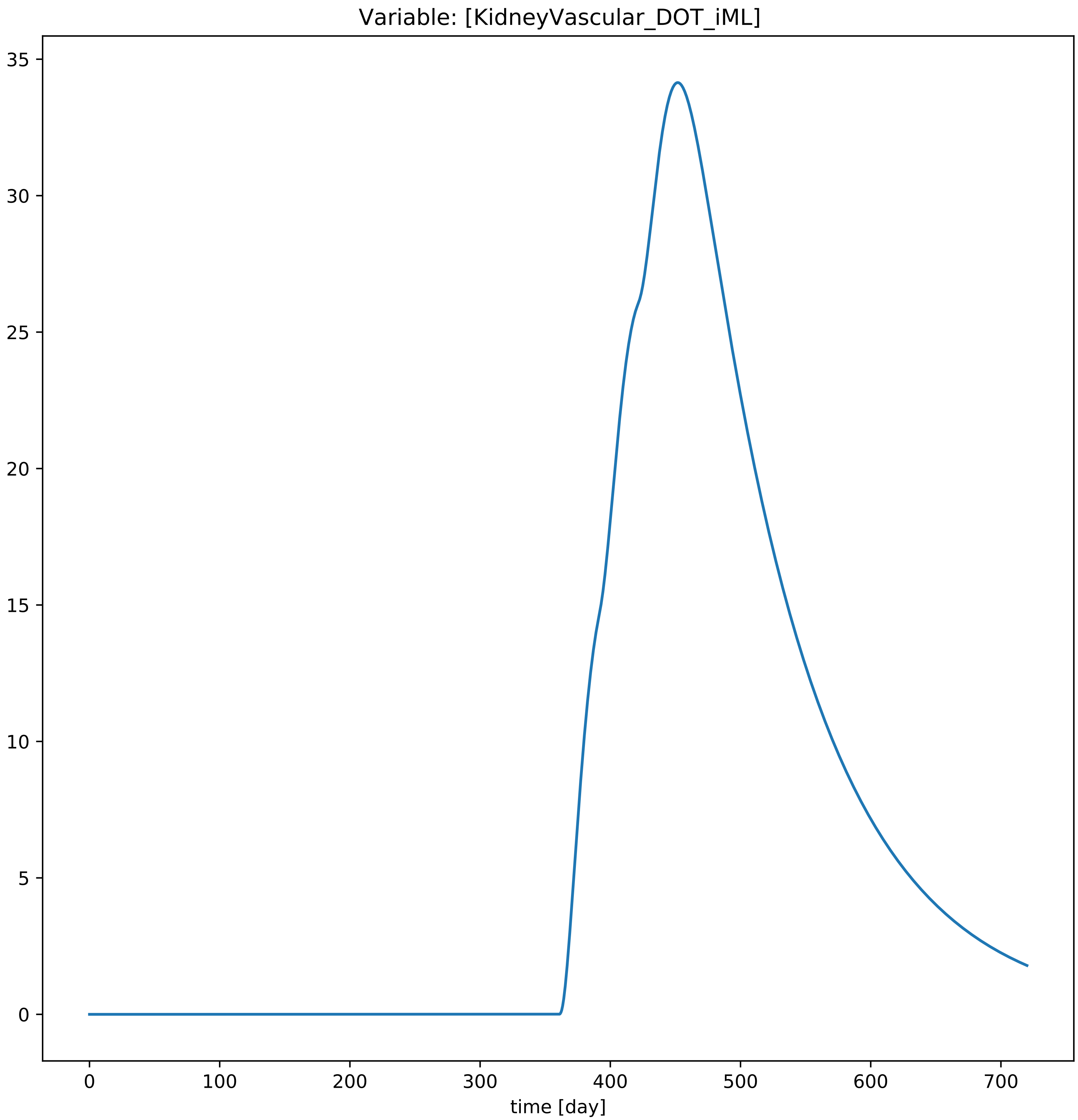

Variable: [KidneyVascular_DOT_iML]
35
30
25
20
15
10
5
0
0
100
200
300
400
500
600
700
time [day]

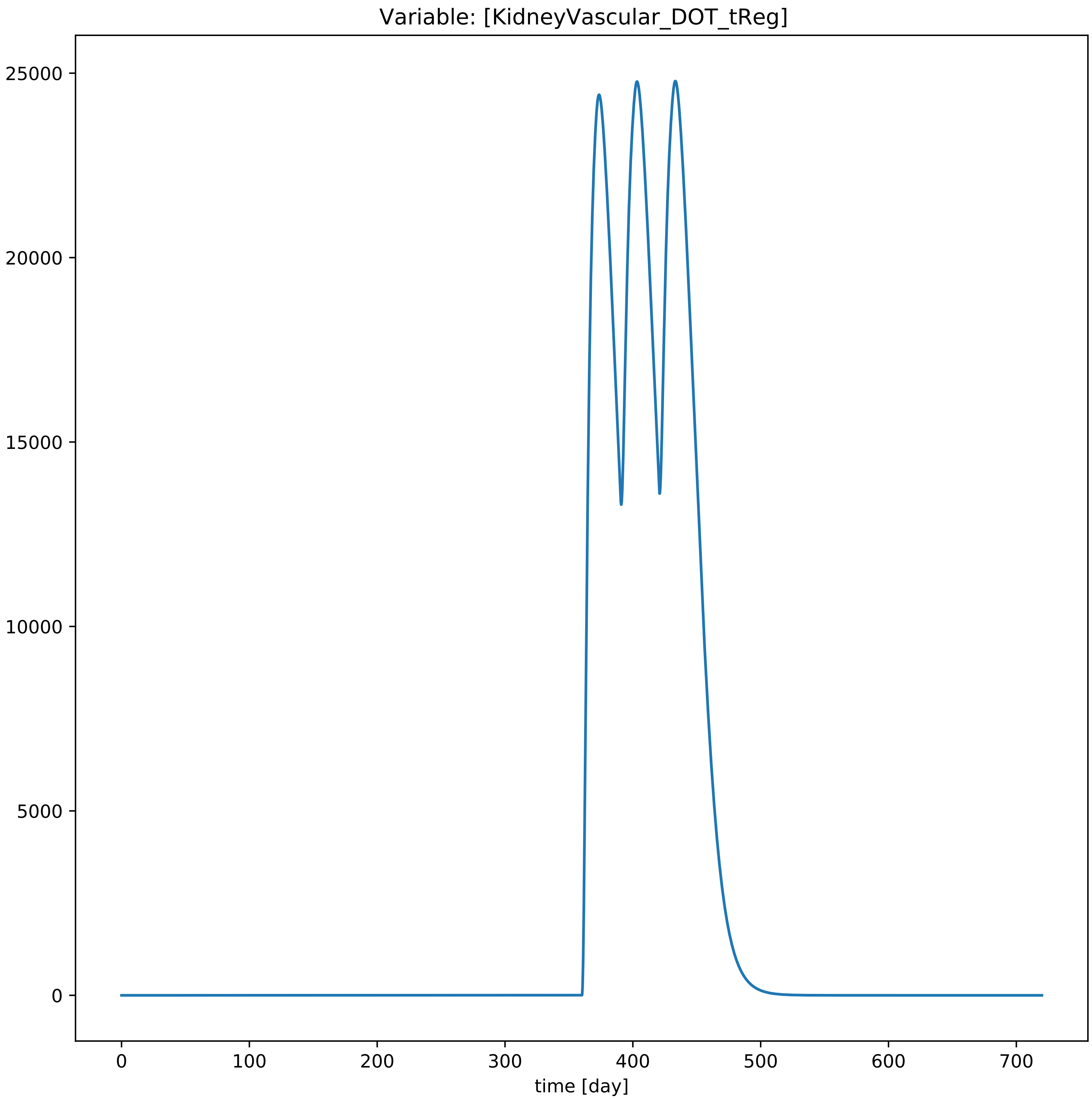

Variable: [KidneyVascular_DOT_tReg]
25000
20000
15000
10000
5000
0
0
100
200
300
400
500
600
700
time [day]

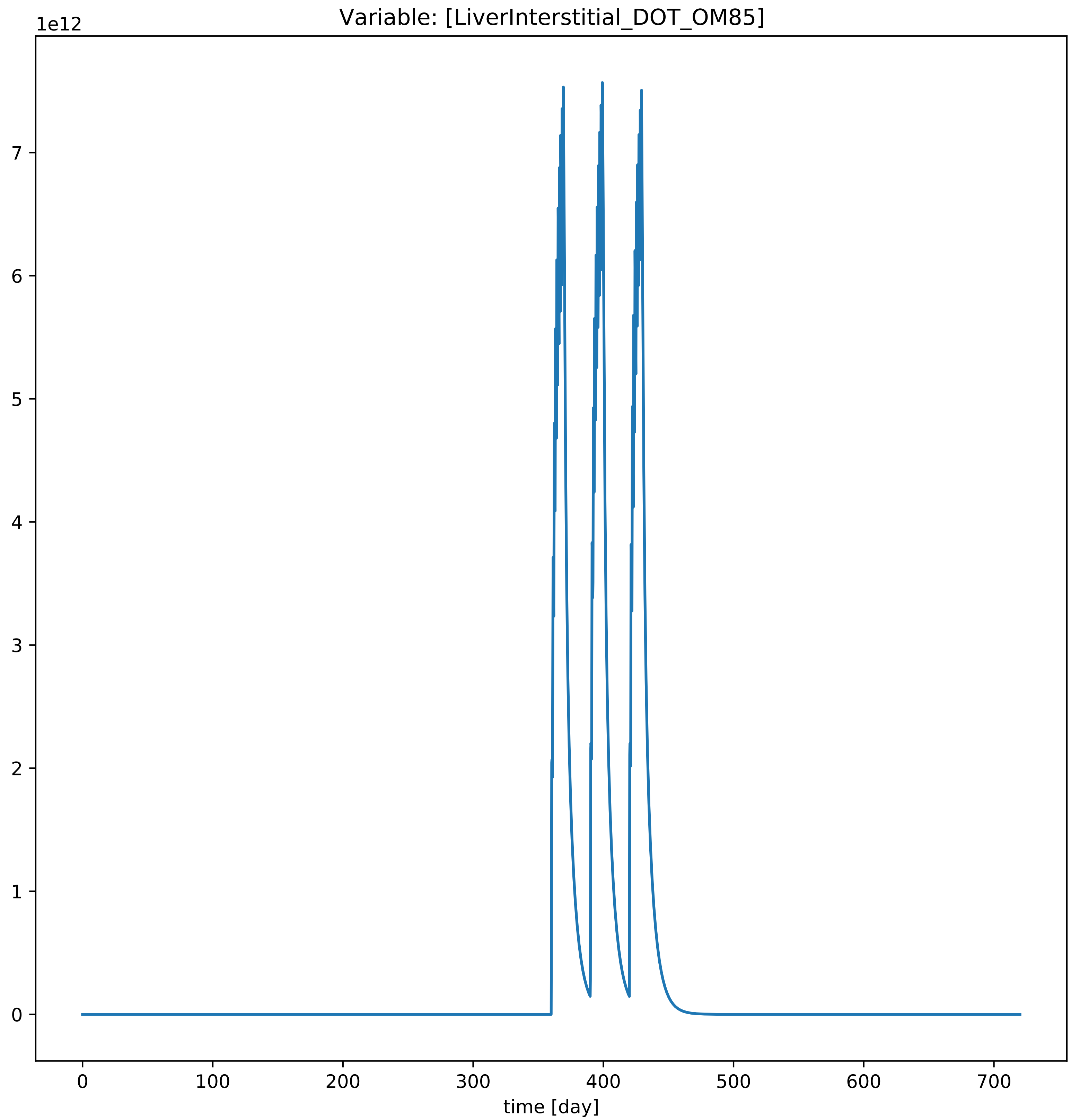

Variable: [LiverInterstitial_DOT_OM85]
1e12
0
1
2
3
4
5
6
7
0
100
200
300
400
500
600
700
time [day]

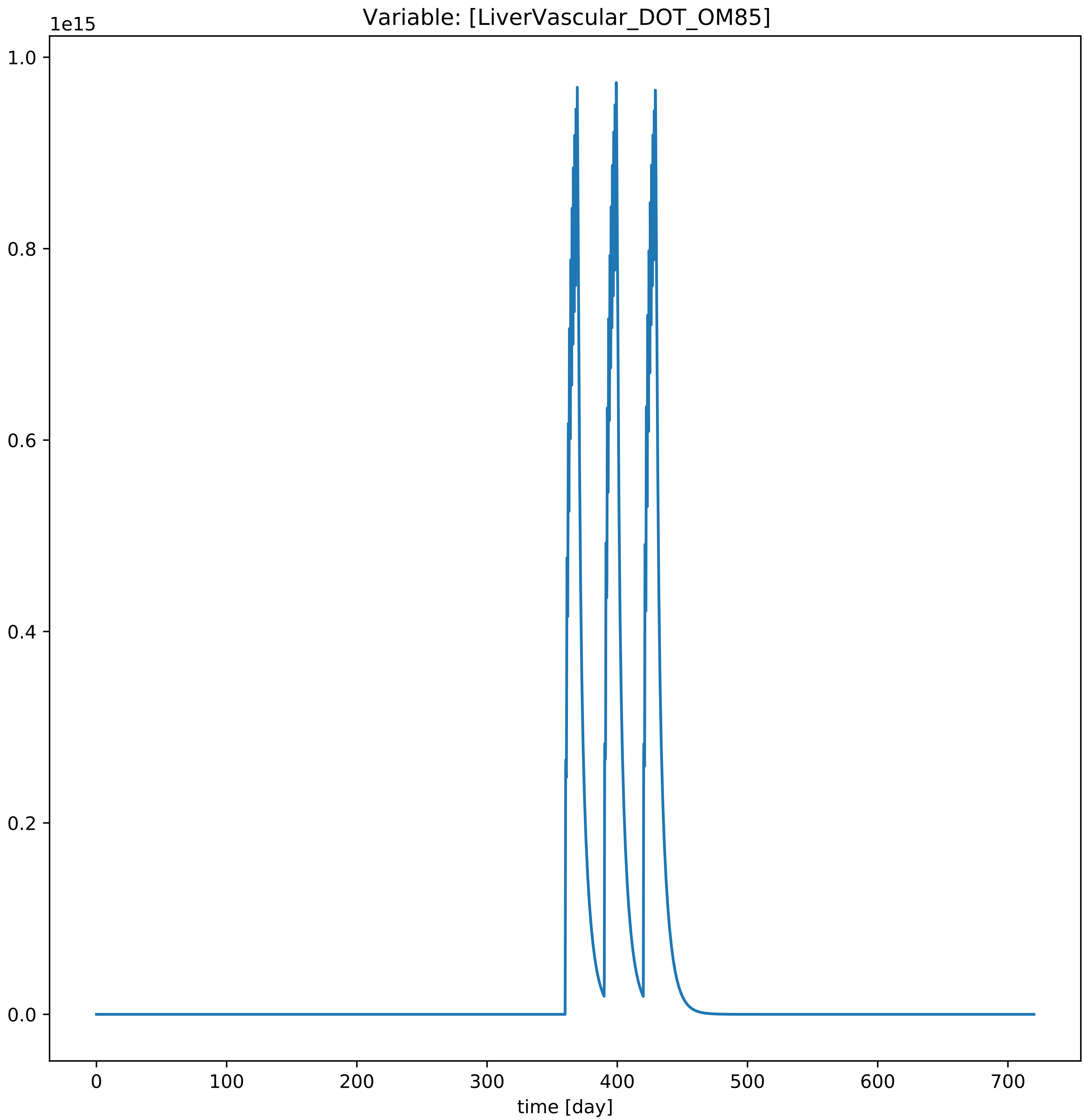

Variable: [LiverVascular_DOT_OM85]
1e15
1.0
0.8
0.6
0.4
0.2
0.0
0
100
200
300
400
500
600
700
time [day]

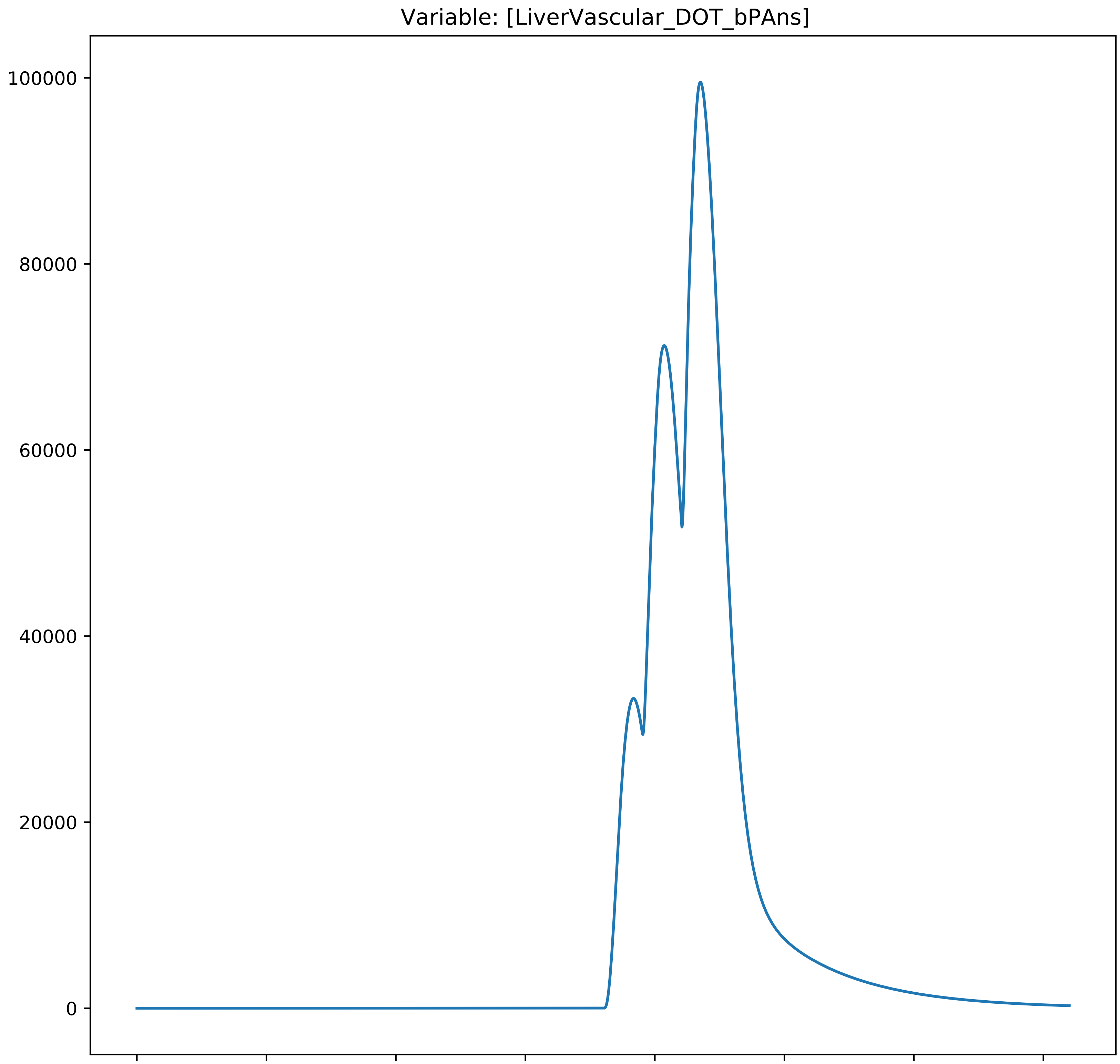

Variable: [LiverVascular_DOT_bPAns]
100000
80000
60000
40000
20000
0
0
100
200
300
400
500
600
700
time [day]

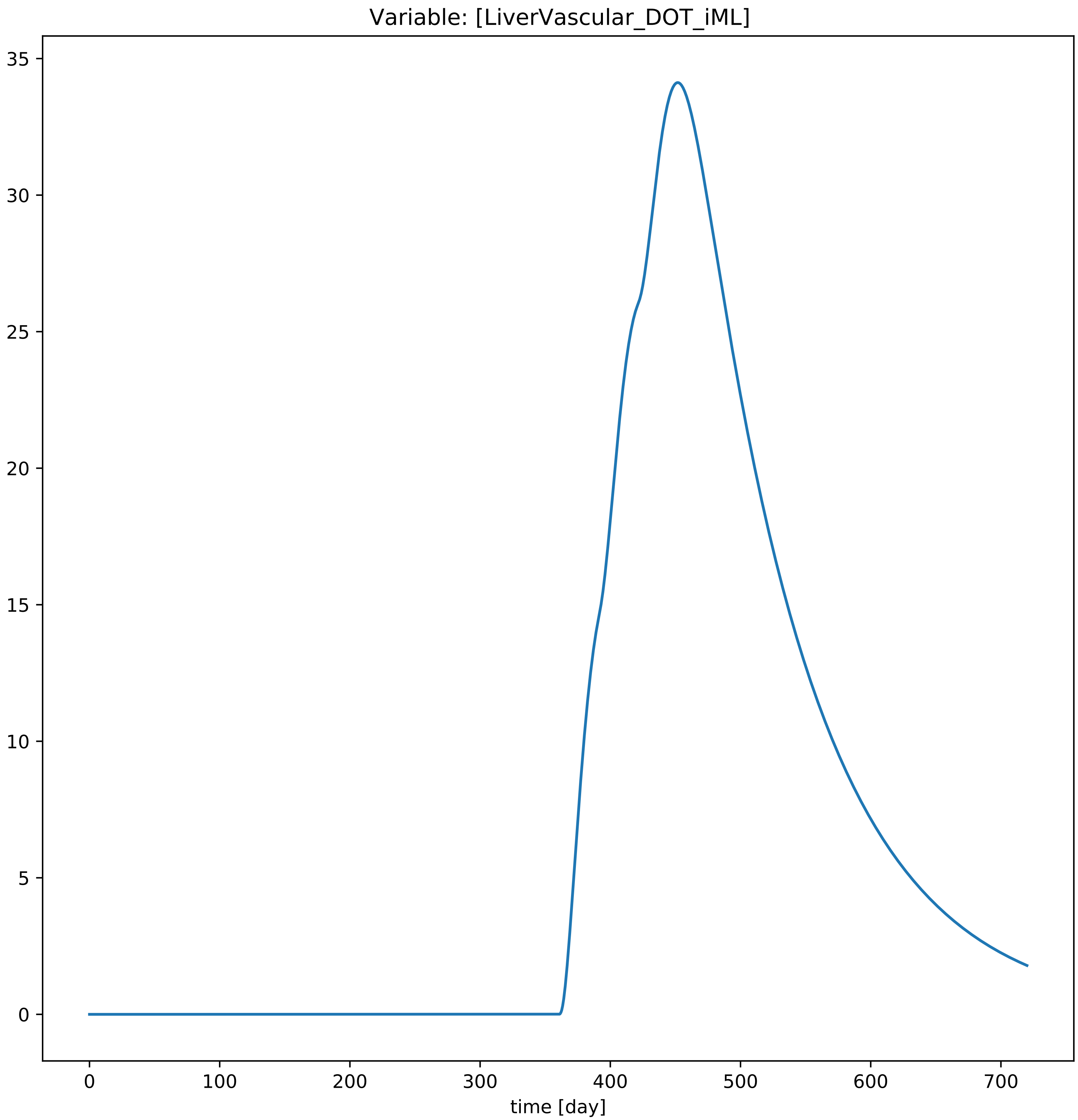

Variable: [LiverVascular_DOT_iML]
35
30
25
20
15
10
5
0
0
100
200
300
400
500
600
700
time [day]

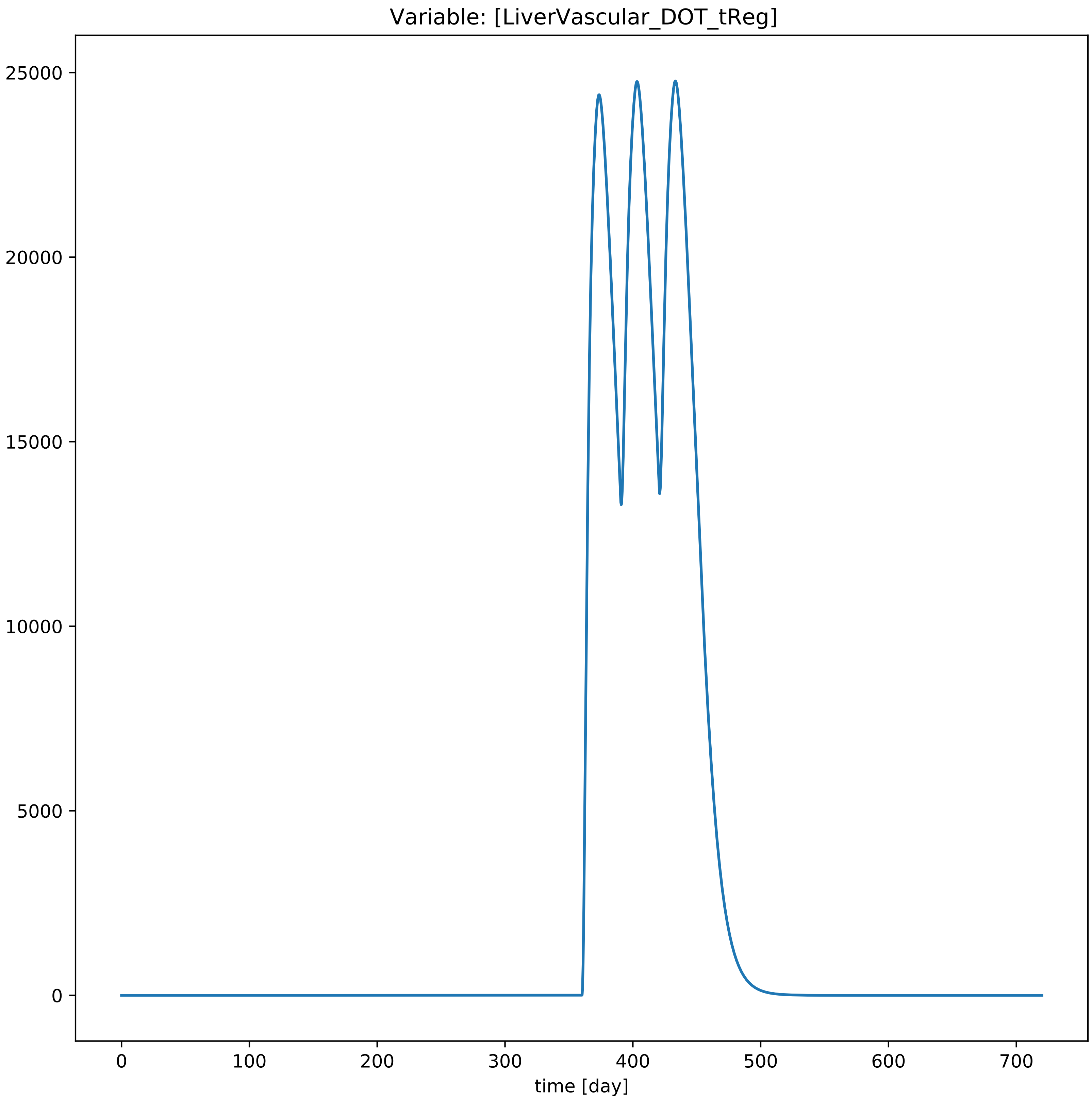
Variable: [LiverVascular_DOT_tReg]
25000
20000
15000
10000
5000
0
0
100
200
300
400
500
600
700
time [day]

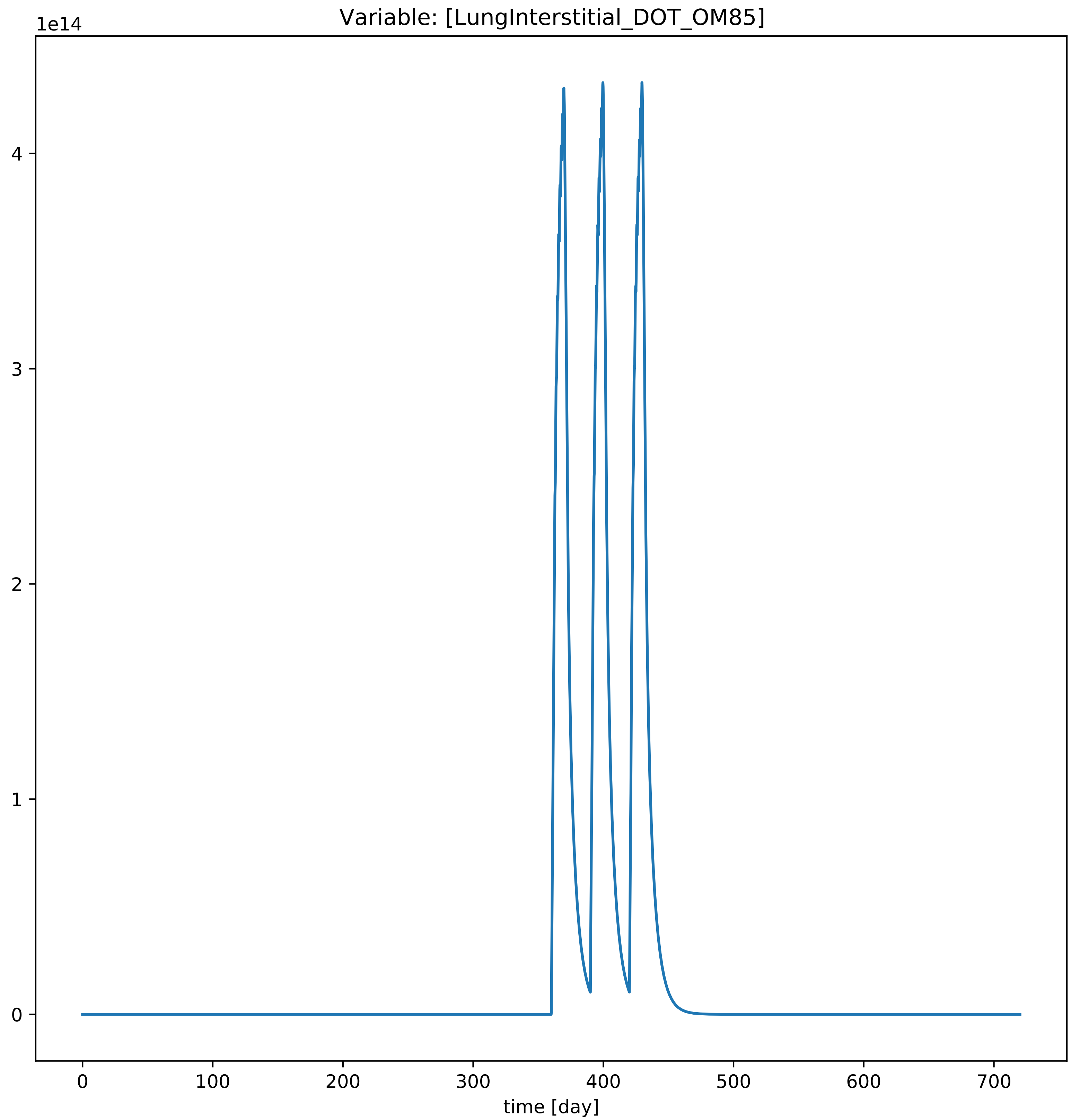

Variable: [LungInterstitial_DOT_OM85]
1e14
0
1
2
3
4
0
100
200
300
400
500
600
700
time [day]

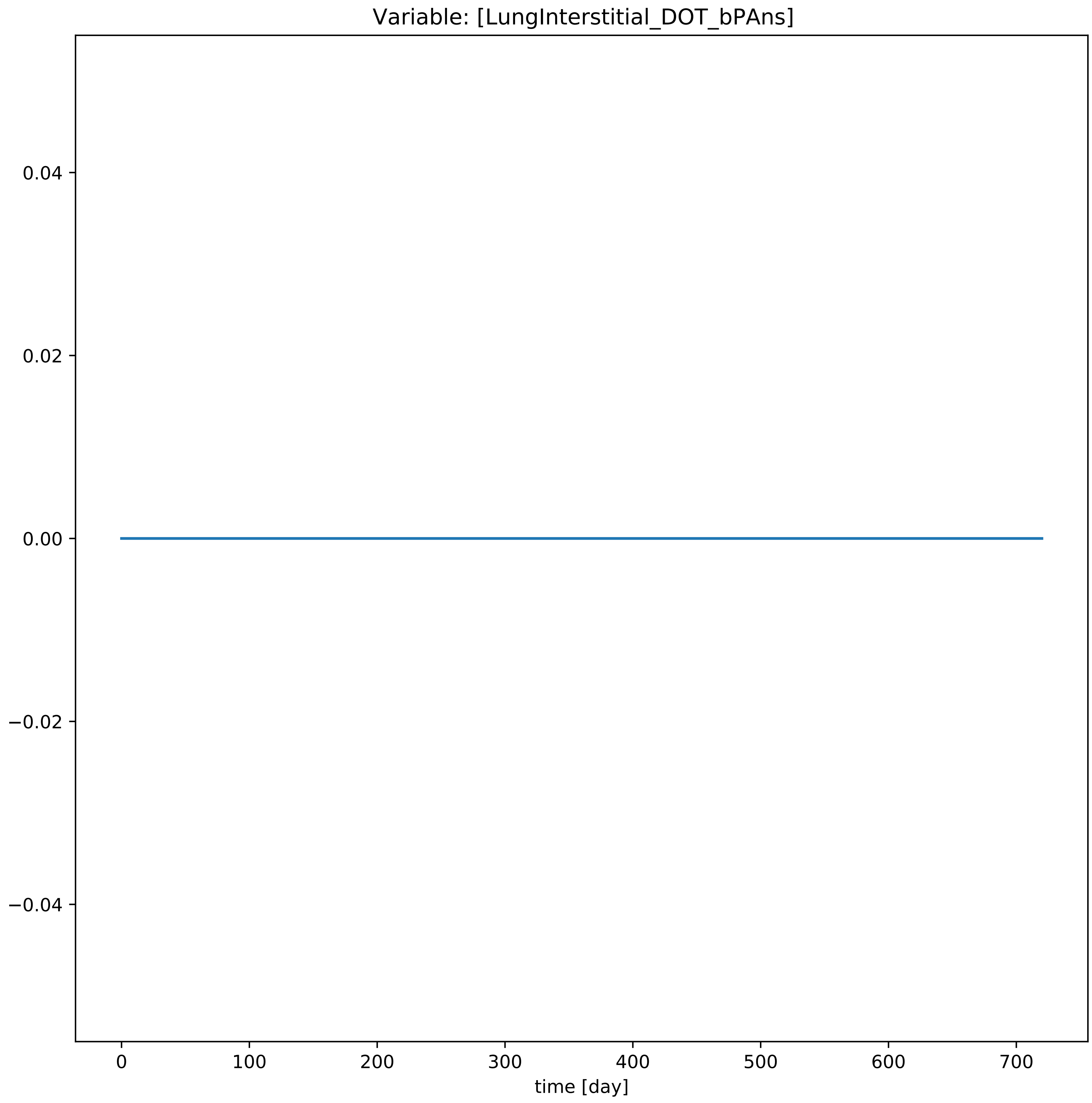
Variable: [LungInterstitial_DOT_bPAns]
0.04
0.02
0.00
−0.02
−0.04
0
100
200
300
400
500
600
700
time [day]

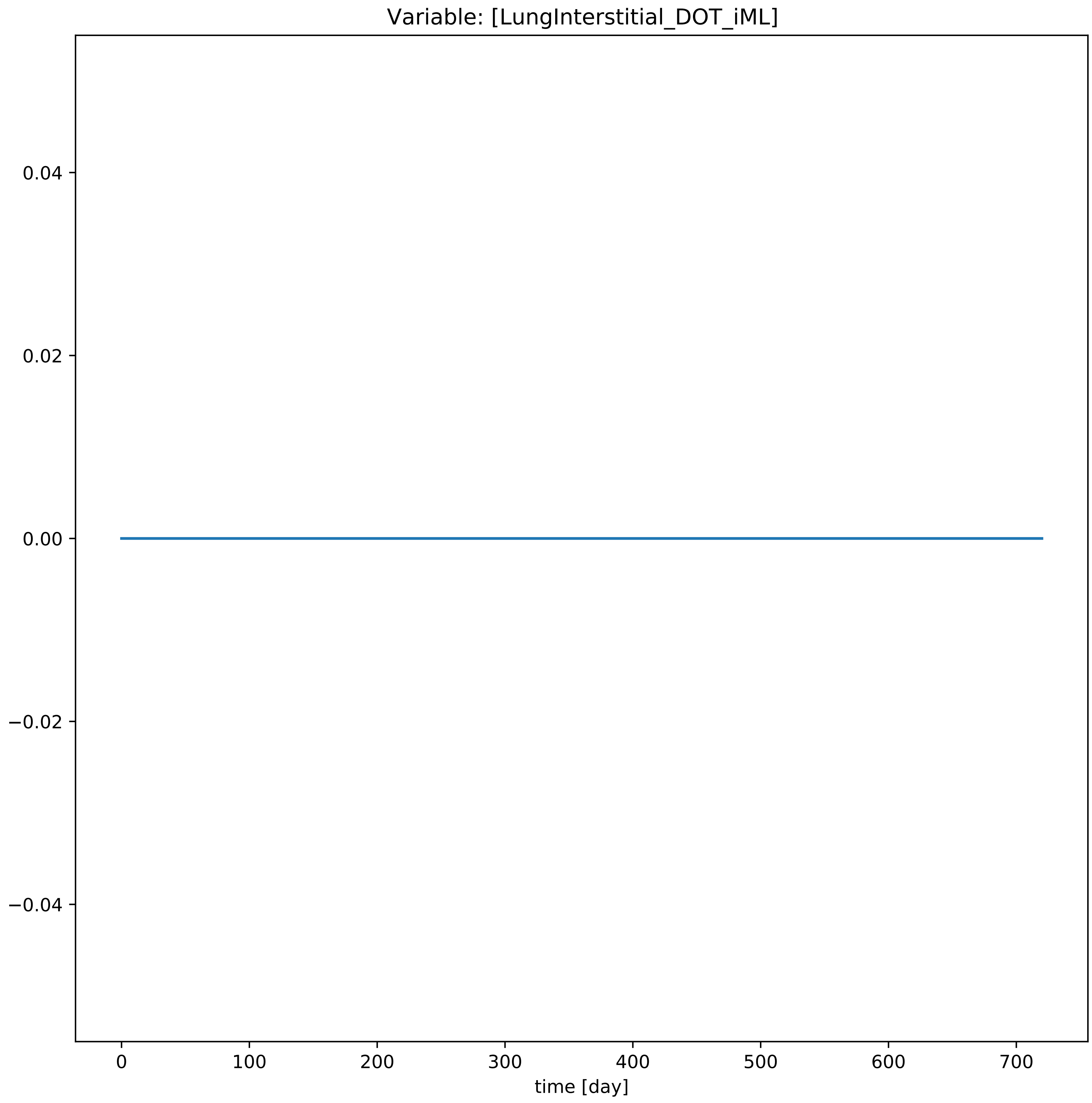
Variable: [LungInterstitial_DOT_iML]
0.04
0.02
0.00
−0.02
−0.04
0
100
200
300
400
500
600
700
time [day]

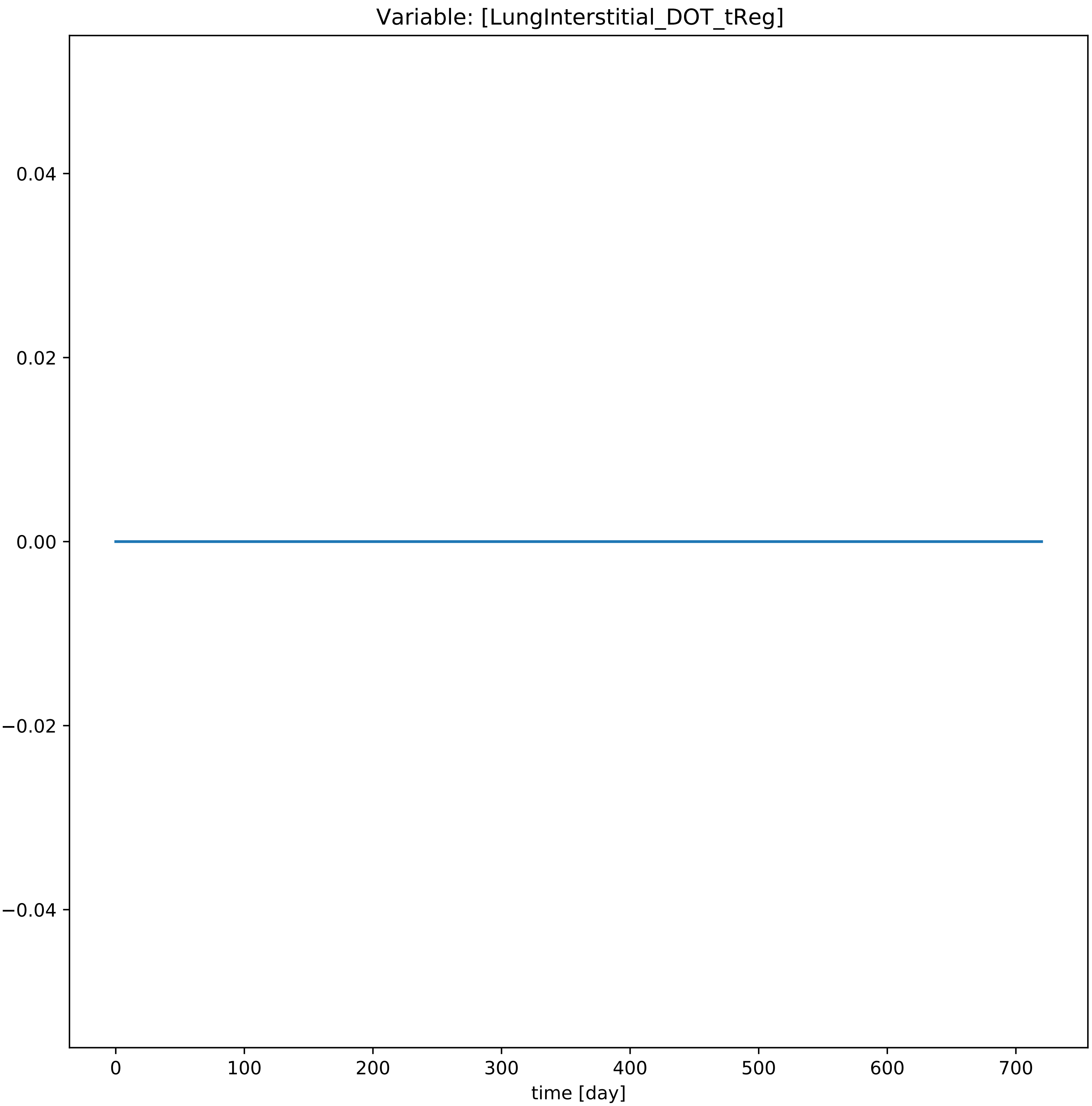

Variable: [LungInterstitial_DOT_tReg]
0.04
0.02
0.00
−0.02
−0.04
0
100
200
300
400
500
600
700
time [day]

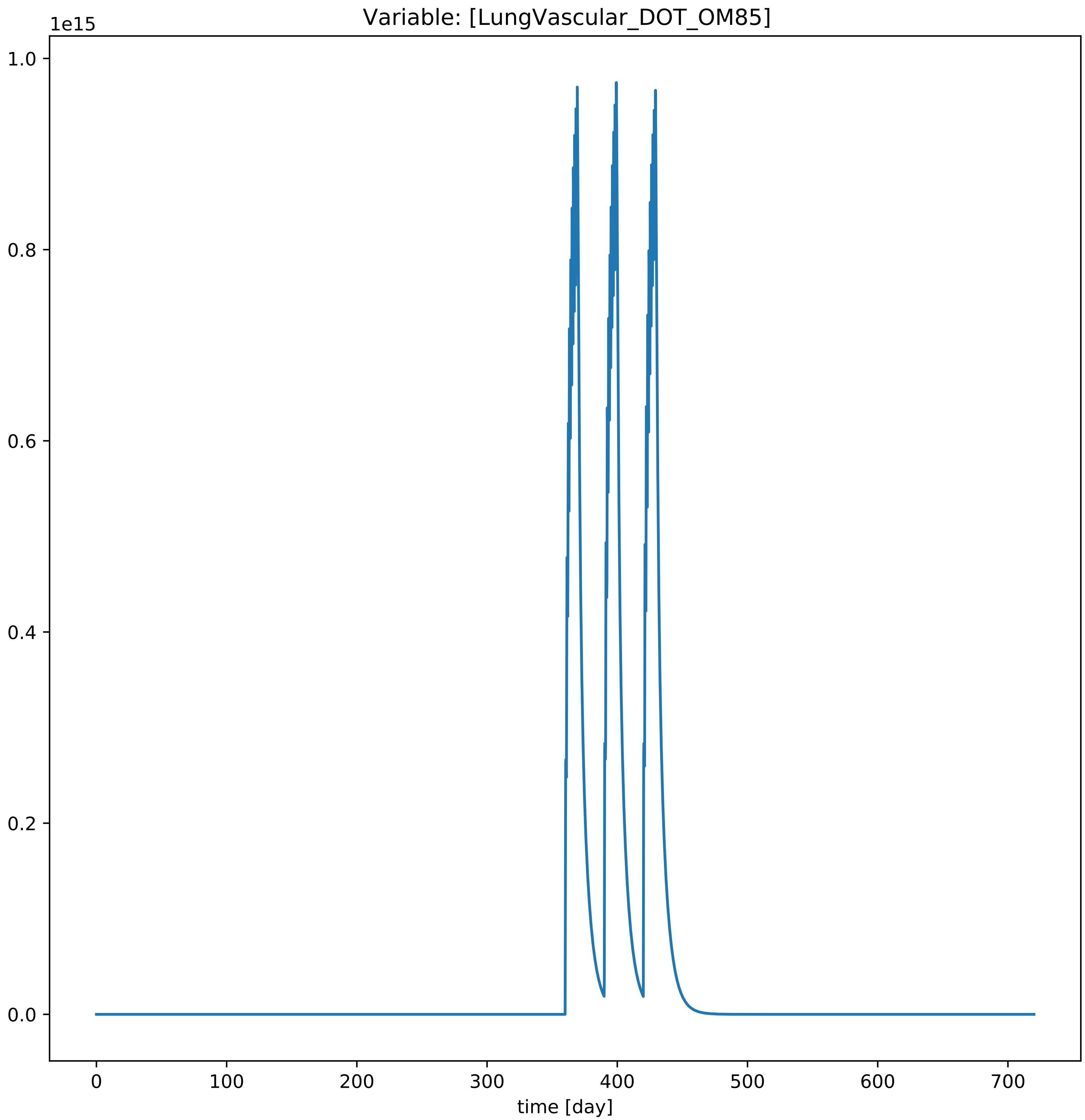

Variable: [LungVascular_DOT_OM85]
1e15
1.0
0.8
0.6
0.4
0.2
0.0
0
100
200
300
400
500
600
700
time [day]

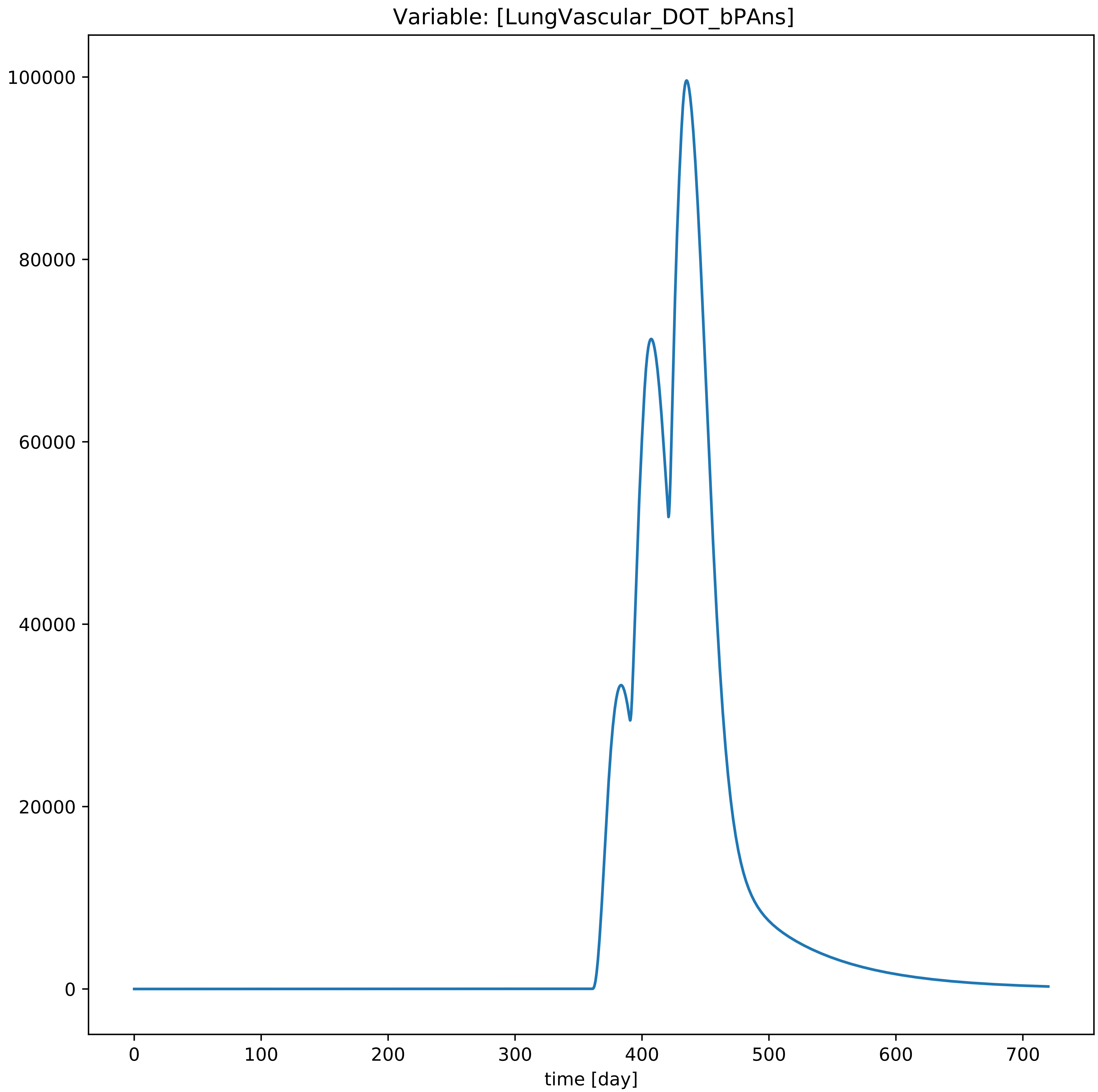

Variable: [LungVascular_DOT_bPAns]
100000
80000
60000
40000
20000
0
0
100
200
300
400
500
600
700
time [day]

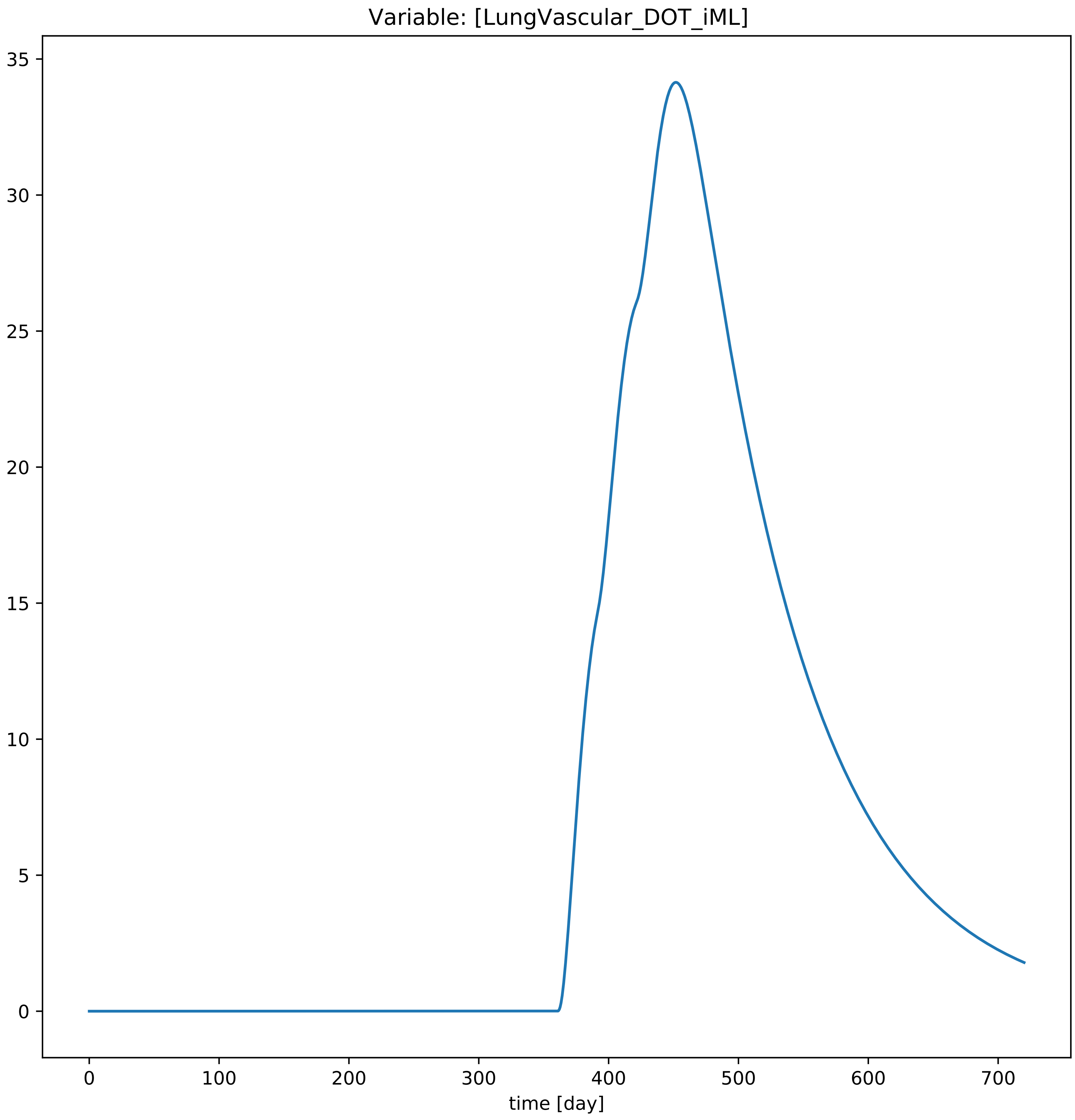
Variable: [LungVascular_DOT_iML]
35
30
25
20
15
10
5
0
0
100
200
300
400
500
600
700
time [day]

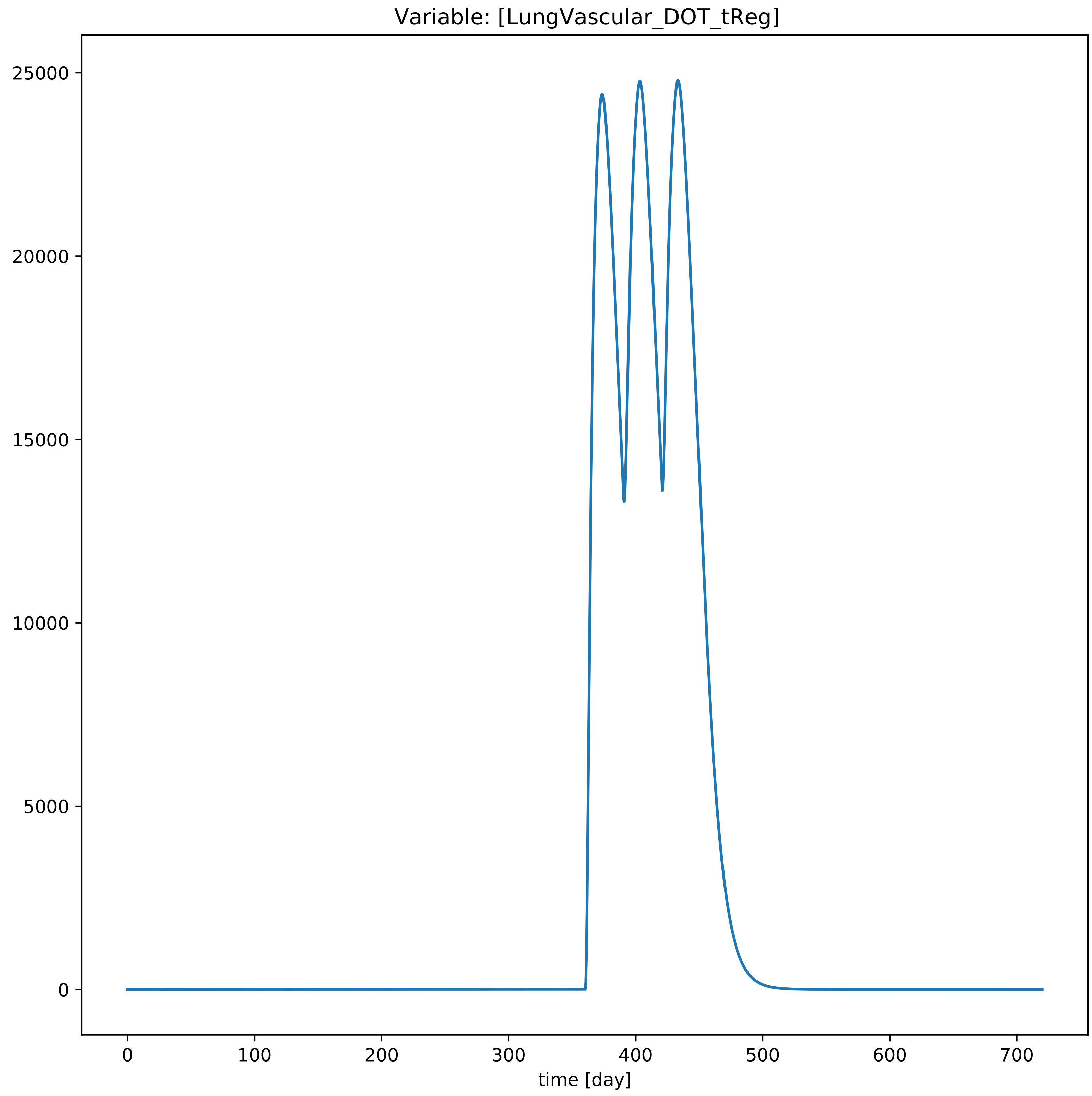

Variable: [LungVascular_DOT_tReg]
25000
20000
15000
10000
5000
0
0
100
200
300
400
500
600
700
time [day]

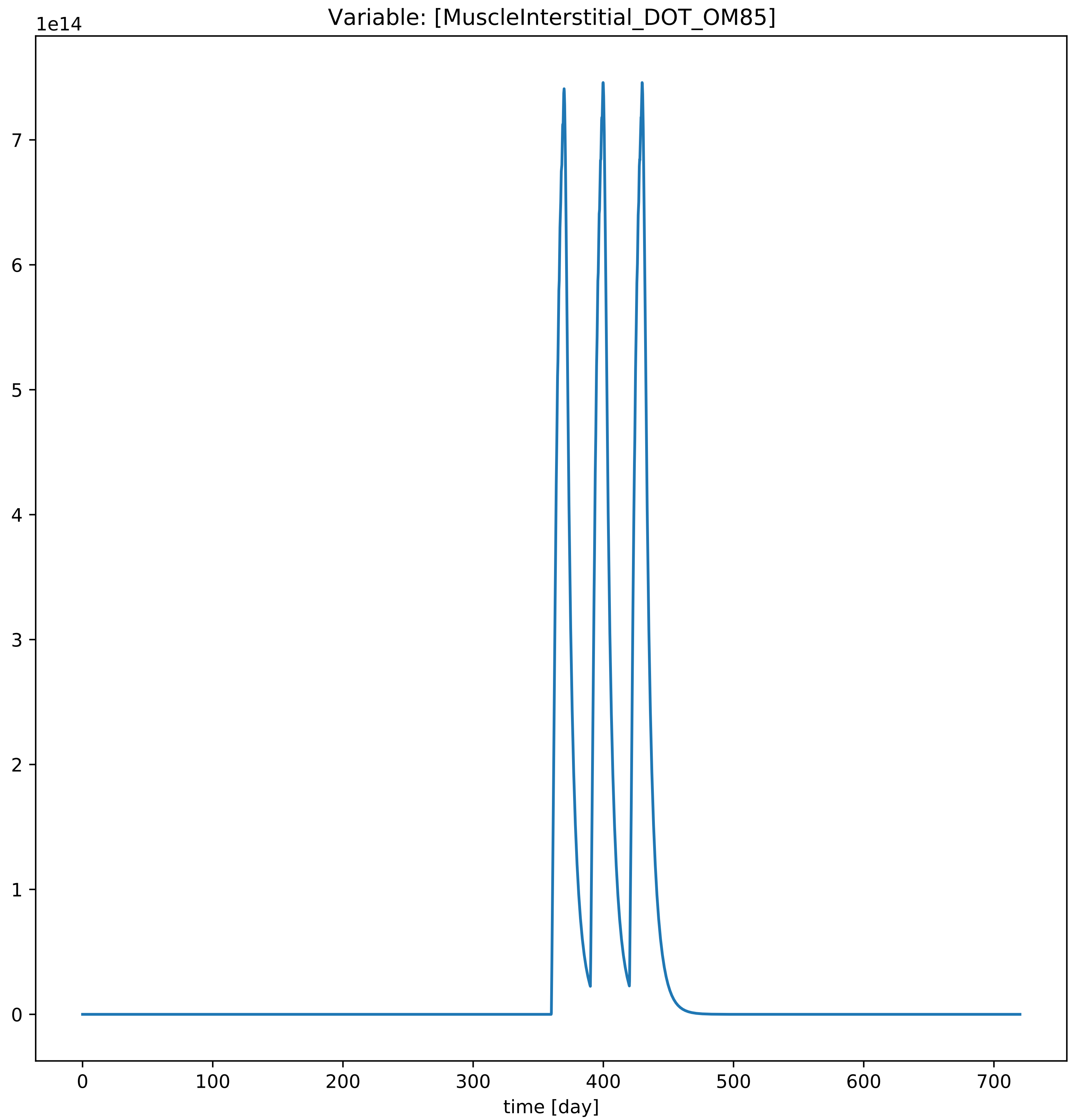

Variable: [MuscleInterstitial_DOT_OM85]
1e14
7
6
5
4
3
2
1
0
0
100
200
300
400
500
600
700
time [day]

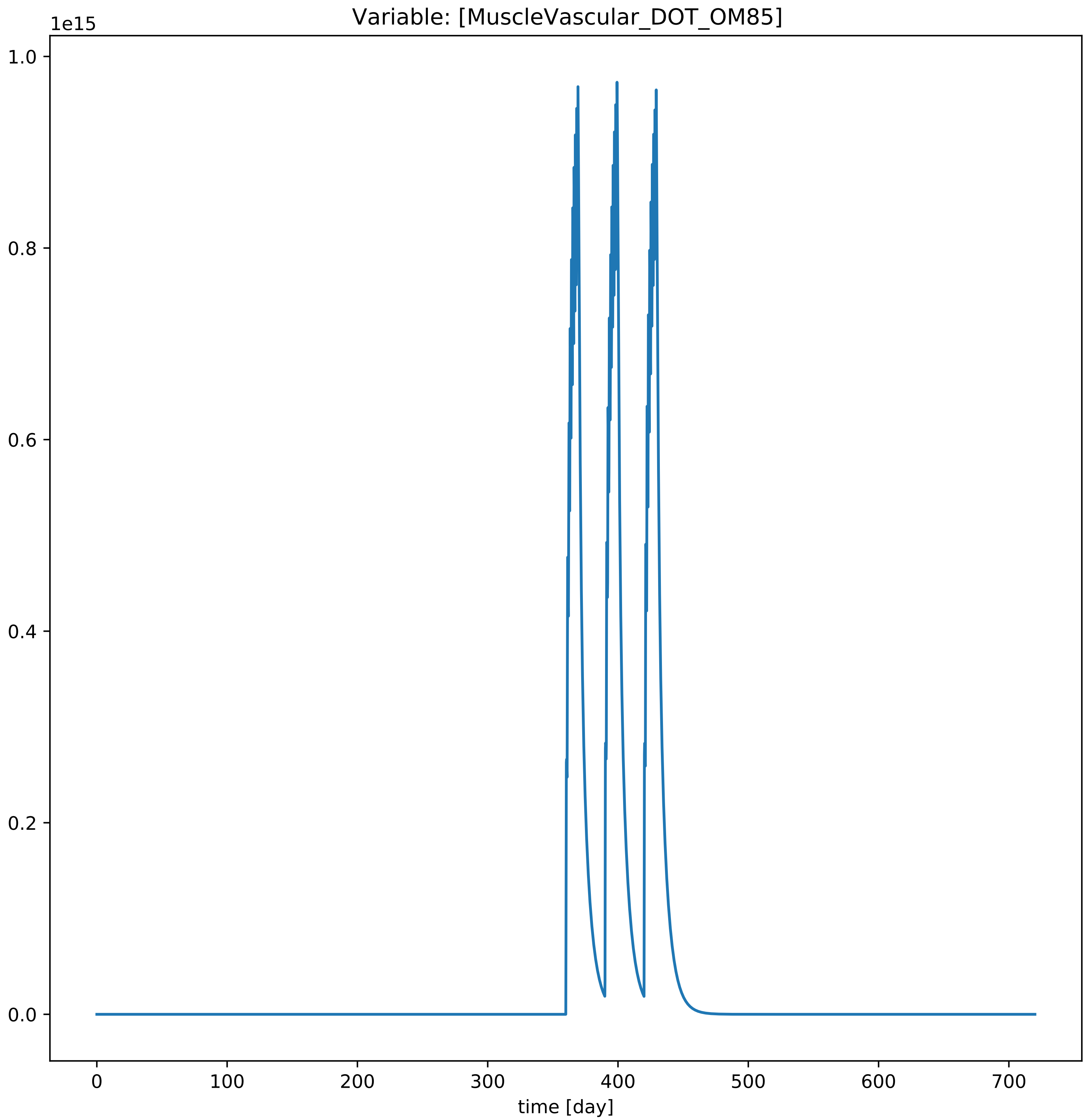

Variable: [MuscleVascular_DOT_OM85]
1e15
1.0
0.8
0.6
0.4
0.2
0.0
0
100
200
300
400
500
600
700
time [day]

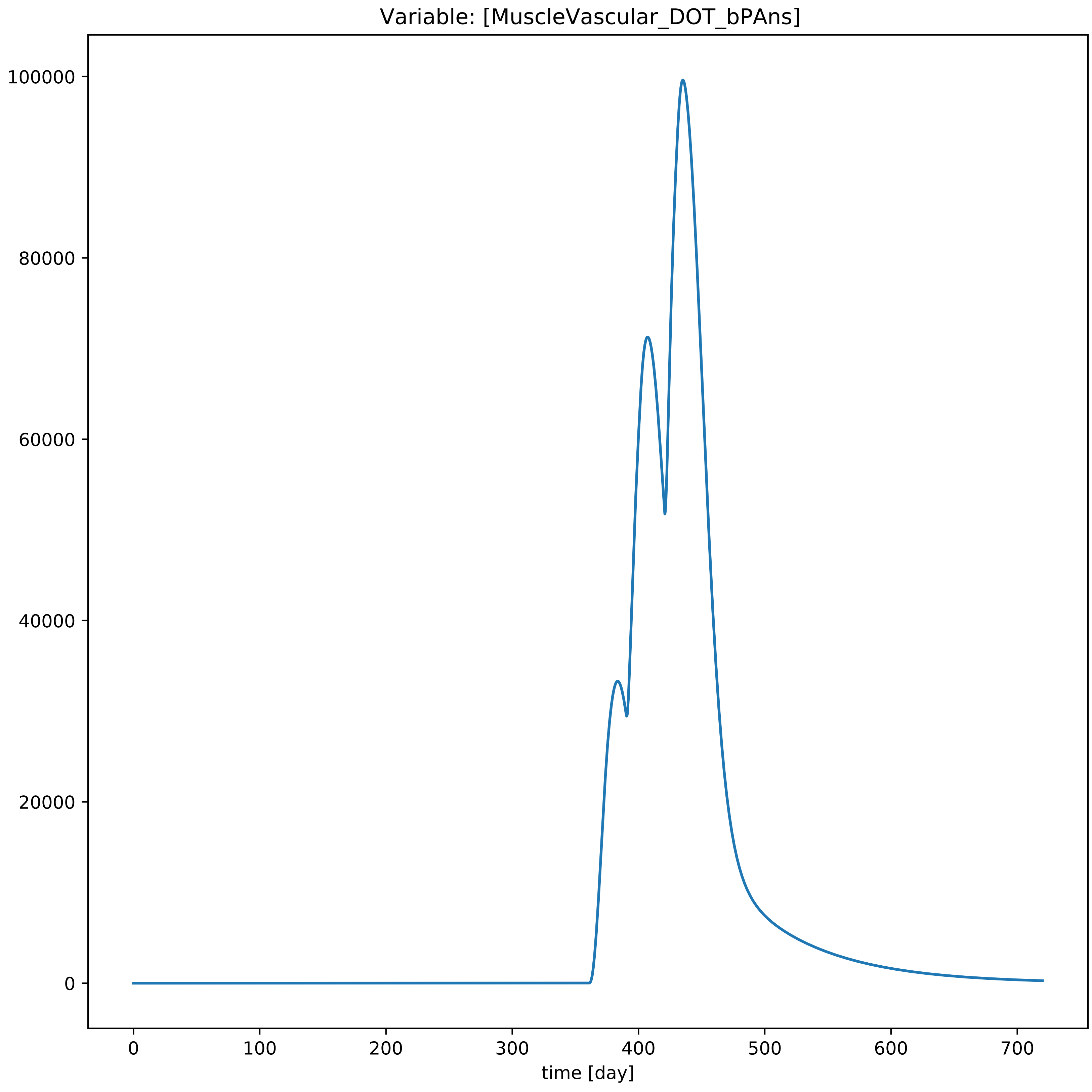
Variable: [MuscleVascular_DOT_bPAns]
100000
80000
60000
40000
20000
0
0
100
200
300
400
500
600
700
time [day]

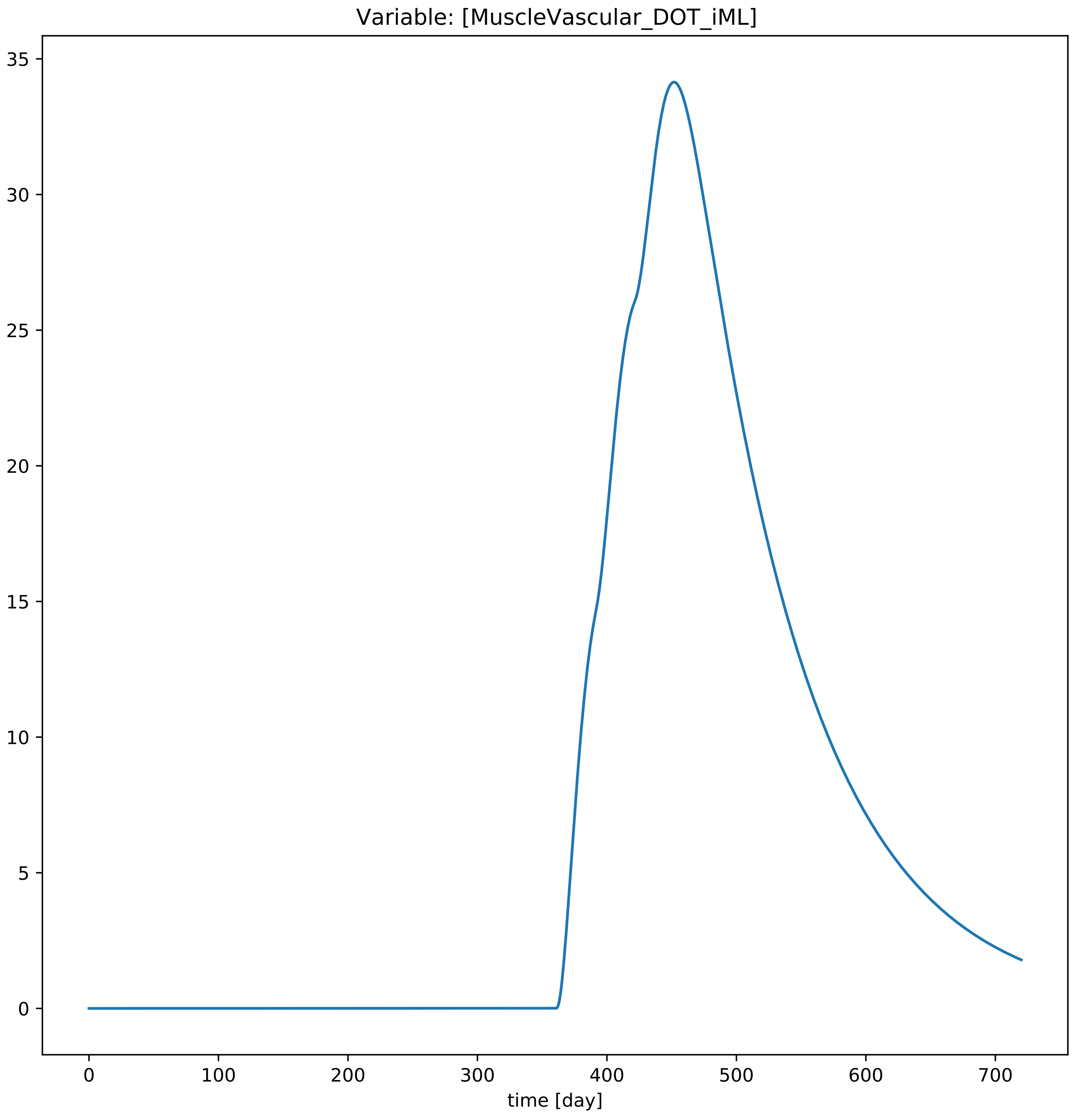

Variable: [MuscleVascular_DOT_iML]
35
30
25
20
15
10
5
0
0
100
200
300
400
500
600
700
time [day]

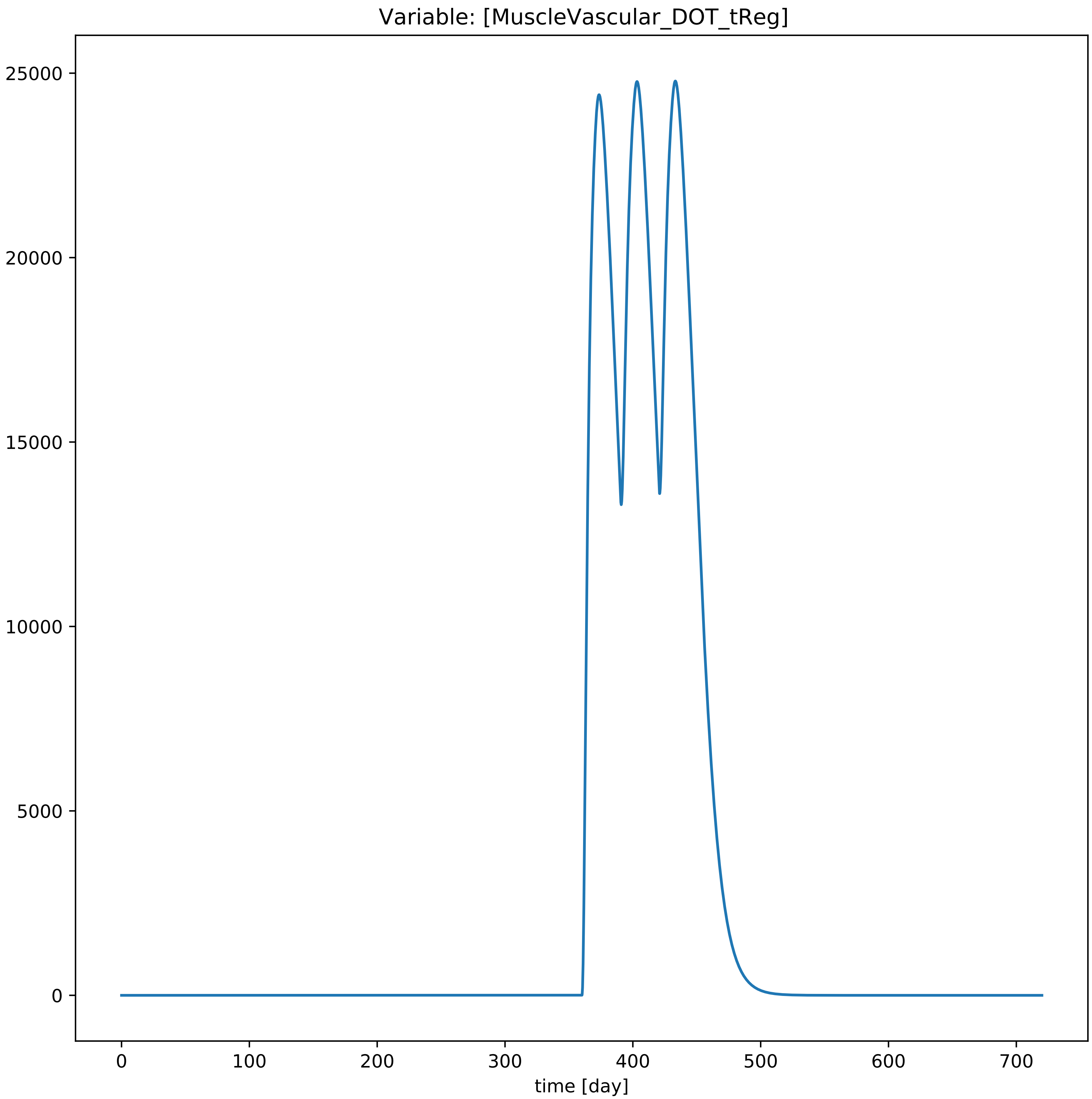
Variable: [MuscleVascular_DOT_tReg]
25000
20000
15000
10000
5000
0
0
100
200
300
400
500
600
700
time [day]

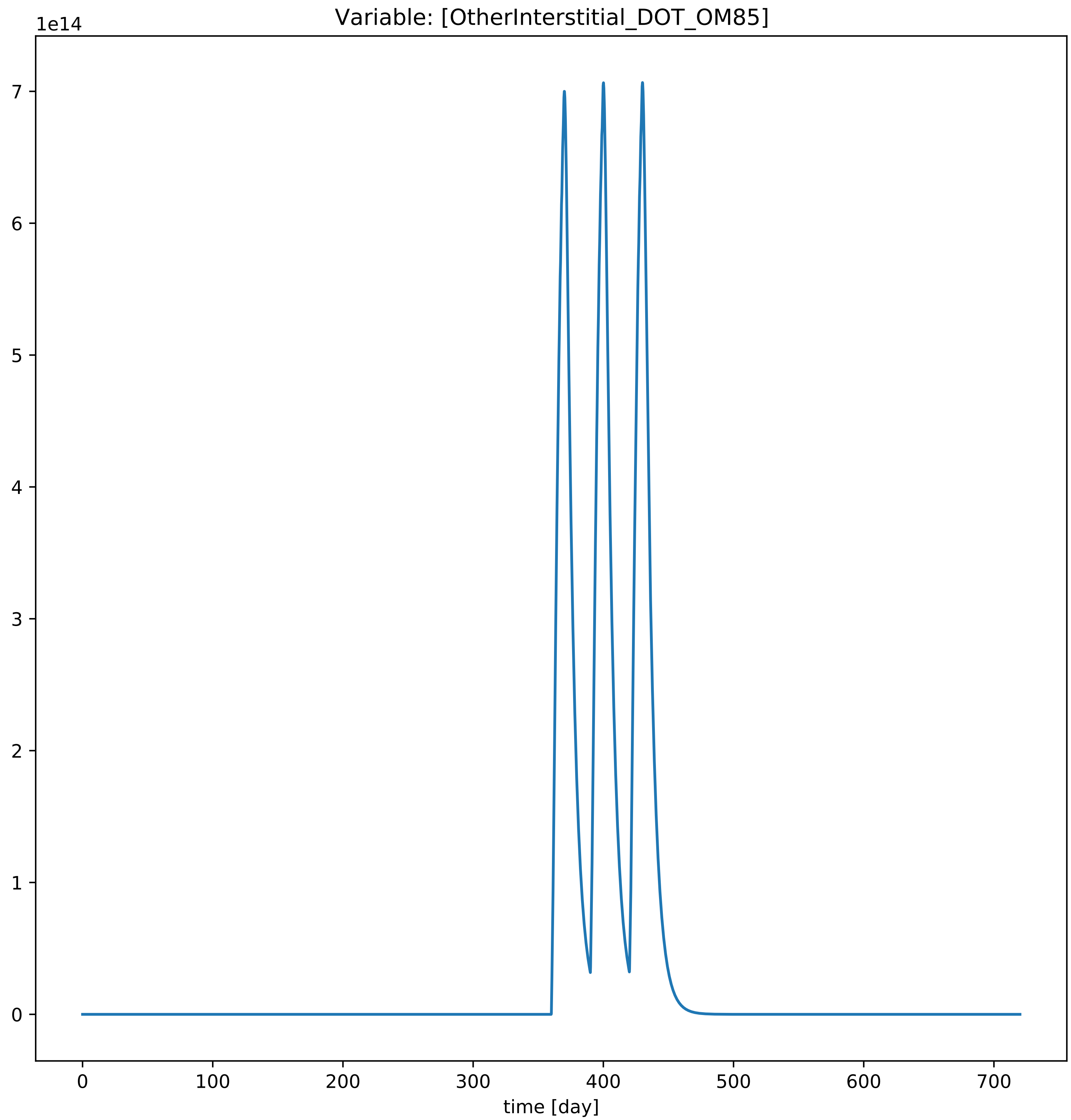

Variable: [OtherInterstitial_DOT_OM85]
1e14
7
6
5
4
3
2
1
0
0
100
200
300
400
500
600
700
time [day]

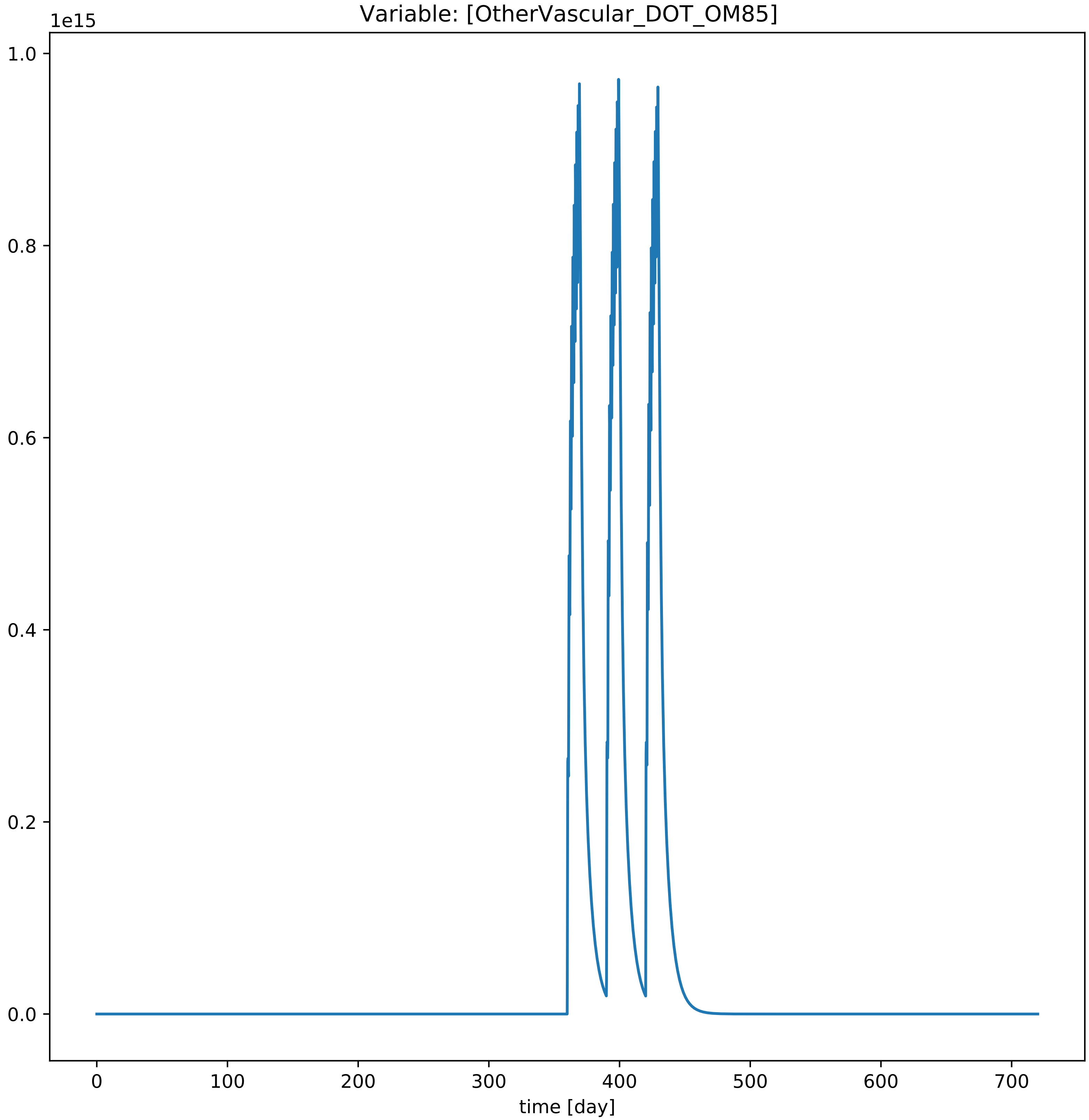

Variable: [OtherVascular_DOT_OM85]
1e15
1.0
0.8
0.6
0.4
0.2
0.0
0
100
200
300
400
500
600
700
time [day]

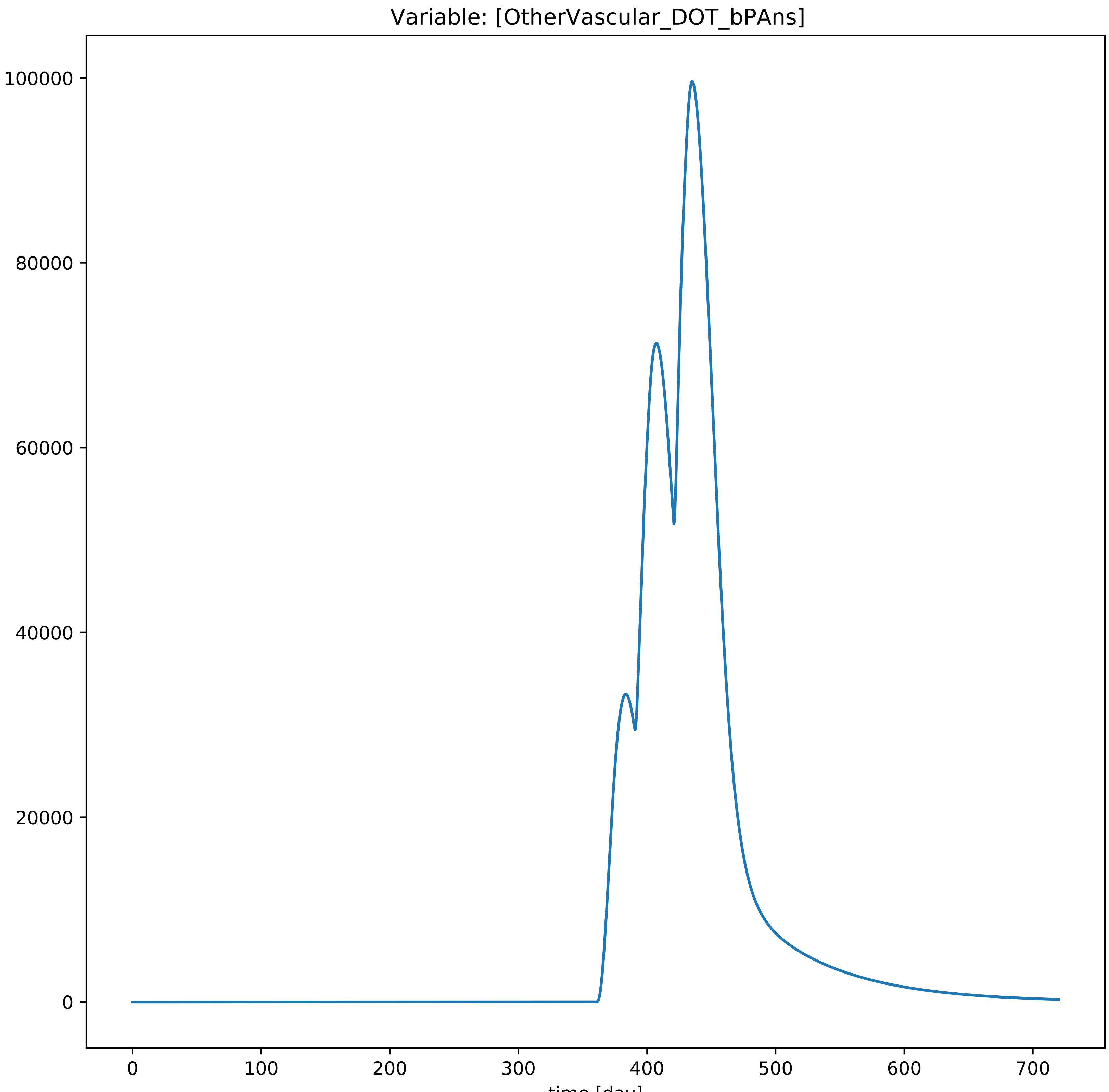

Variable: [OtherVascular_DOT_bPAns]
100000
80000
60000
40000
20000
0
0
100
200
300
400
500
600
700
time [day]

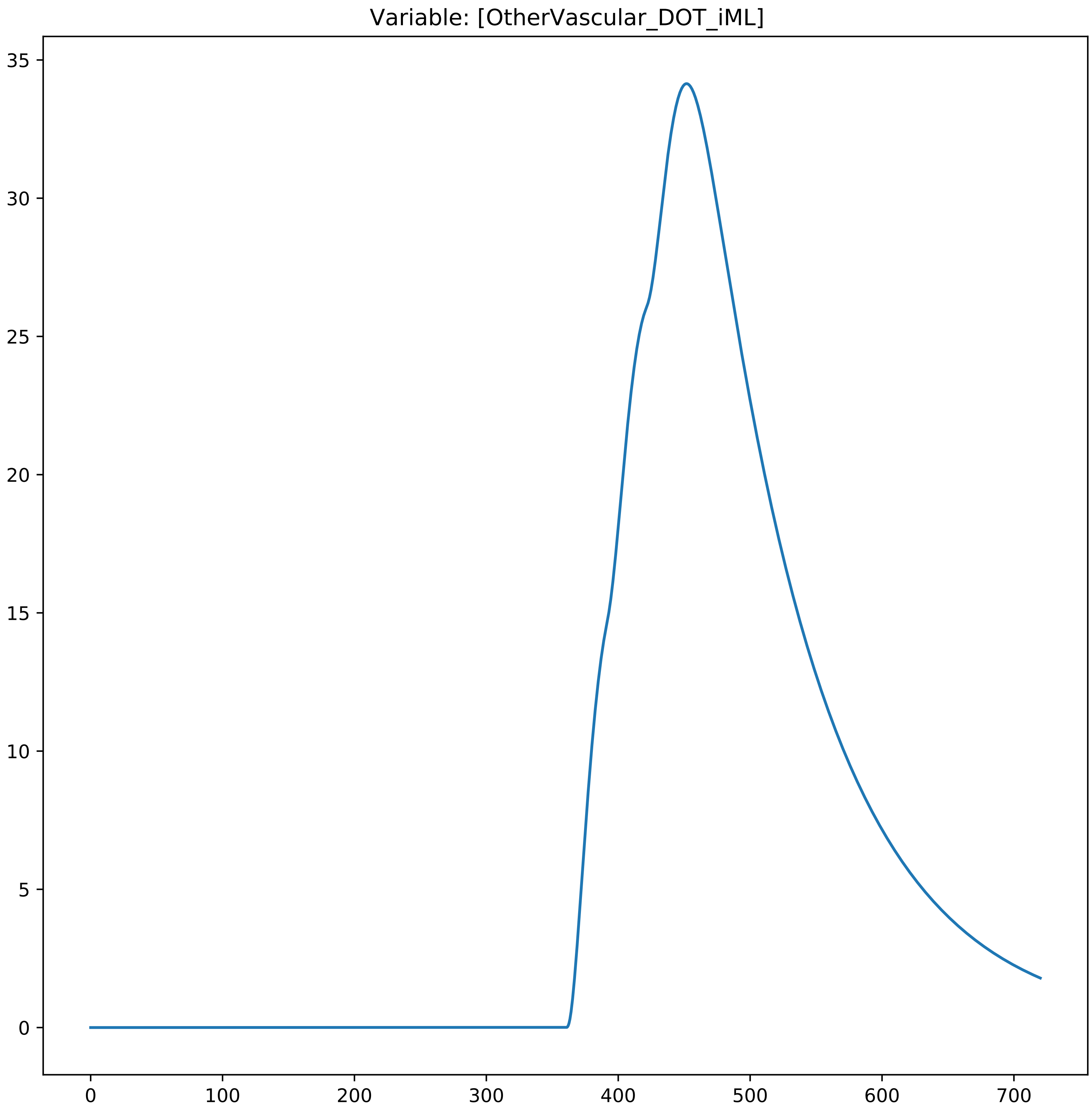

Variable: [OtherVascular_DOT_iML]
35
30
25
20
15
10
5
0
0
100
200
300
400
500
600
700
time [day]

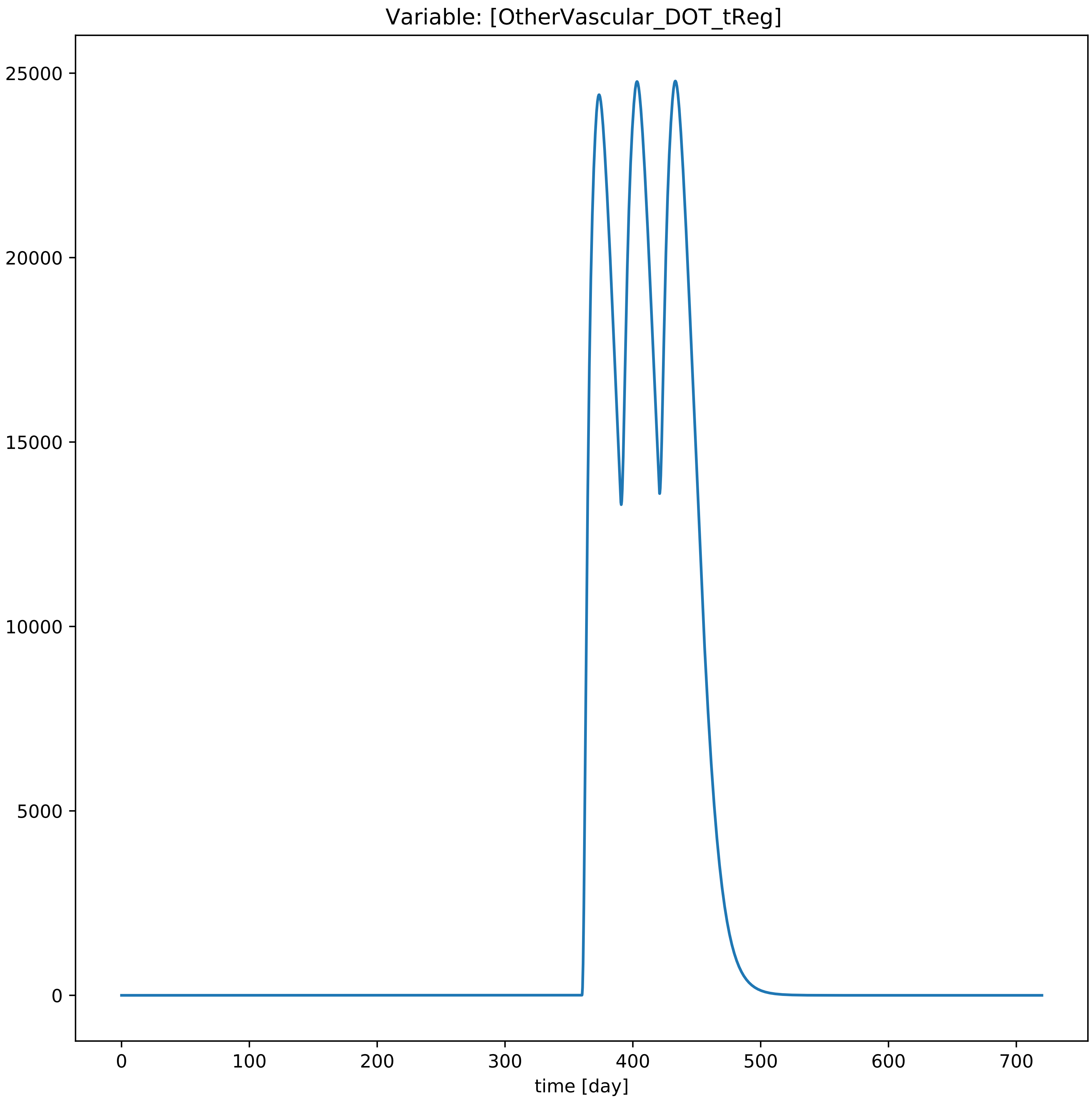

Variable: [OtherVascular_DOT_tReg]
25000
20000
15000
10000
5000
0
0
100
200
300
400
500
600
700
time [day]

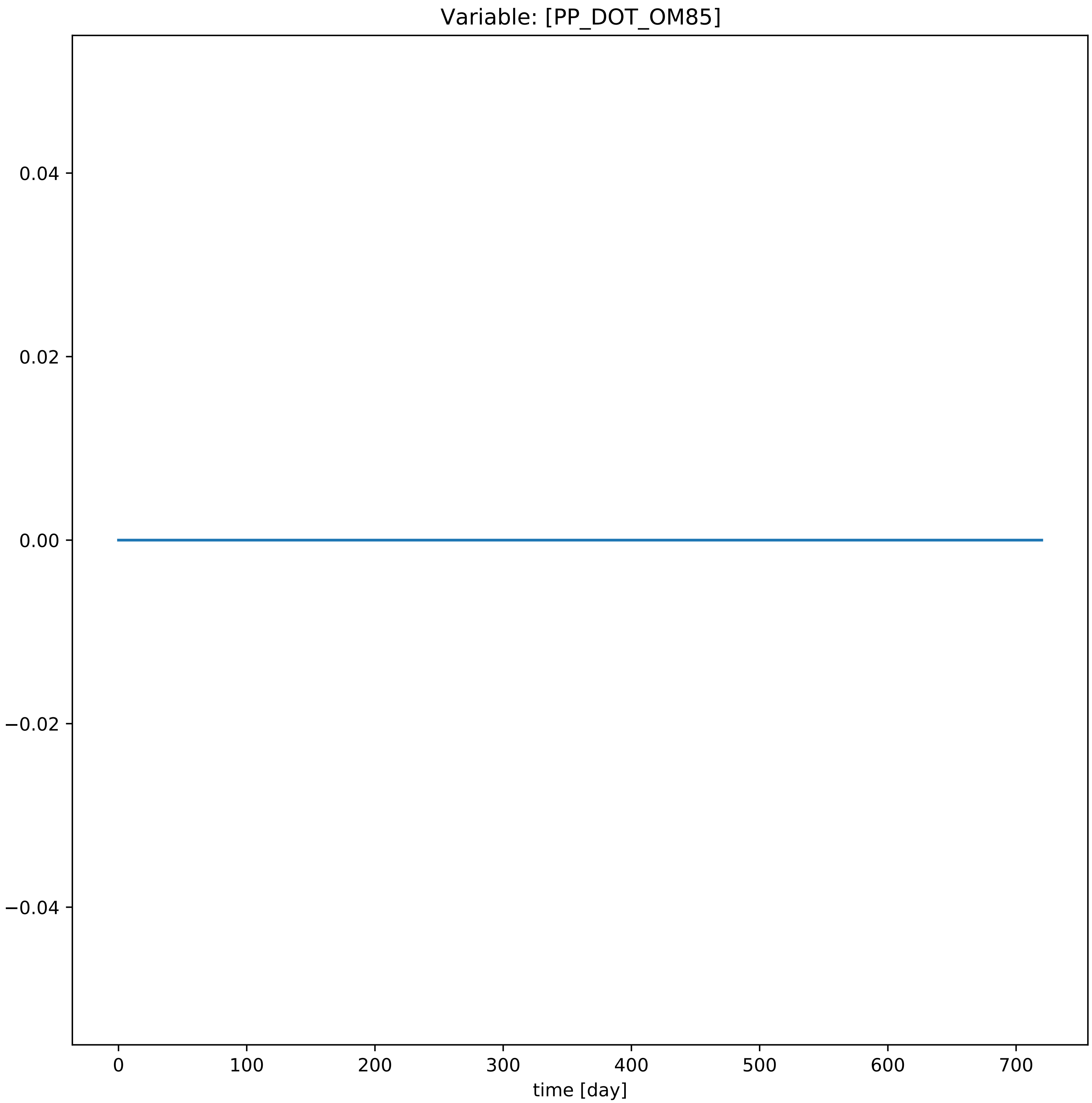
Variable: [PP_DOT_OM85]
0.04
0.02
0.00
−0.02
−0.04
0
100
200
300
400
500
600
700
time [day]

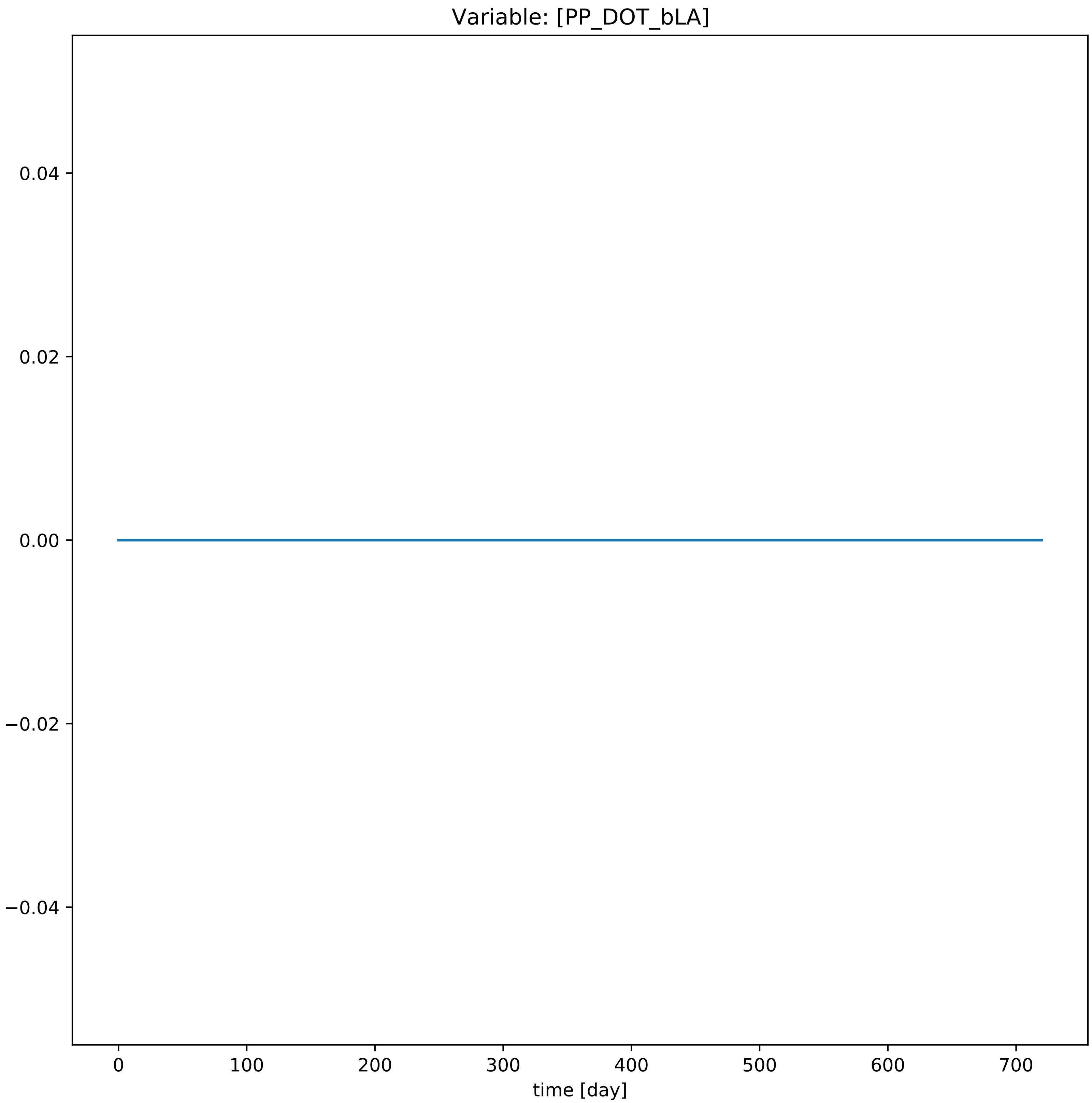

Variable: [PP_DOT_bLA]
0.04
0.02
0.00
−0.02
−0.04
0
100
200
300
400
500
600
700
time [day]

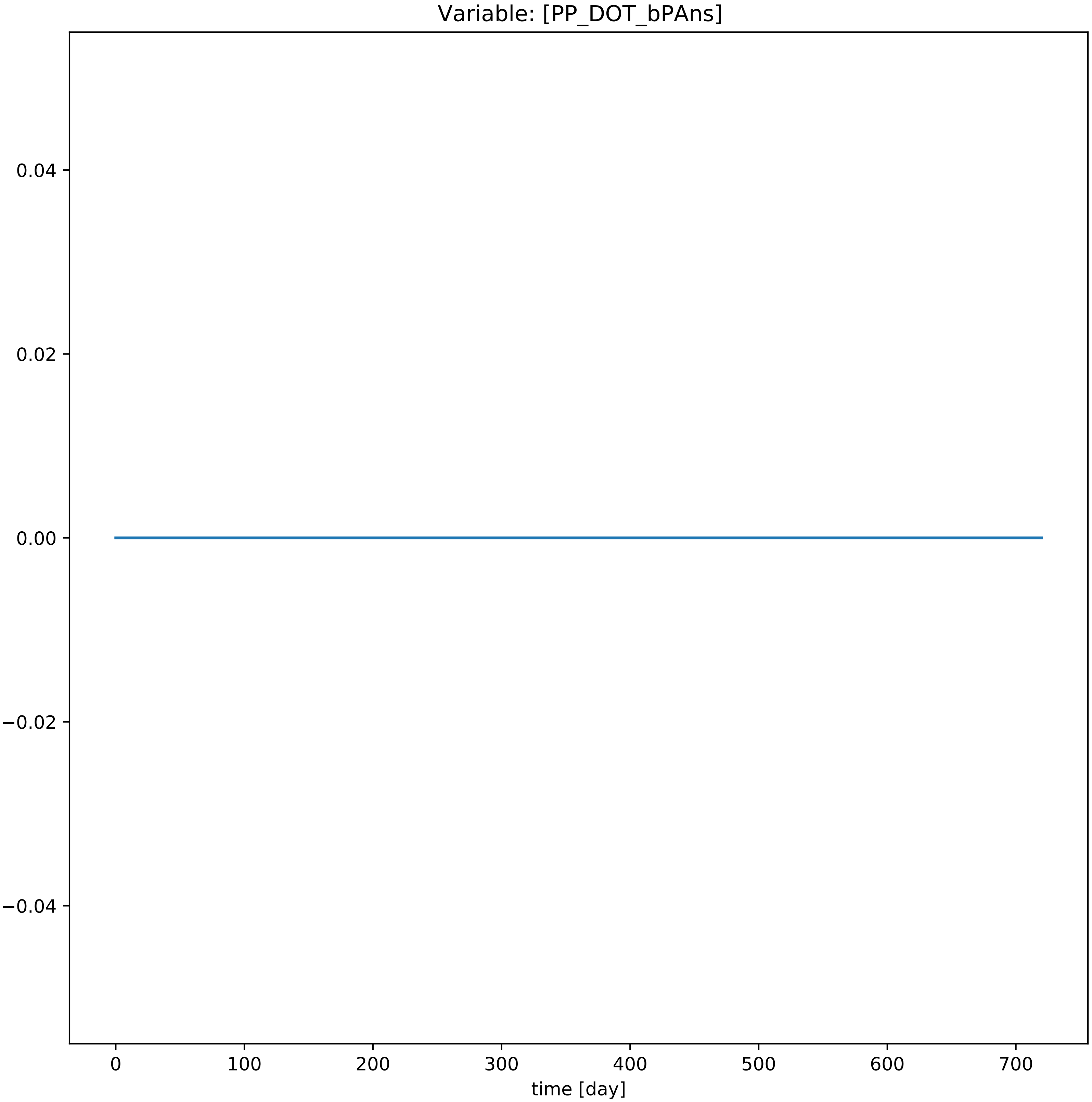
Variable: [PP_DOT_bPAns]
0.04
0.02
0.00
−0.02
−0.04
0
100
200
300
400
500
600
700
time [day]

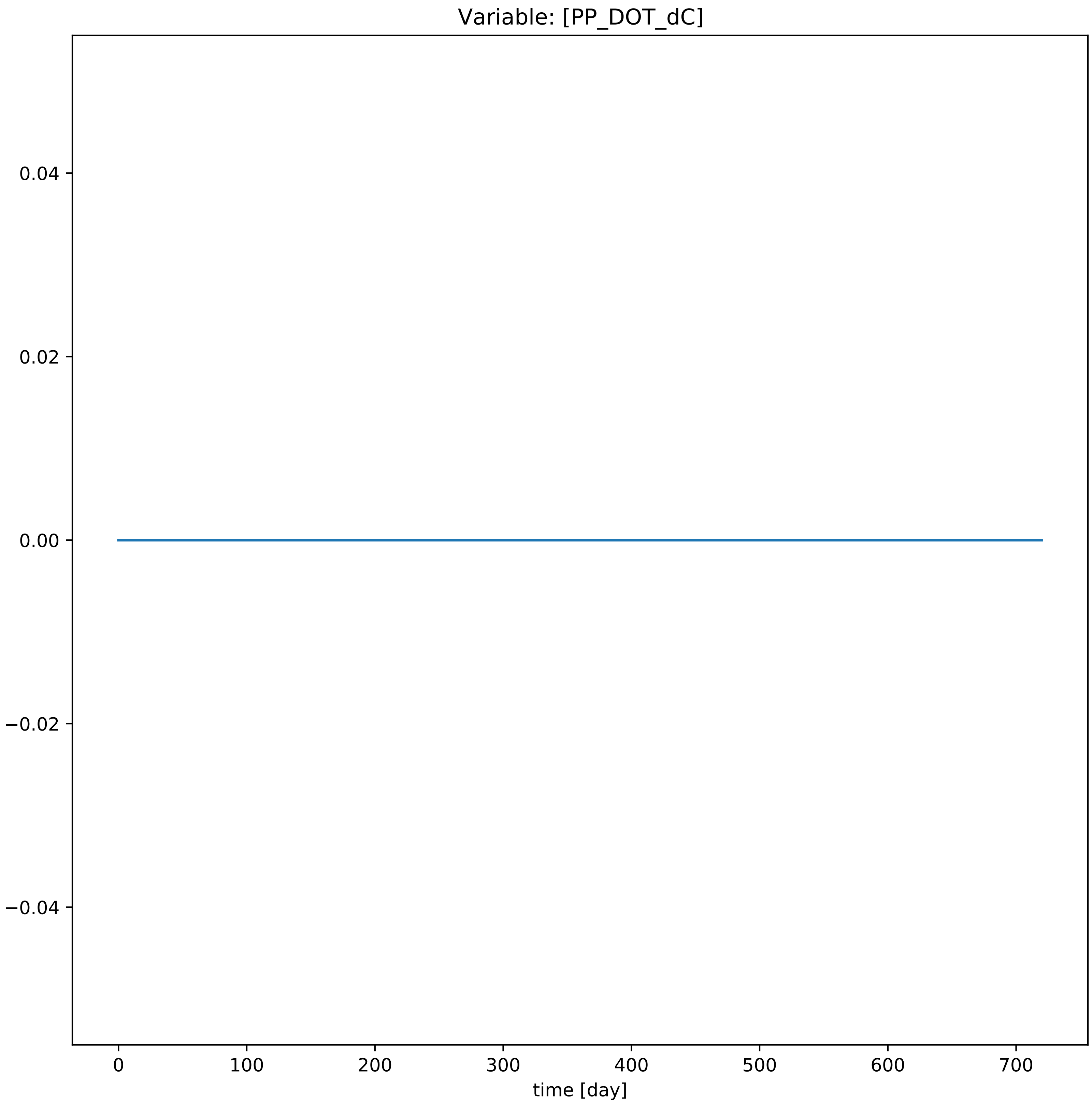

Variable: [PP_DOT_dC]
0.04
0.02
0.00
−0.02
−0.04
0
100
200
300
400
500
600
700
time [day]

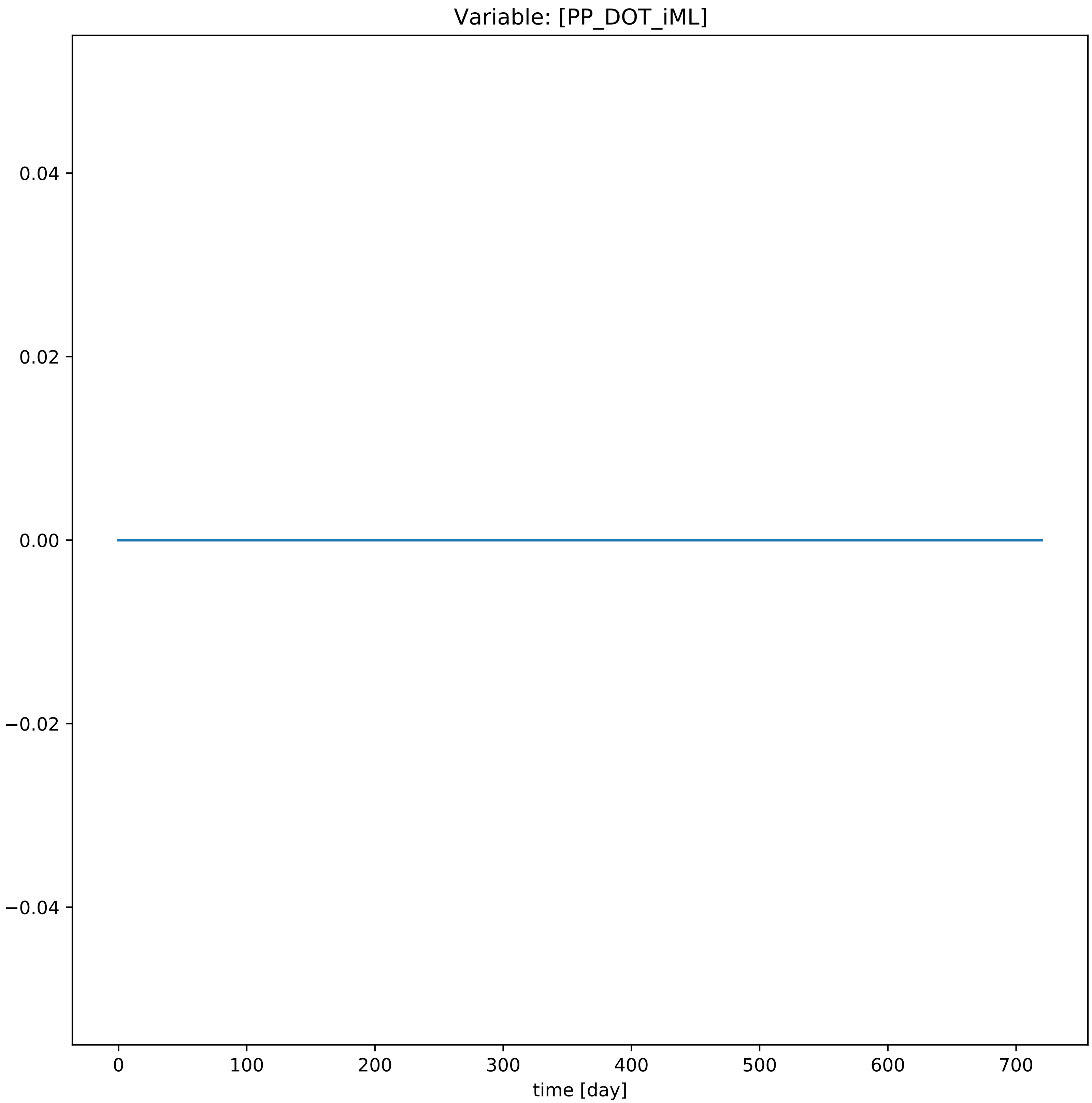

Variable: [PP_DOT_iML]
0.04
0.02
0.00
−0.02
−0.04
0
100
200
300
400
500
600
700
time [day]

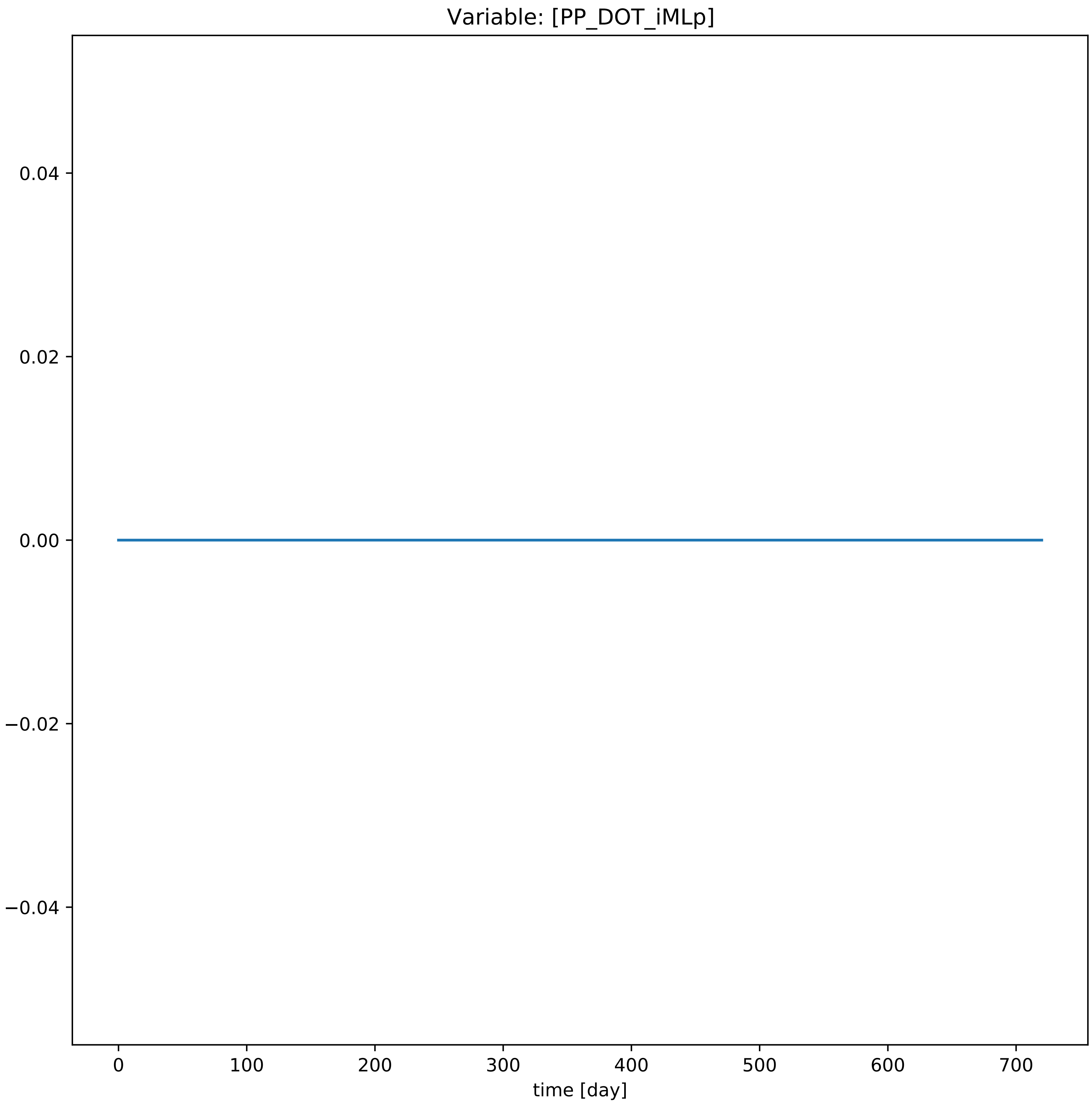

Variable: [PP_DOT_iMLp]
0.04
0.02
0.00
−0.02
−0.04
0
100
200
300
400
500
600
700
time [day]

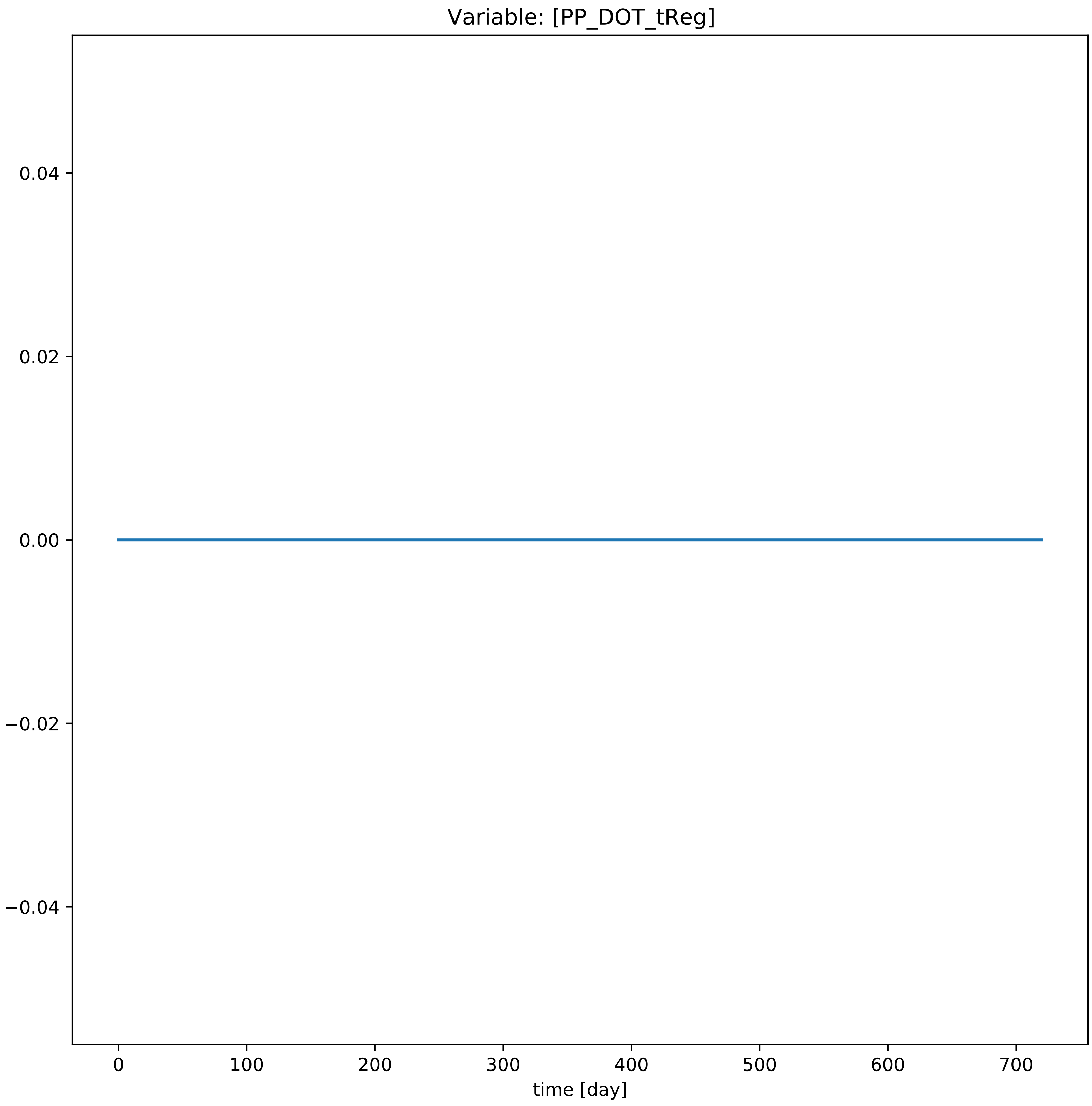
Variable: [PP_DOT_tReg]
0.04
0.02
0.00
−0.02
−0.04
0
100
200
300
400
500
600
700
time [day]

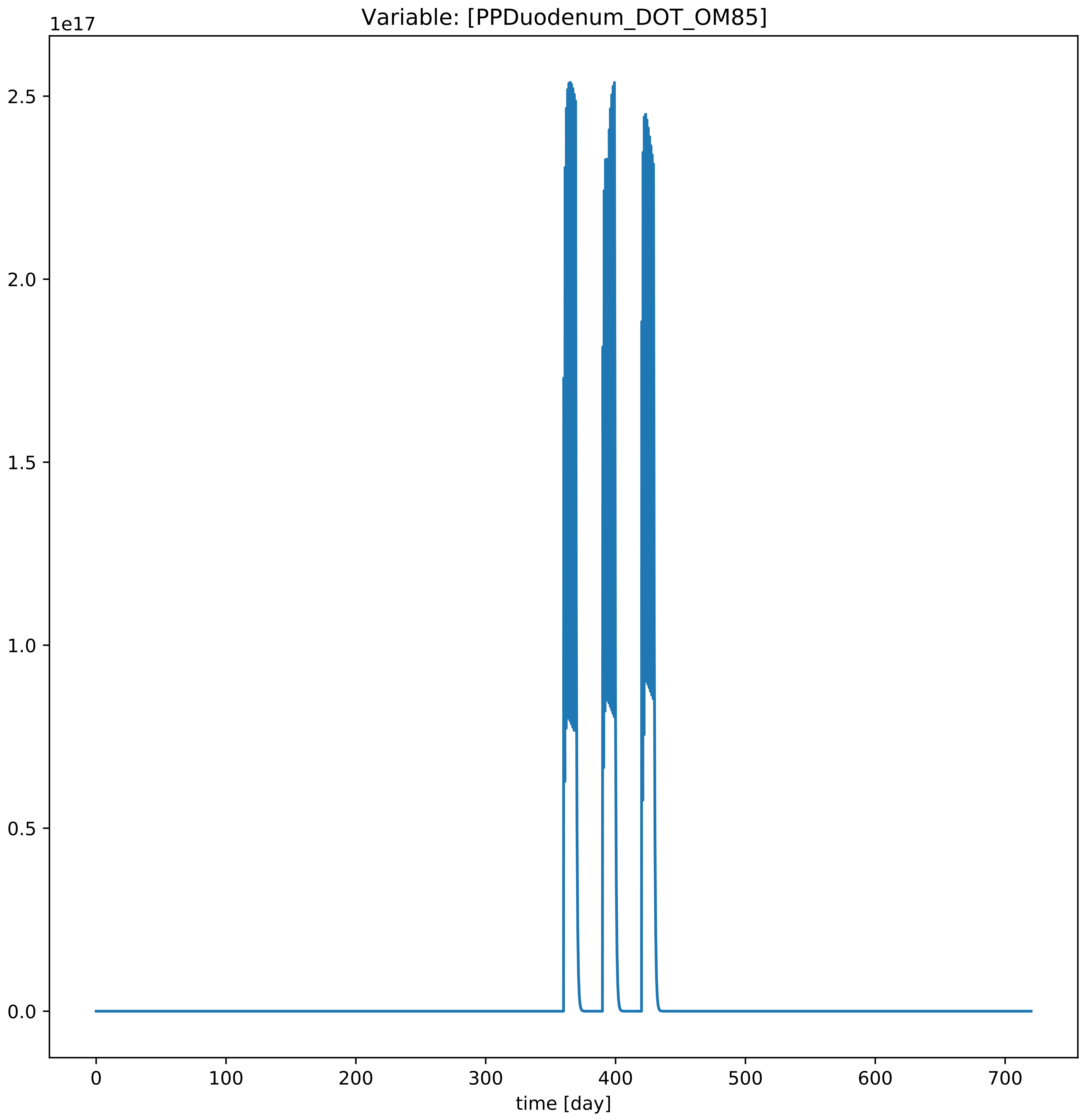
Variable: [PPDuodenum_DOT_OM85]
1e17
2.5
2.0
1.5
1.0
0.5
0.0
0
100
200
300
400
500
600
700
time [day]

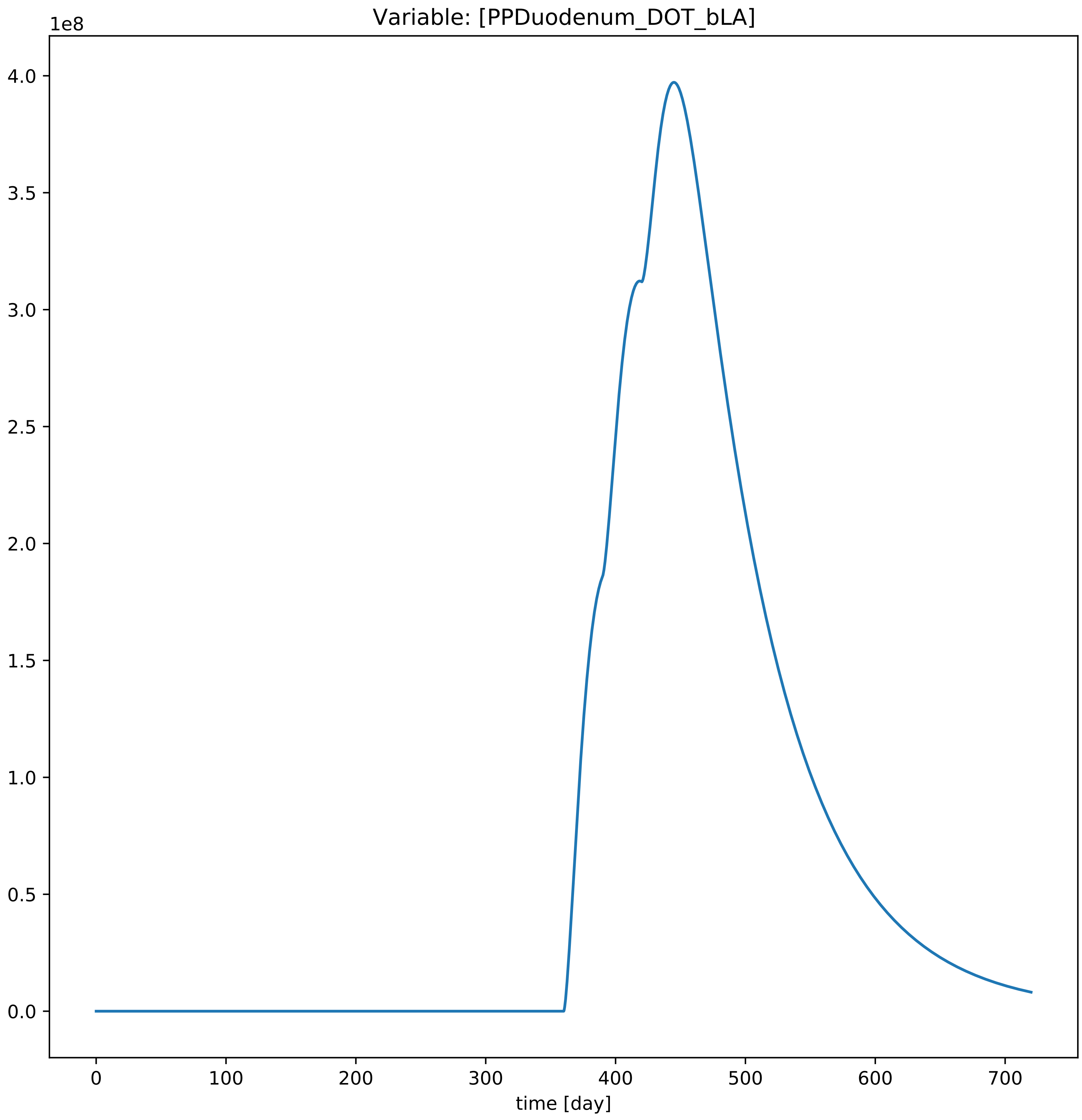

Variable: [PPDuodenum_DOT_bLA]
1e8
4.0
3.5
3.0
2.5
2.0
1.5
1.0
0.5
0.0
0
100
200
300
400
500
600
700
time [day]

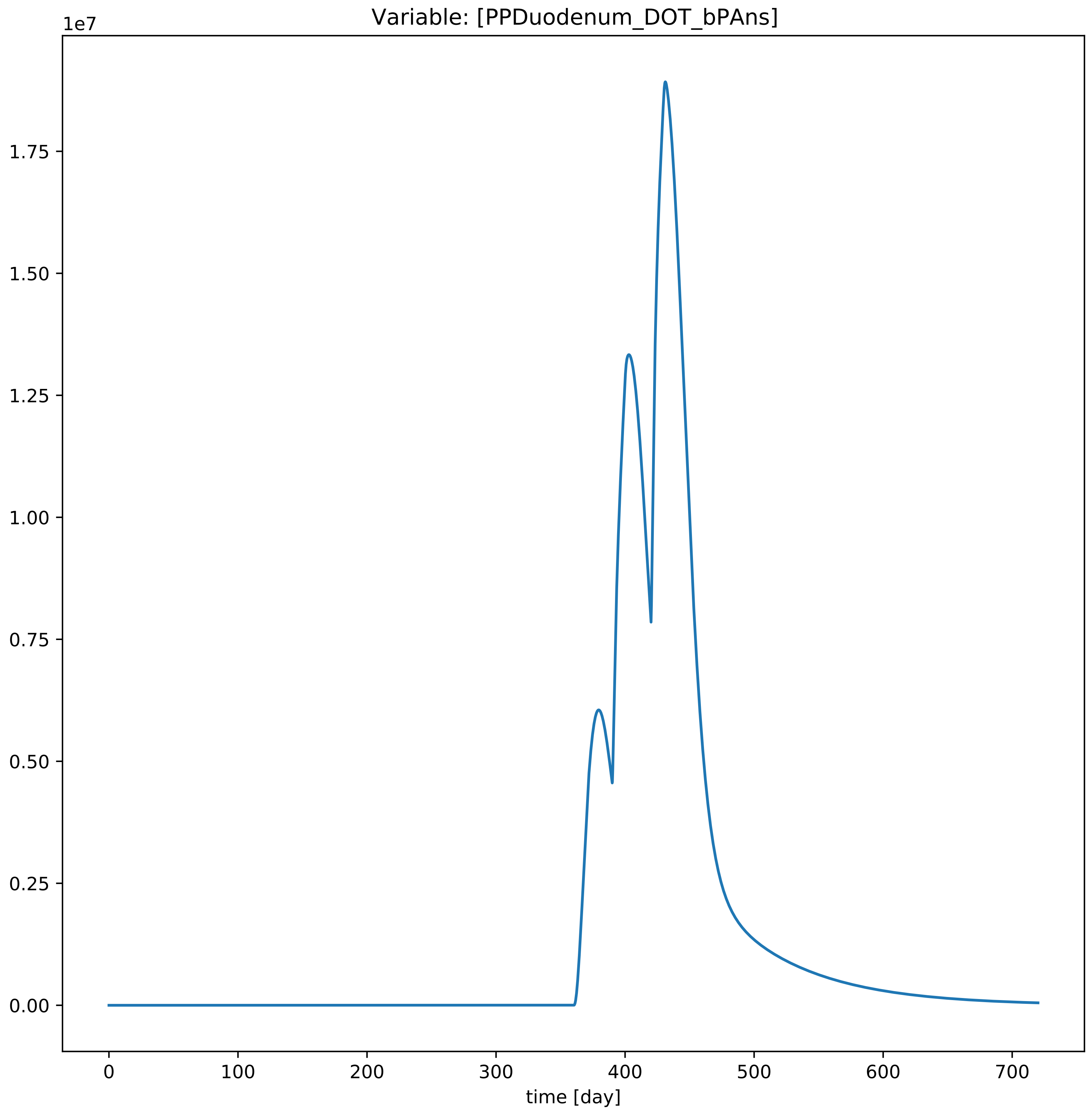

Variable: [PPDuodenum_DOT_bPAns]
1e7
1.75
1.50
1.25
1.00
0.75
0.50
0.25
0.00
0
100
200
300
400
500
600
700
time [day]

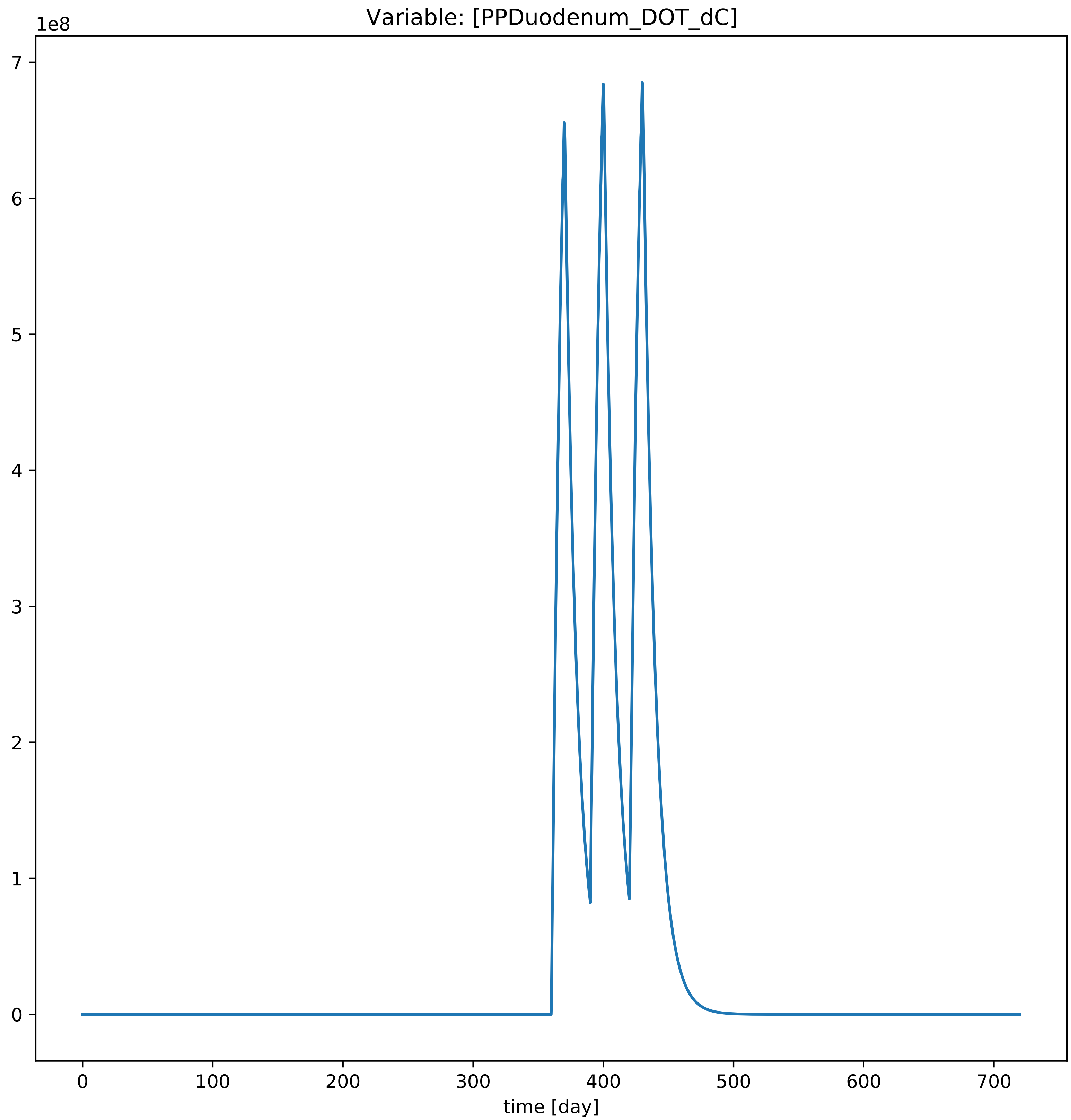
Variable: [PPDuodenum_DOT_dC]
1e8
7
6
5
4
3
2
1
0
0
100
200
300
400
500
600
700
time [day]

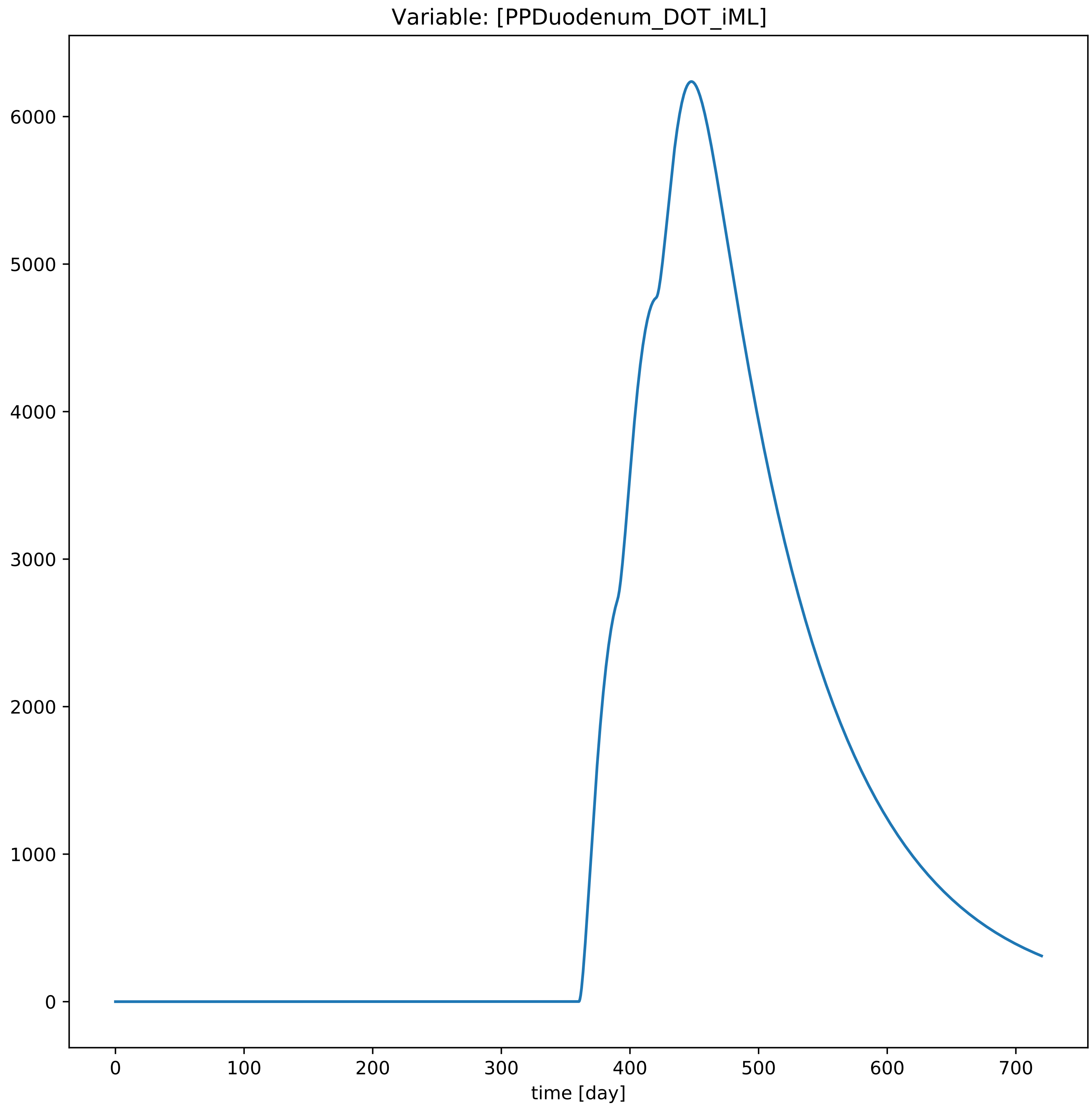

Variable: [PPDuodenum_DOT_iML]
6000
5000
4000
3000
2000
1000
0
0
100
200
300
400
500
600
700
time [day]

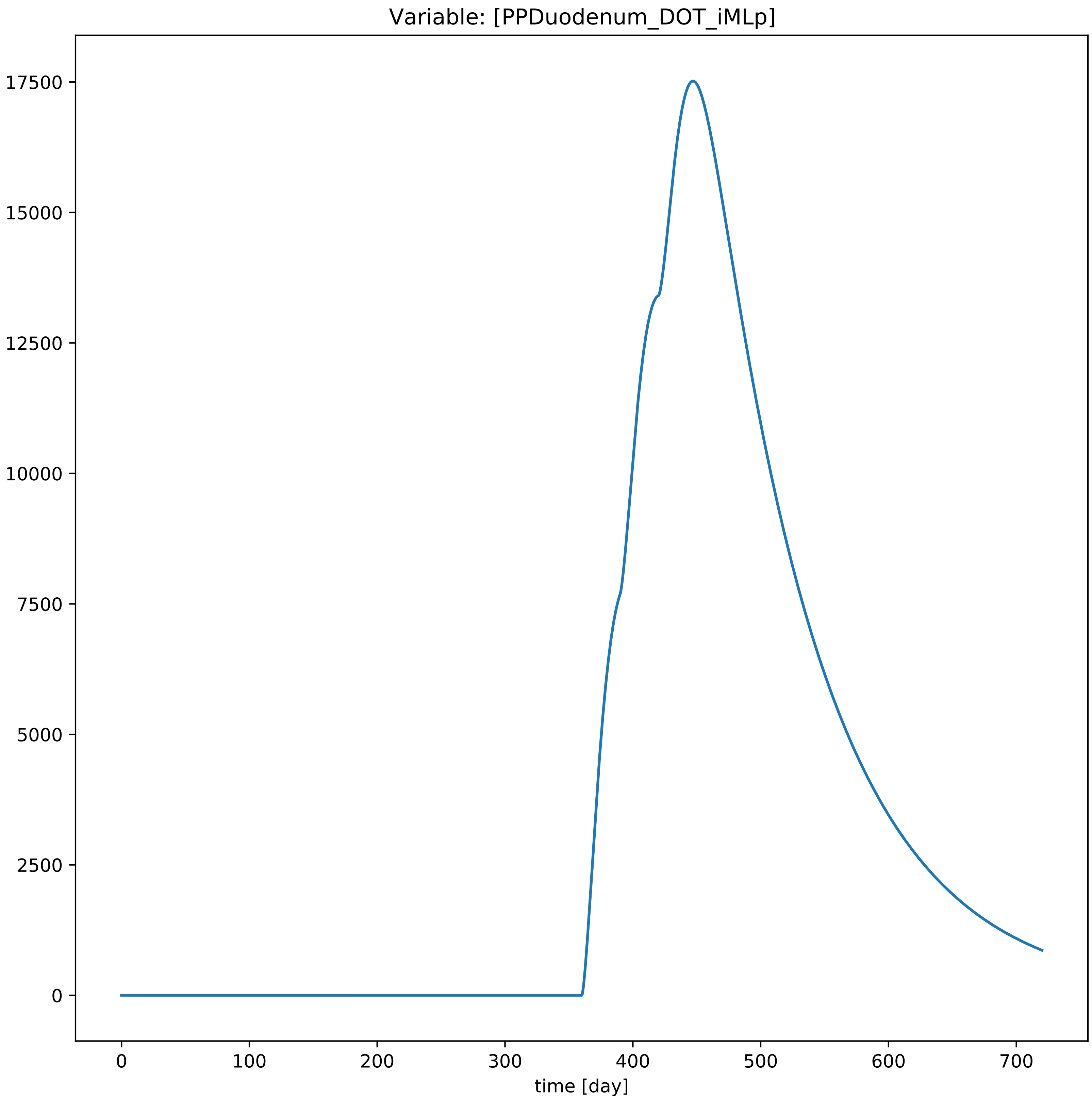

Variable: [PPDuodenum_DOT_iMLp]
17500
15000
12500
10000
7500
5000
2500
0
0
100
200
300
400
500
600
700
time [day]

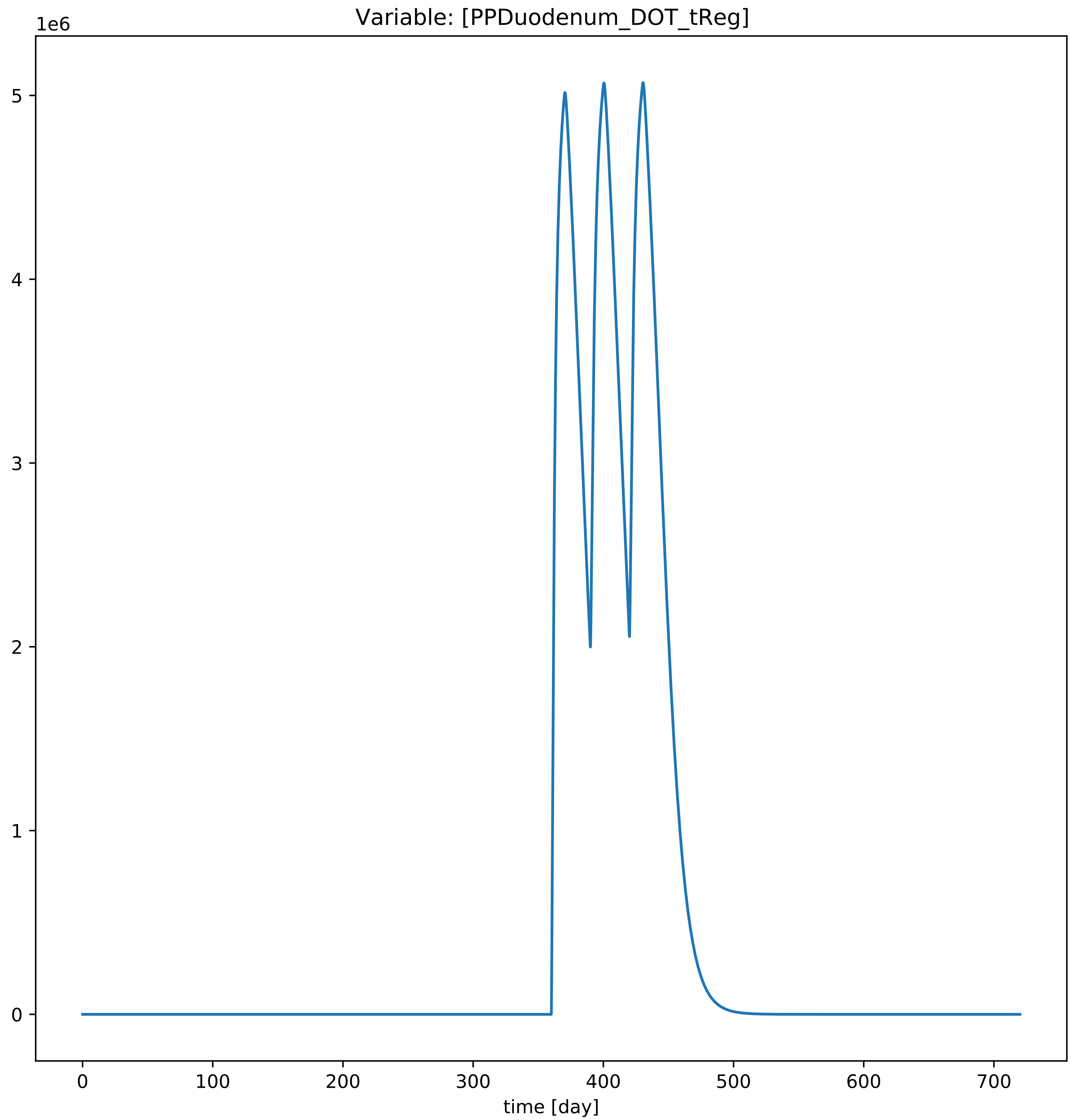

Variable: [PPDuodenum_DOT_tReg]
1e6
5
4
3
2
1
0
0
100
200
300
400
500
600
700
time [day]

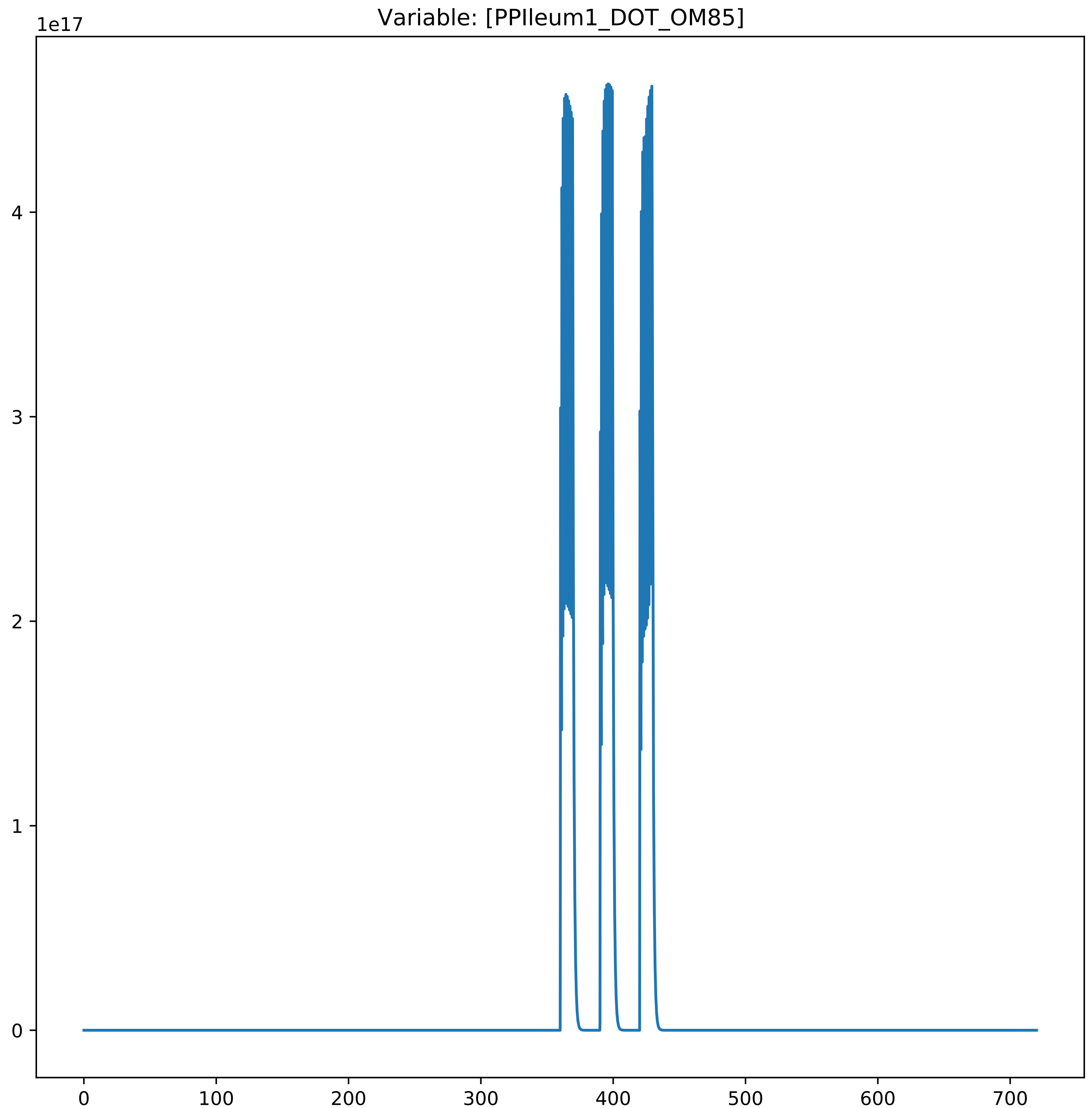

Variable: [PPIleum1_DOT_OM85]
1e17
0
1
2
3
4
0
100
200
300
400
500
600
700
time [day]

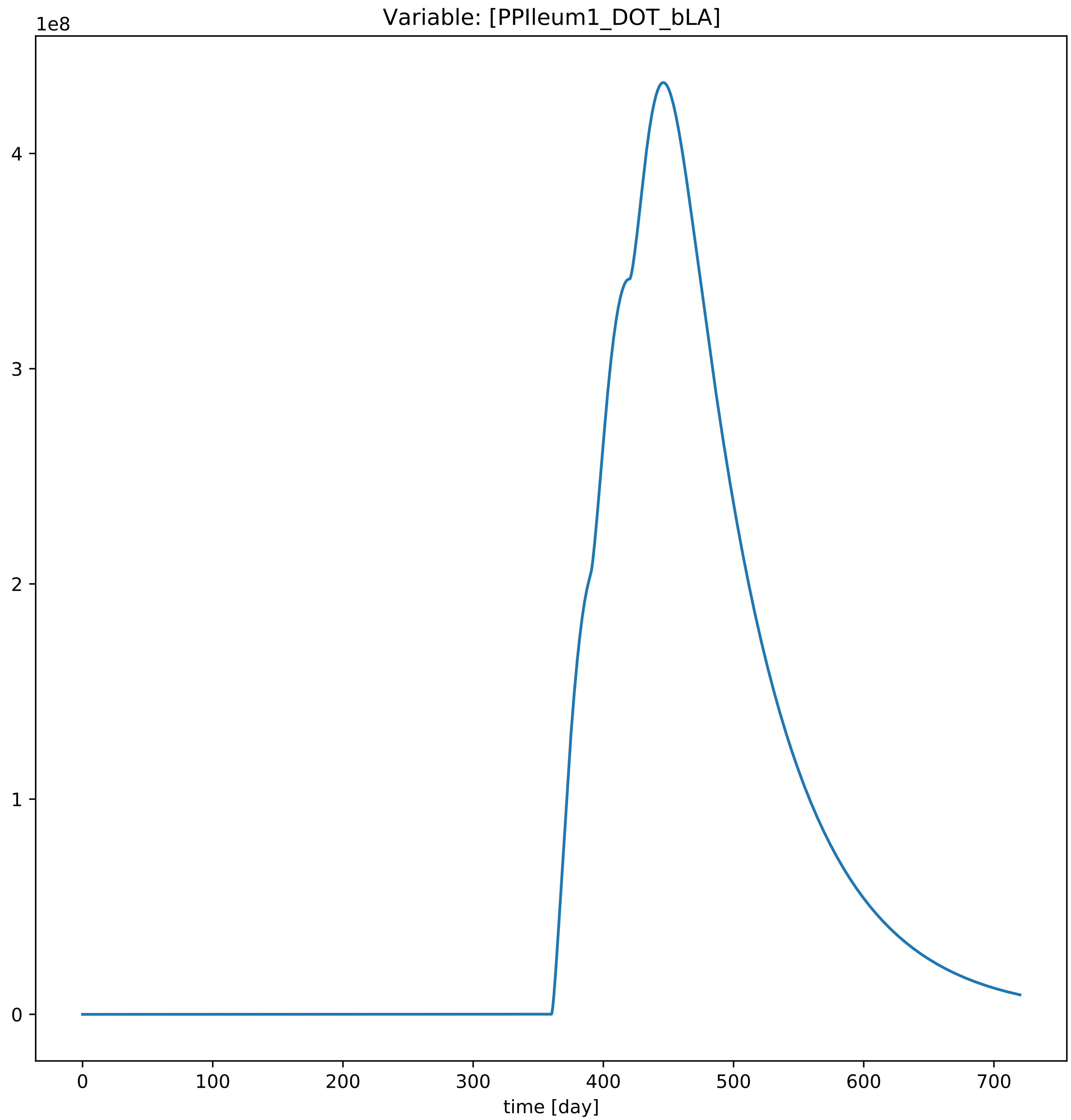

Variable: [PPIleum1_DOT_bLA]
1e8
4
3
2
1
0
0
100
200
300
400
500
600
700
time [day]

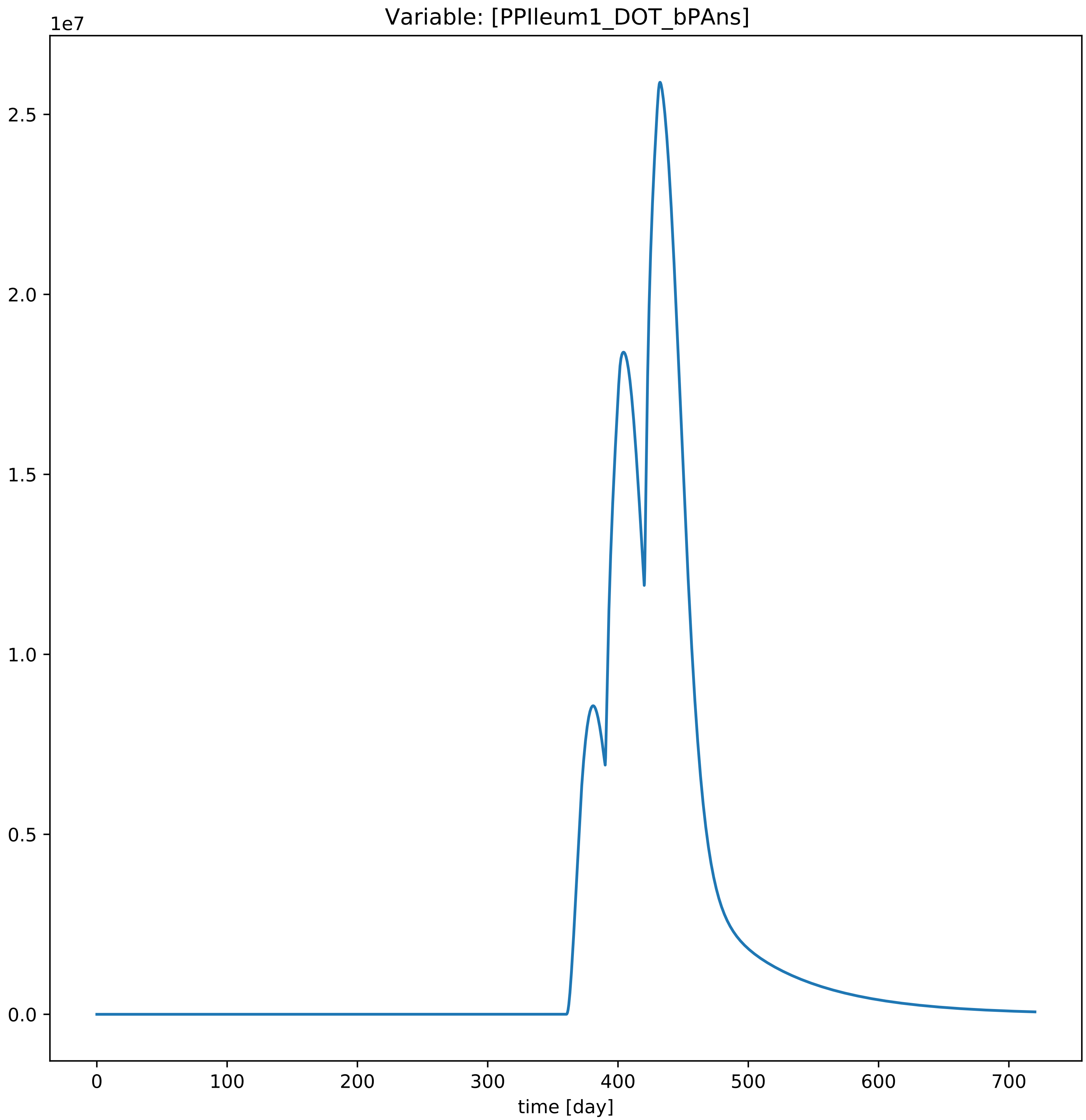

Variable: [PPIleum1_DOT_bPAns]
1e7
2.5
2.0
1.5
1.0
0.5
0.0
0
100
200
300
400
500
600
700
time [day]

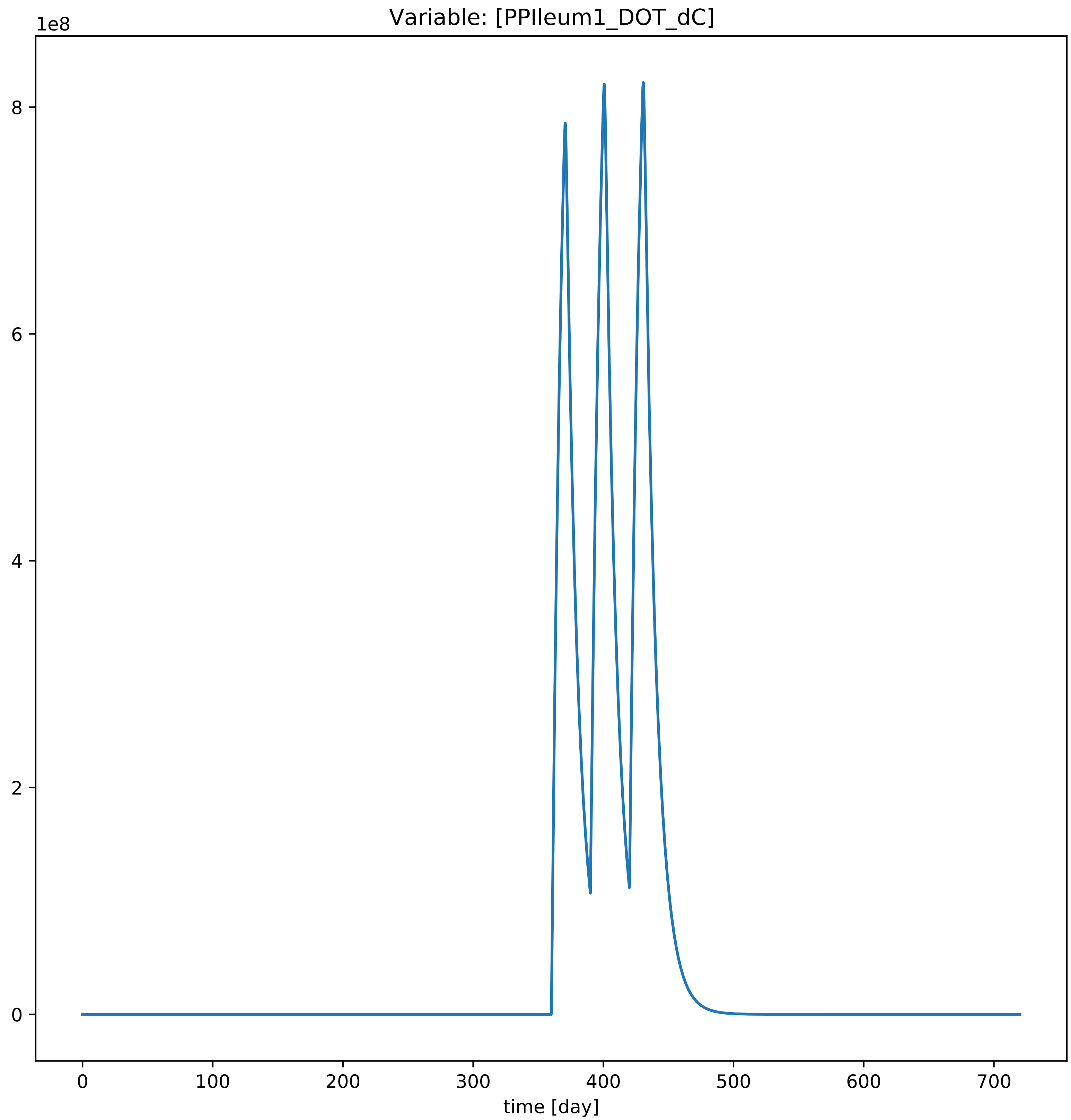

Variable: [PPIleum1_DOT_dC]
1e8
8
6
4
2
0
0
100
200
300
400
500
600
700
time [day]

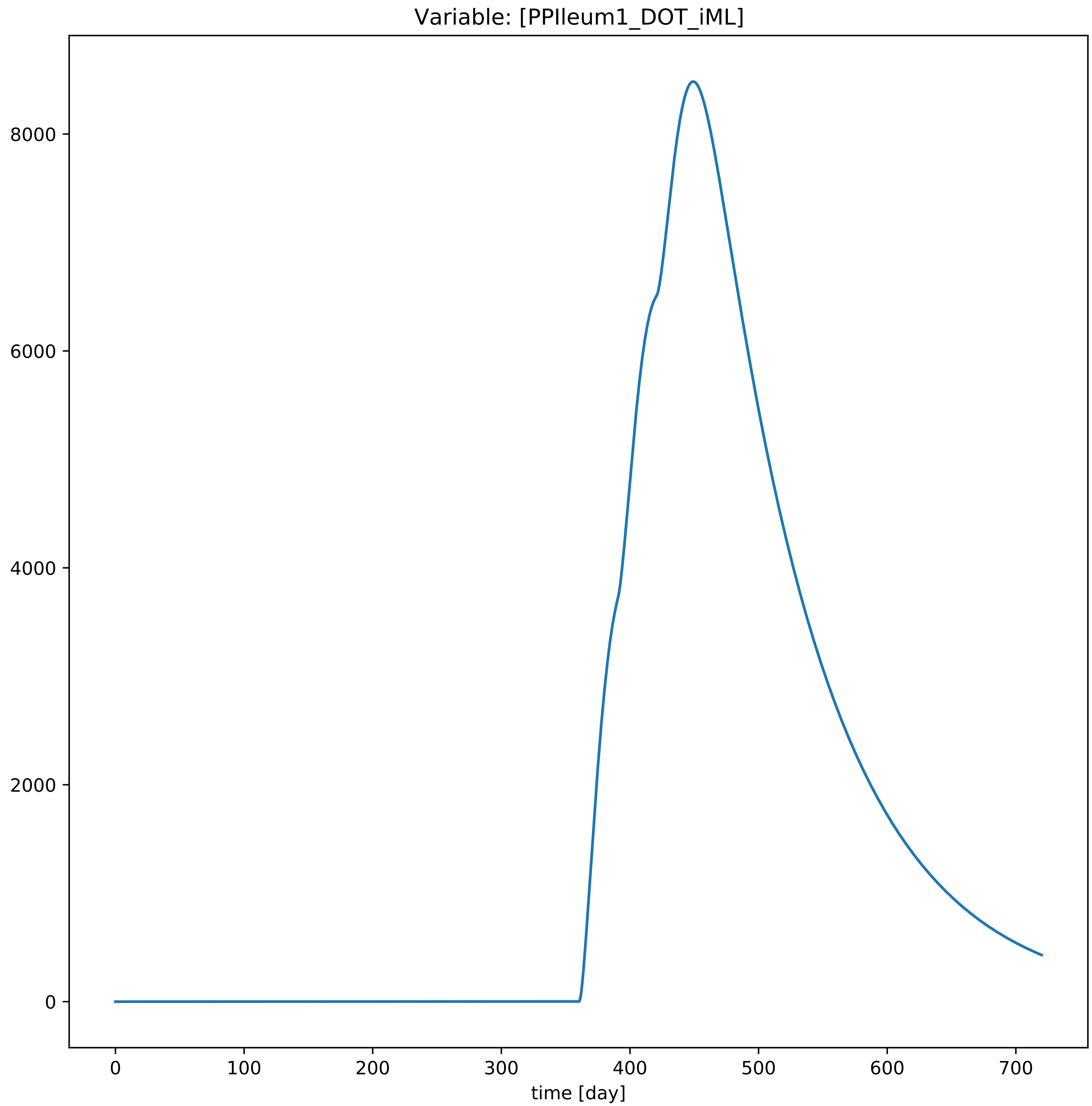

Variable: [PPIleum1_DOT_iML]
8000
6000
4000
2000
0
0
100
200
300
400
500
600
700
time [day]

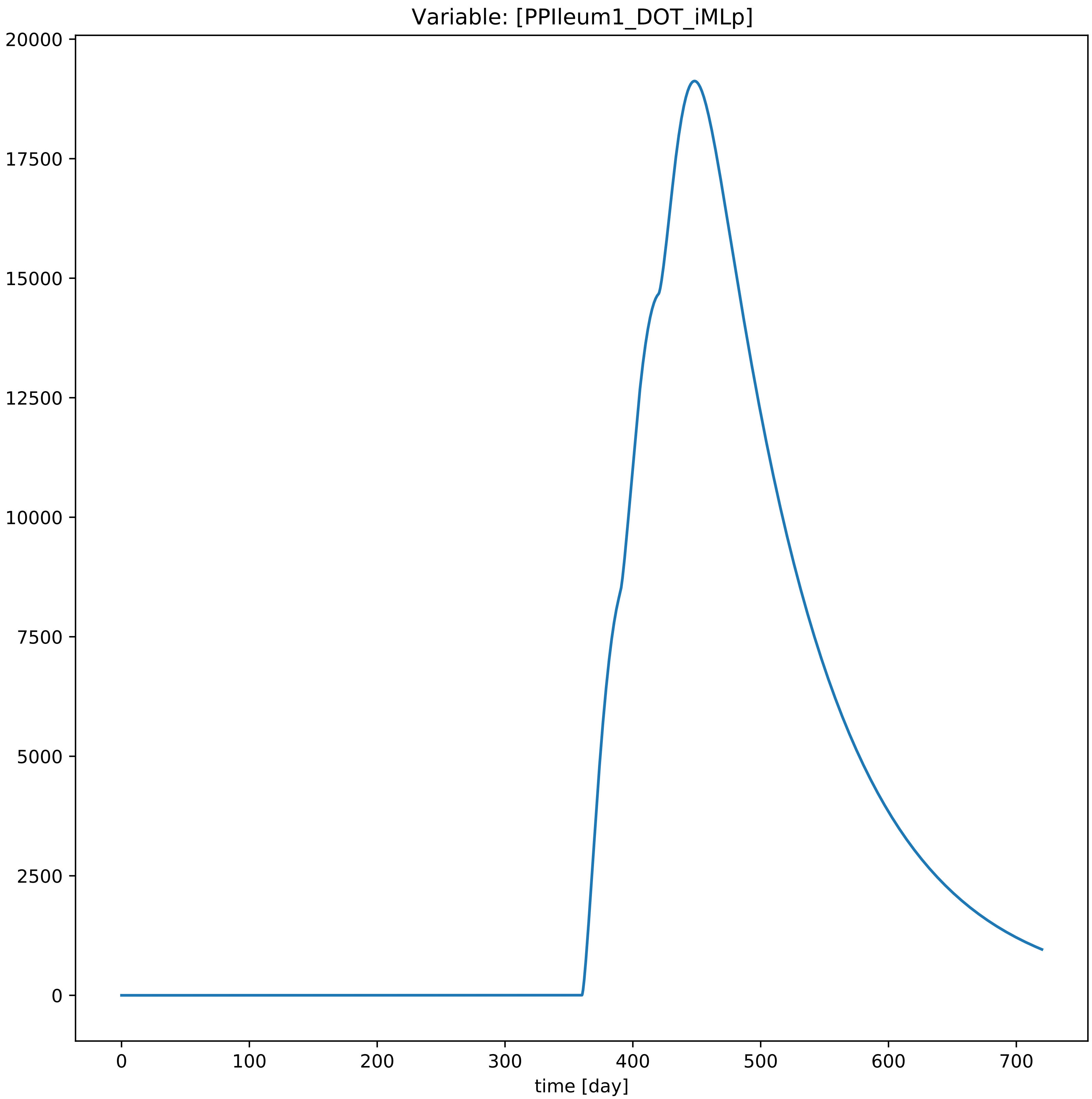

Variable: [PPIleum1_DOT_iMLp]
20000
17500
15000
12500
10000
7500
5000
2500
0
0
100
200
300
400
500
600
700
time [day]

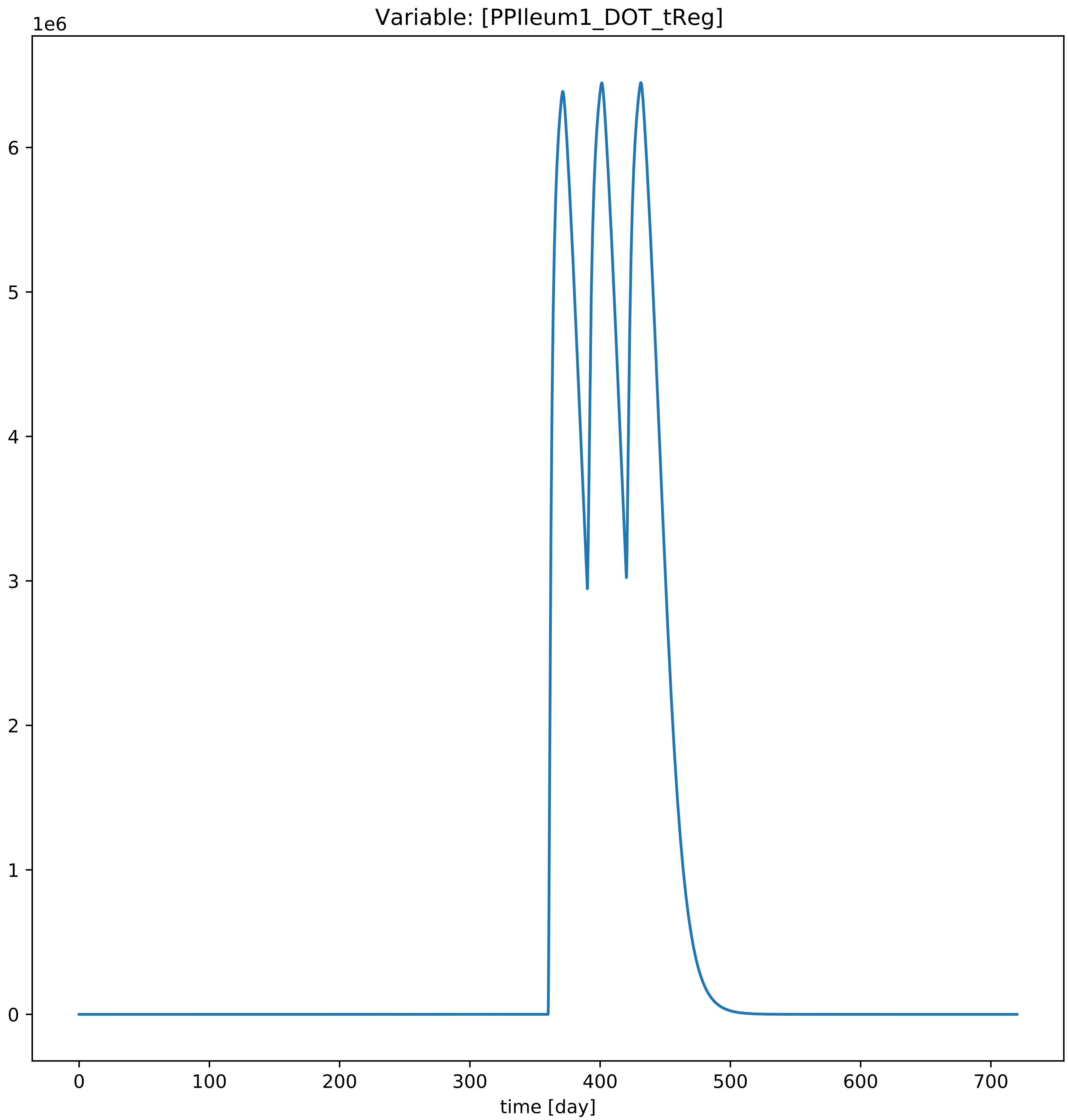

Variable: [PPIleum1_DOT_tReg]
1e6
0
1
2
3
4
5
6
0
100
200
300
400
500
600
700
time [day]

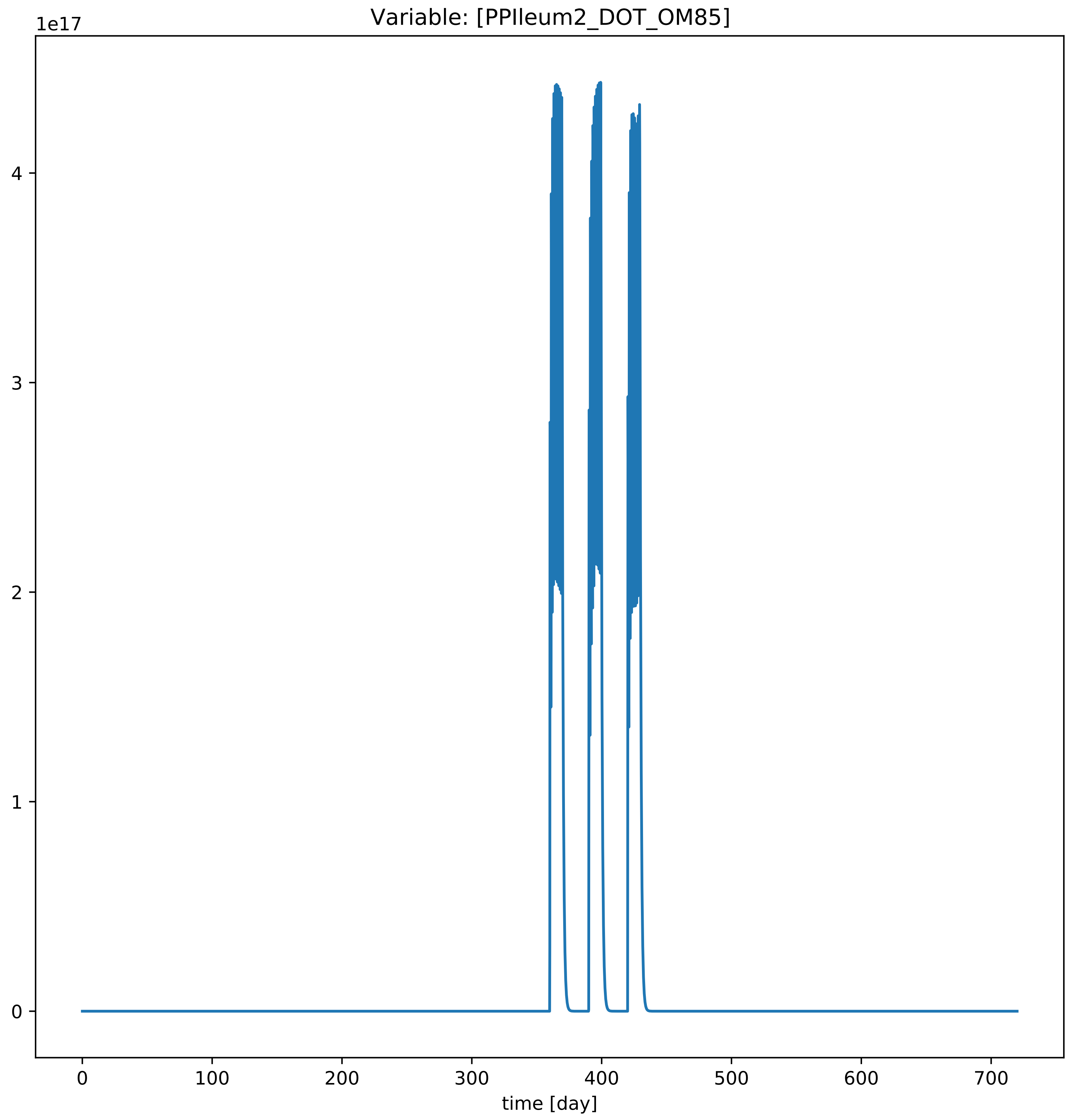

Variable: [PPIleum2_DOT_OM85]
1e17
0
1
2
3
4
0
100
200
300
400
500
600
700
time [day]

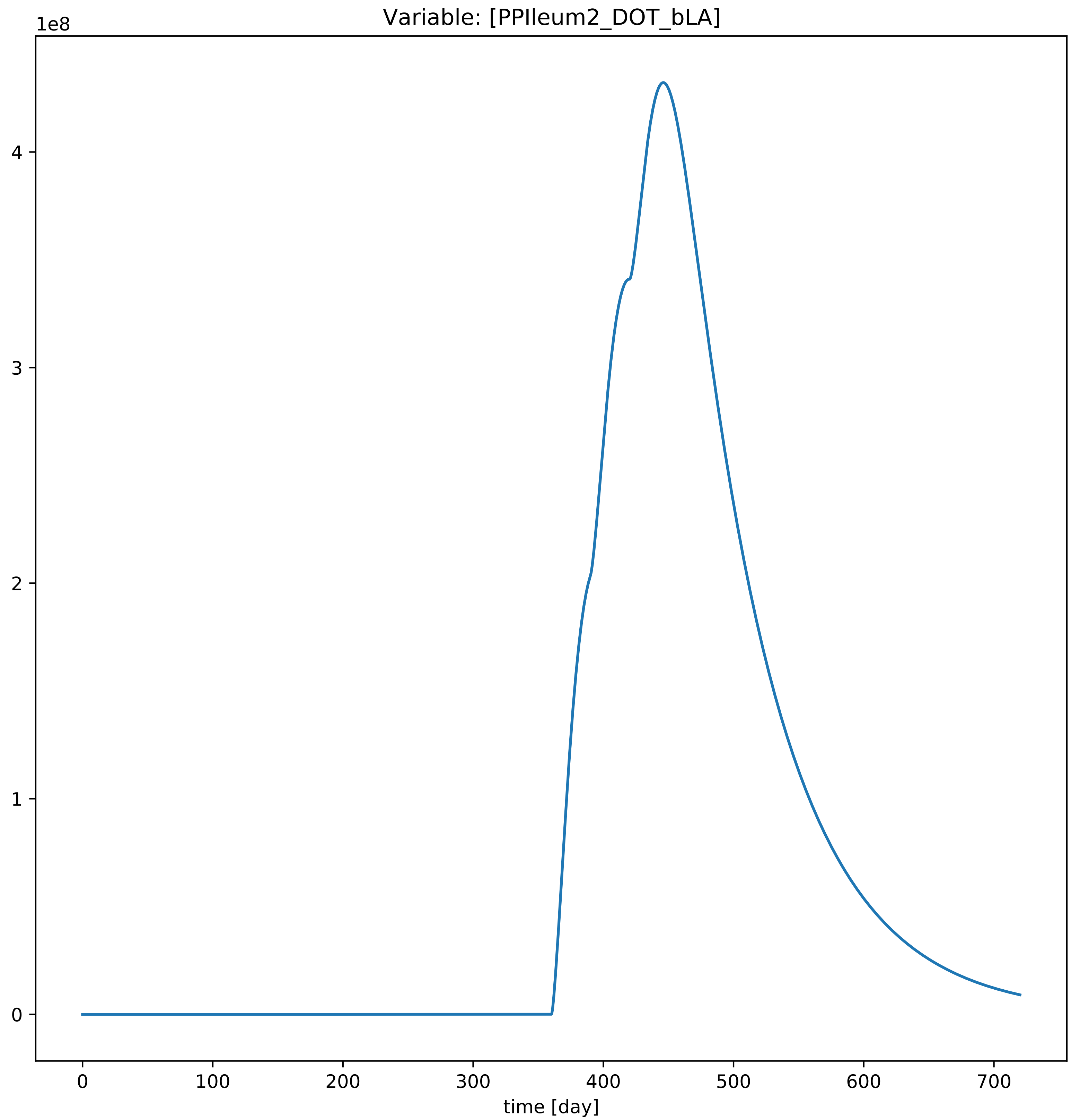

Variable: [PPIleum2_DOT_bLA]
1e8
4
3
2
1
0
0
100
200
300
400
500
600
700
time [day]

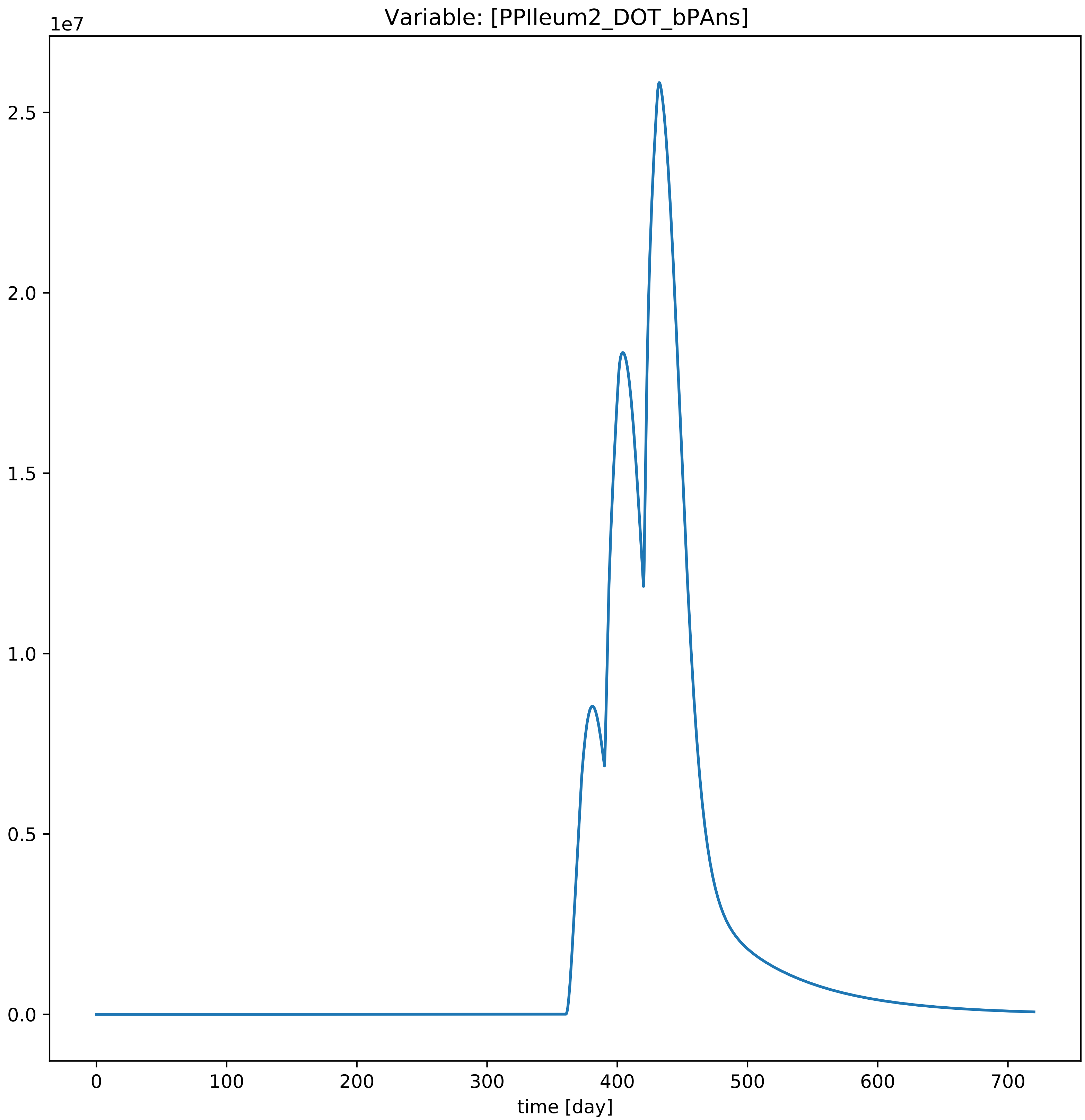

Variable: [PPIleum2_DOT_bPAns]
1e7
2.5
2.0
1.5
1.0
0.5
0.0
0
100
200
300
400
500
600
700
time [day]

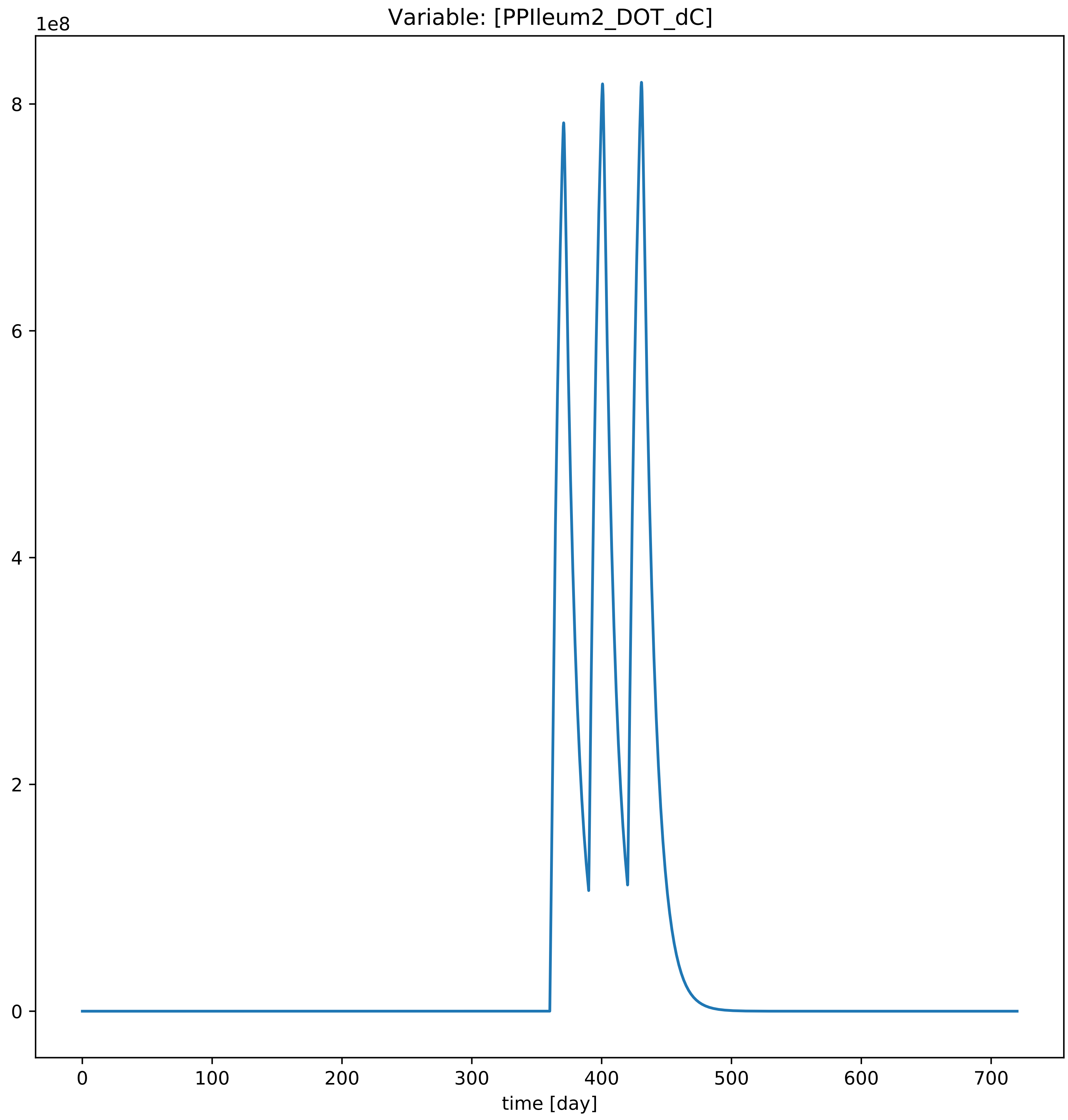

Variable: [PPIleum2_DOT_dC]
1e8
8
6
4
2
0
0
100
200
300
400
500
600
700
time [day]

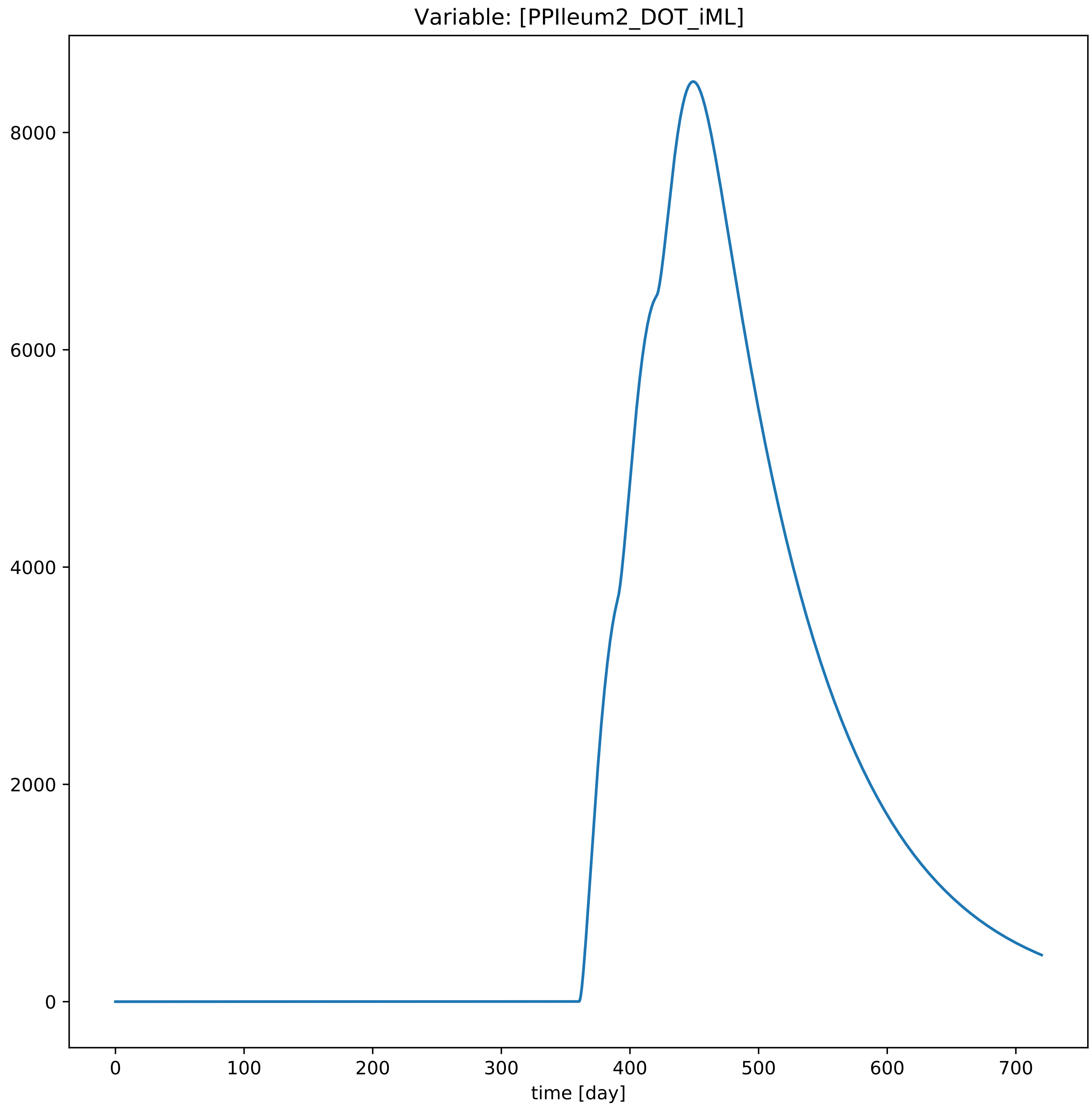
Variable: [PPIleum2_DOT_iML]
8000
6000
4000
2000
0
0
100
200
300
400
500
600
700
time [day]

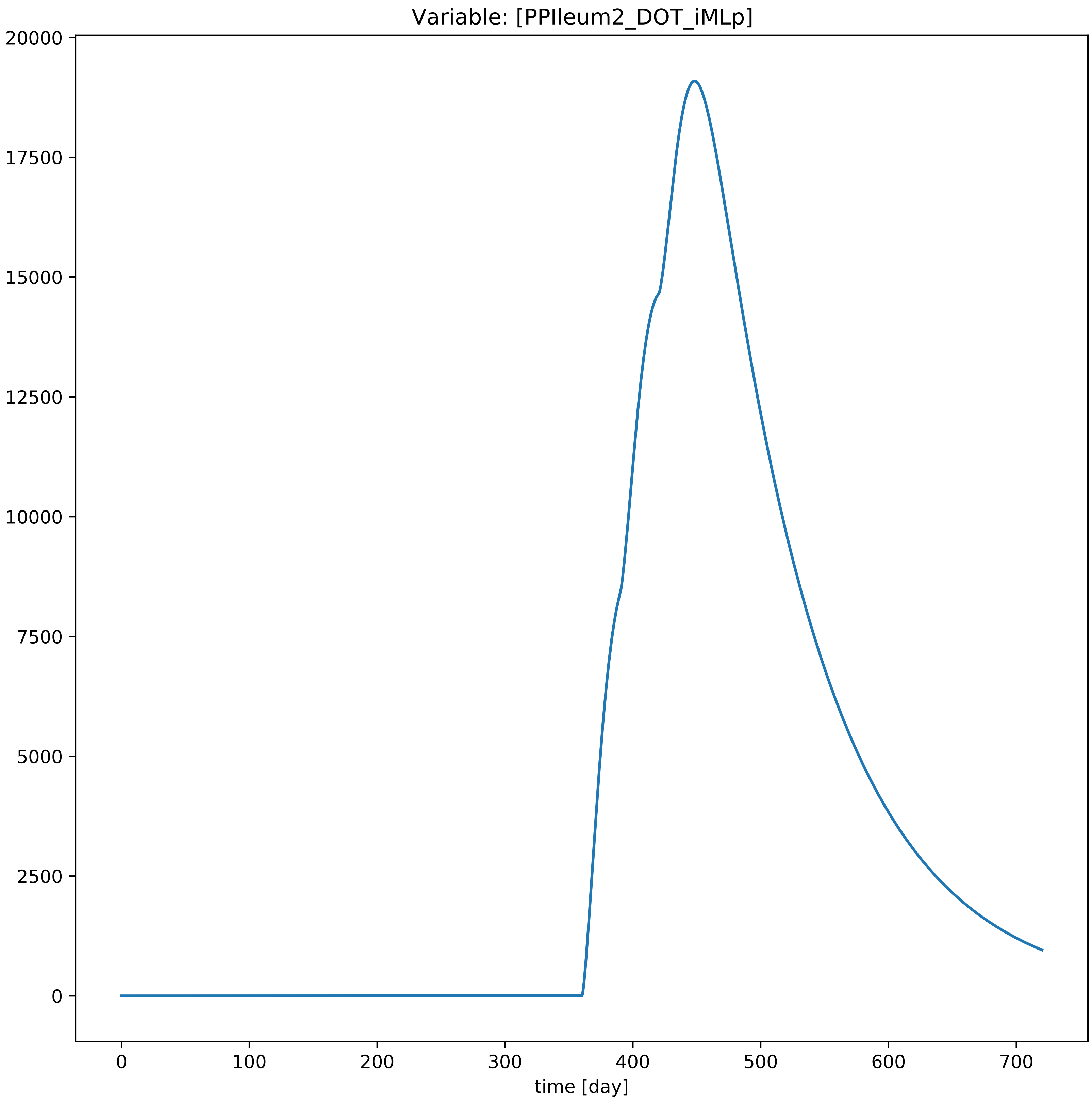
Variable: [PPIleum2_DOT_iMLp]
20000
17500
15000
12500
10000
7500
5000
2500
0
0
100
200
300
400
500
600
700
time [day]

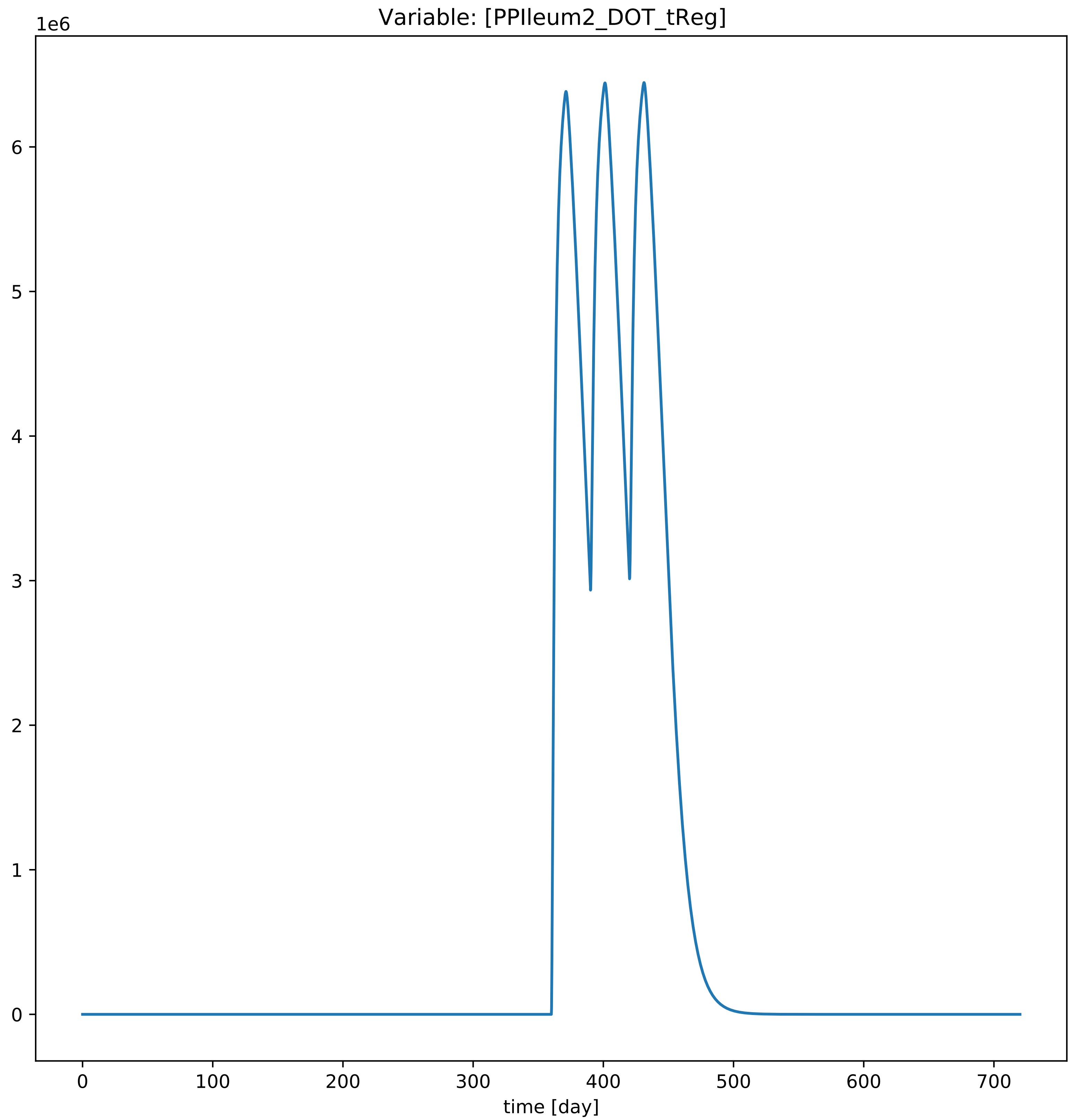

Variable: [PPIleum2_DOT_tReg]
1e6
6
5
4
3
2
1
0
0
100
200
300
400
500
600
700
time [day]

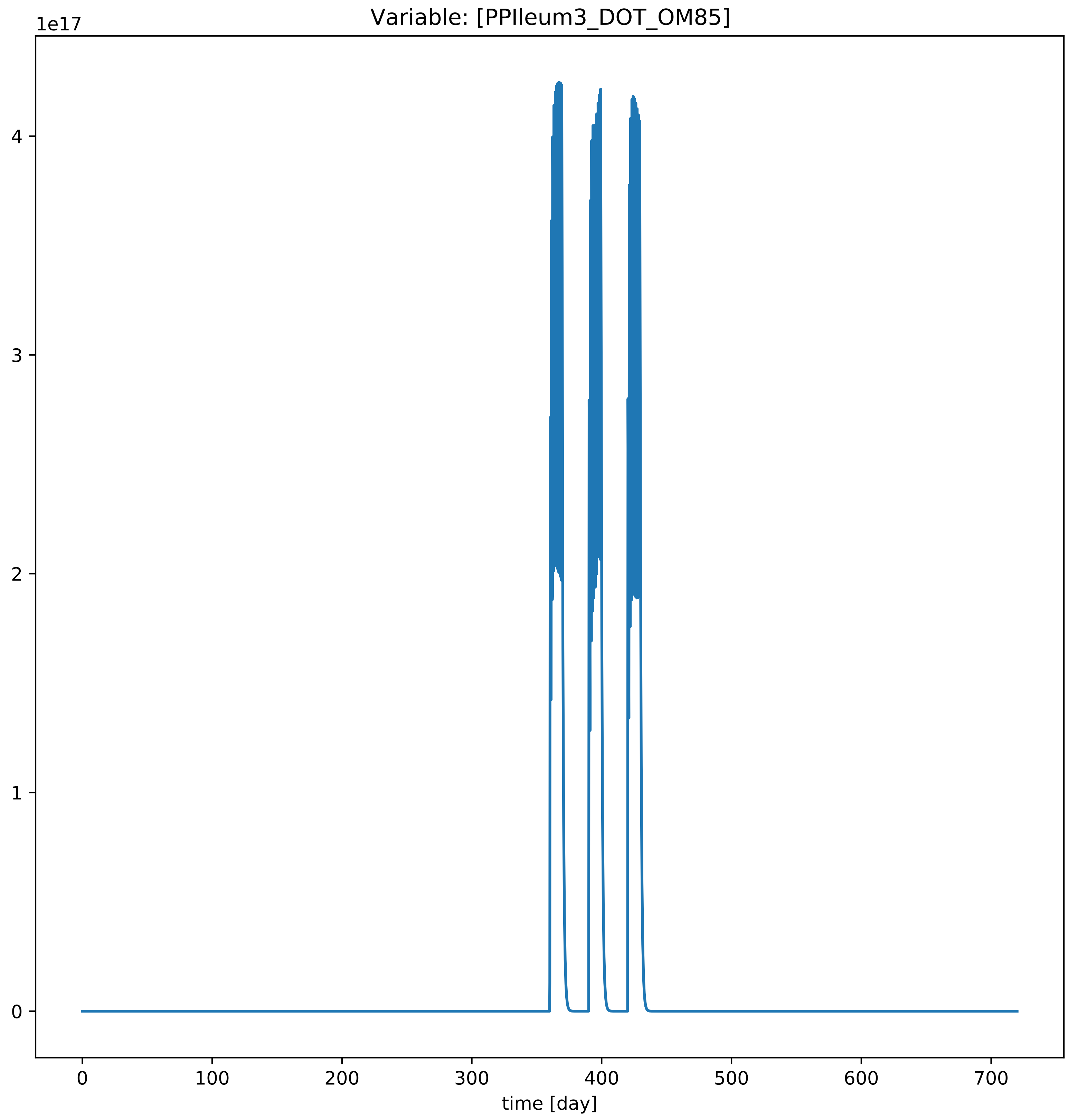

Variable: [PPIleum3_DOT_OM85]
1e17
4
3
2
1
0
0
100
200
300
400
500
600
700
time [day]

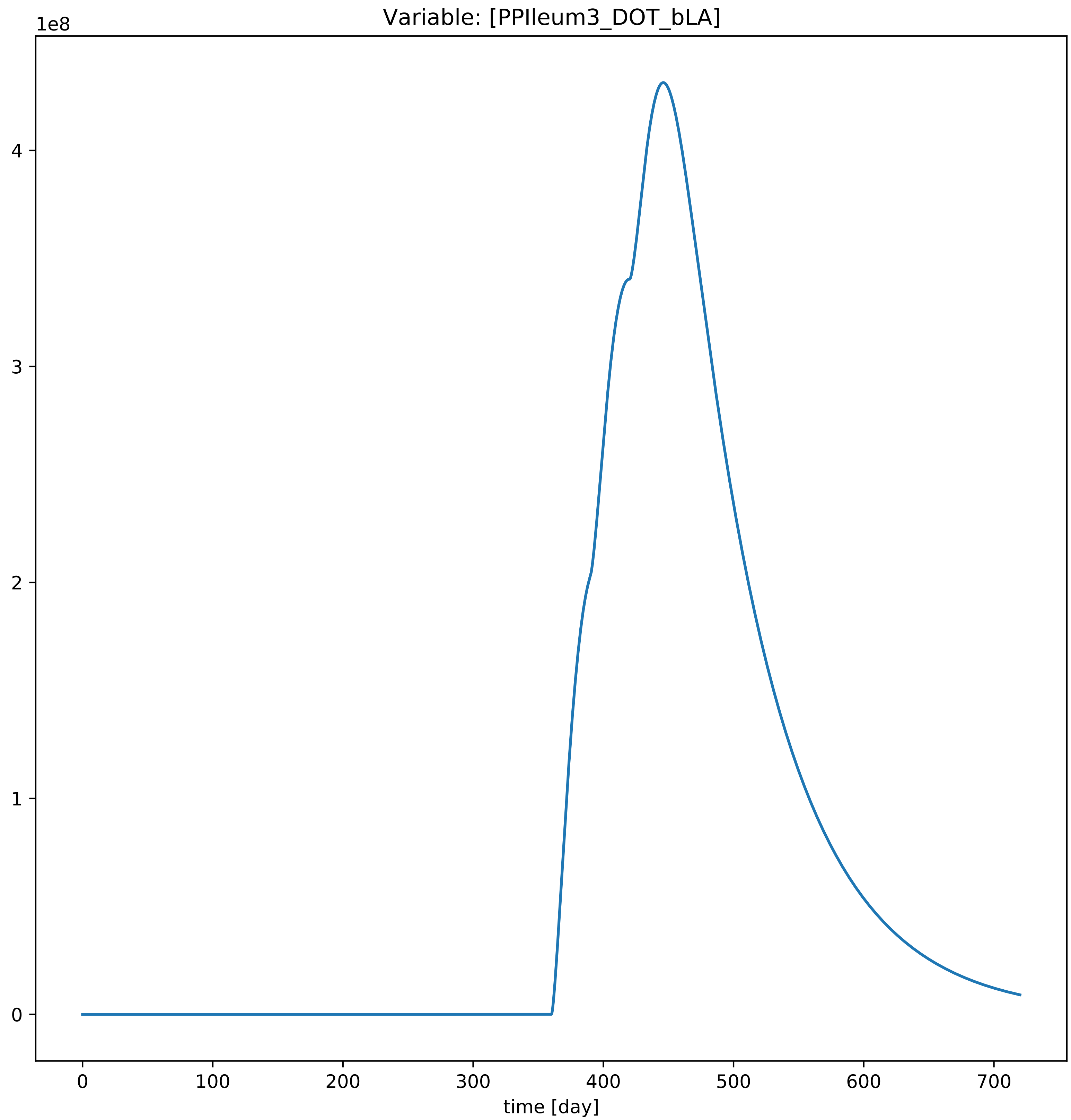

Variable: [PPIleum3_DOT_bLA]
1e8
4
3
2
1
0
0
100
200
300
400
500
600
700
time [day]

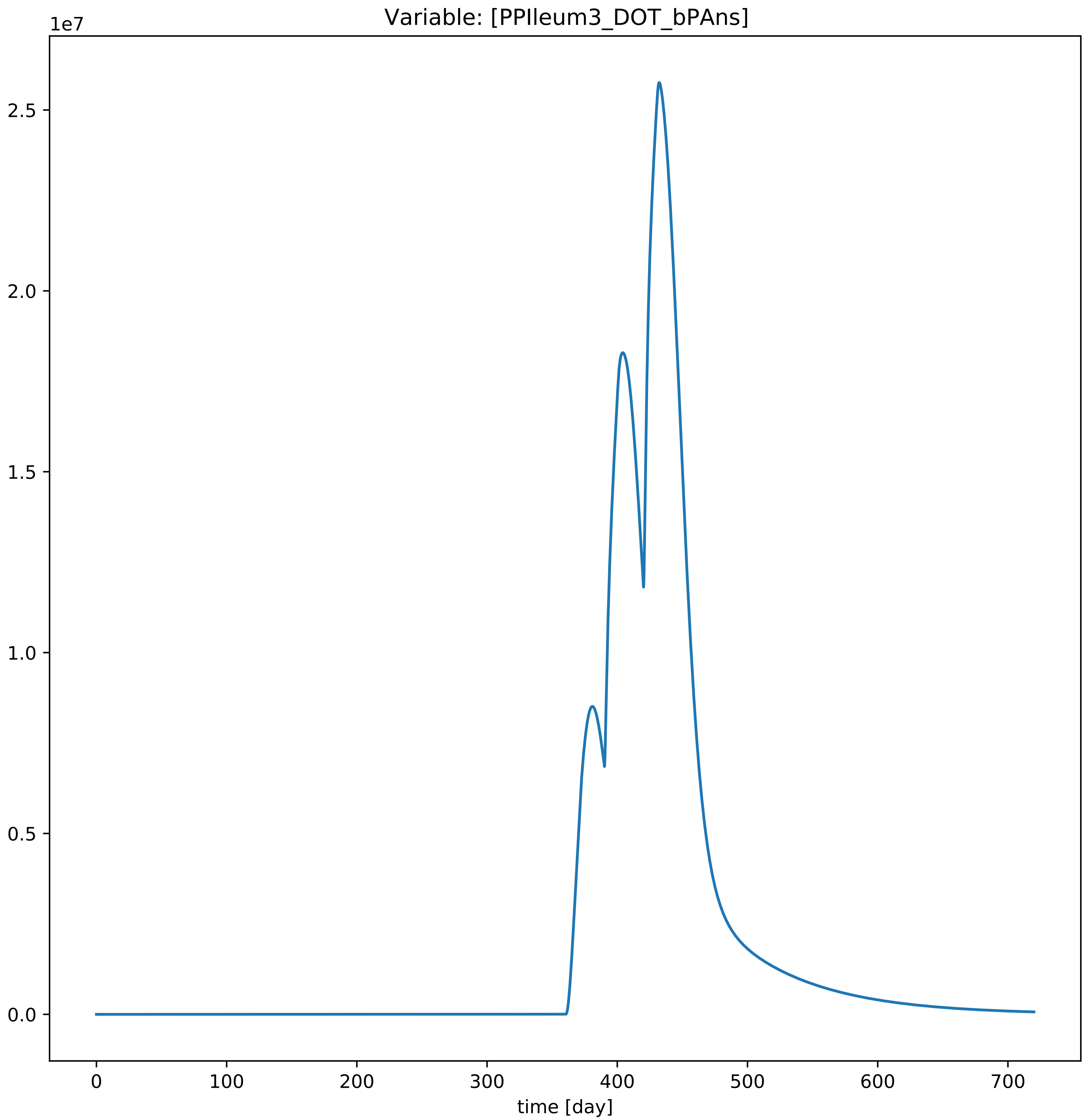

Variable: [PPIleum3_DOT_bPAns]
1e7
2.5
2.0
1.5
1.0
0.5
0.0
0
100
200
300
400
500
600
700
time [day]

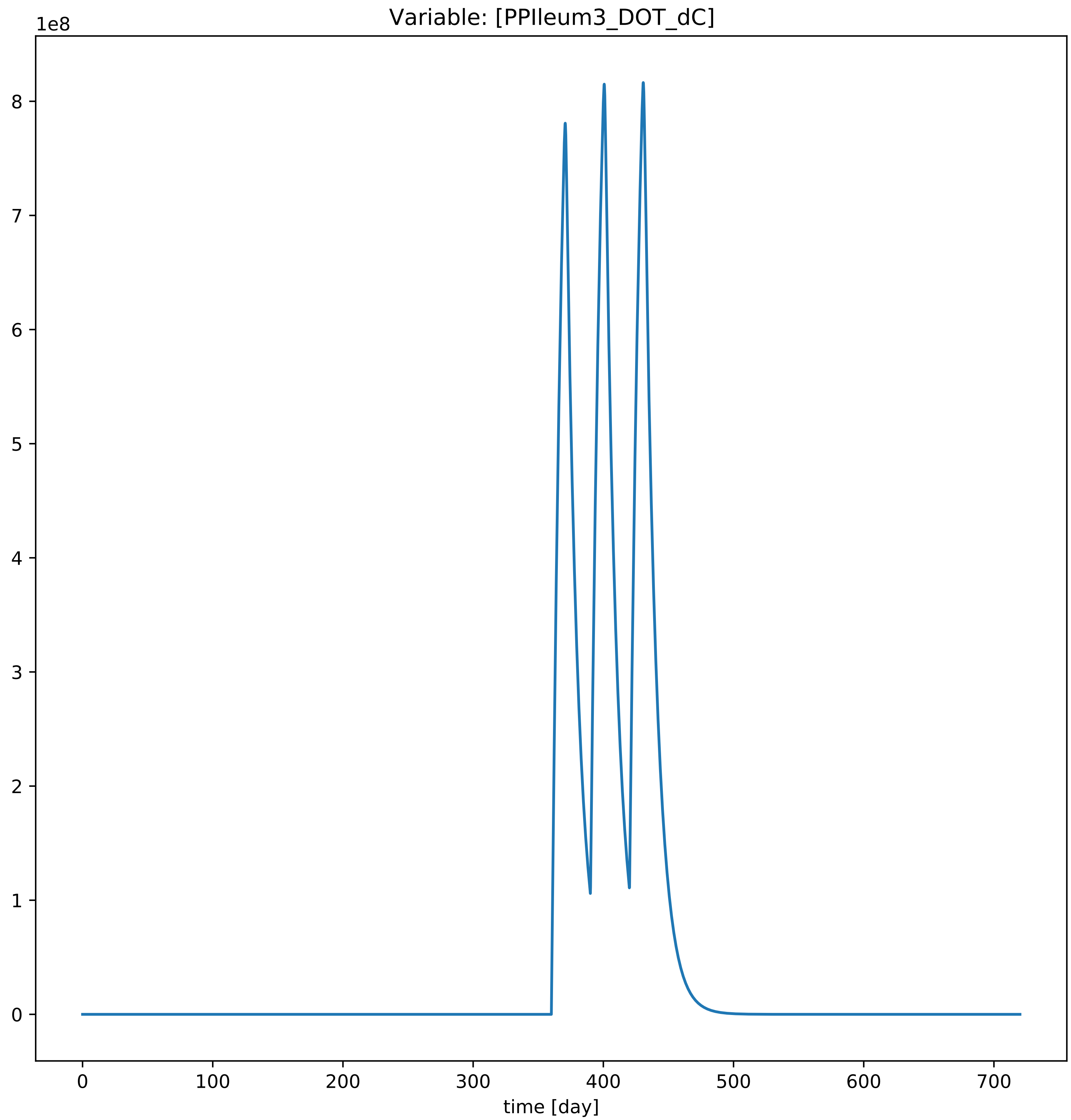

Variable: [PPIleum3_DOT_dC]
1e8
8
7
6
5
4
3
2
1
0
0
100
200
300
400
500
600
700
time [day]

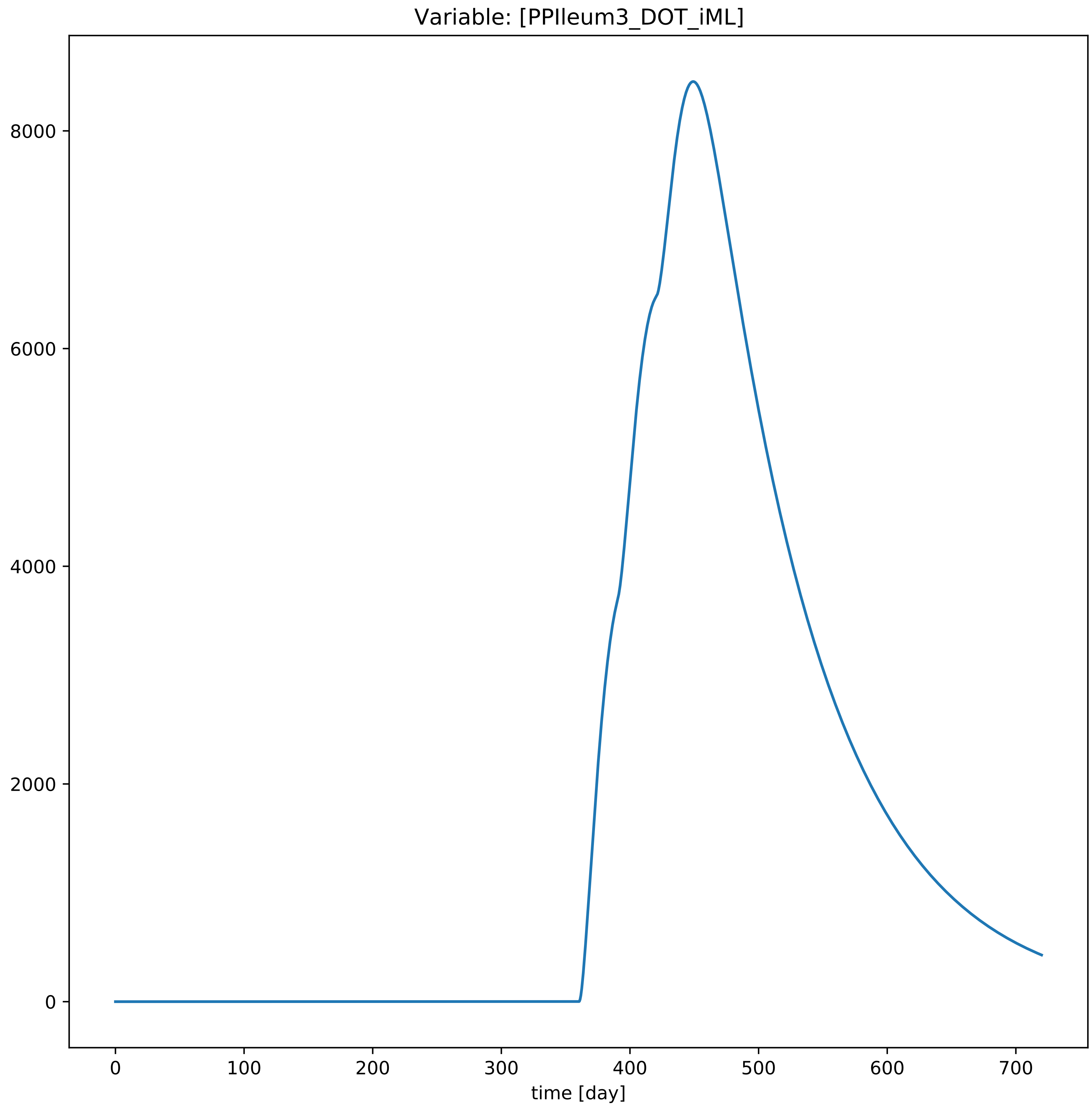
Variable: [PPIleum3_DOT_iML]
8000
6000
4000
2000
0
0
100
200
300
400
500
600
700
time [day]

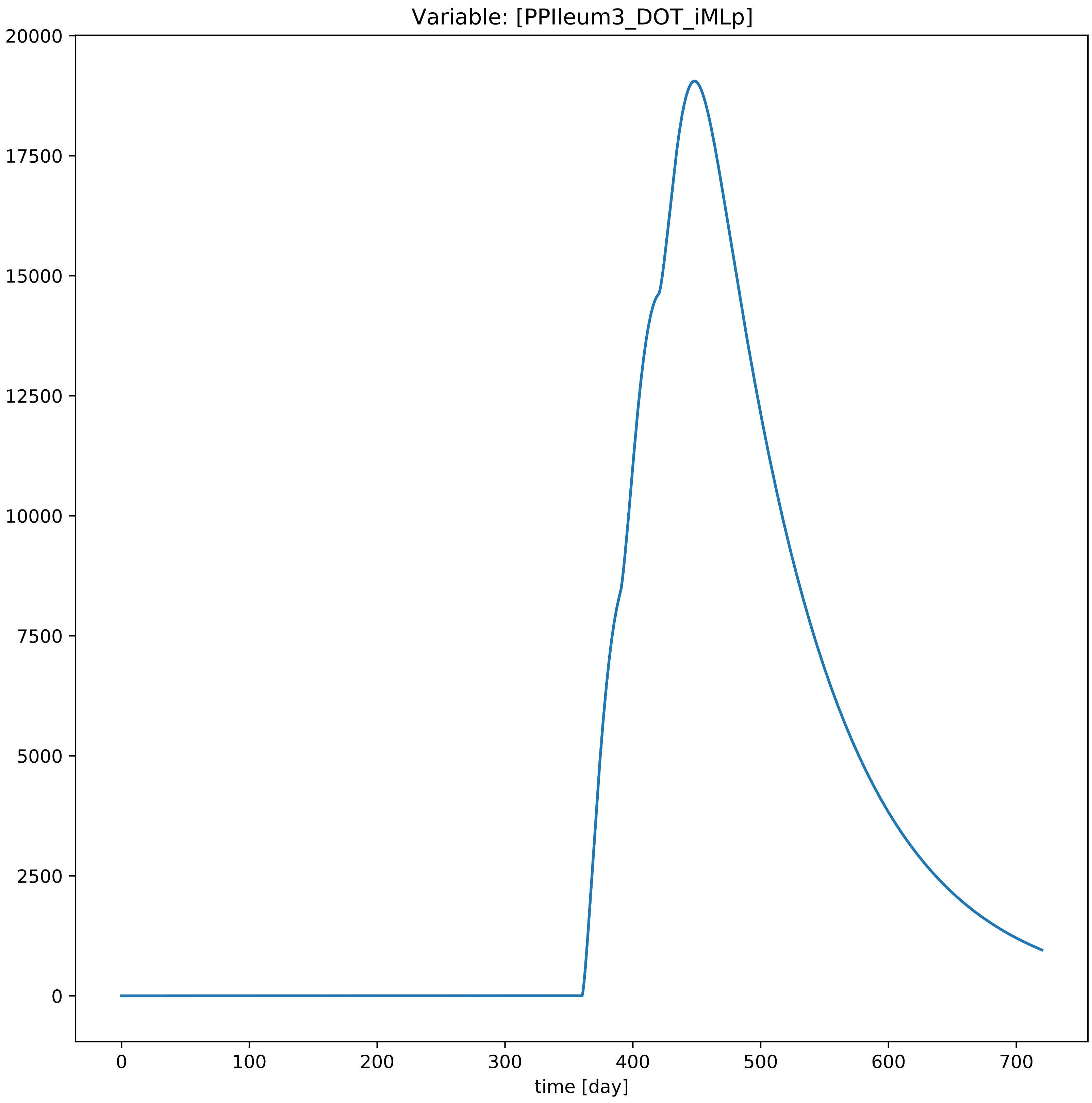
Variable: [PPIleum3_DOT_iMLp]
20000
17500
15000
12500
10000
7500
5000
2500
0
0
100
200
300
400
500
600
700
time [day]

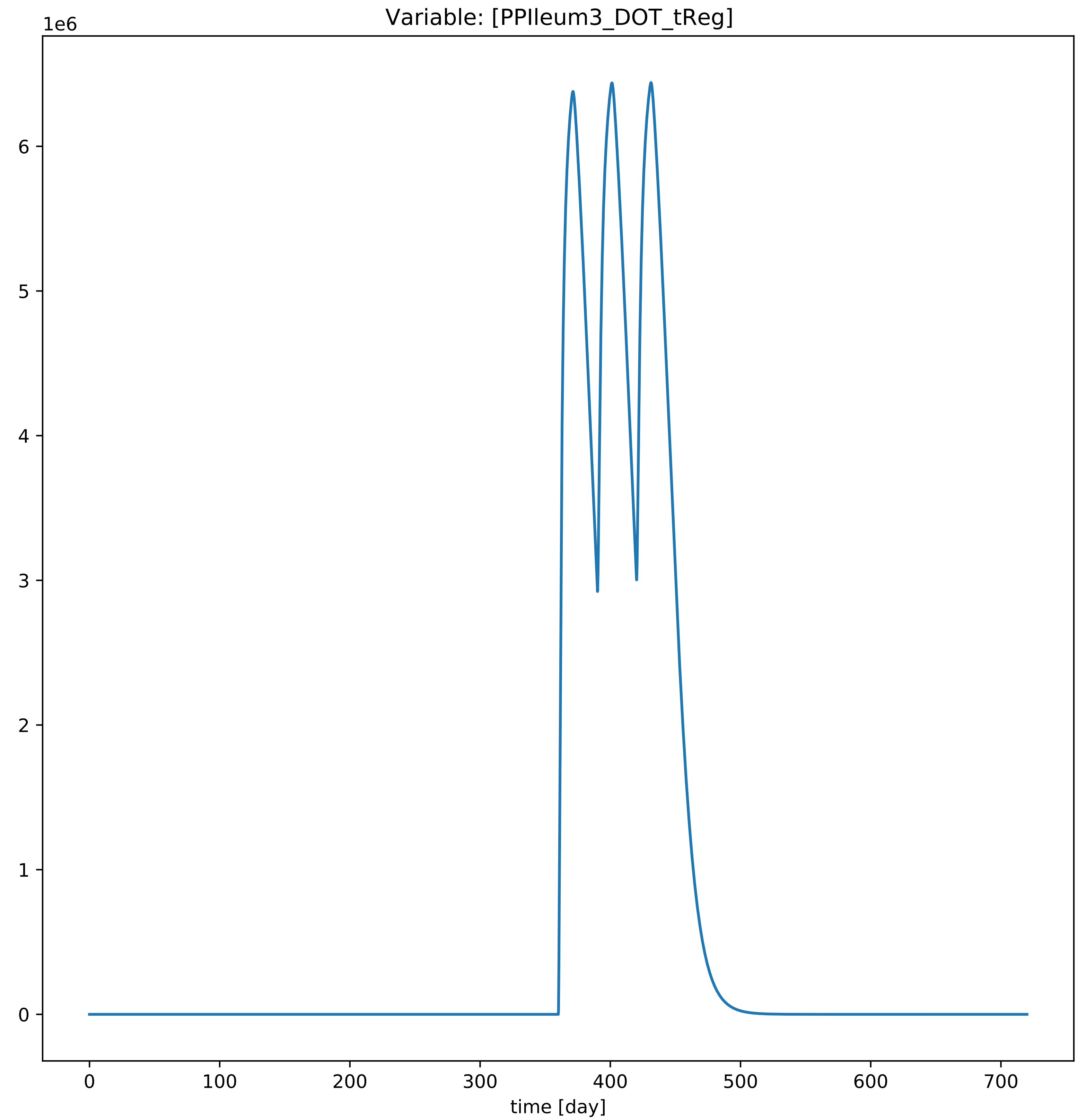

Variable: [PPIleum3_DOT_tReg]
1e6
0
1
2
3
4
5
6
0
100
200
300
400
500
600
700
time [day]

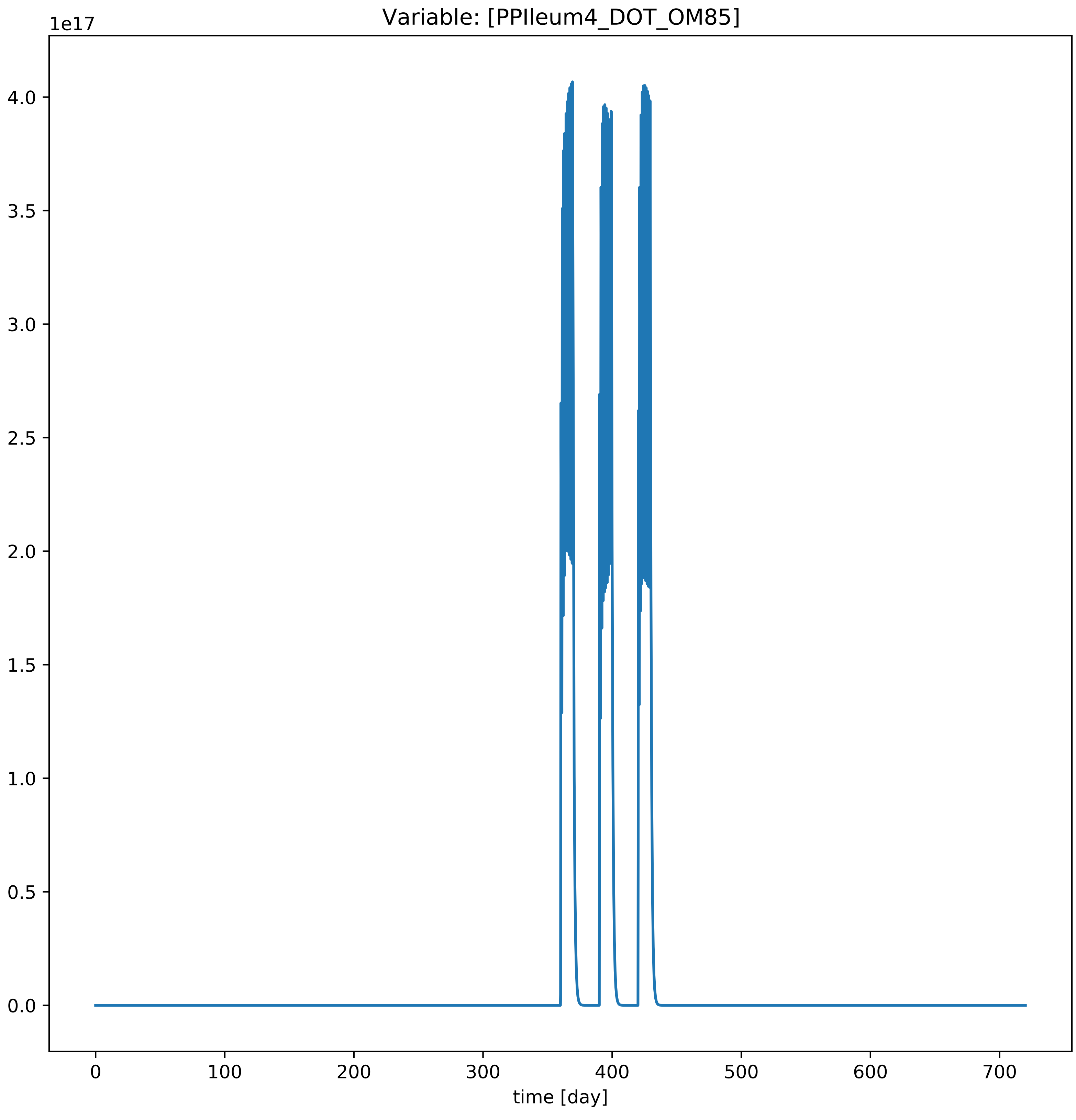

Variable: [PPIleum4_DOT_OM85]
1e17
4.0
3.5
3.0
2.5
2.0
1.5
1.0
0.5
0.0
0
100
200
300
400
500
600
700
time [day]

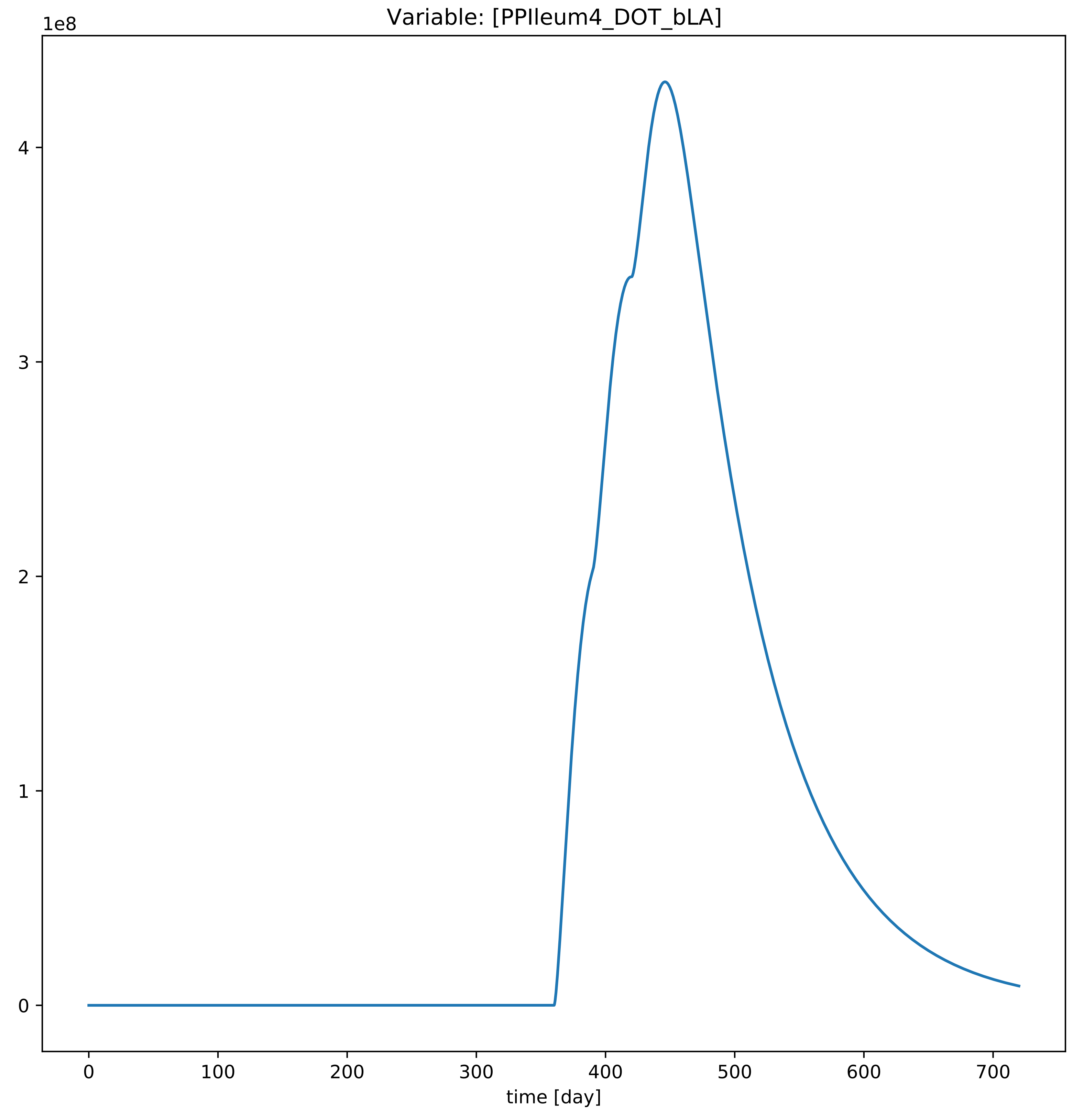
Variable: [PPIleum4_DOT_bLA]
1e8
4
3
2
1
0
0
100
200
300
400
500
600
700
time [day]

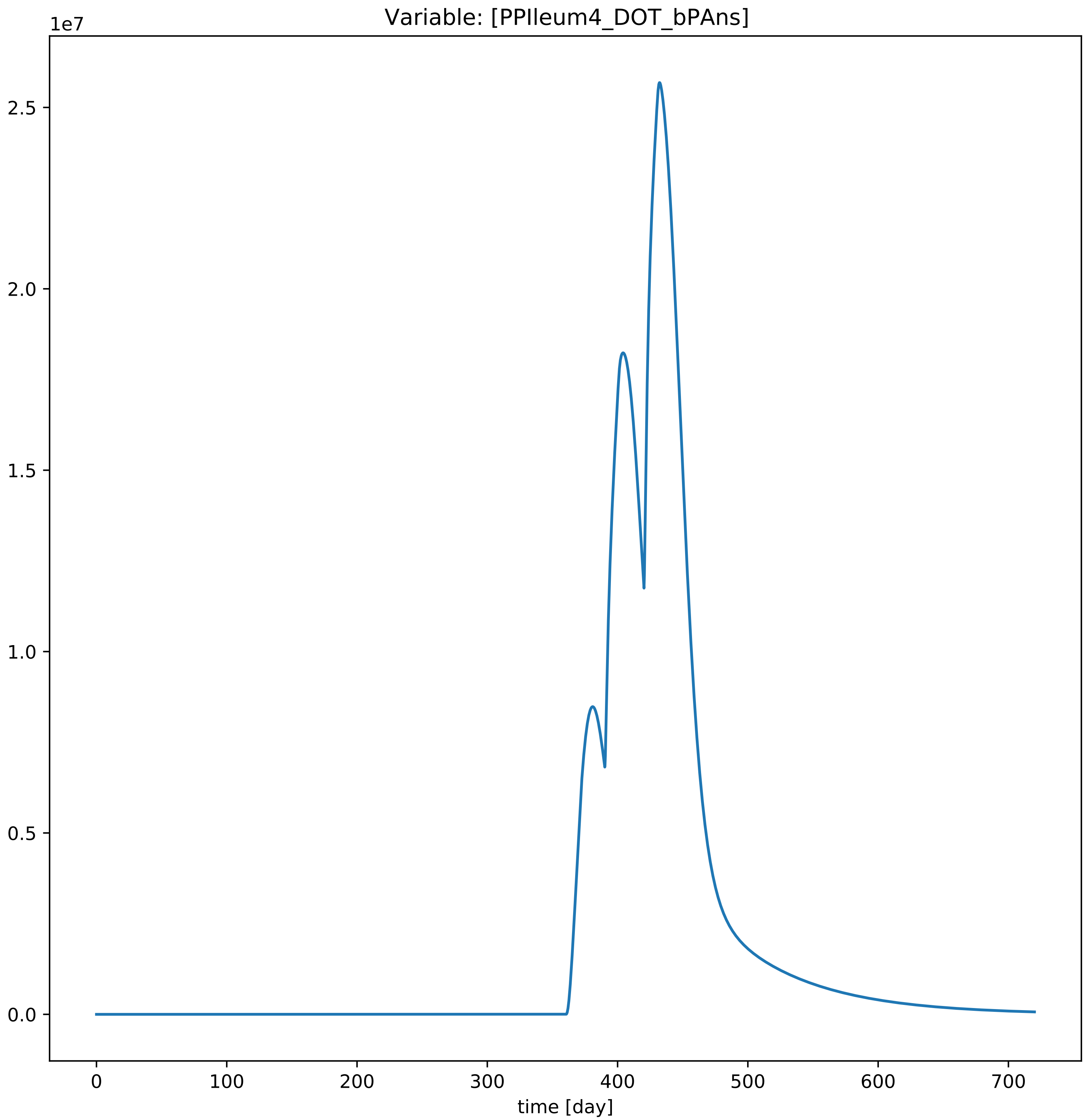

Variable: [PPIleum4_DOT_bPAns]
1e7
2.5
2.0
1.5
1.0
0.5
0.0
0
100
200
300
400
500
600
700
time [day]

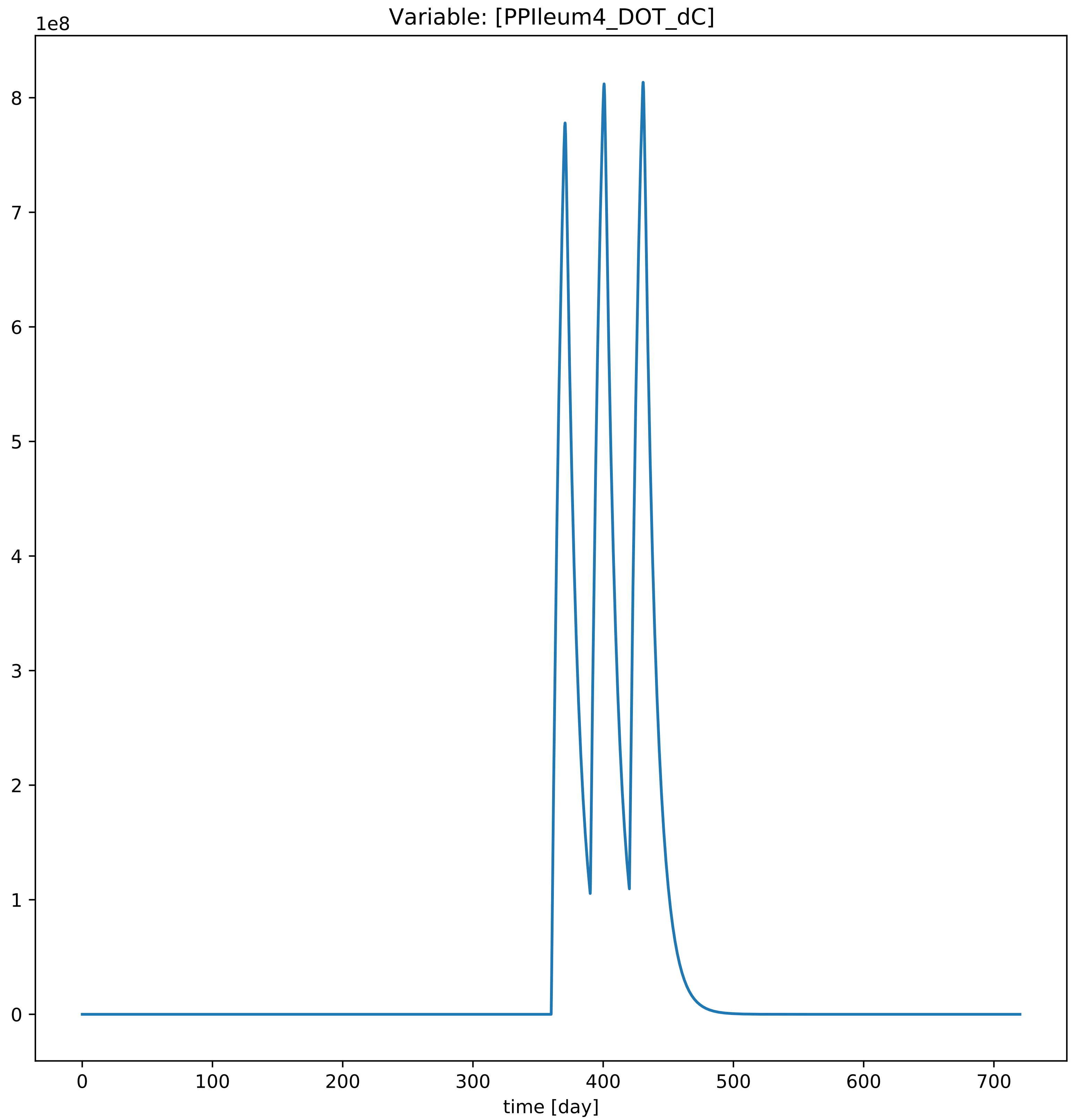

Variable: [PPIleum4_DOT_dC]
1e8
8
7
6
5
4
3
2
1
0
0
100
200
300
400
500
600
700
time [day]

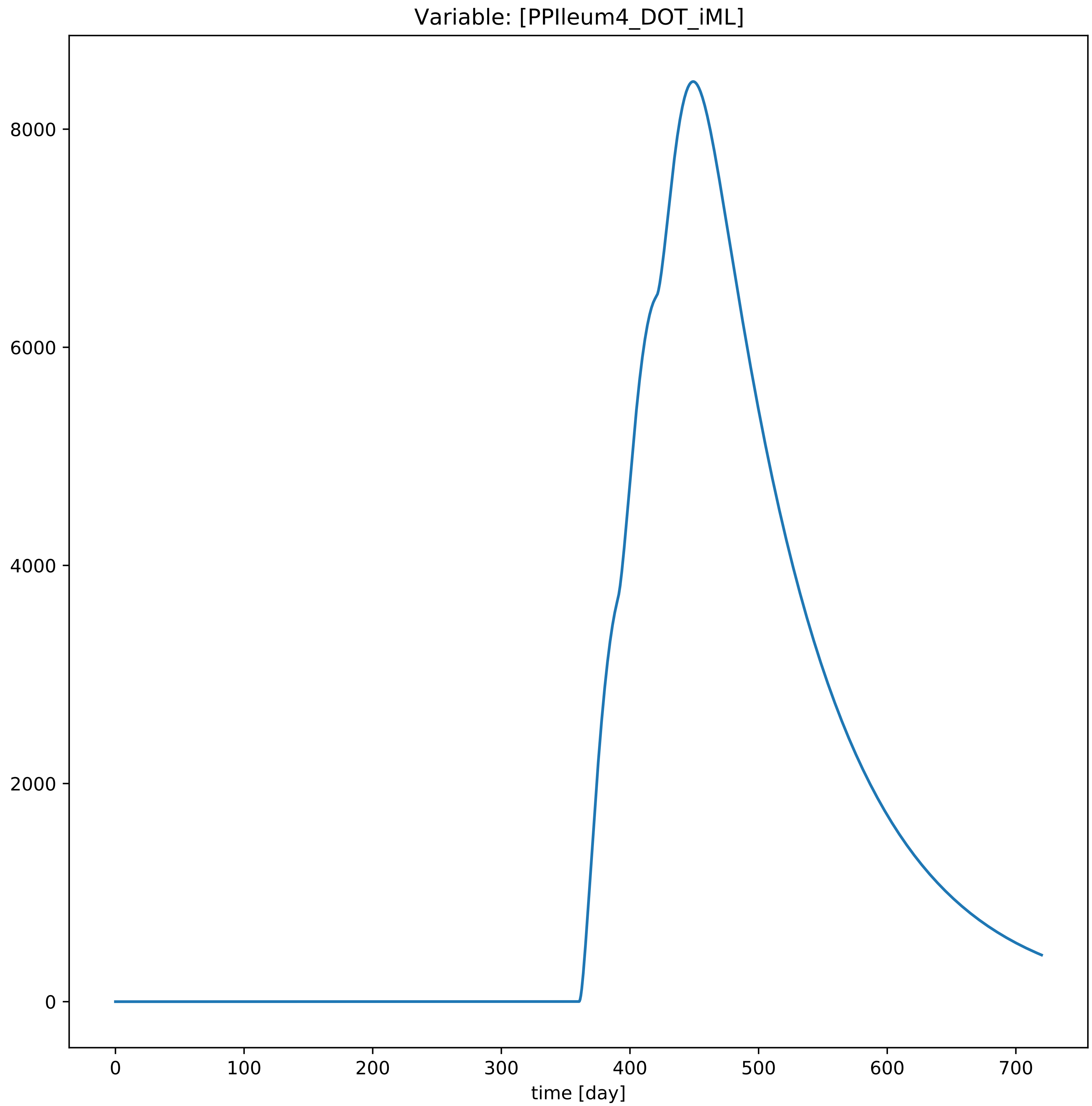
Variable: [PPIleum4_DOT_iML]
8000
6000
4000
2000
0
0
100
200
300
400
500
600
700
time [day]

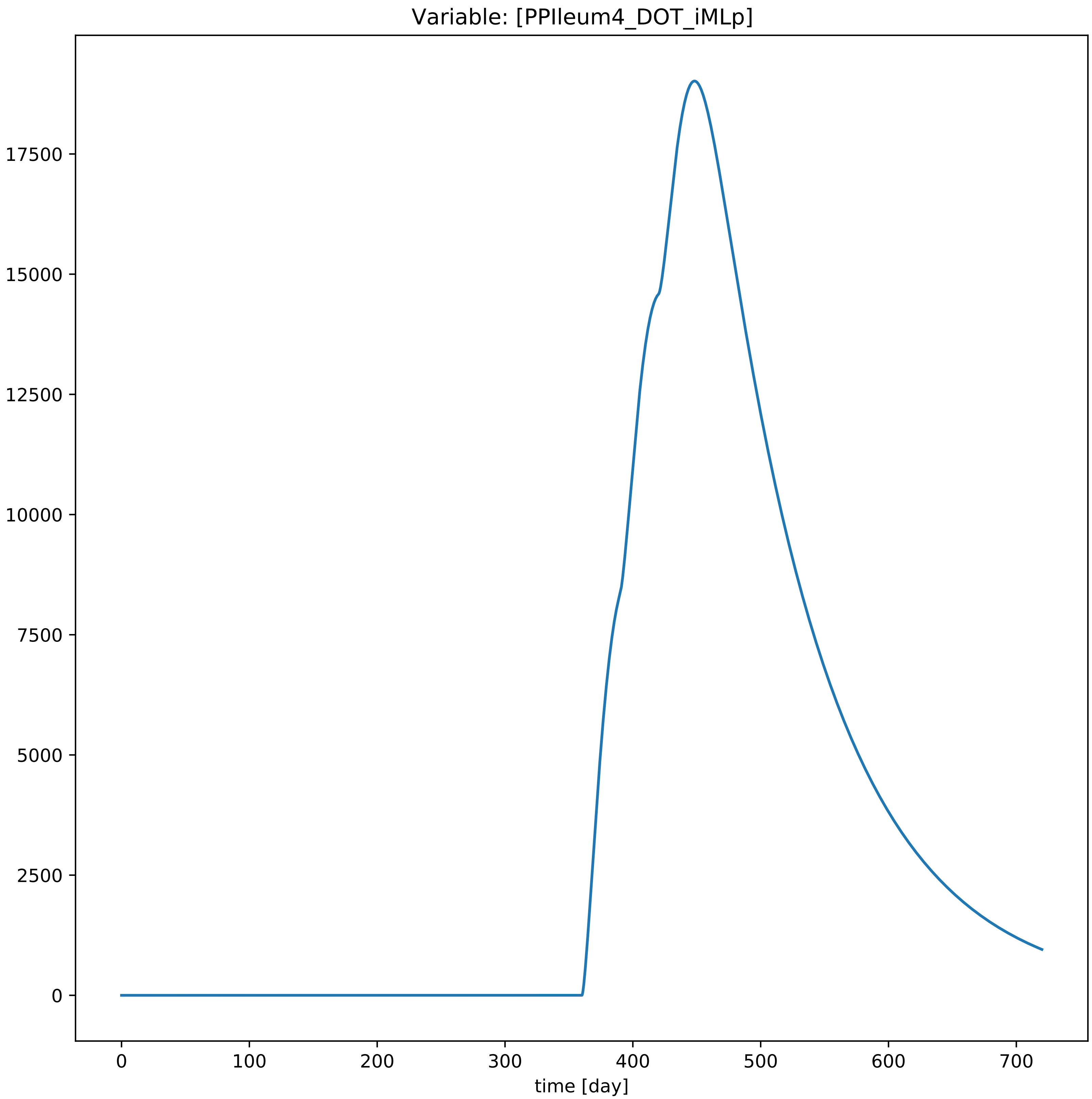

Variable: [PPIleum4_DOT_iMLp]
17500
15000
12500
10000
7500
5000
2500
0
0
100
200
300
400
500
600
700
time [day]

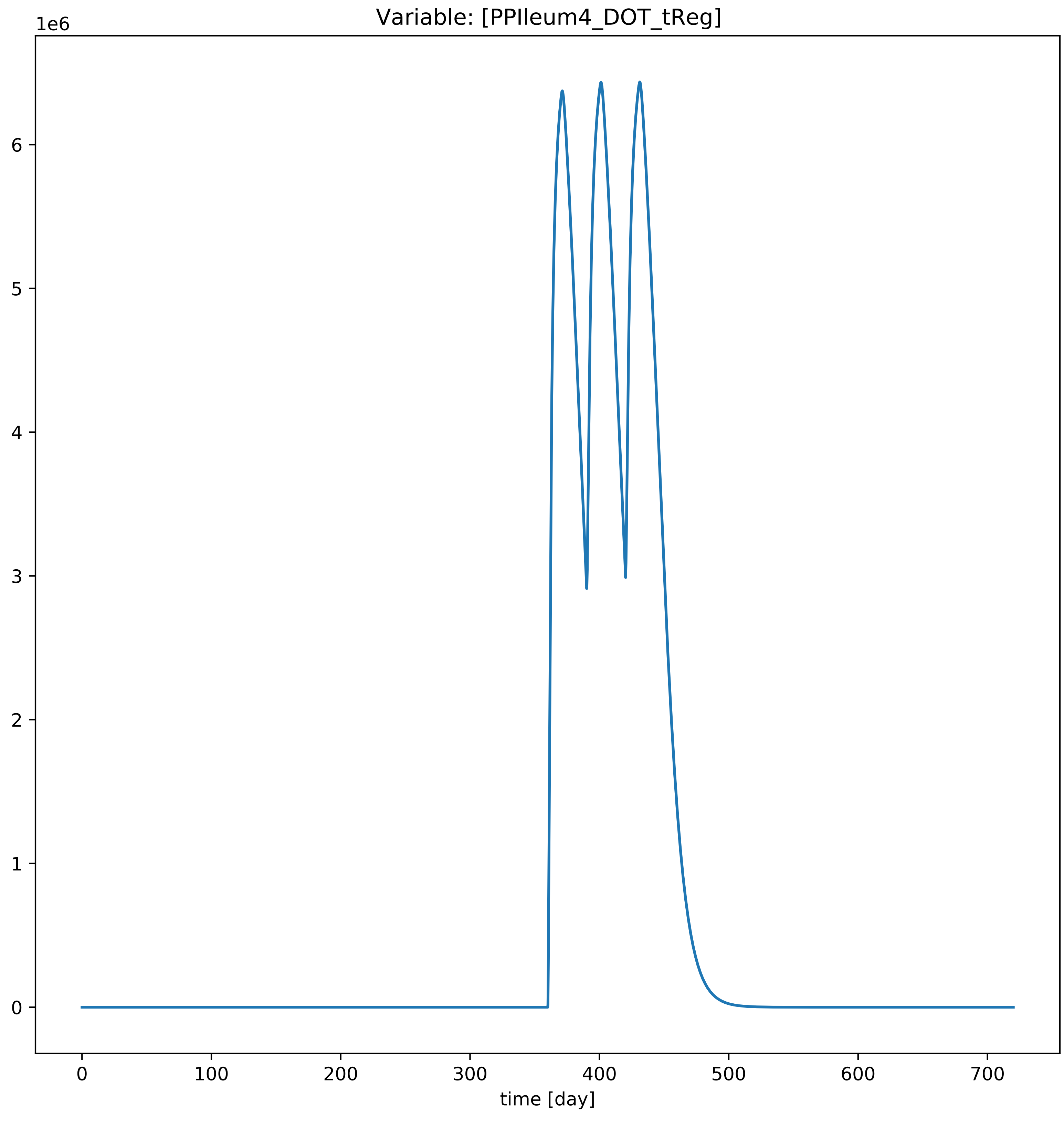

Variable: [PPIleum4_DOT_tReg]
1e6
0
1
2
3
4
5
6
0
100
200
300
400
500
600
700
time [day]

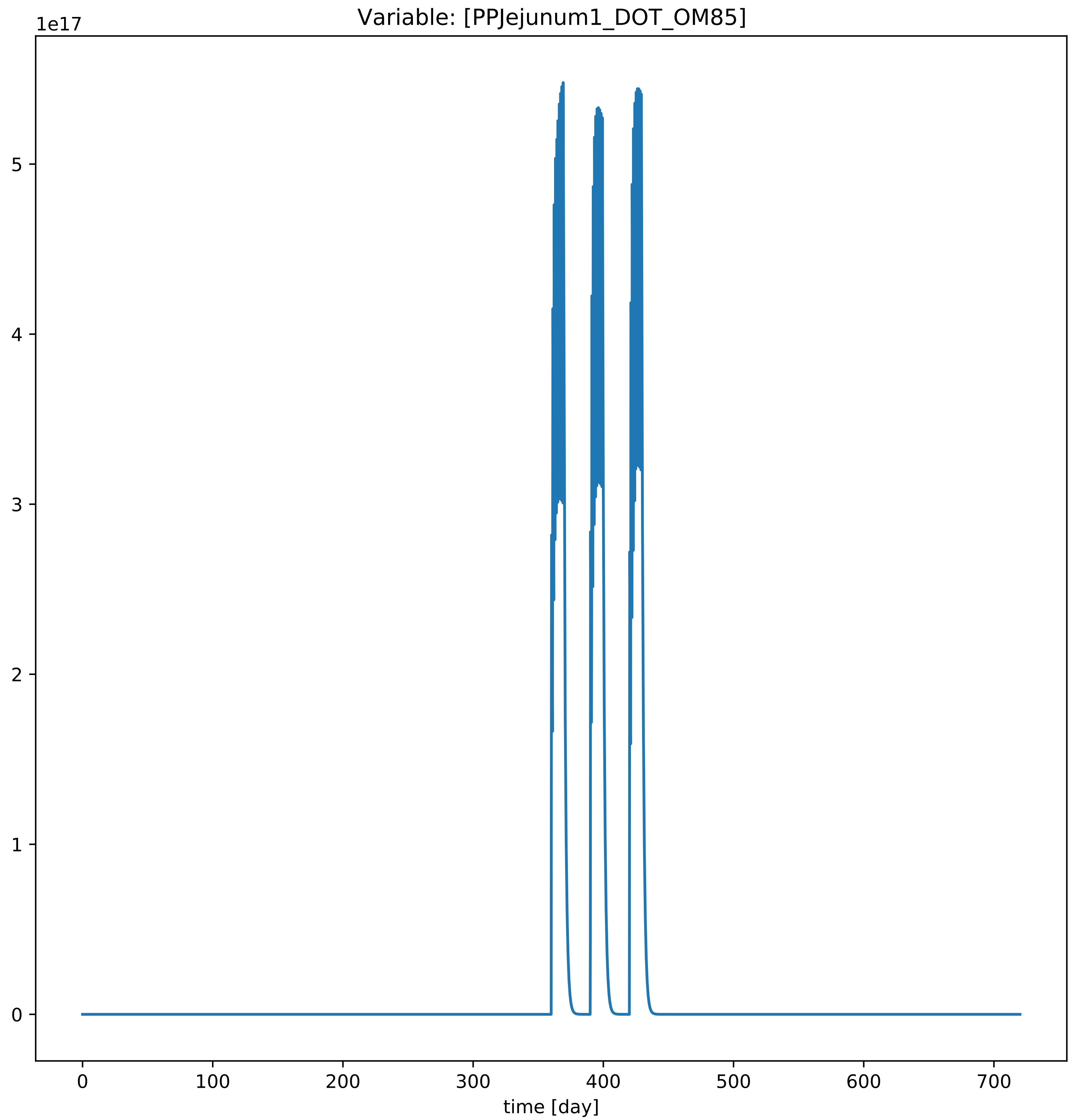

Variable: [PPJejunum1_DOT_OM85]
1e17
0
1
2
3
4
5
0
100
200
300
400
500
600
700
time [day]

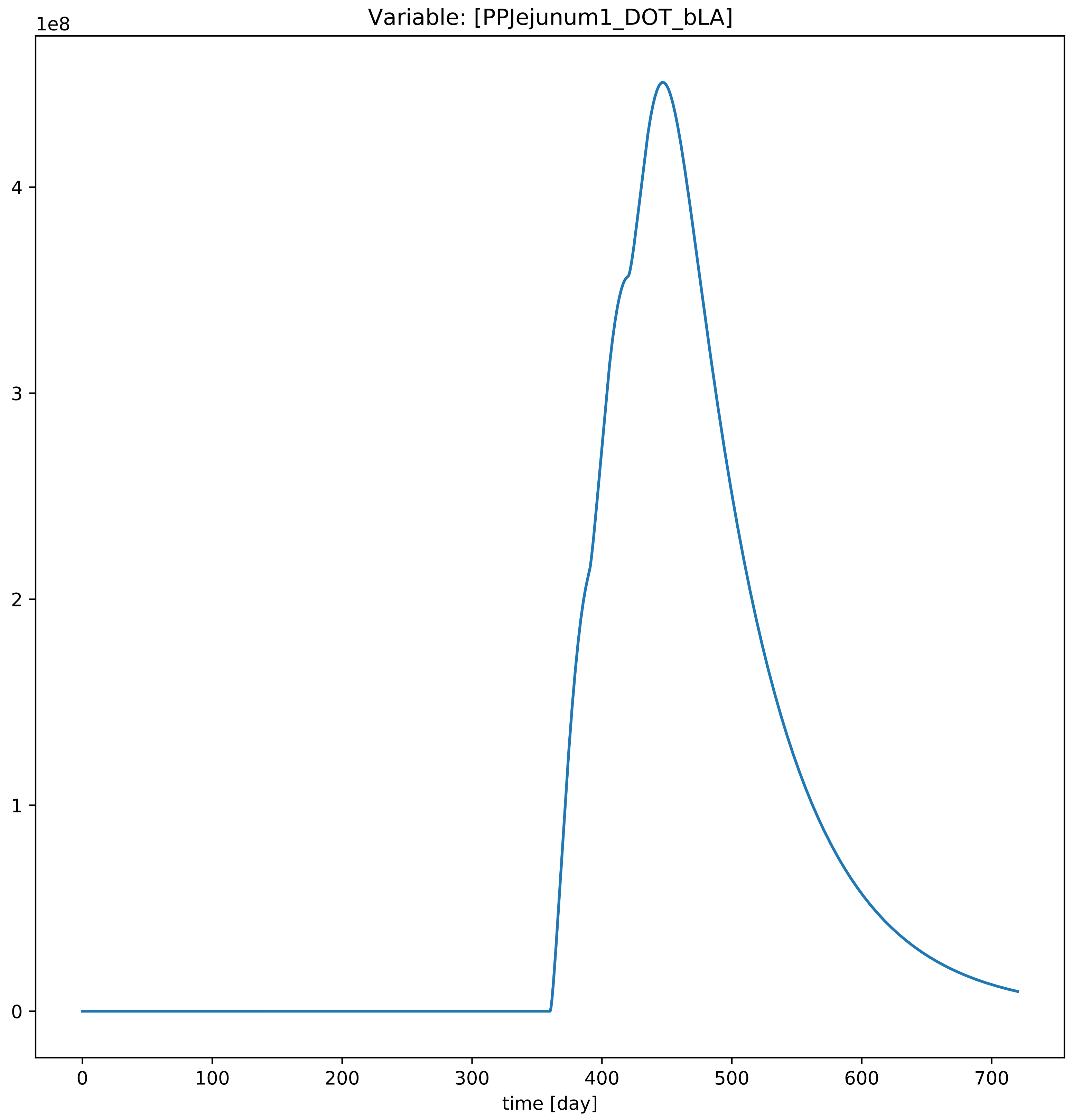

Variable: [PPJejunum1_DOT_bLA]
1e8
4
3
2
1
0
0
100
200
300
400
500
600
700
time [day]

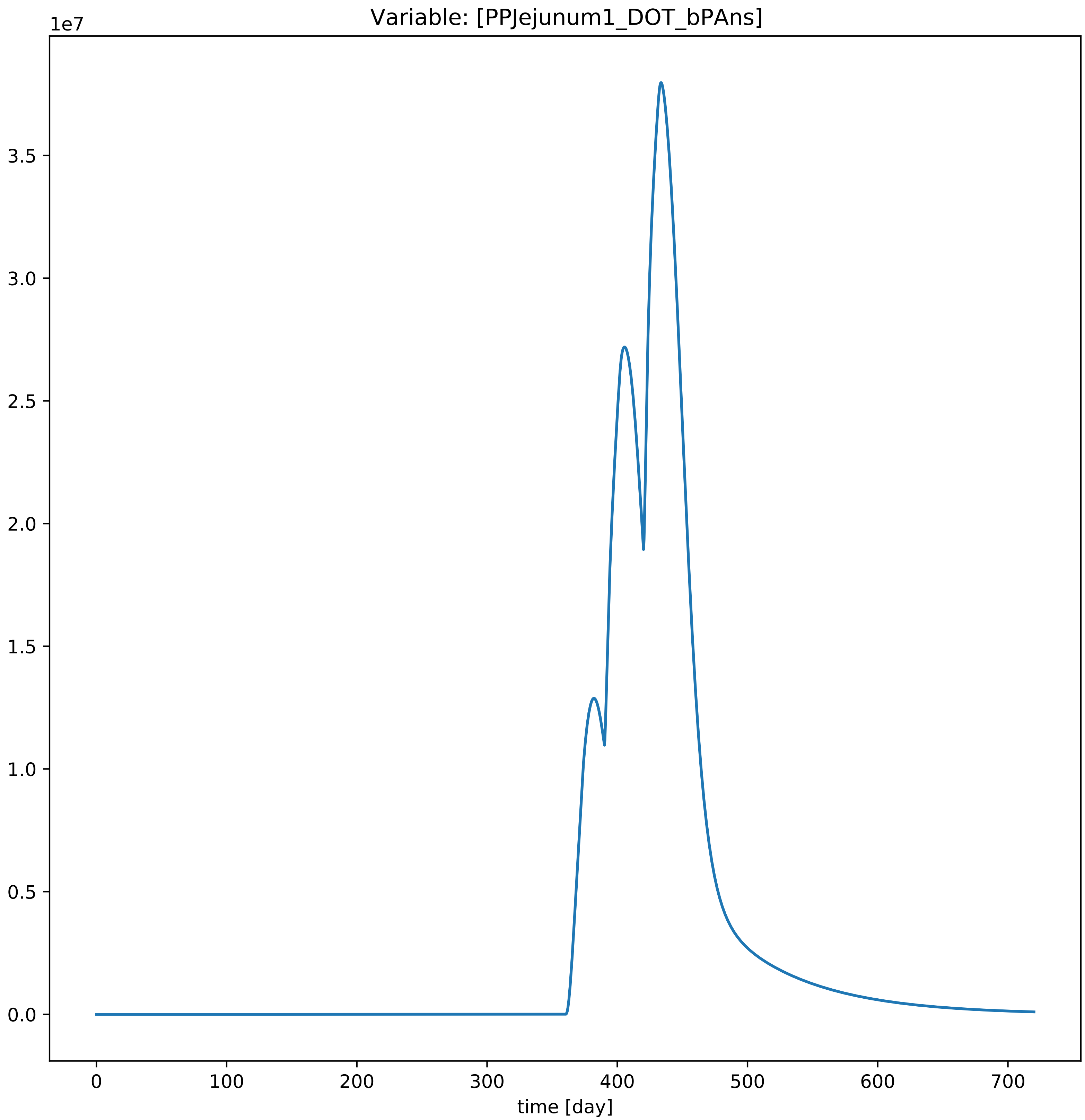

Variable: [PPJejunum1_DOT_bPAns]
1e7
3.5
3.0
2.5
2.0
1.5
1.0
0.5
0.0
0
100
200
300
400
500
600
700
time [day]

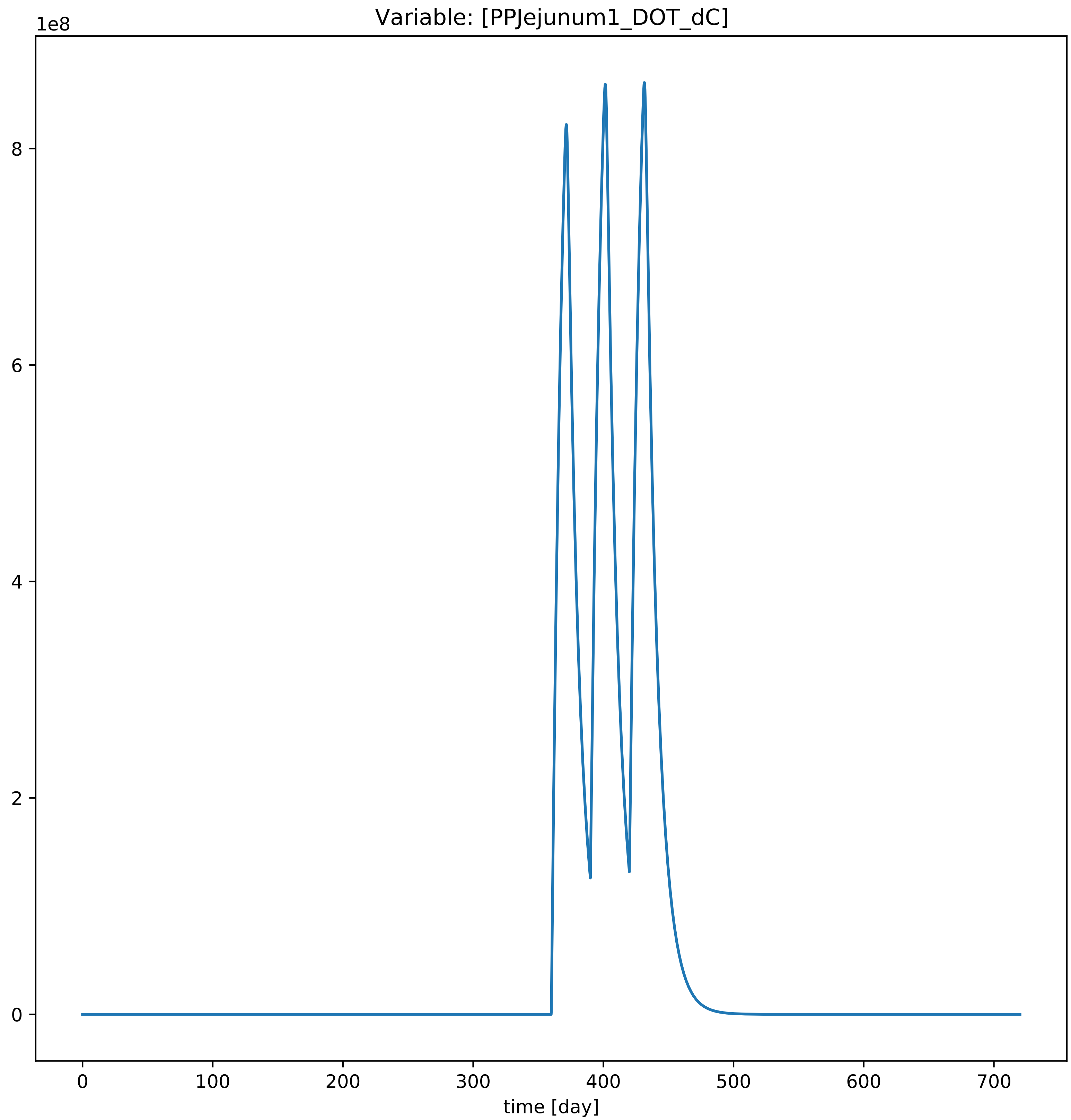

Variable: [PPJejunum1_DOT_dC]
1e8
8
6
4
2
0
0
100
200
300
400
500
600
700
time [day]

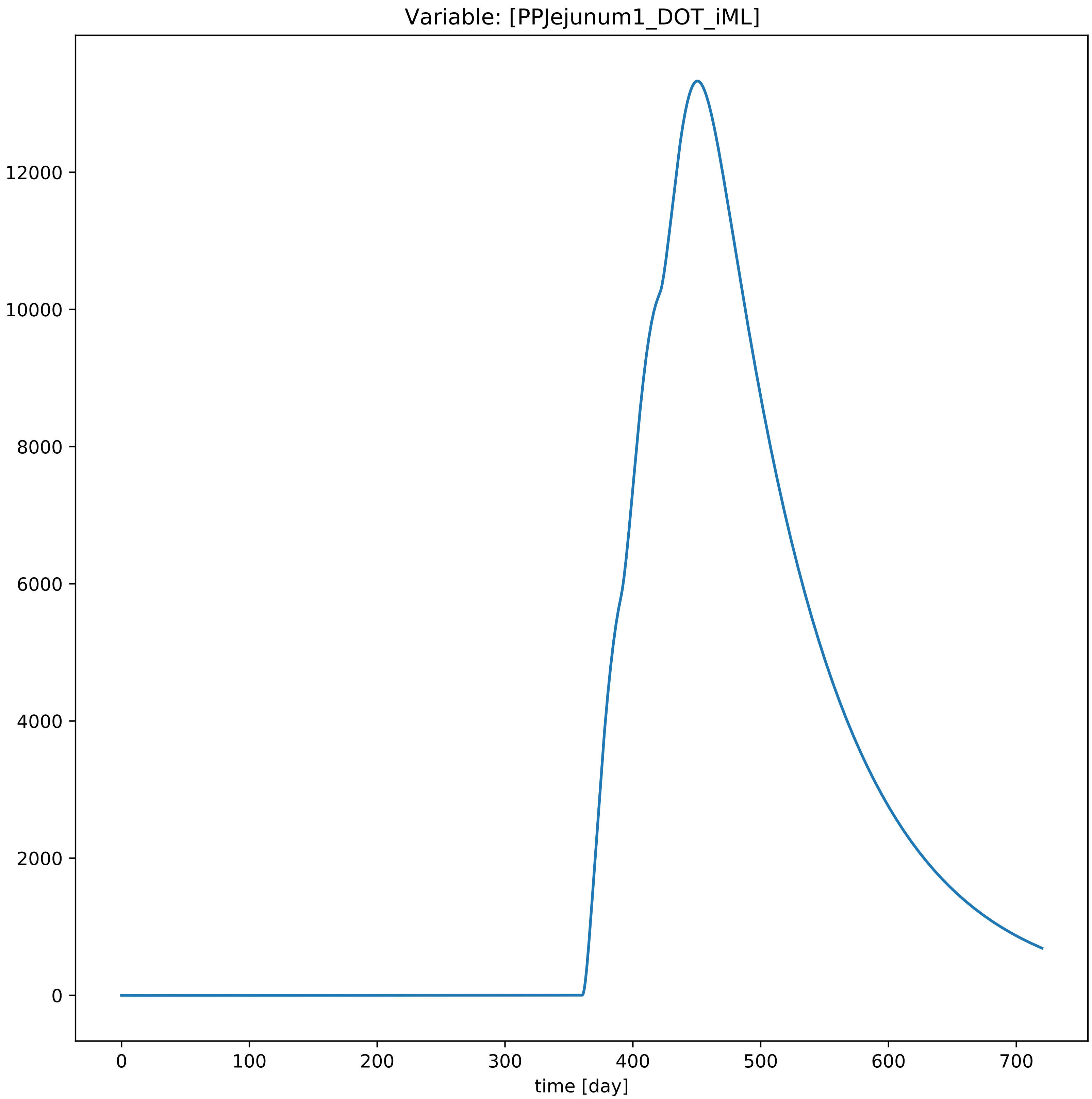

Variable: [PPJejunum1_DOT_iML]
12000
10000
8000
6000
4000
2000
0
0
100
200
300
400
500
600
700
time [day]

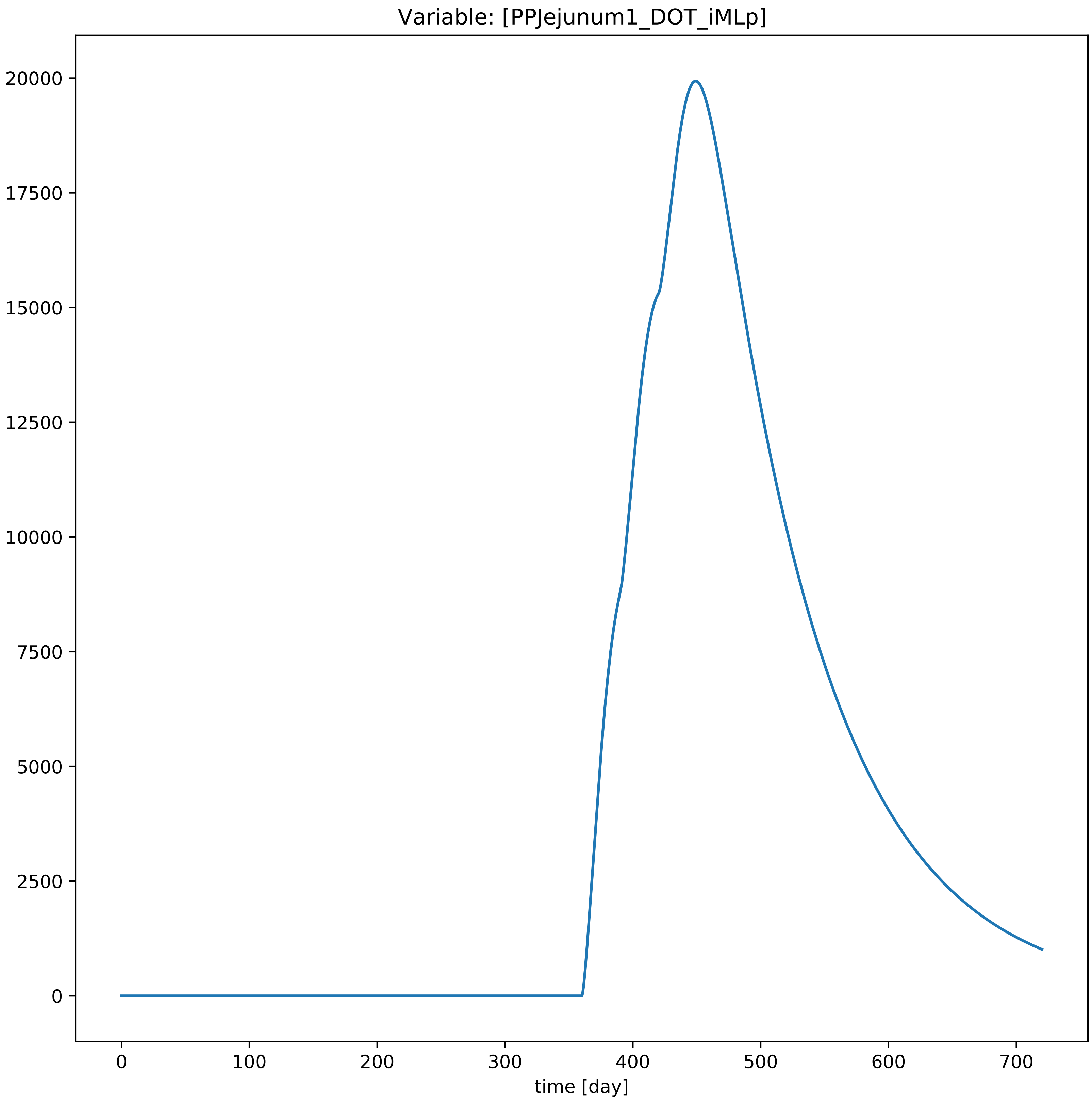

Variable: [PPJejunum1_DOT_iMLp]
20000
17500
15000
12500
10000
7500
5000
2500
0
0
100
200
300
400
500
600
700
time [day]

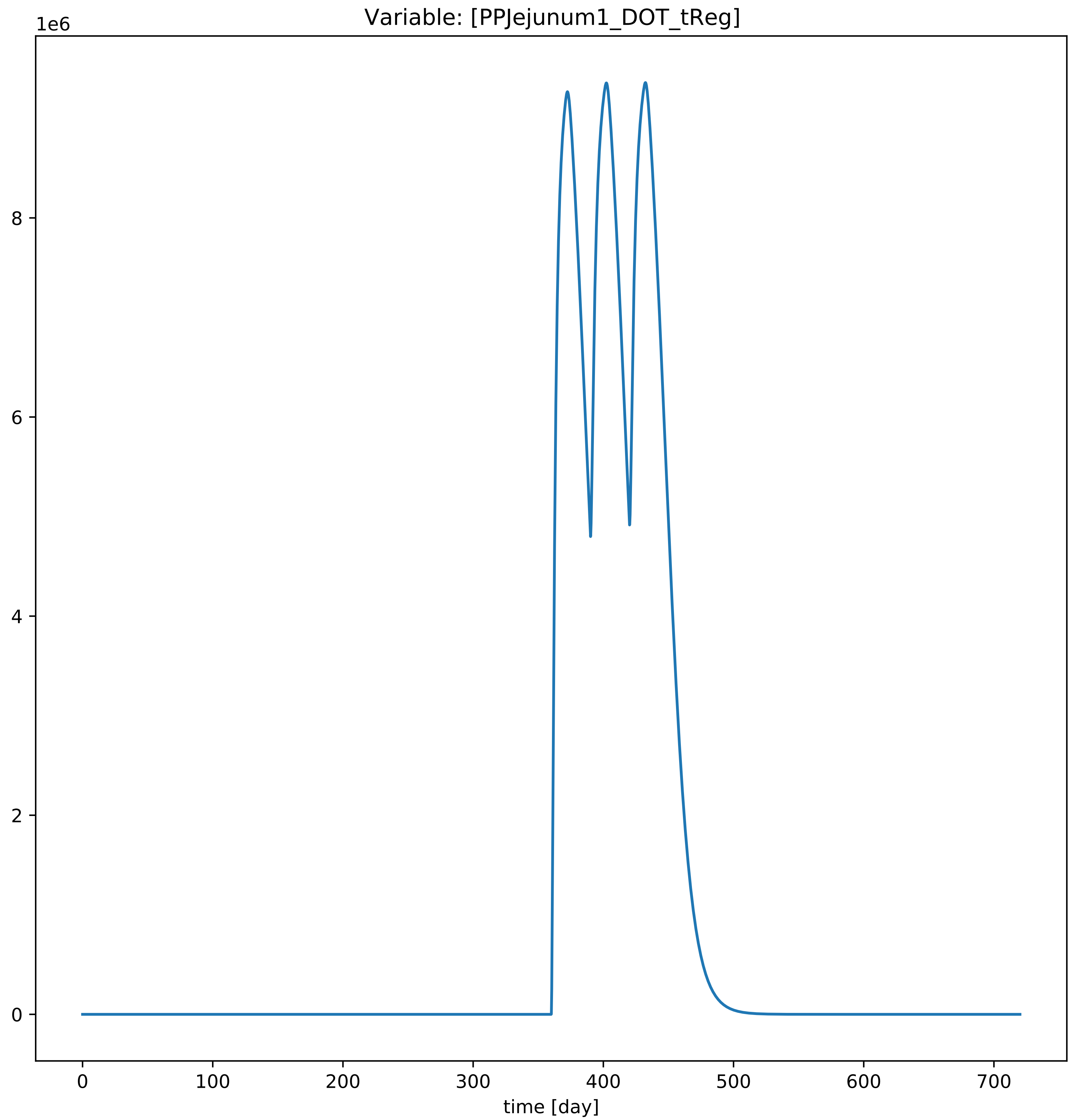

Variable: [PPJejunum1_DOT_tReg]
1e6
8
6
4
2
0
0
100
200
300
400
500
600
700
time [day]

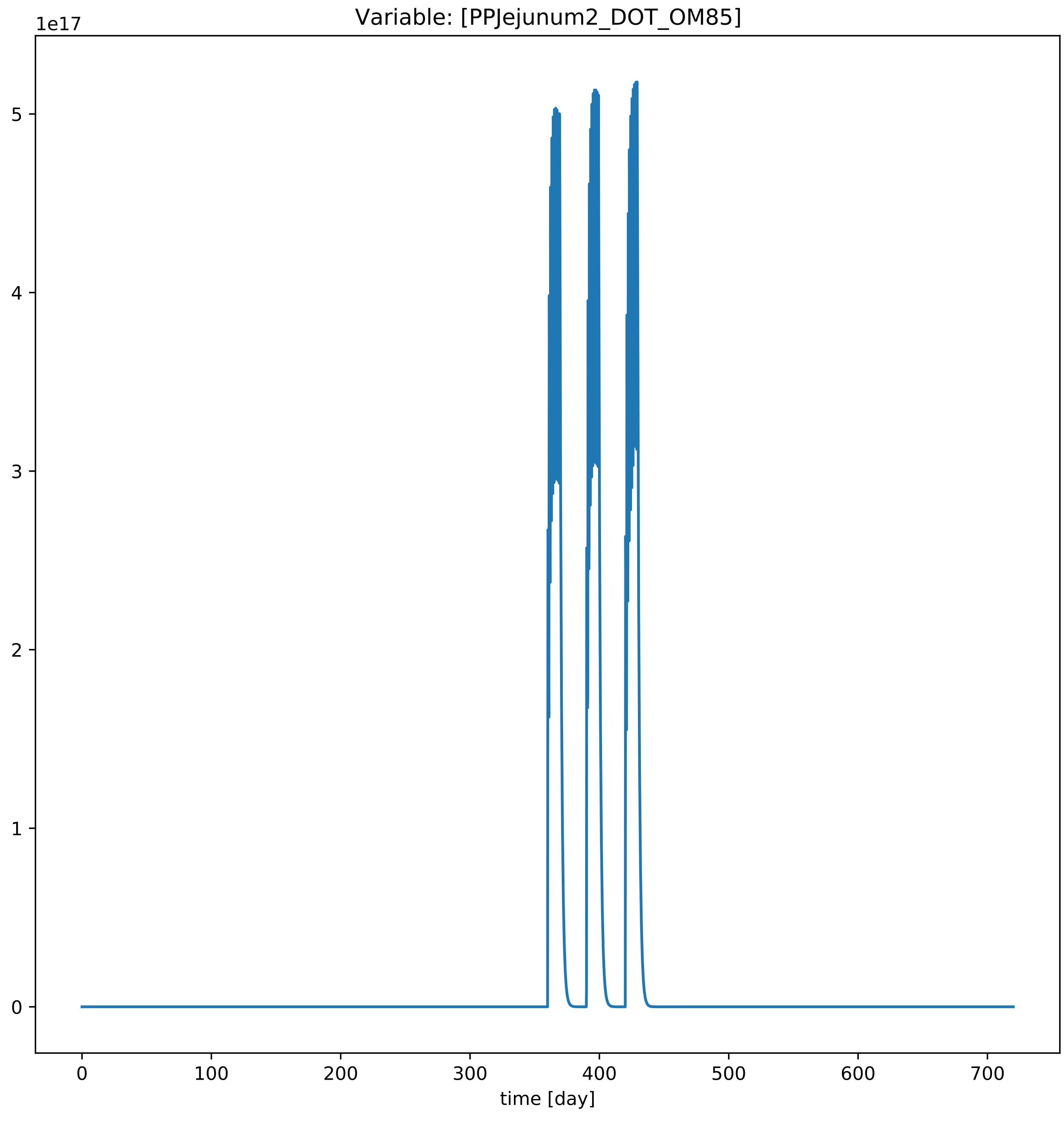

Variable: [PPJejunum2_DOT_OM85]
1e17
0
1
2
3
4
5
0
100
200
300
400
500
600
700
time [day]

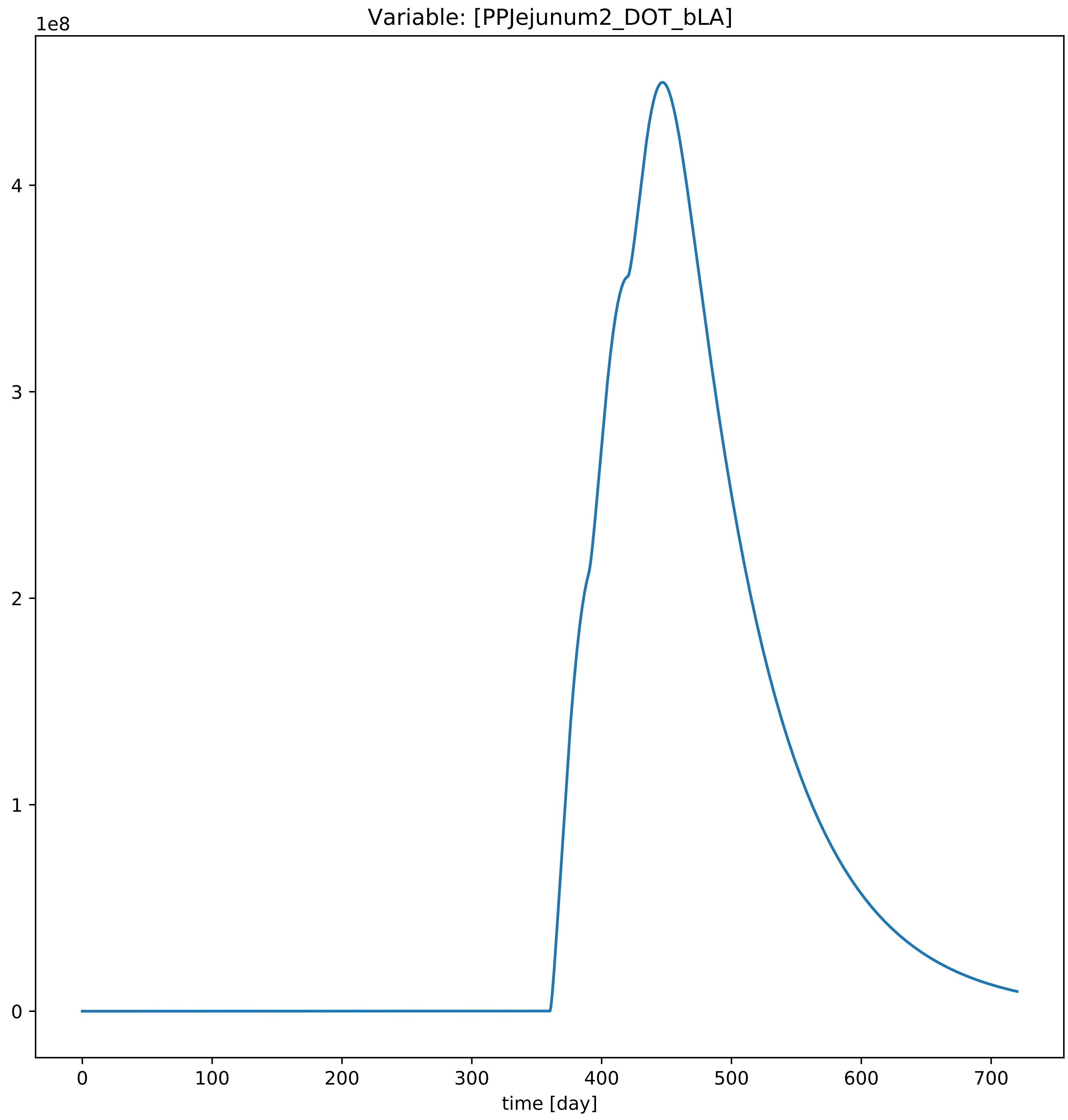
Variable: [PPJejunum2_DOT_bLA]
1e8
4
3
2
1
0
0
100
200
300
400
500
600
700
time [day]

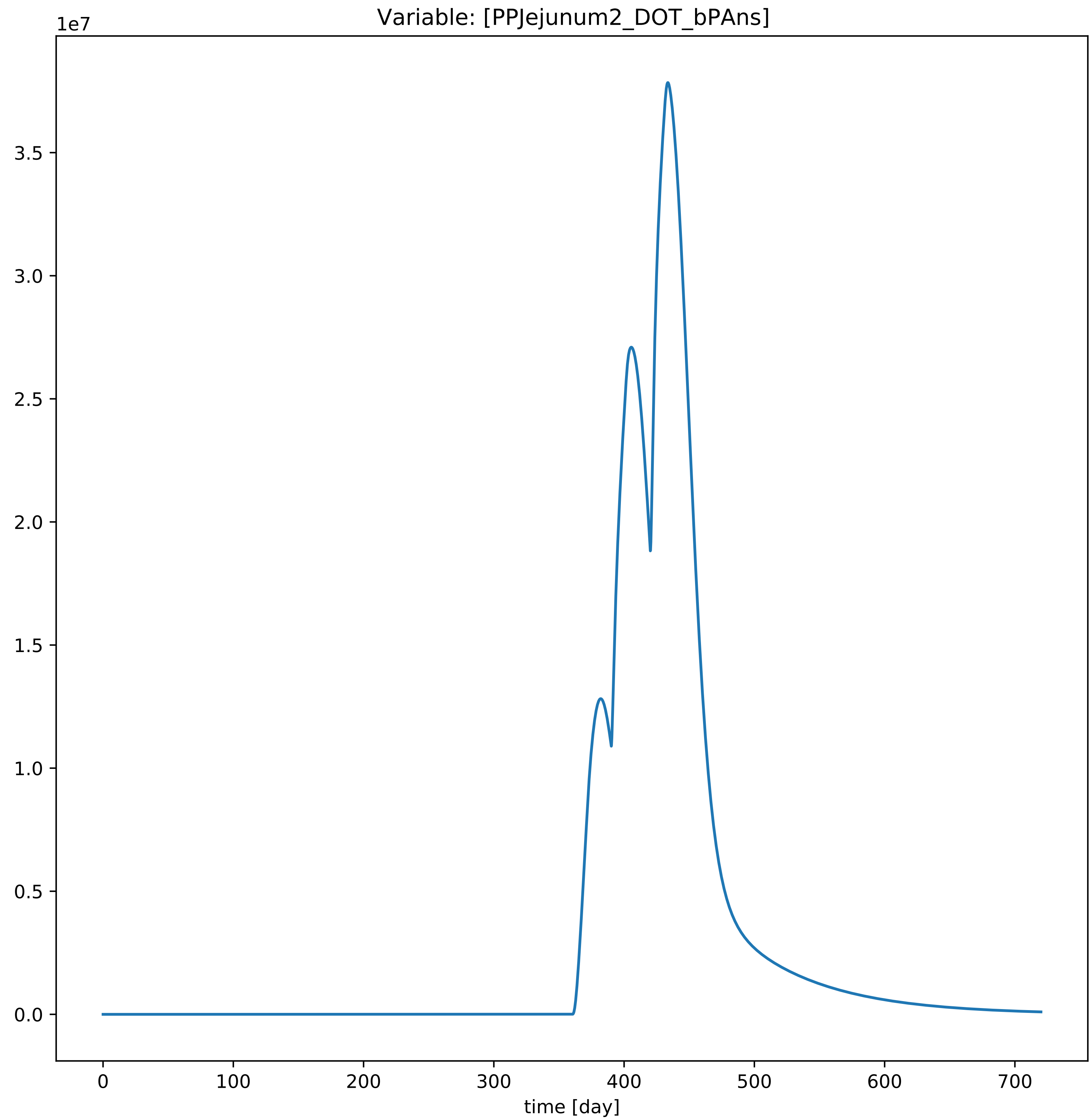

Variable: [PPJejunum2_DOT_bPAns]
1e7
3.5
3.0
2.5
2.0
1.5
1.0
0.5
0.0
0
100
200
300
400
500
600
700
time [day]

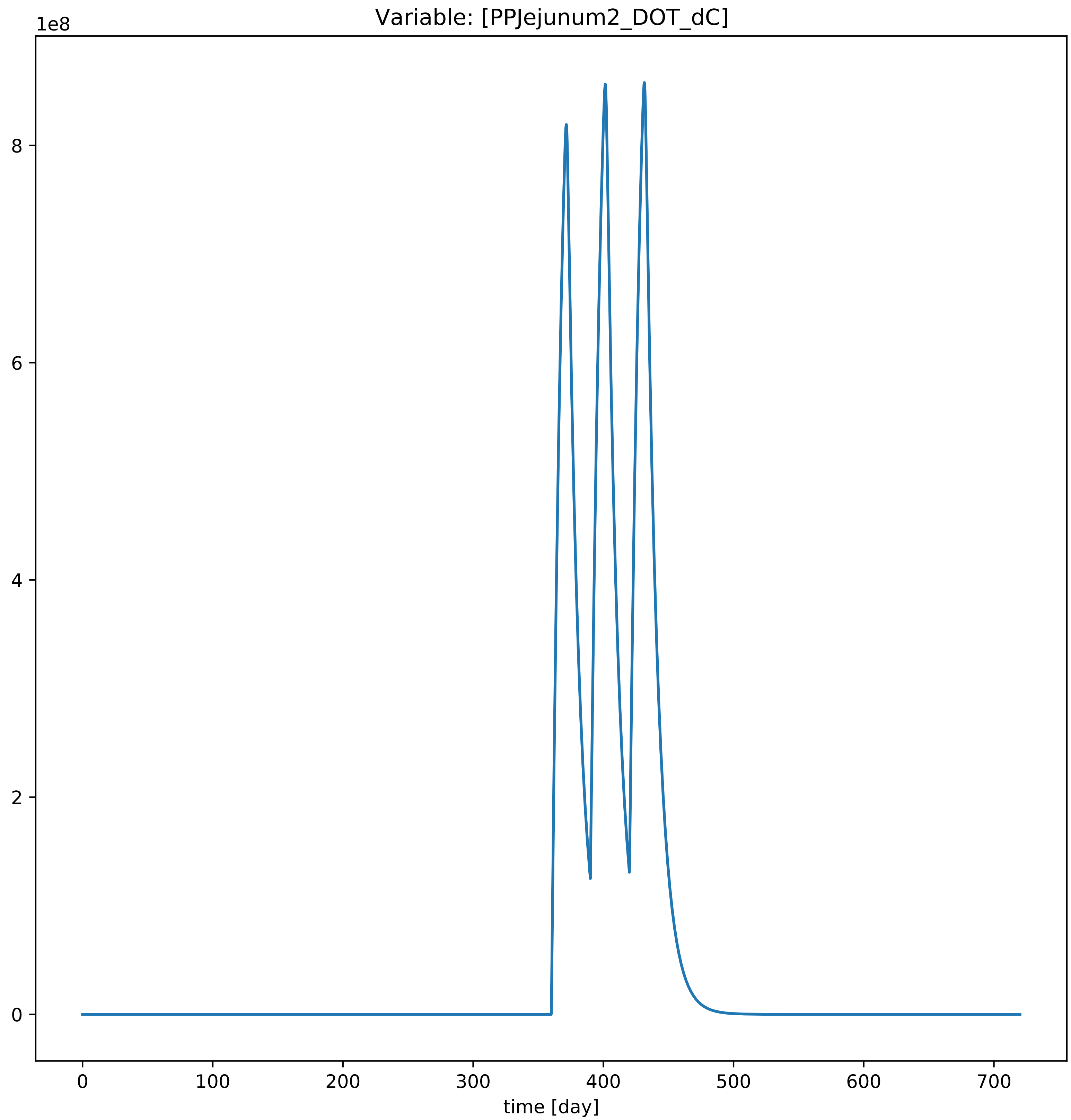
Variable: [PPJejunum2_DOT_dC]
1e8
0
2
4
6
8
0
100
200
300
400
500
600
700
time [day]

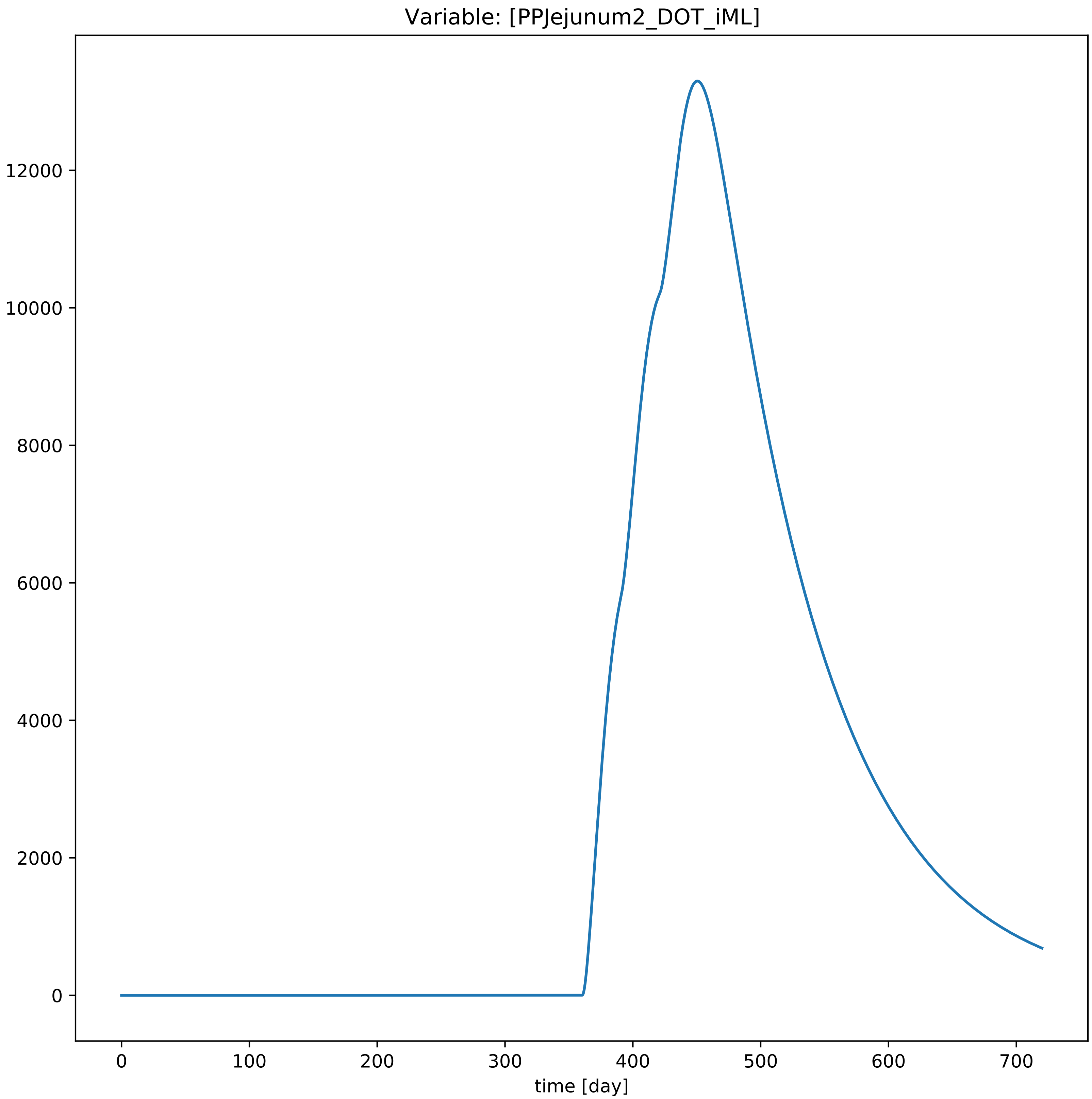
Variable: [PPJejunum2_DOT_iML]
12000
10000
8000
6000
4000
2000
0
0
100
200
300
400
500
600
700
time [day]

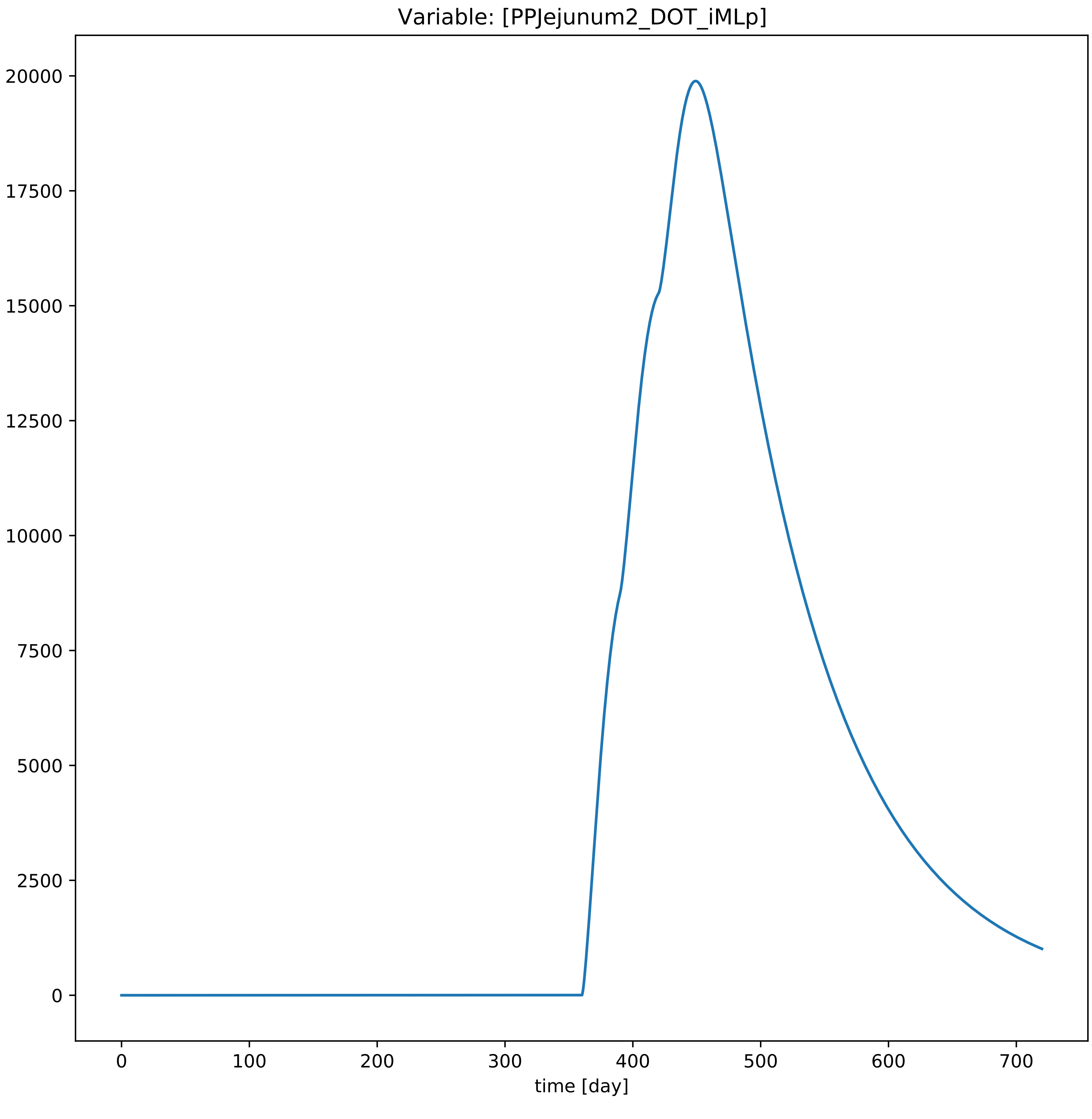
Variable: [PPJejunum2_DOT_iMLp]
20000
17500
15000
12500
10000
7500
5000
2500
0
0
100
200
300
400
500
600
700
time [day]

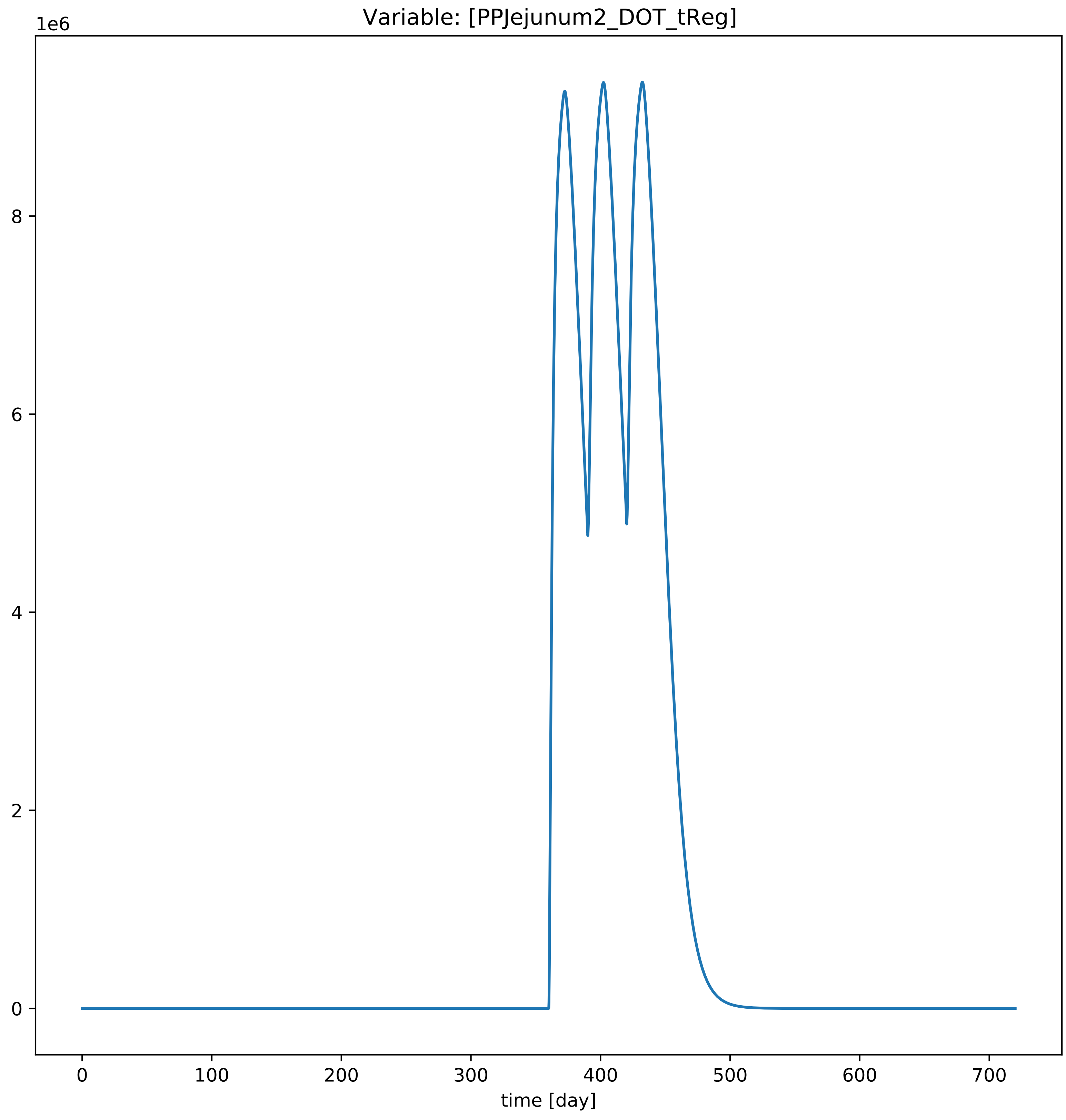
Variable: [PPJejunum2_DOT_tReg]
1e6
8
6
4
2
0
0
100
200
300
400
500
600
700
time [day]

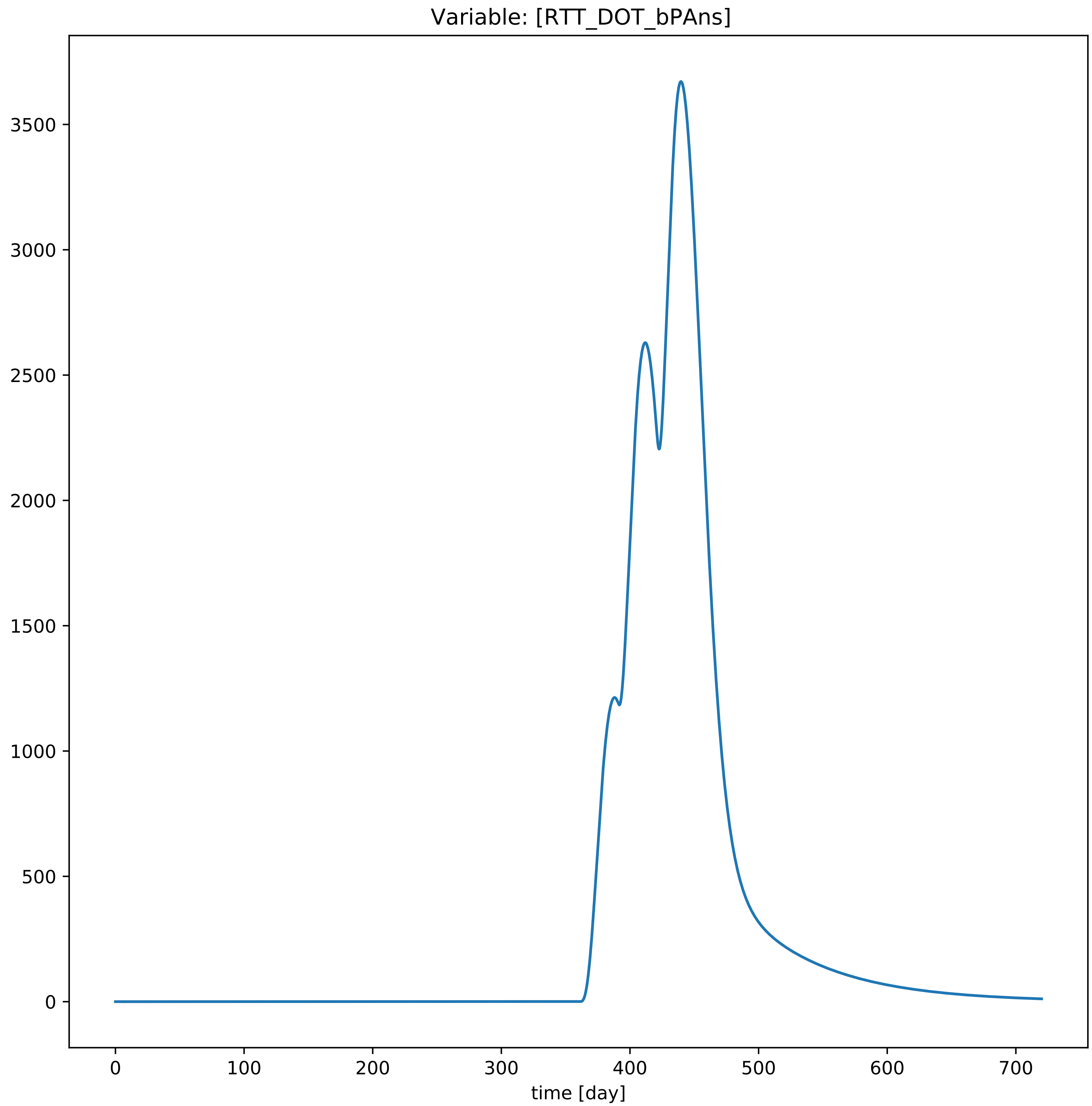

Variable: [RTT_DOT_bPAns]
3500
3000
2500
2000
1500
1000
500
0
0
100
200
300
400
500
600
700
time [day]

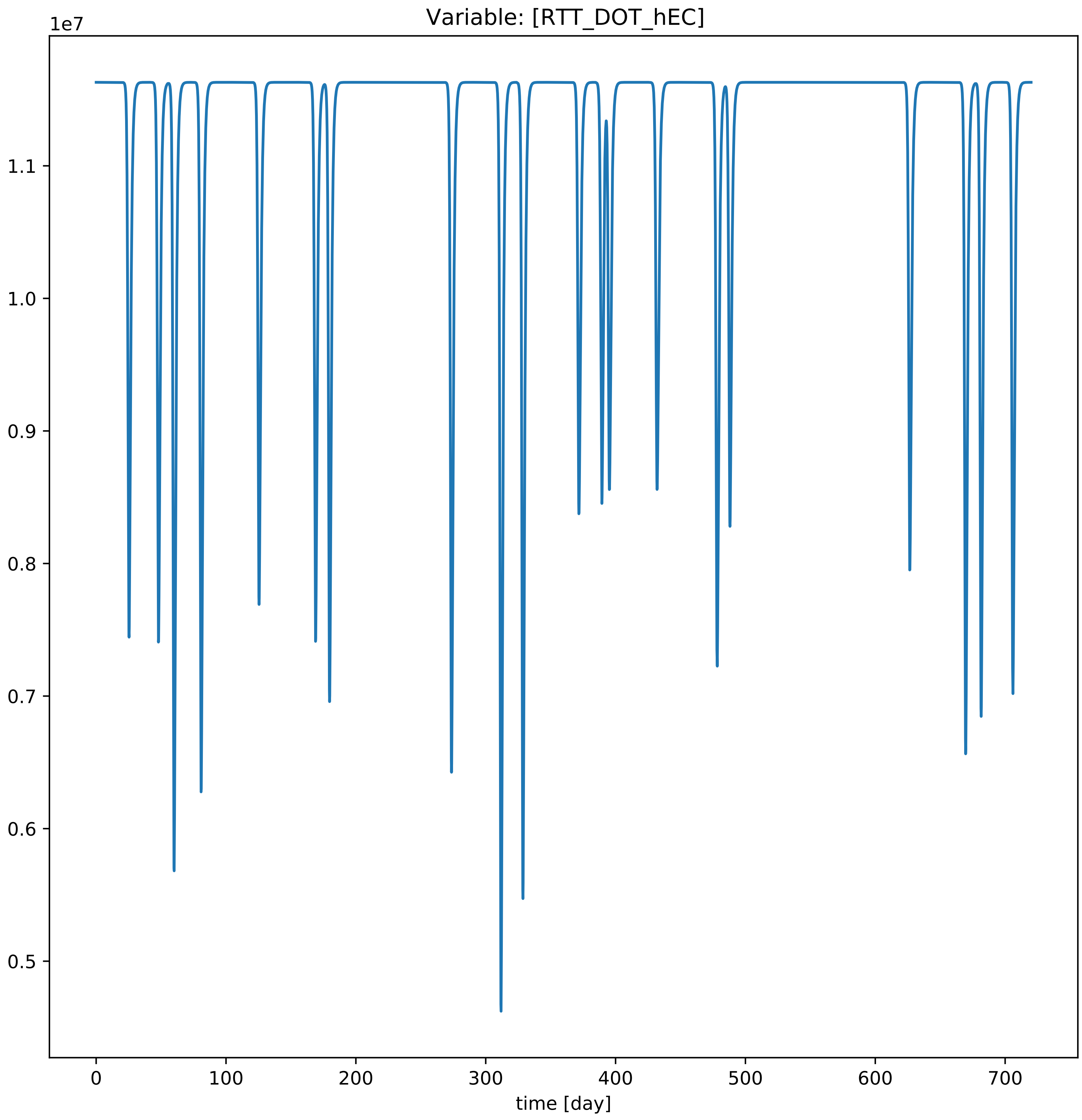

Variable: [RTT_DOT_hEC]
1e7
1.1
1.0
0.9
0.8
0.7
0.6
0.5
0
100
200
300
400
500
600
700
time [day]

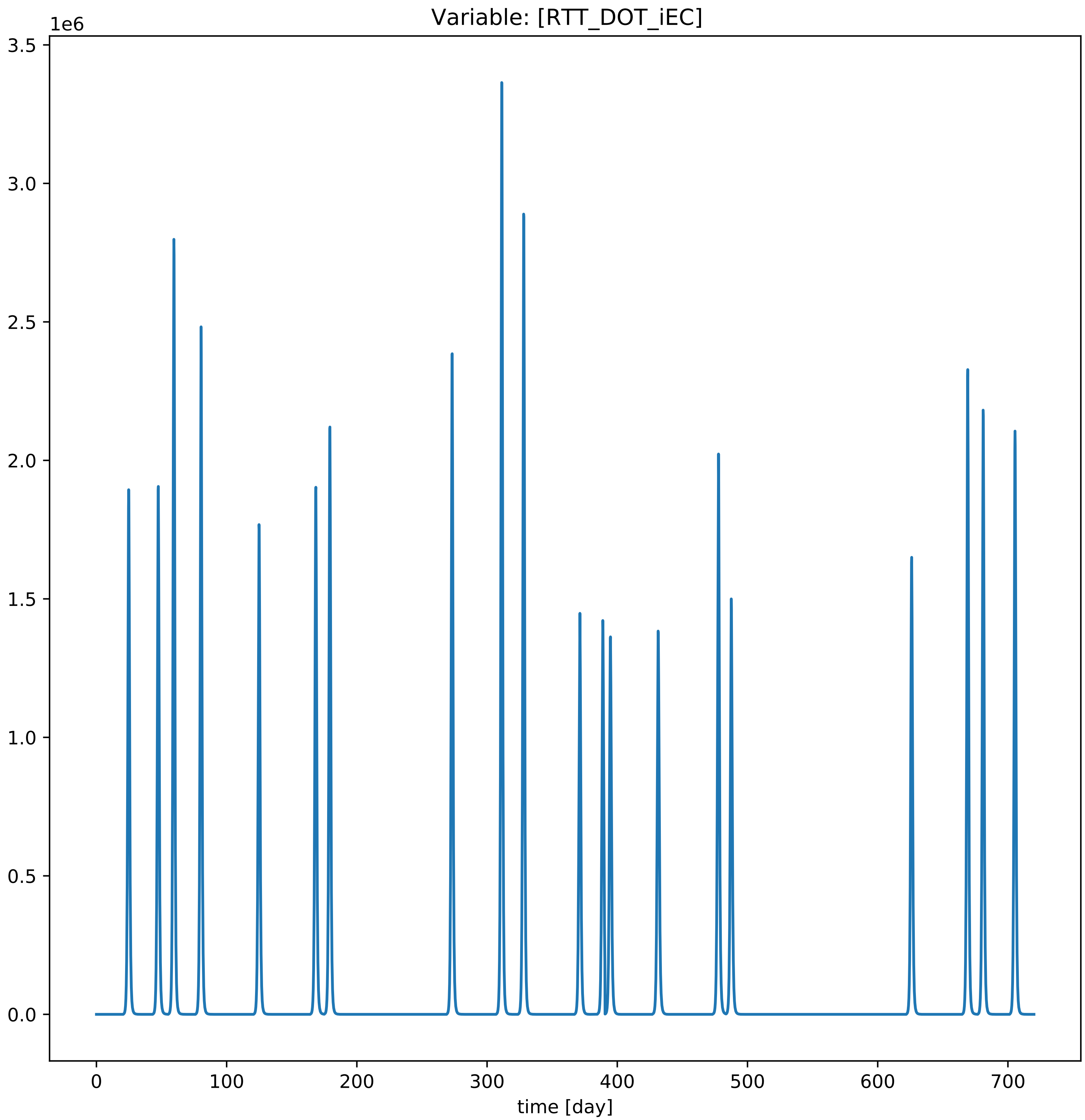

Variable: [RTT_DOT_iEC]
1e6
3.5
3.0
2.5
2.0
1.5
1.0
0.5
0.0
0
100
200
300
400
500
600
700
time [day]

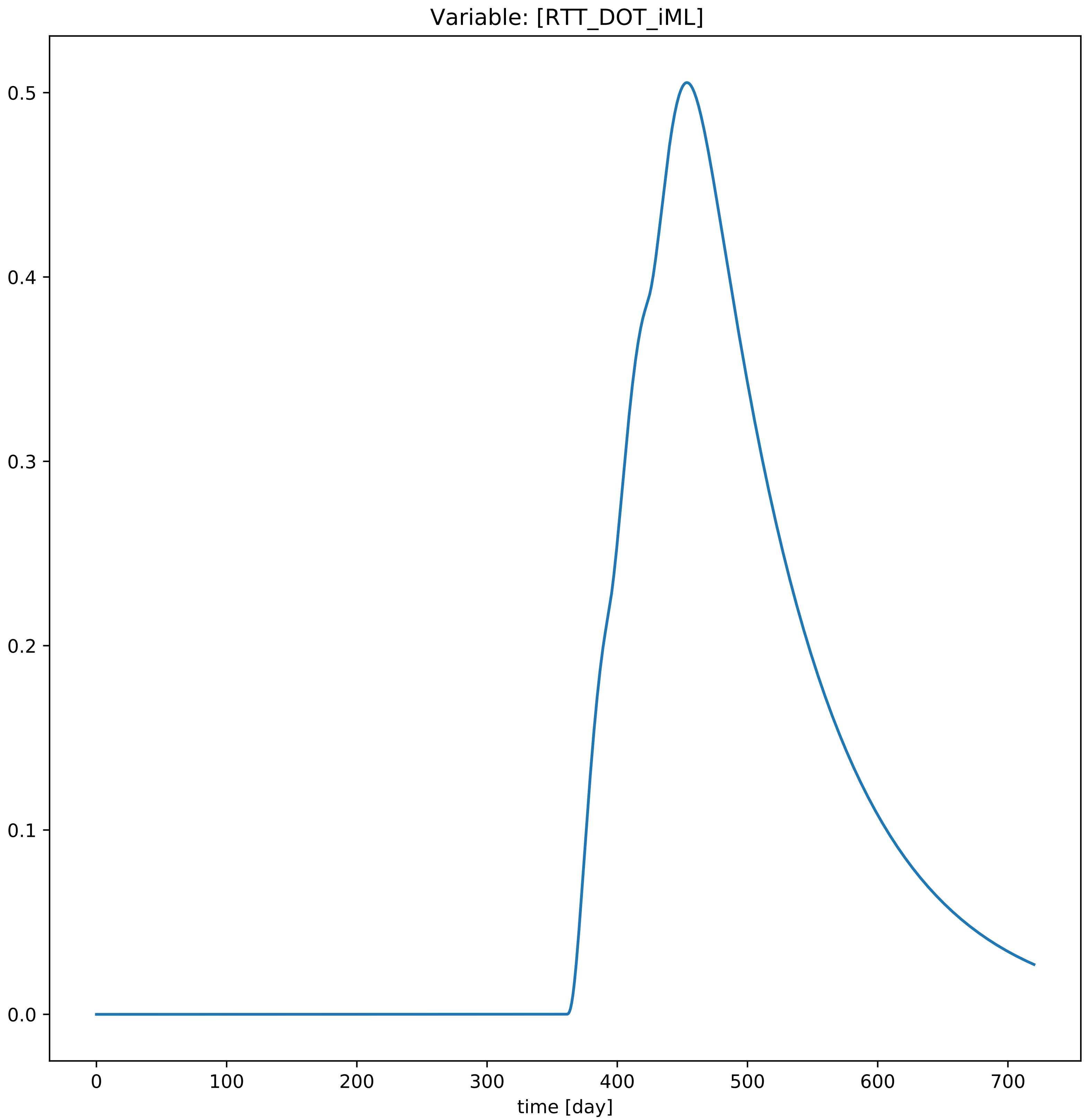
Variable: [RTT_DOT_iML]
0.5
0.4
0.3
0.2
0.1
0.0
0
100
200
300
400
500
600
700
time [day]

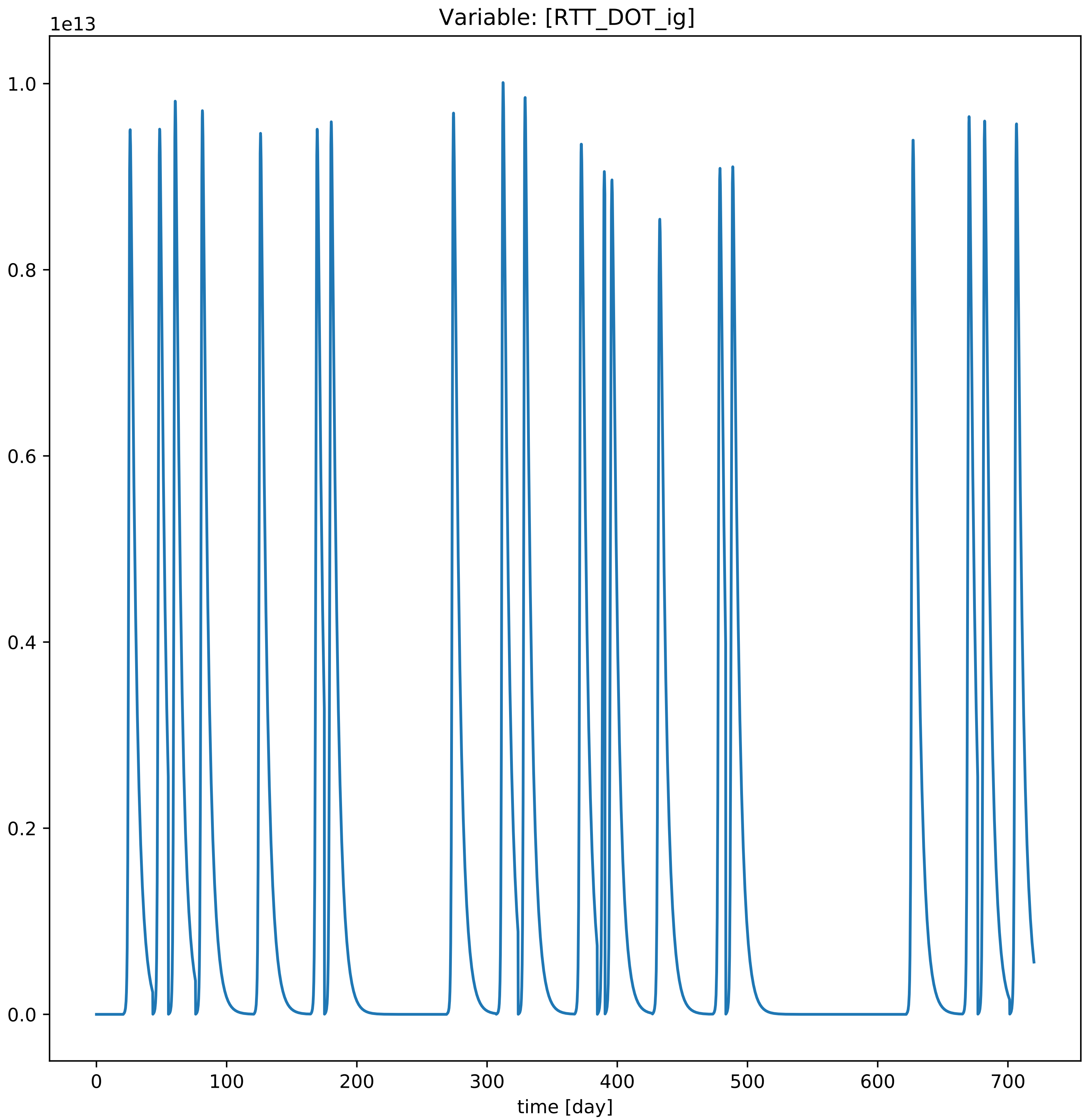

Variable: [RTT_DOT_ig]
1e13
1.0
0.8
0.6
0.4
0.2
0.0
0
100
200
300
400
500
600
700
time [day]

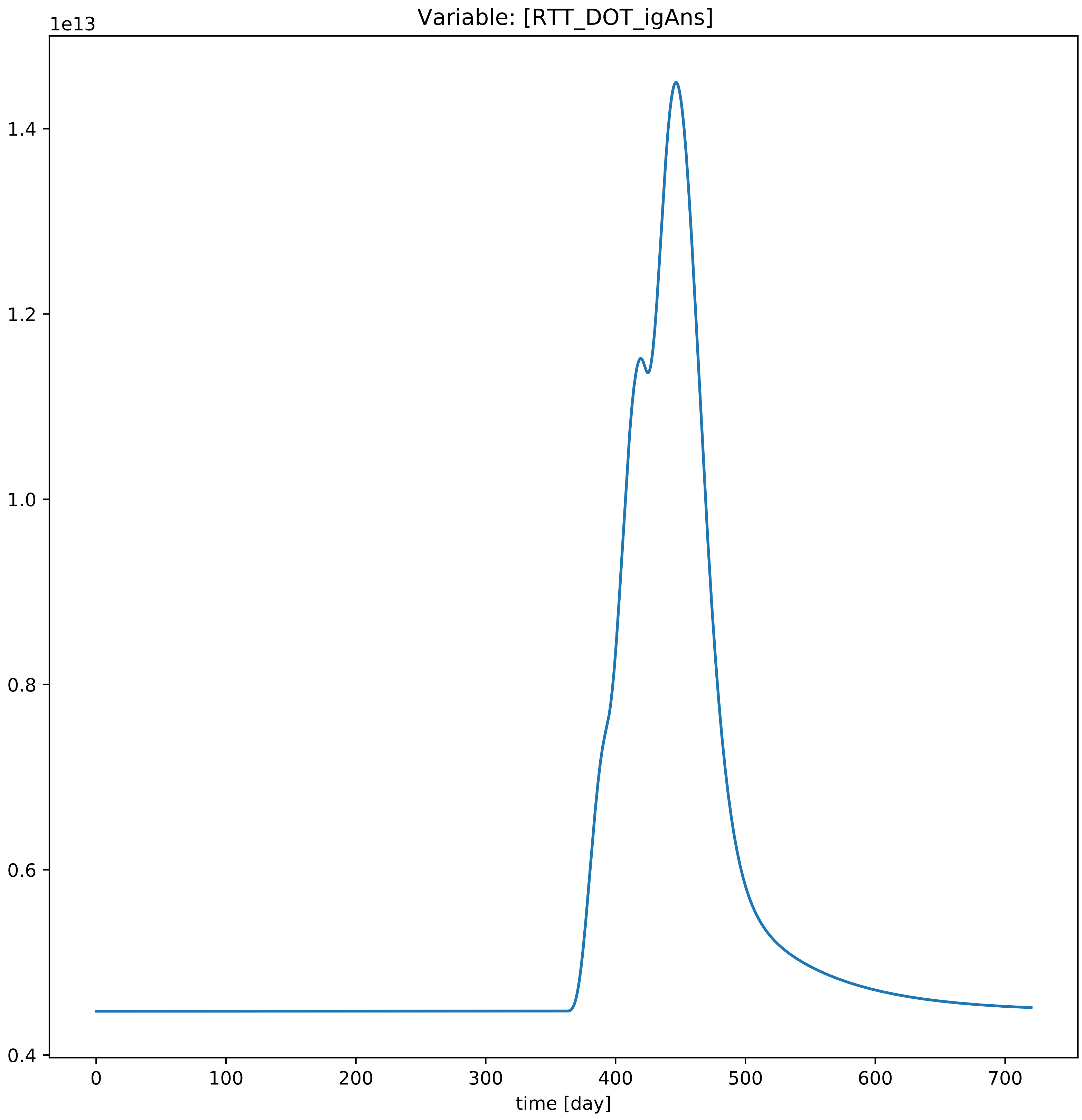
Variable: [RTT_DOT_igAns]
1e13
1.4
1.2
1.0
0.8
0.6
0.4
0
100
200
300
400
500
600
700
time [day]

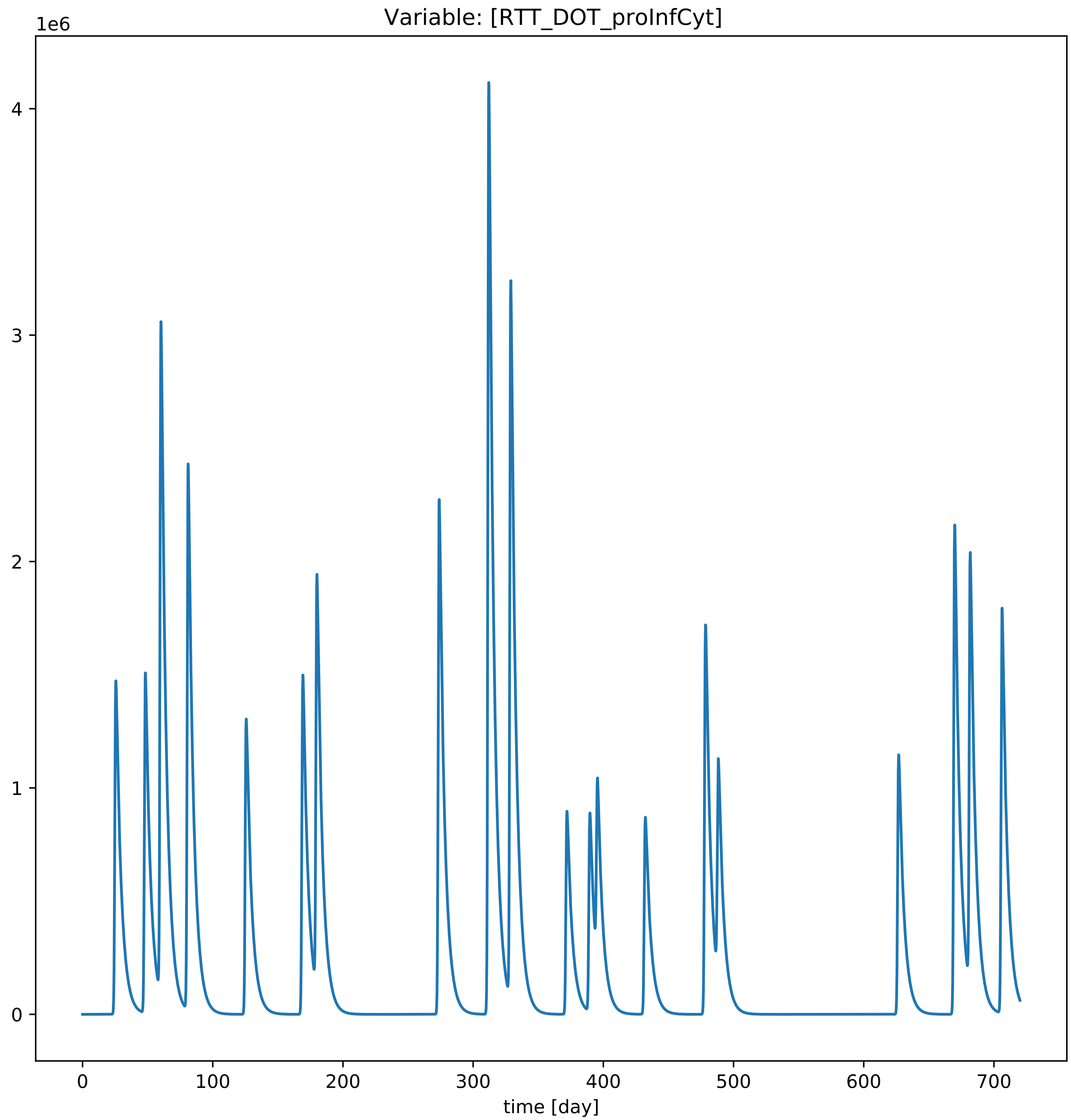
Variable: [RTT_DOT_proInfCyt]
1e6
4
3
2
1
0
0
100
200
300
400
500
600
700
time [day]

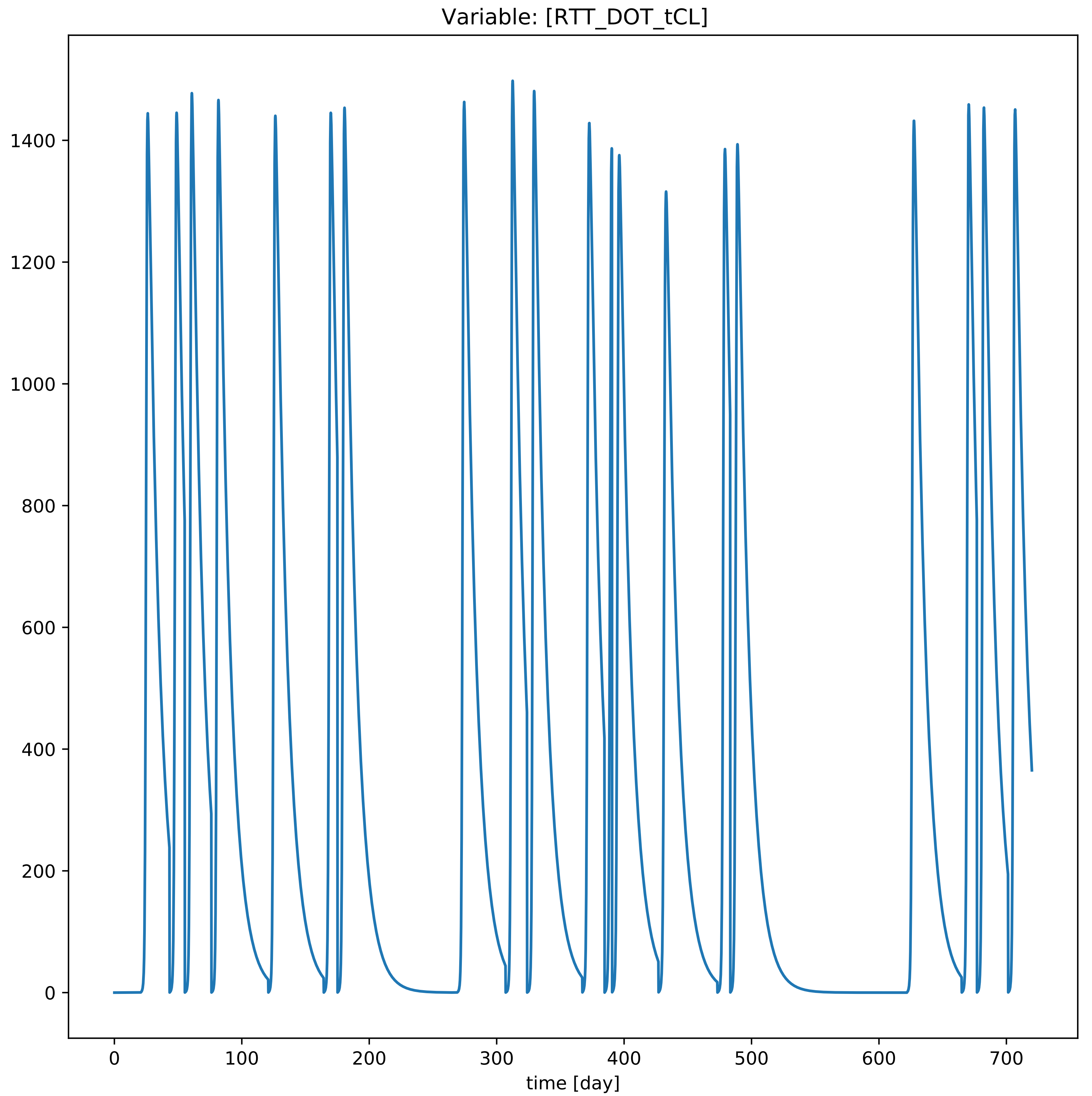

Variable: [RTT_DOT_tCL]
0
200
400
600
800
1000
1200
1400
0
100
200
300
400
500
600
700
time [day]

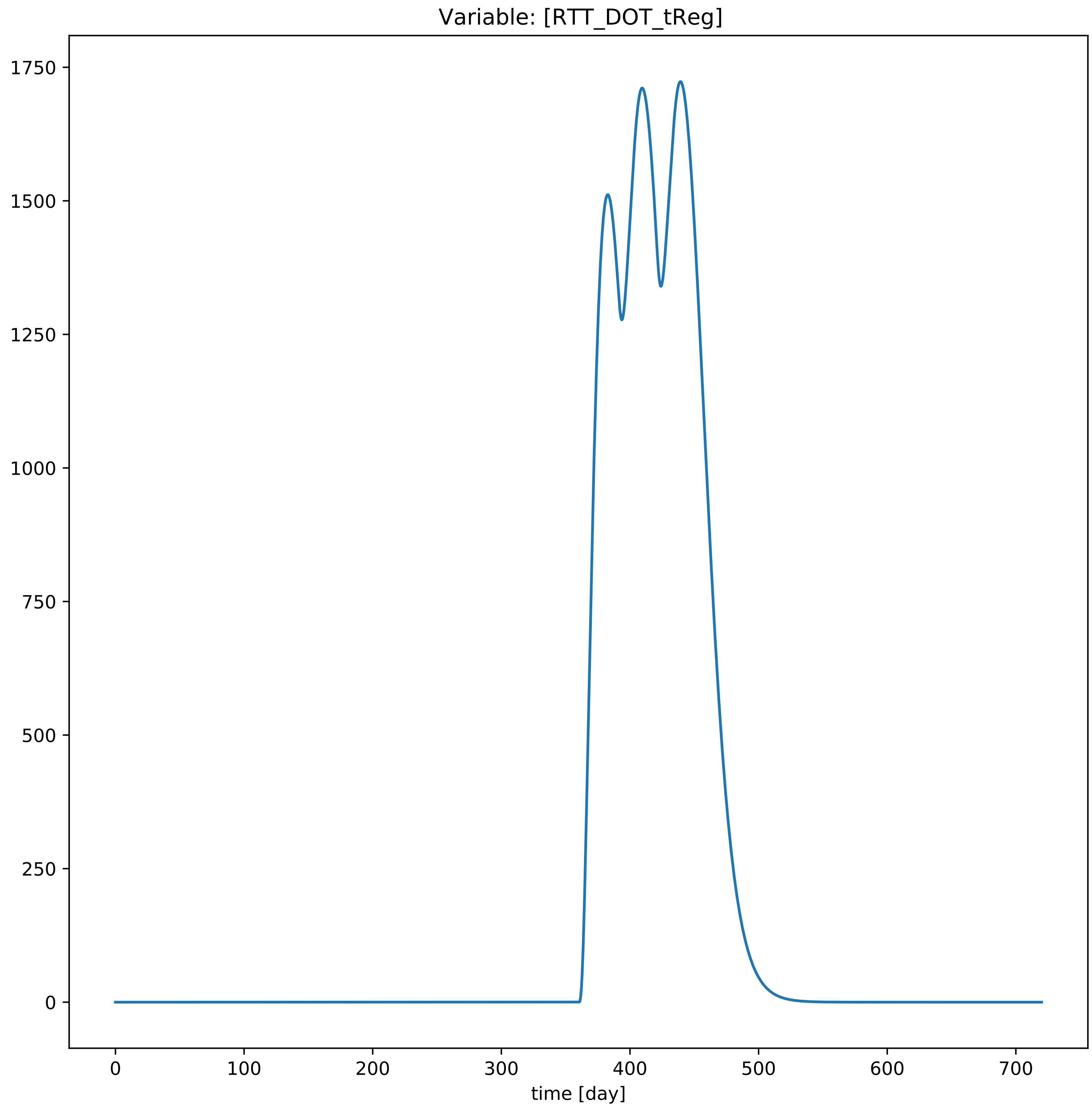
Variable: [RTT_DOT_tReg]
1750
1500
1250
1000
750
500
250
0
0
100
200
300
400
500
600
700
time [day]

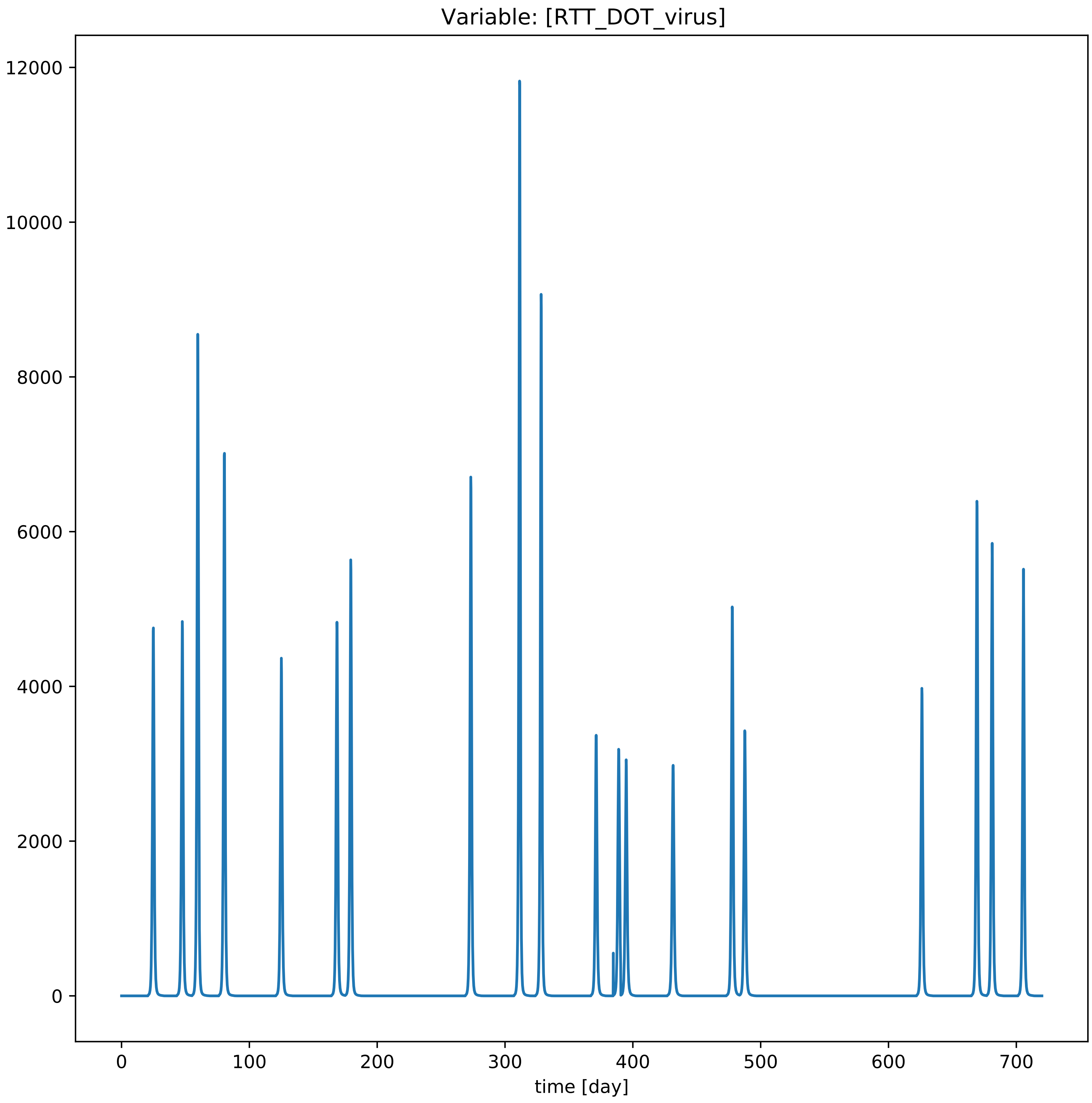

Variable: [RTT_DOT_virus]
12000
10000
8000
6000
4000
2000
0
0
100
200
300
400
500
600
700
time [day]

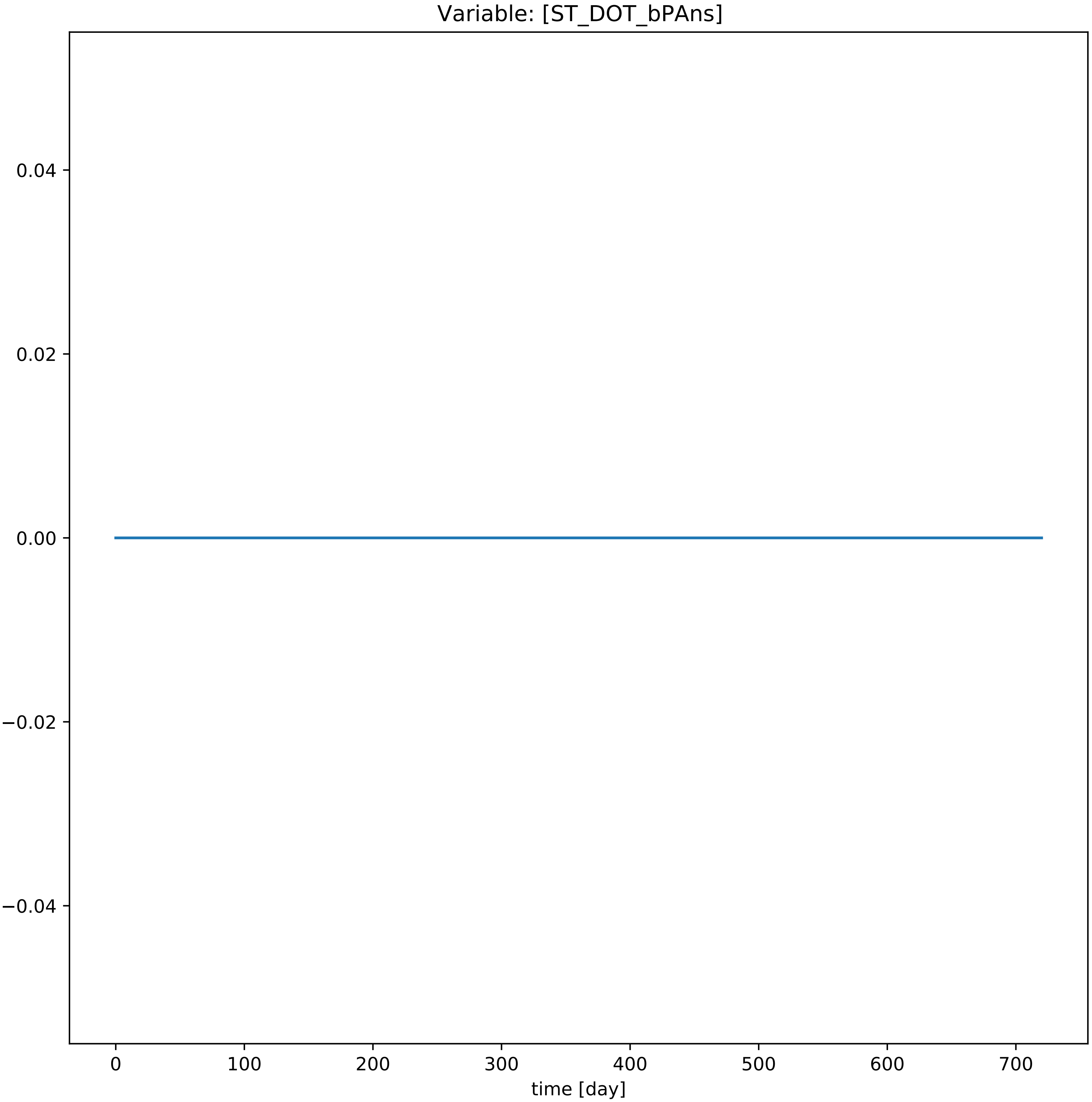

Variable: [ST_DOT_bPAns]
0.04
0.02
0.00
−0.02
−0.04
0
100
200
300
400
500
600
700
time [day]

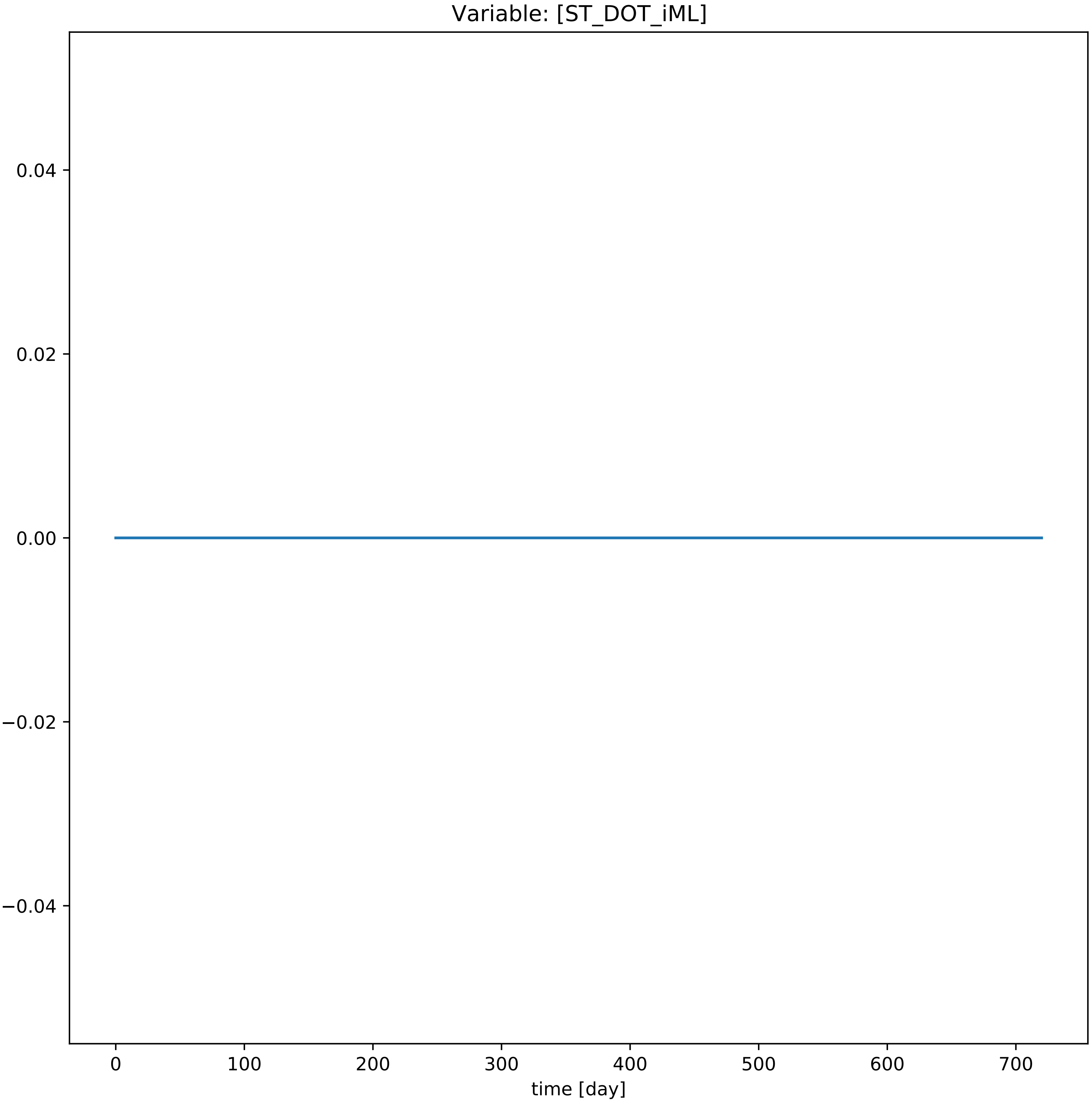

Variable: [ST_DOT_iML]
0.04
0.02
0.00
−0.02
−0.04
0
100
200
300
400
500
600
700
time [day]

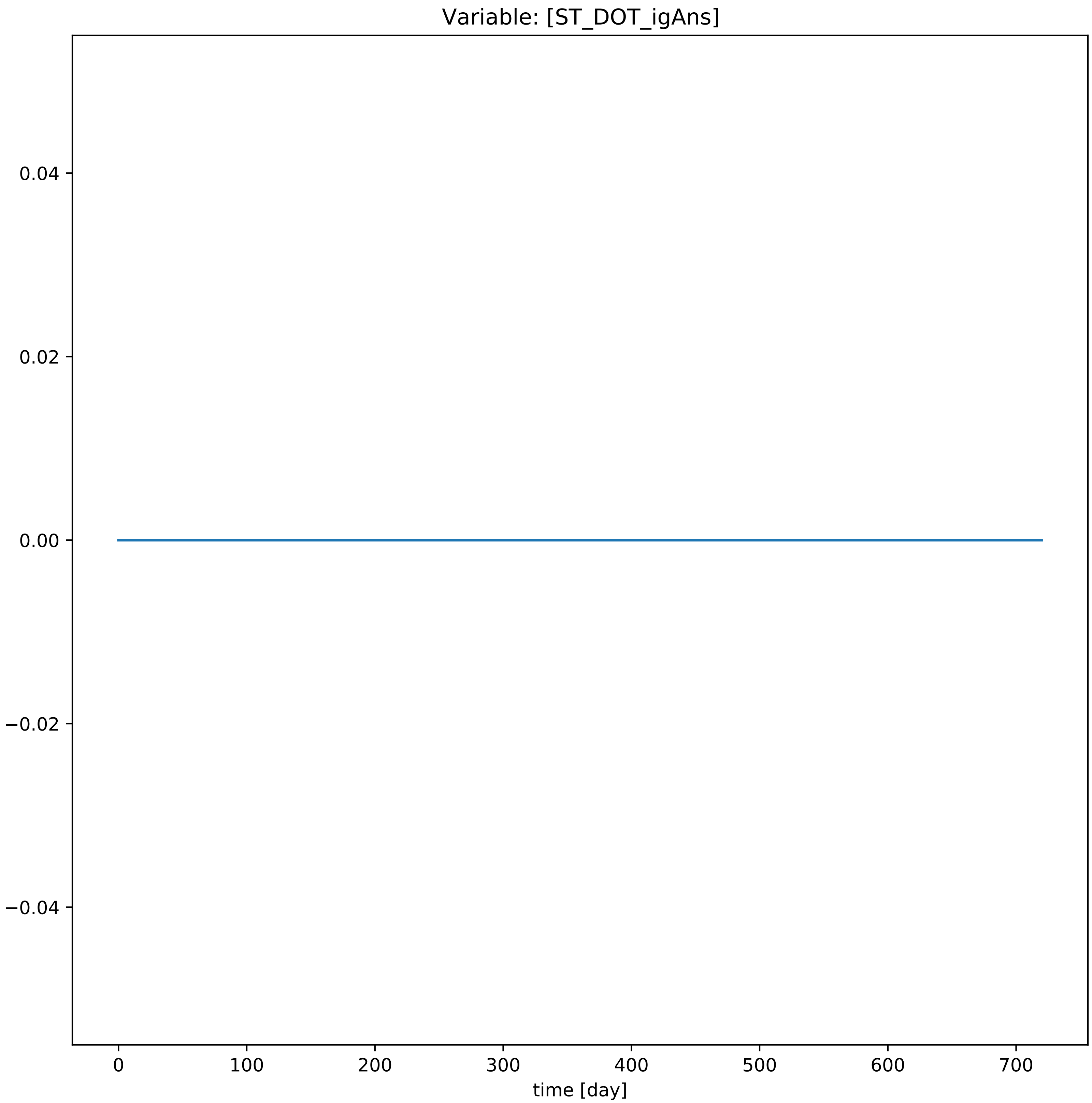

Variable: [ST_DOT_igAns]
0.04
0.02
0.00
−0.02
−0.04
0
100
200
300
400
500
600
700
time [day]

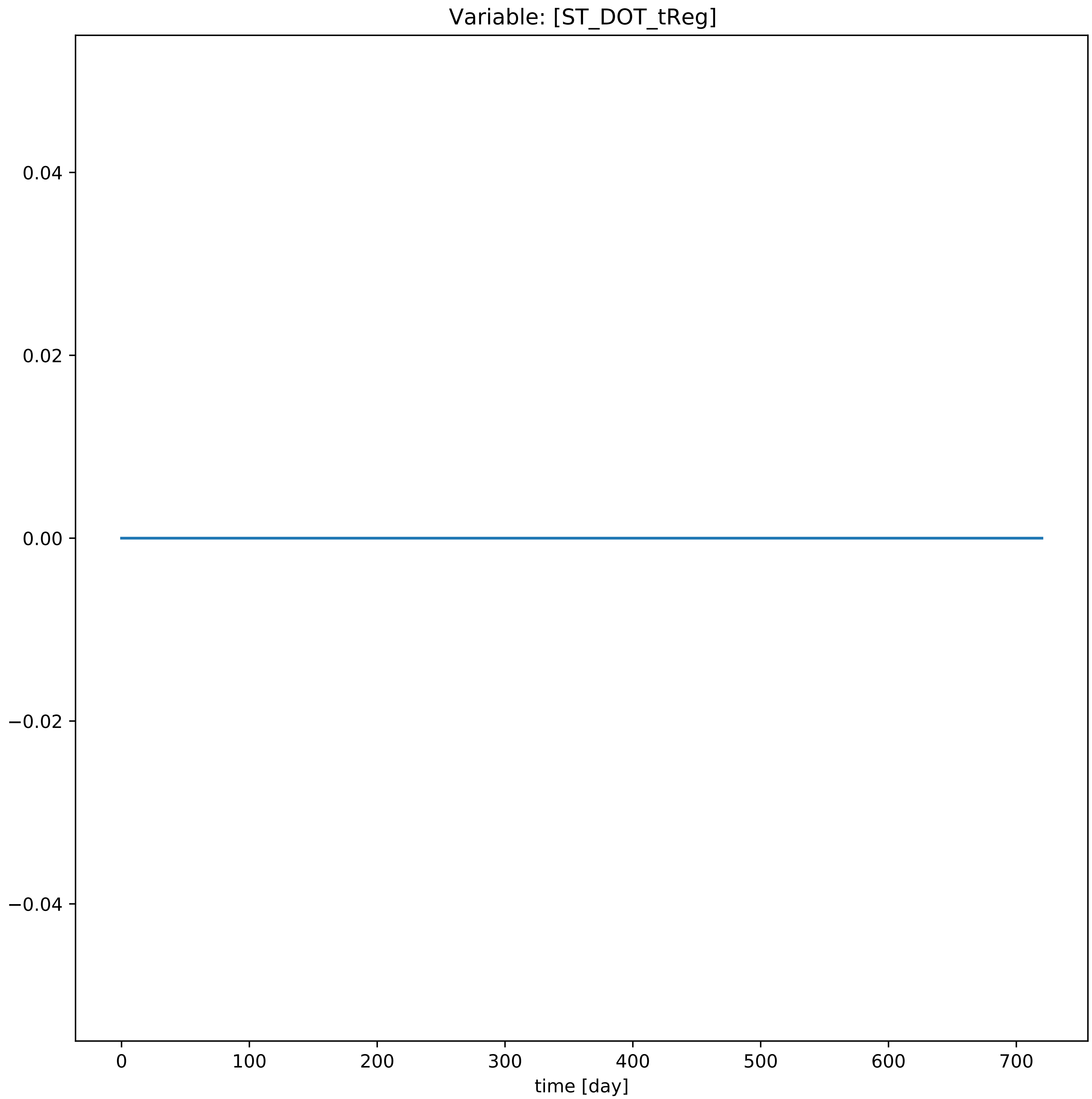
Variable: [ST_DOT_tReg]
0.04
0.02
0.00
−0.02
−0.04
0
100
200
300
400
500
600
700
time [day]

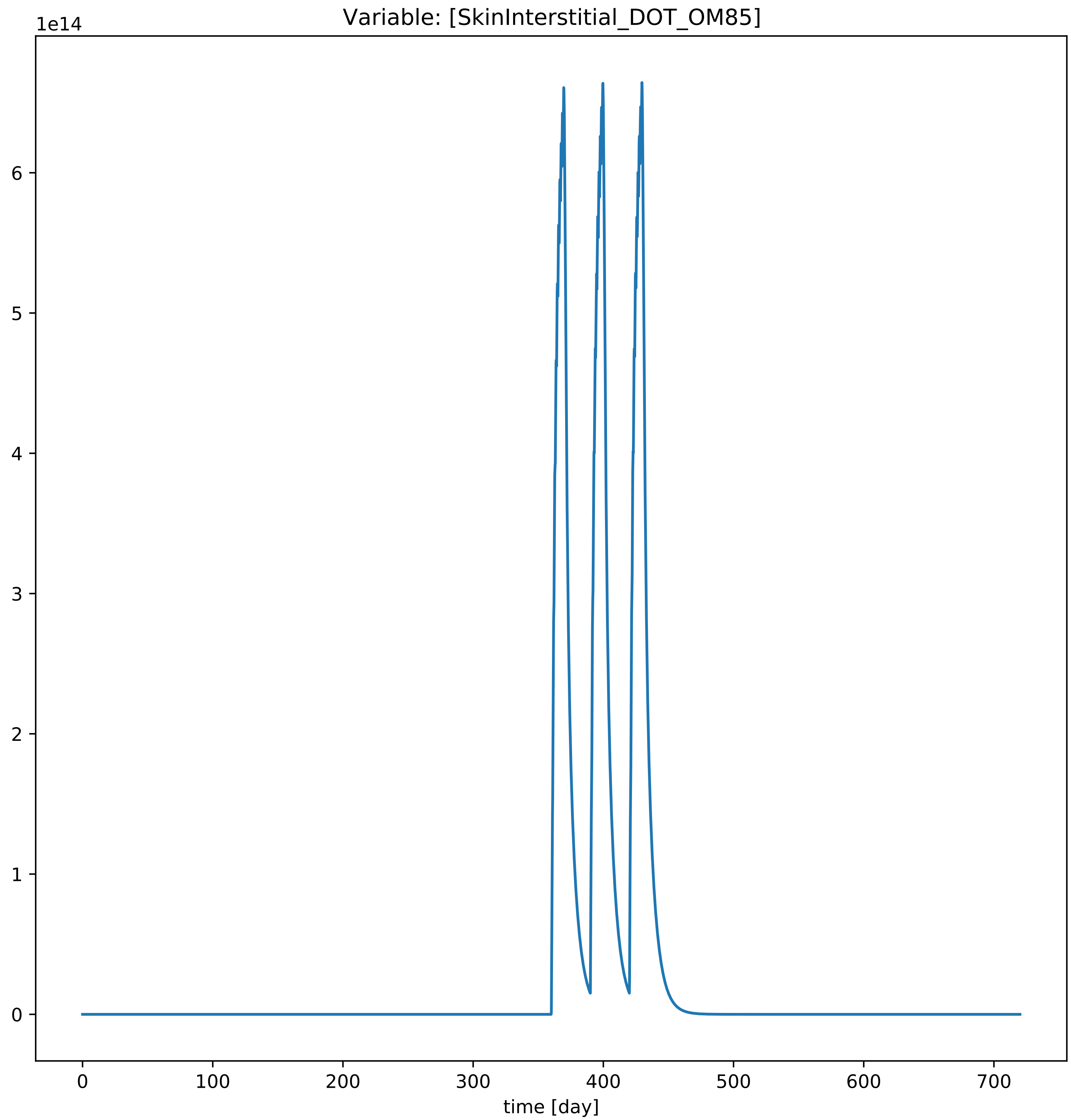
Variable: [SkinInterstitial_DOT_OM85]
1e14
0
1
2
3
4
5
6
0
100
200
300
400
500
600
700
time [day]

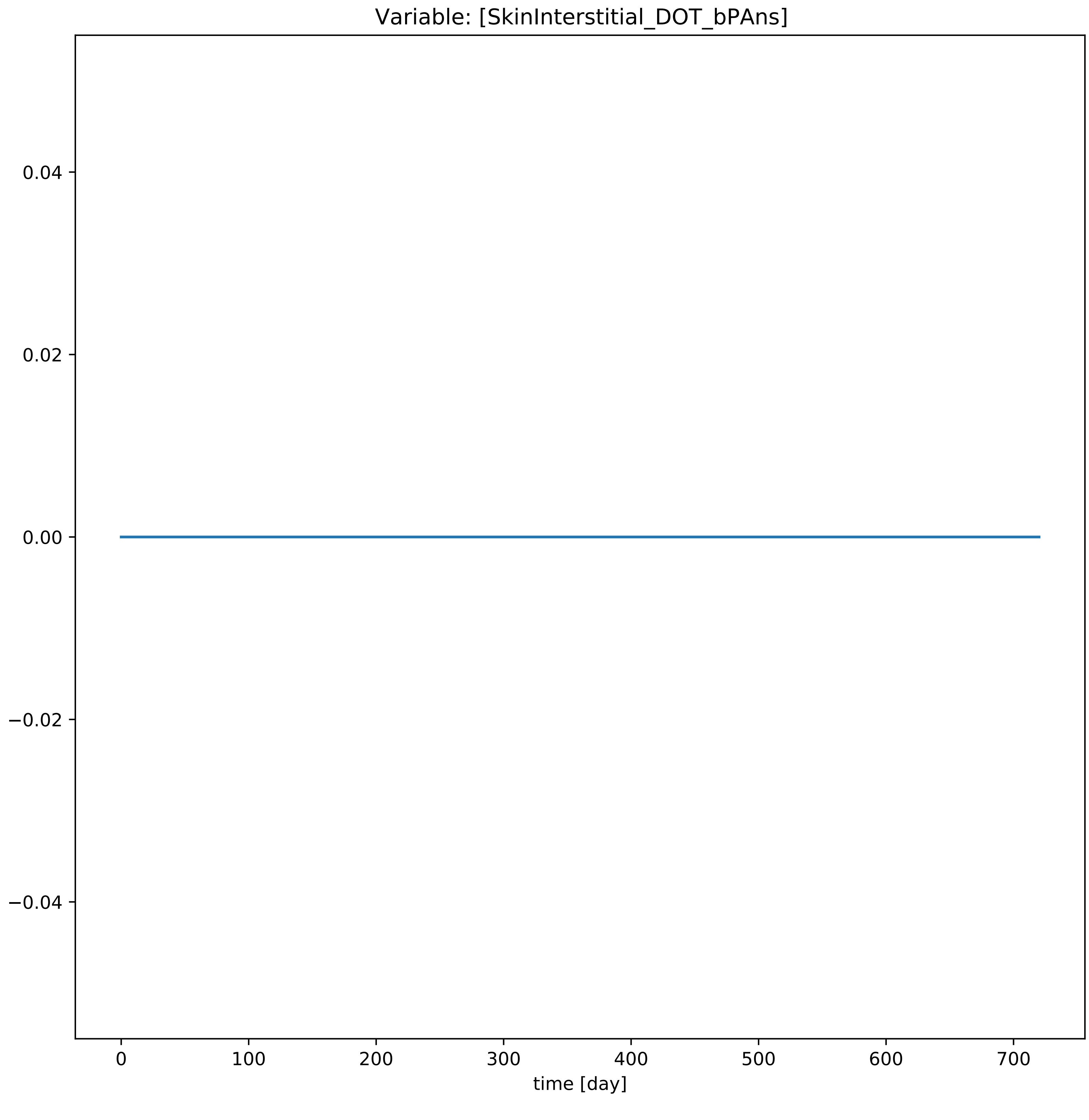

Variable: [SkinInterstitial_DOT_bPAns]
0.04
0.02
0.00
−0.02
−0.04
0
100
200
300
400
500
600
700
time [day]

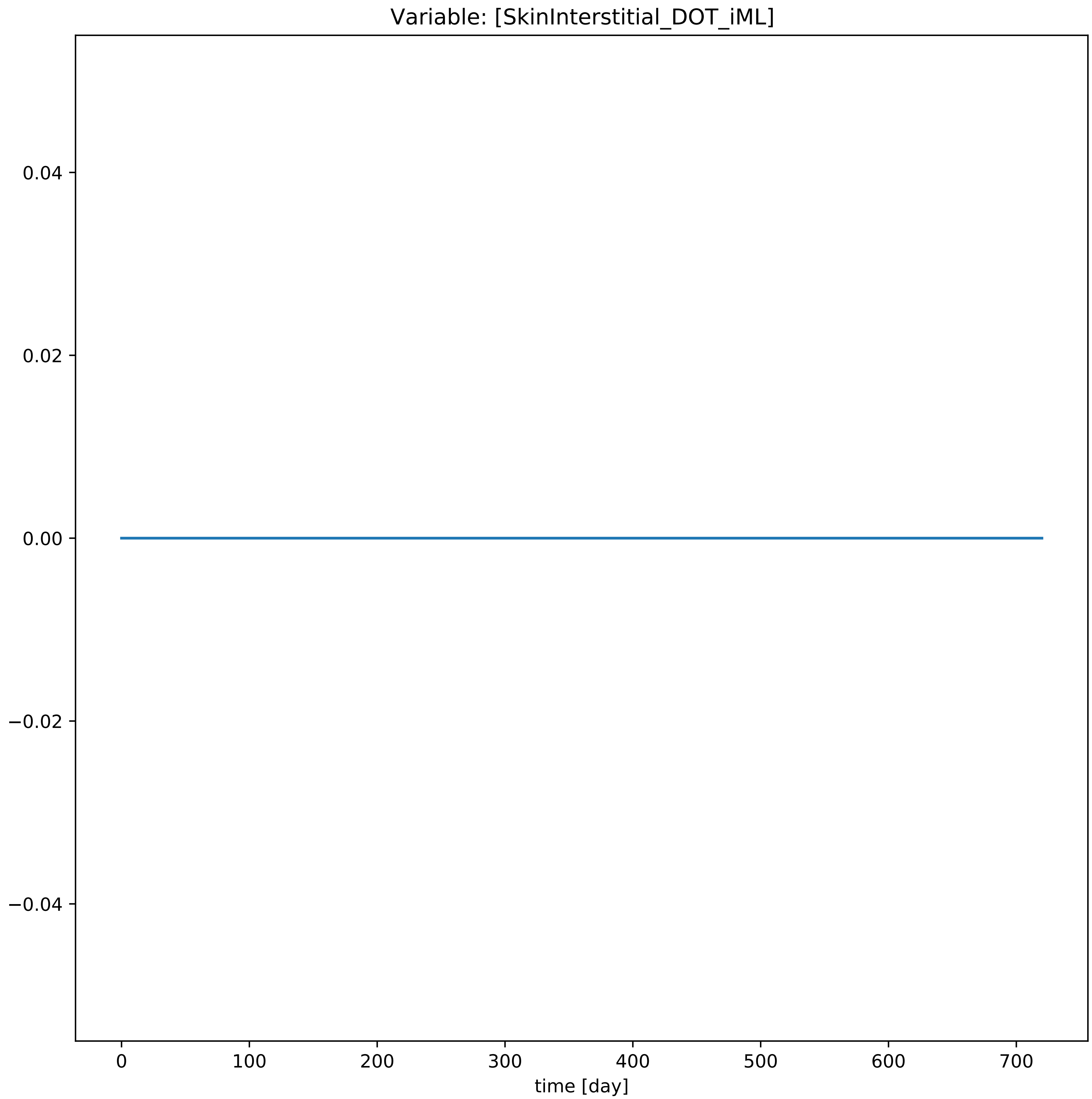
Variable: [SkinInterstitial_DOT_iML]
0.04
0.02
0.00
−0.02
−0.04
0
100
200
300
400
500
600
700
time [day]

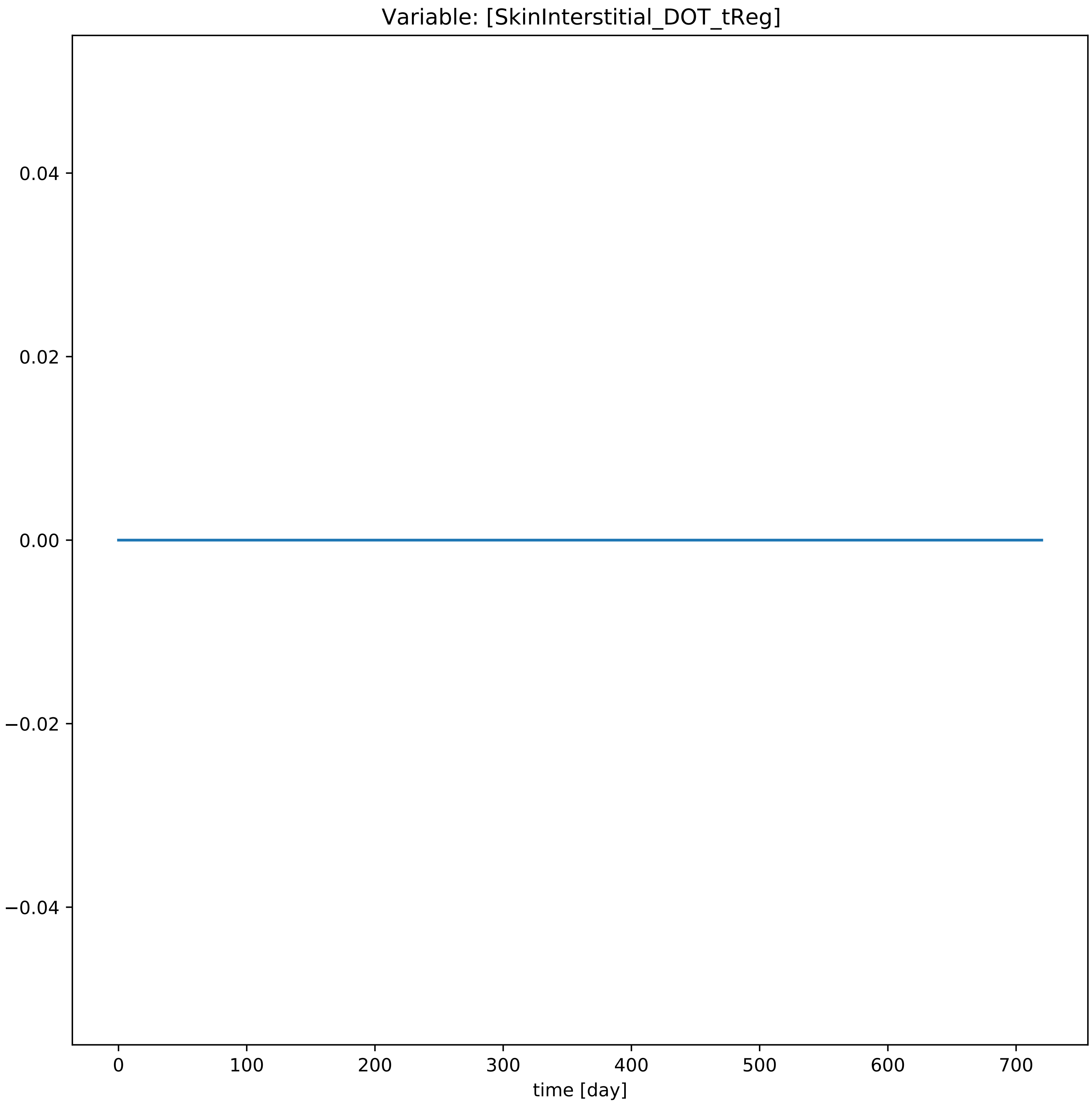

Variable: [SkinInterstitial_DOT_tReg]
0.04
0.02
0.00
−0.02
−0.04
0
100
200
300
400
500
600
700
time [day]

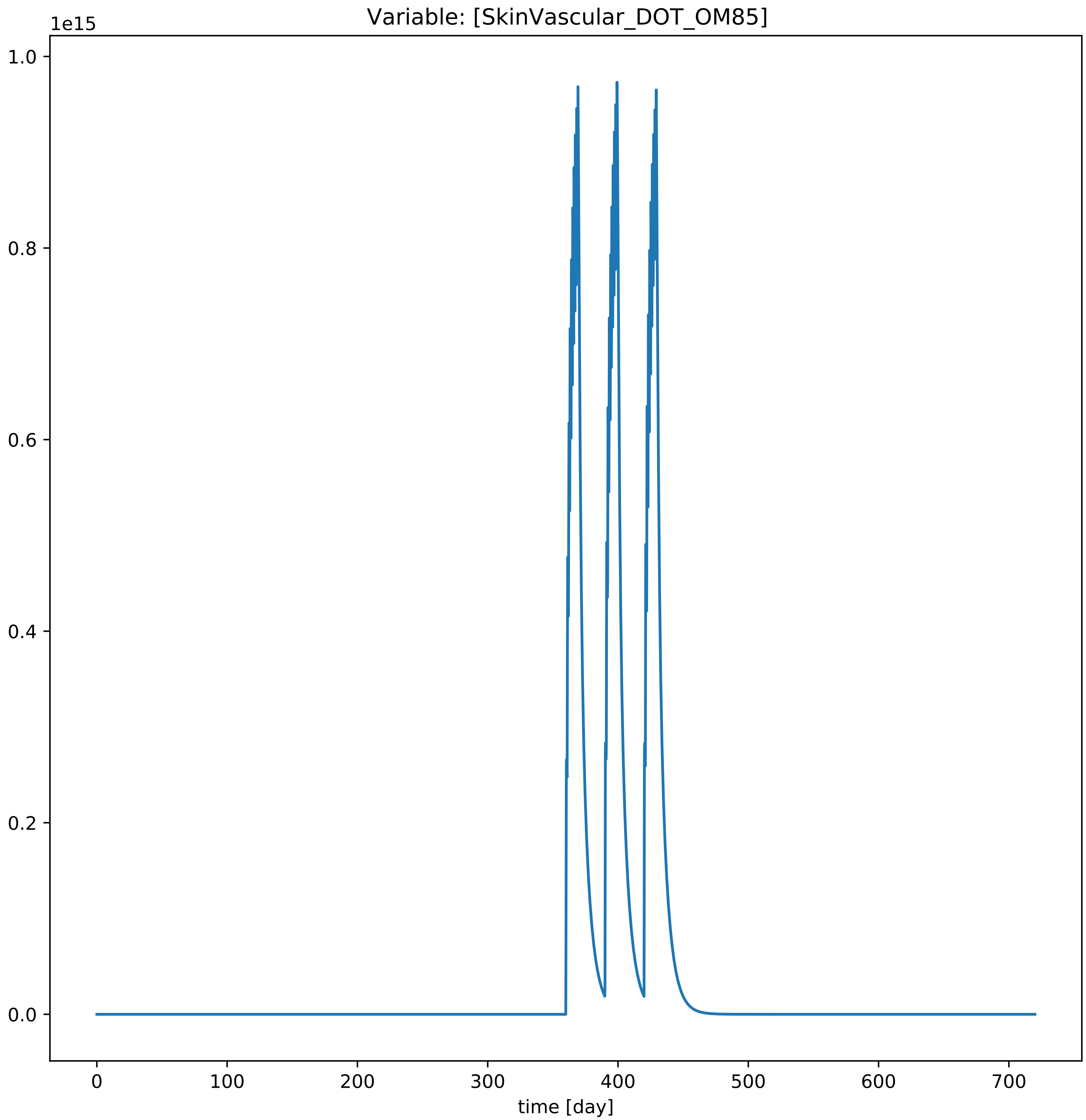

Variable: [SkinVascular_DOT_OM85]
1e15
1.0
0.8
0.6
0.4
0.2
0.0
0
100
200
300
400
500
600
700
time [day]

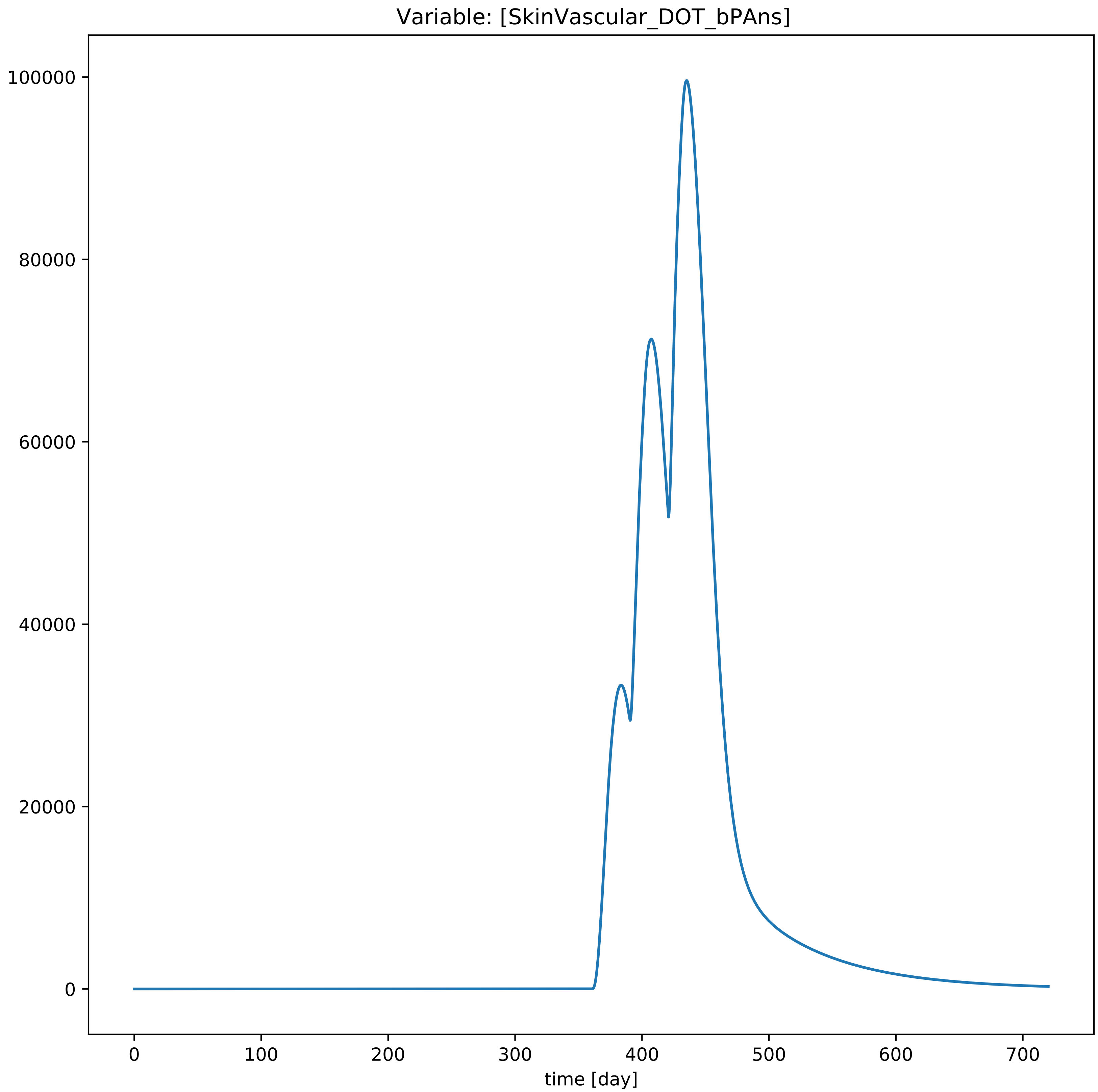

Variable: [SkinVascular_DOT_bPAns]
100000
80000
60000
40000
20000
0
0
100
200
300
400
500
600
700
time [day]

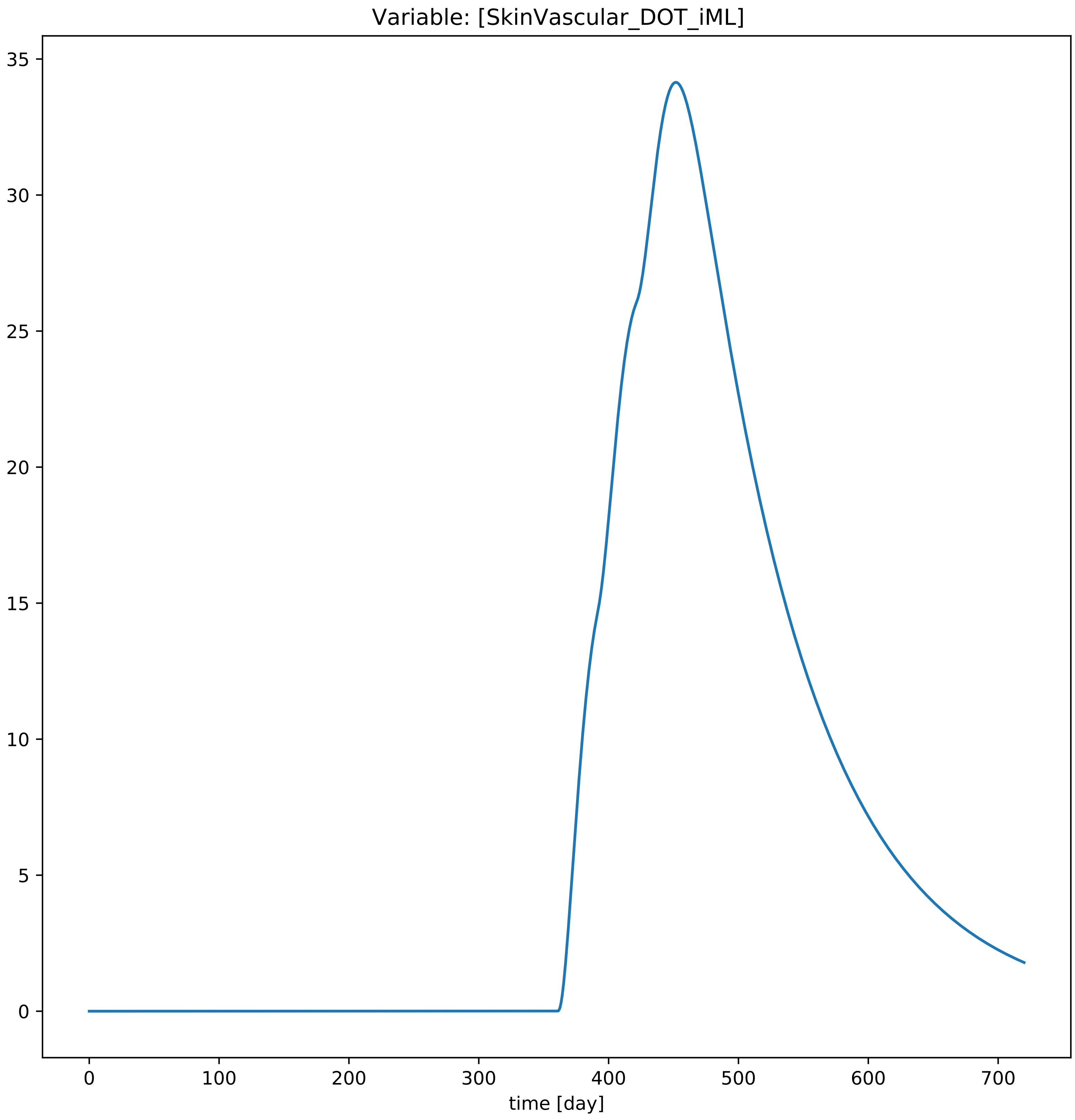

Variable: [SkinVascular_DOT_iML]
35
30
25
20
15
10
5
0
0
100
200
300
400
500
600
700
time [day]

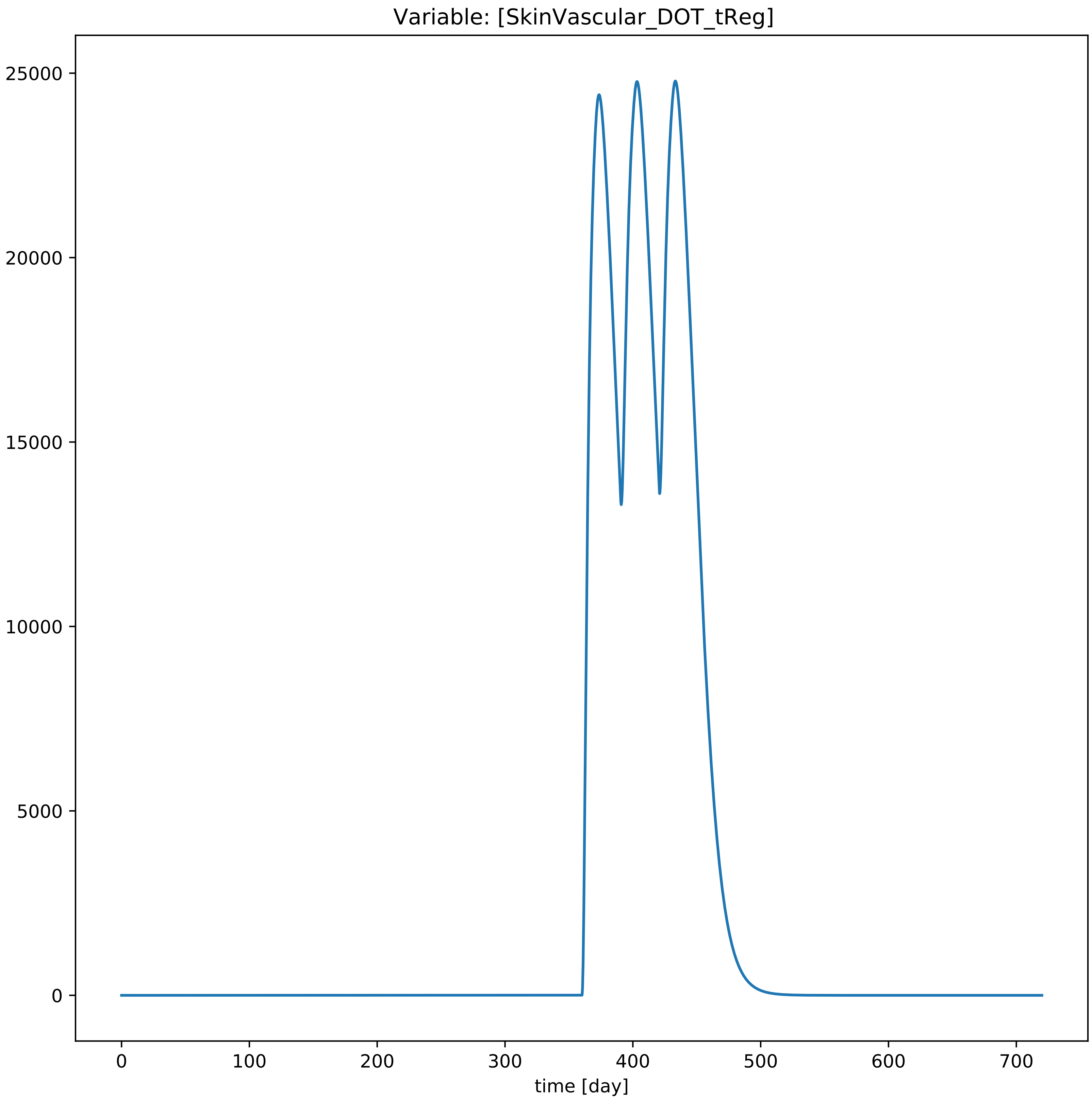

Variable: [SkinVascular_DOT_tReg]
25000
20000
15000
10000
5000
0
0
100
200
300
400
500
600
700
time [day]

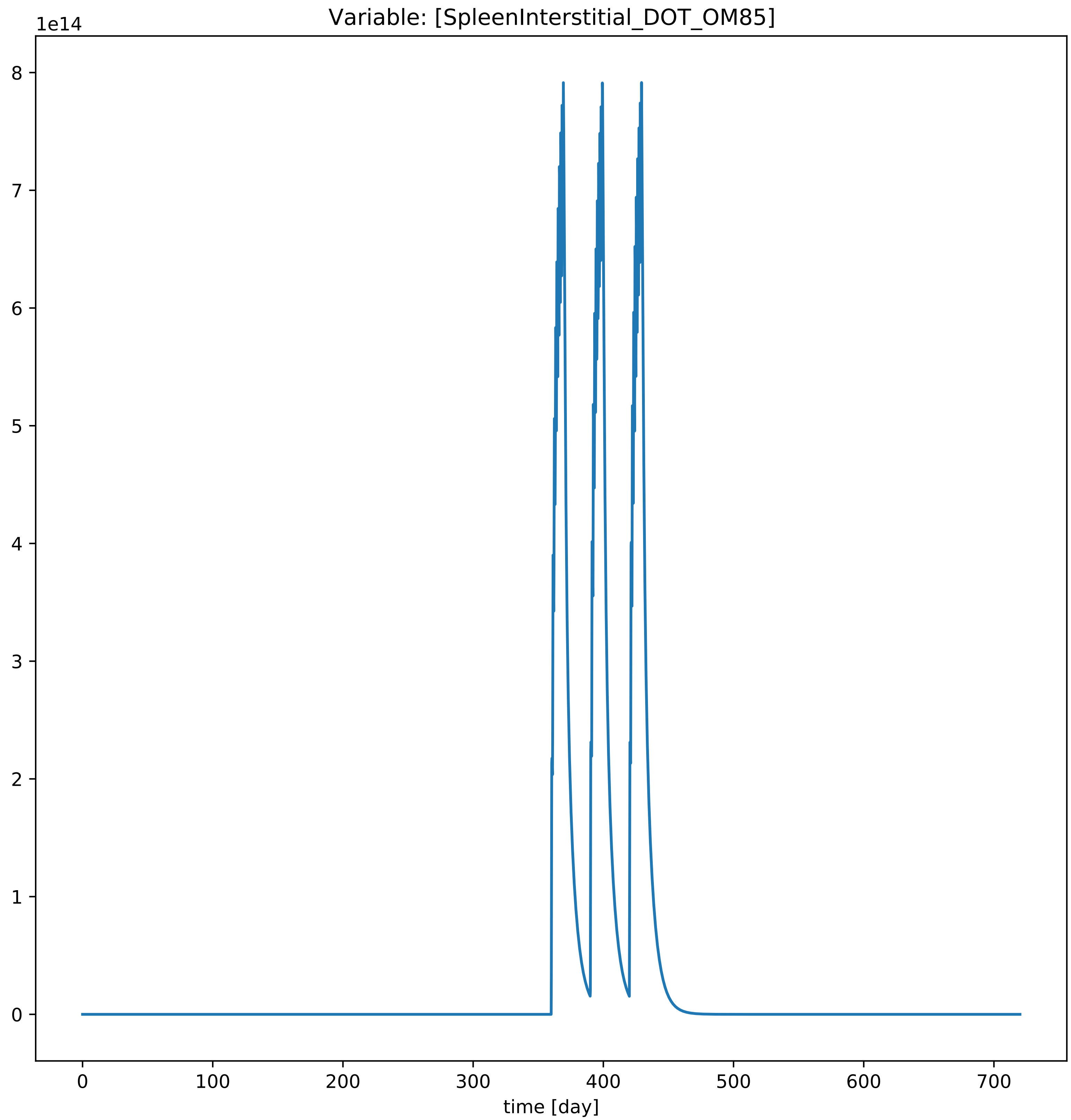
Variable: [SpleenInterstitial_DOT_OM85]
1e14
8
7
6
5
4
3
2
1
0
0
100
200
300
400
500
600
700
time [day]

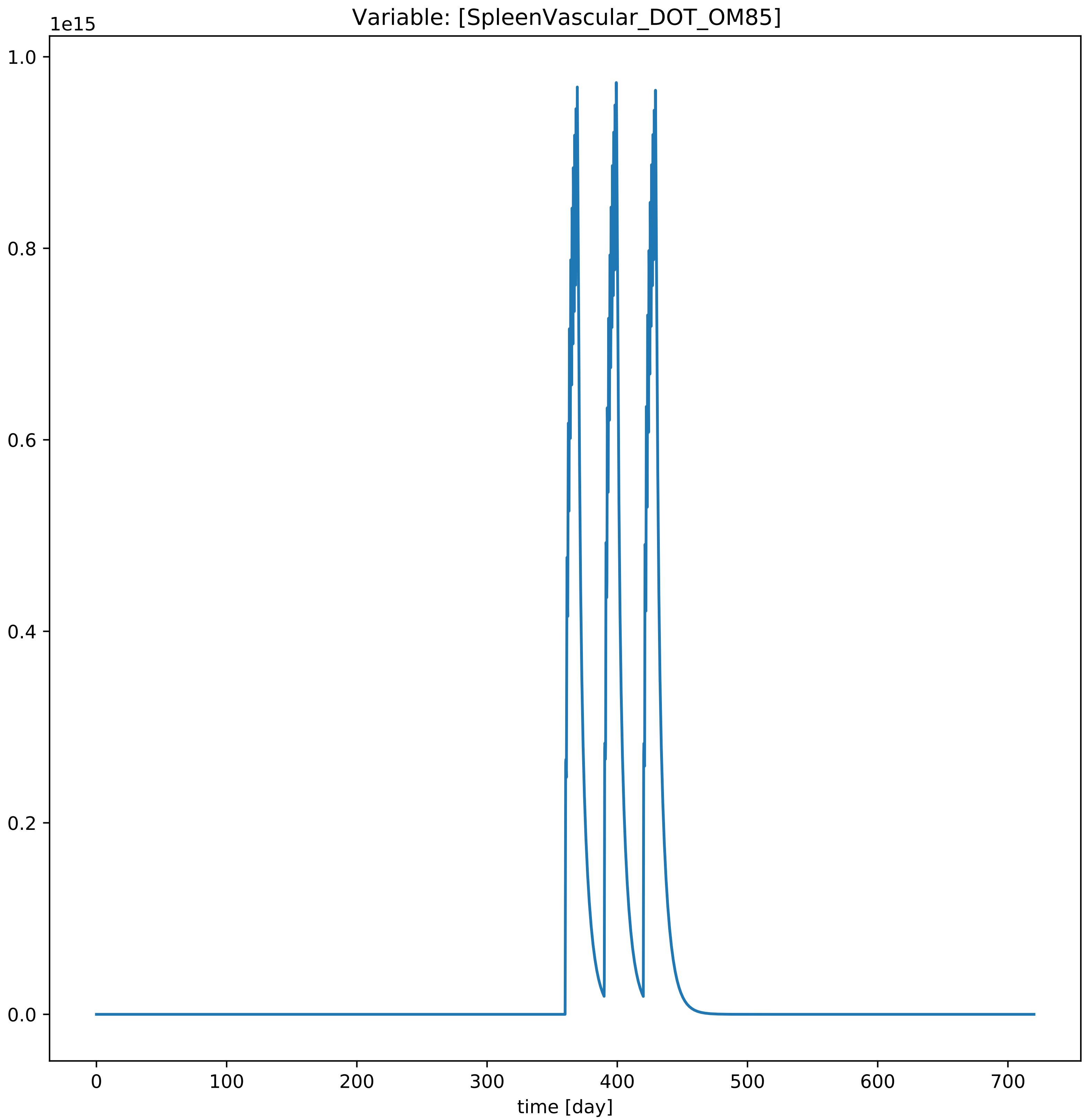

Variable: [SpleenVascular_DOT_OM85]
1e15
1.0
0.8
0.6
0.4
0.2
0.0
0
100
200
300
400
500
600
700
time [day]

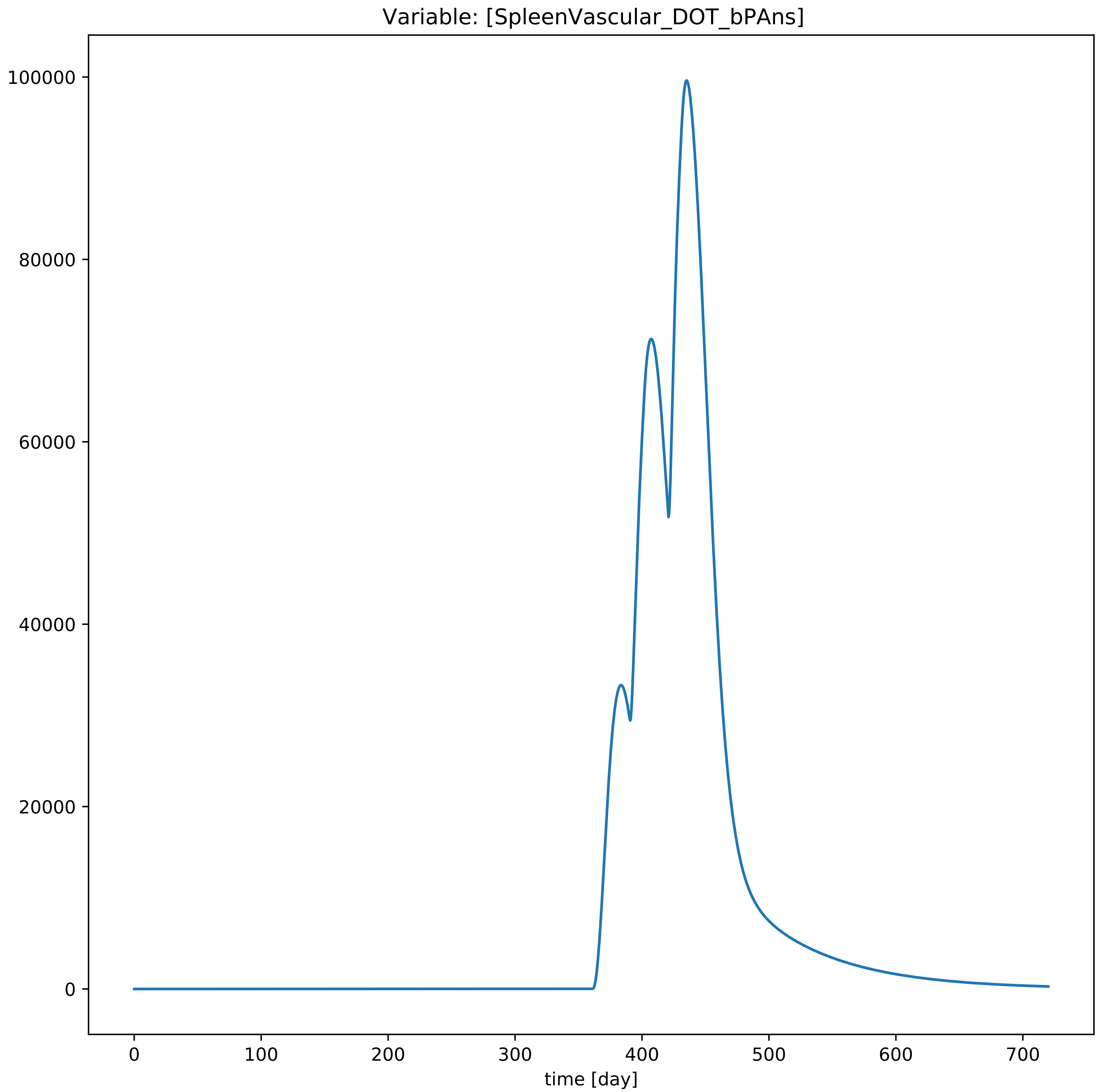

Variable: [SpleenVascular_DOT_bPAns]
100000
80000
60000
40000
20000
0
0
100
200
300
400
500
600
700
time [day]

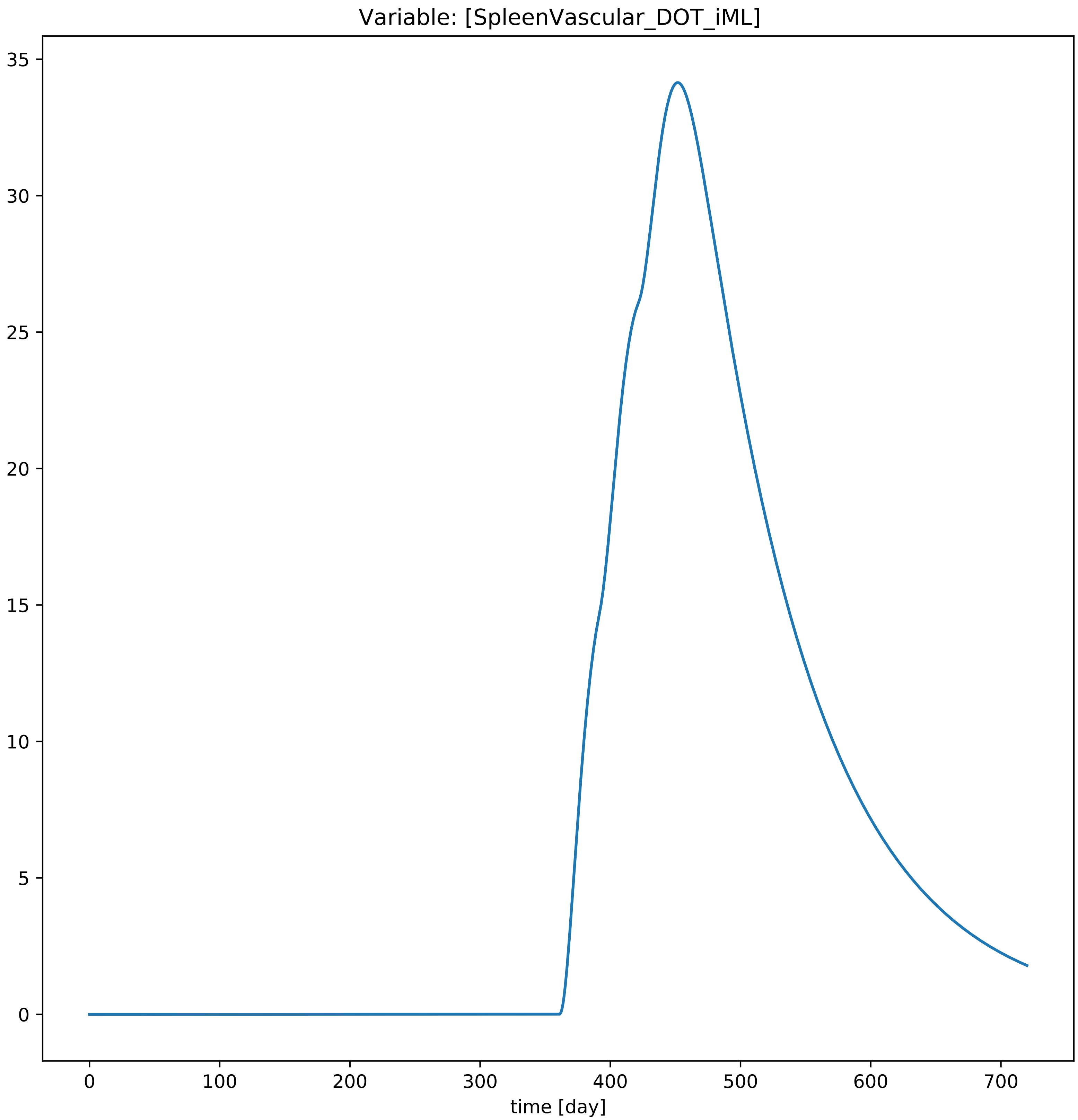

Variable: [SpleenVascular_DOT_iML]
35
30
25
20
15
10
5
0
0
100
200
300
400
500
600
700
time [day]

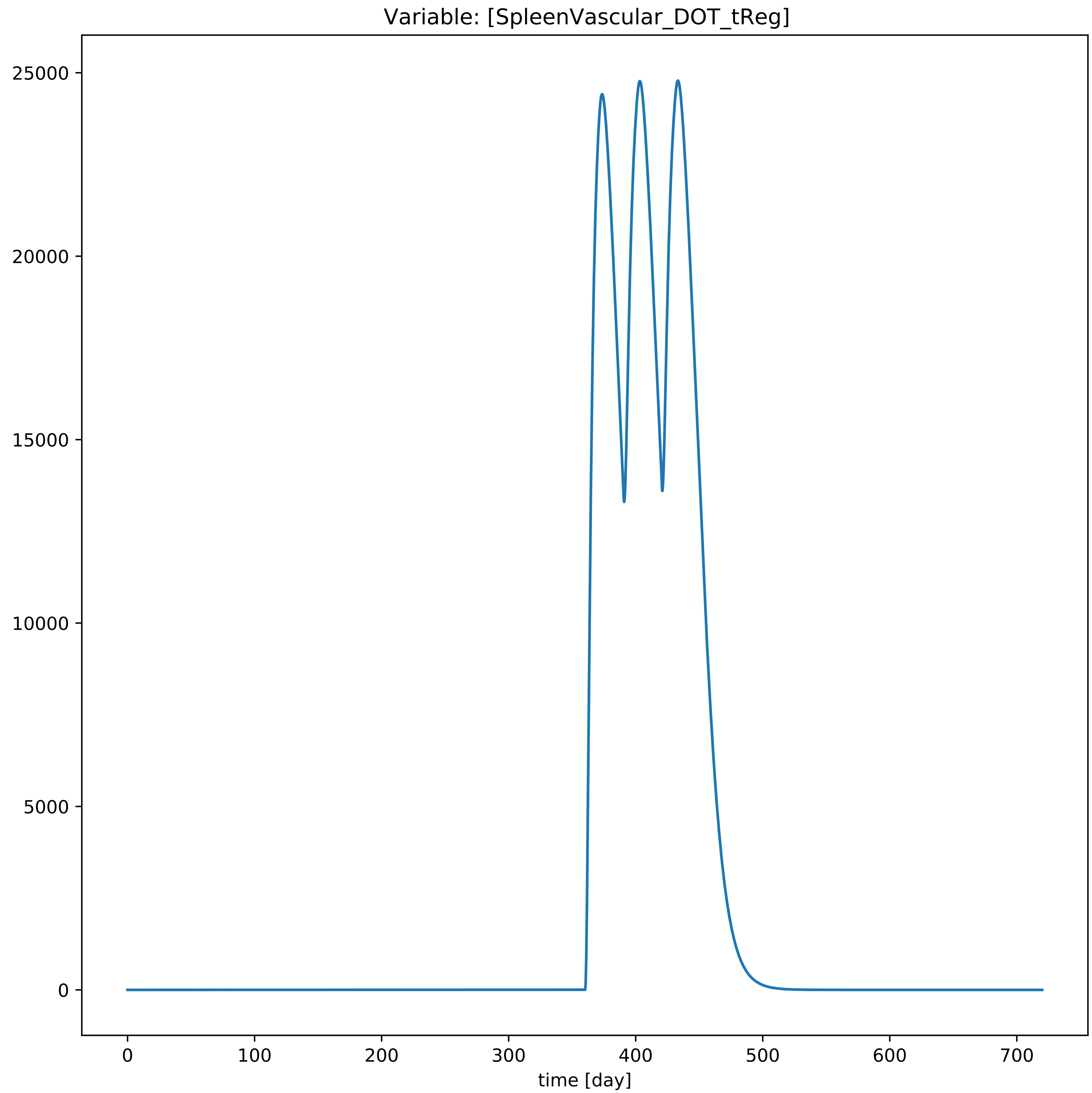

Variable: [SpleenVascular_DOT_tReg]
25000
20000
15000
10000
5000
0
0
100
200
300
400
500
600
700
time [day]

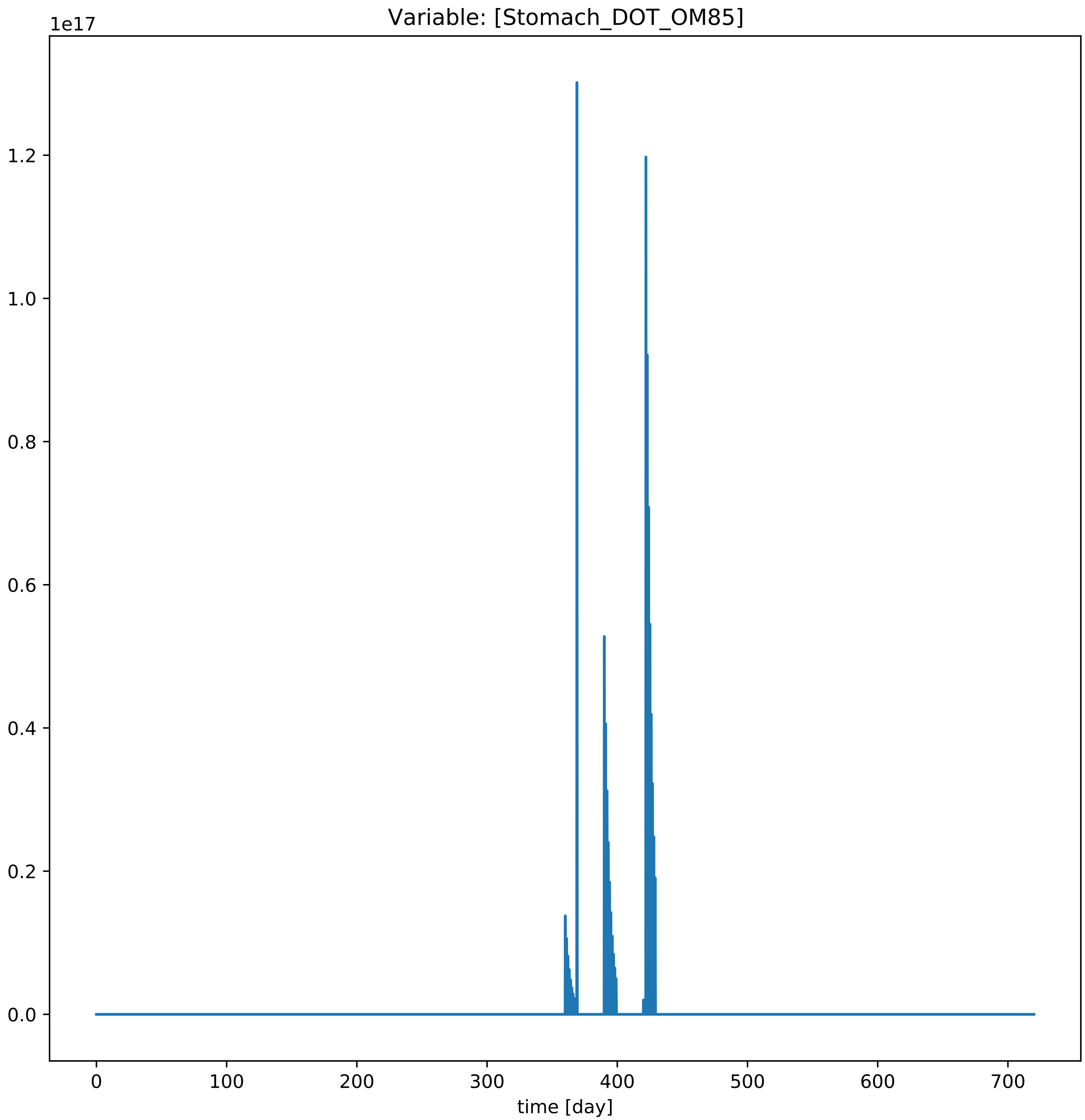

Variable: [Stomach_DOT_OM85]
1e17
1.2
1.0
0.8
0.6
0.4
0.2
0.0
0
100
200
300
400
500
600
700
time [day]

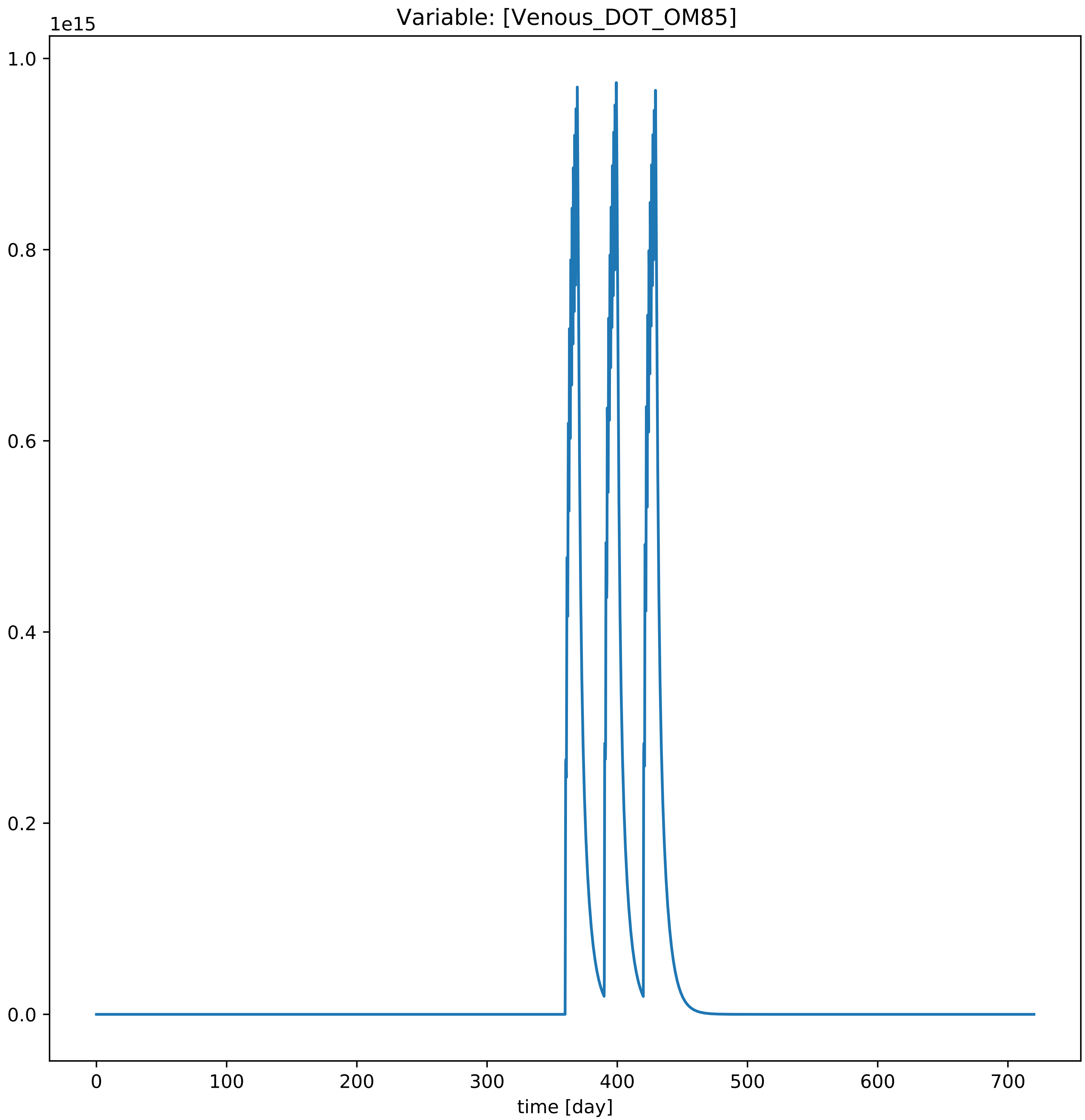
Variable: [Venous_DOT_OM85]
1e15
1.0
0.8
0.6
0.4
0.2
0.0
0
100
200
300
400
500
600
700
time [day]

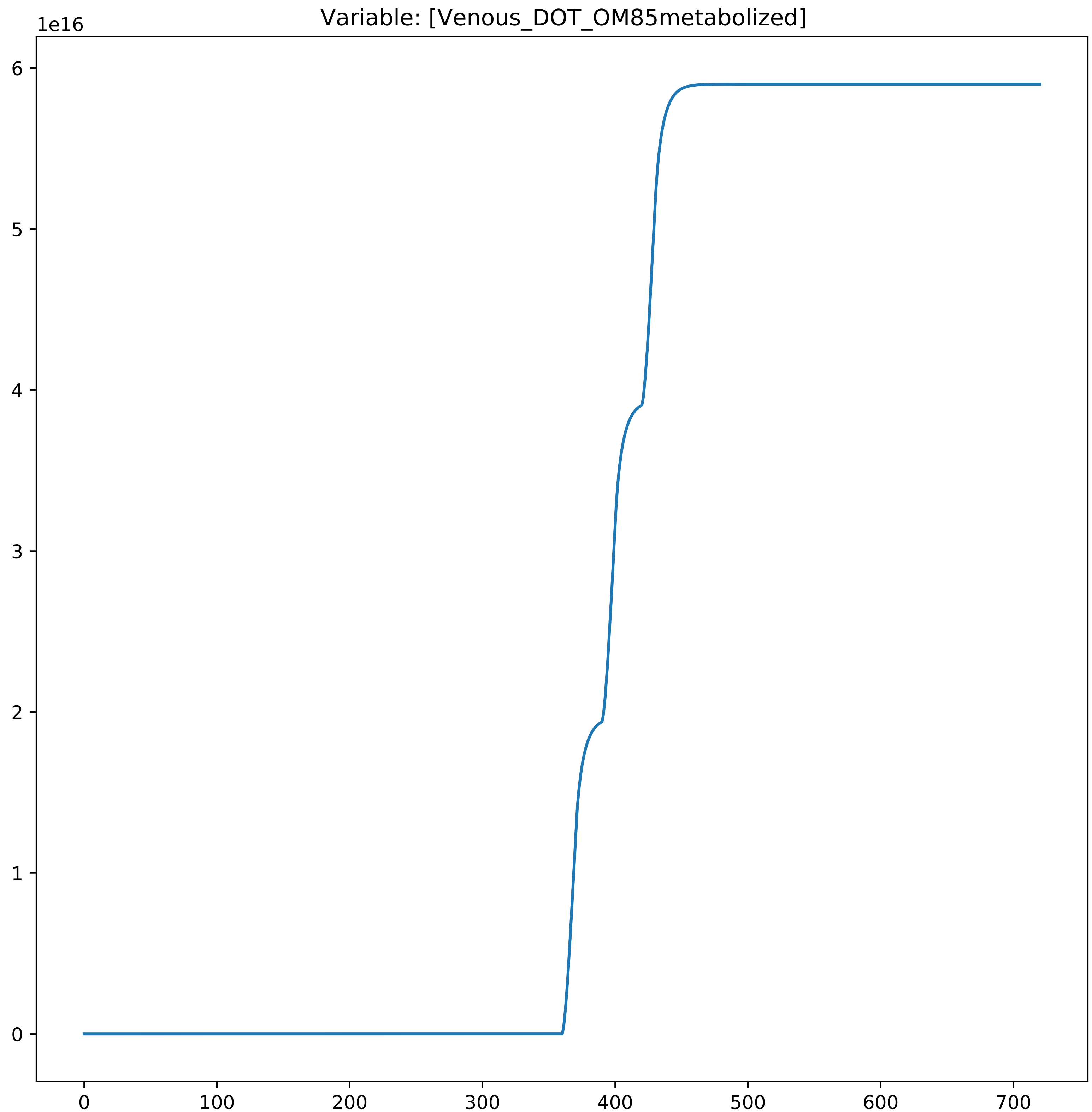
Variable: [Venous_DOT_OM85metabolized]
1e16
6
5
4
3
2
1
0
0
100
200
300
400
500
600
700
time [day]

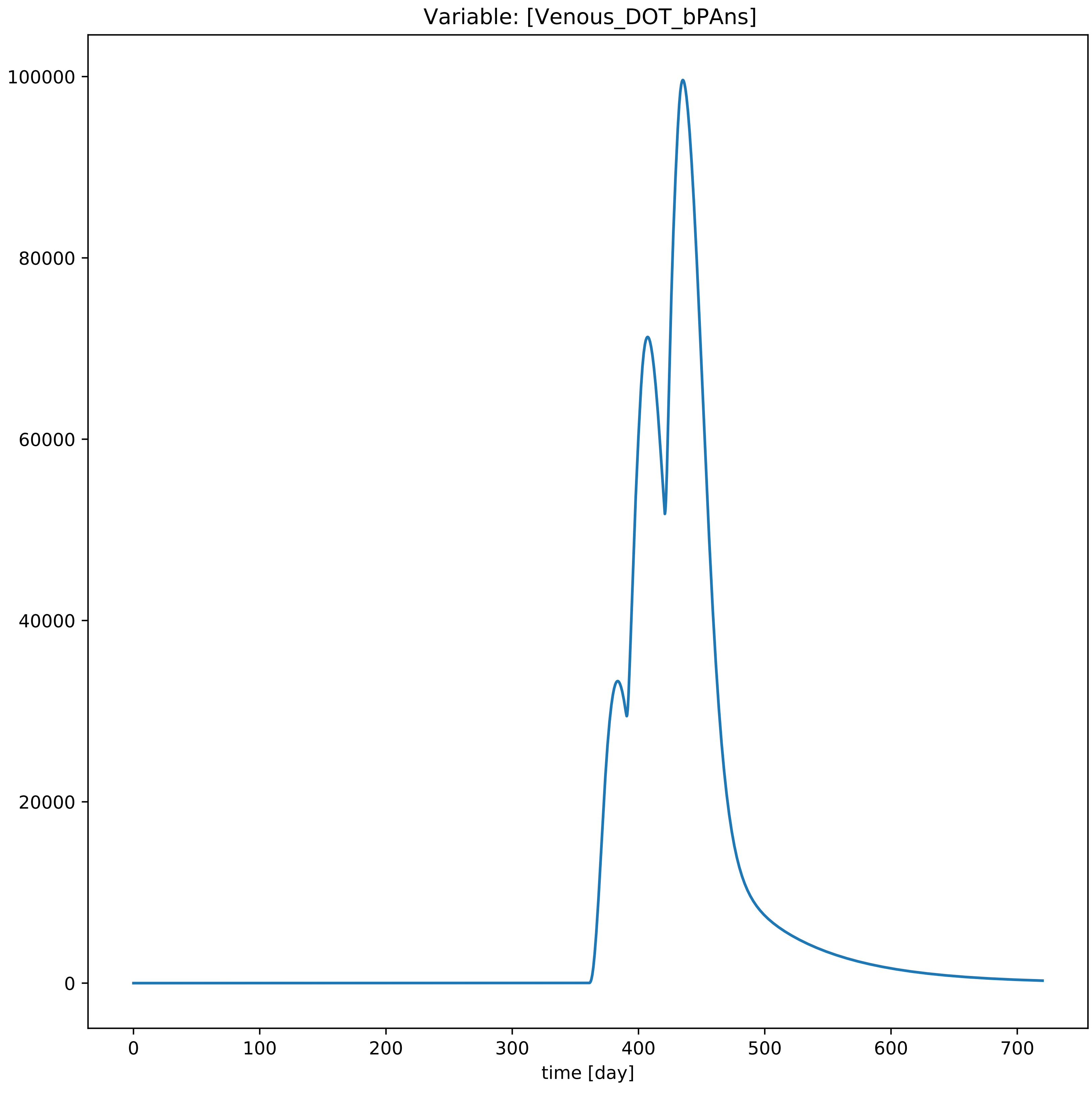

Variable: [Venous_DOT_bPAns]
100000
80000
60000
40000
20000
0
0
100
200
300
400
500
600
700
time [day]

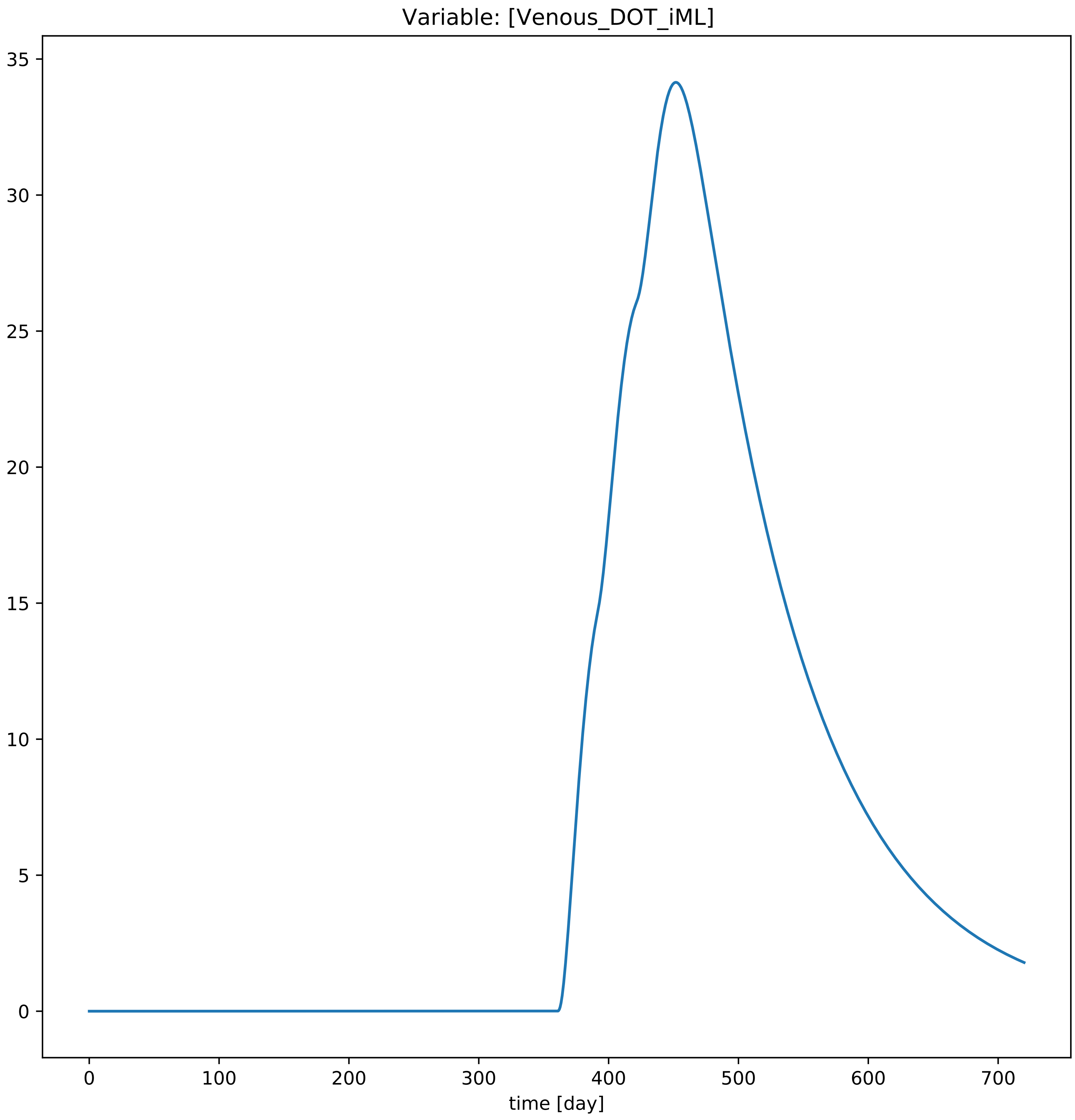

Variable: [Venous_DOT_iML]
35
30
25
20
15
10
5
0
0
100
200
300
400
500
600
700
time [day]

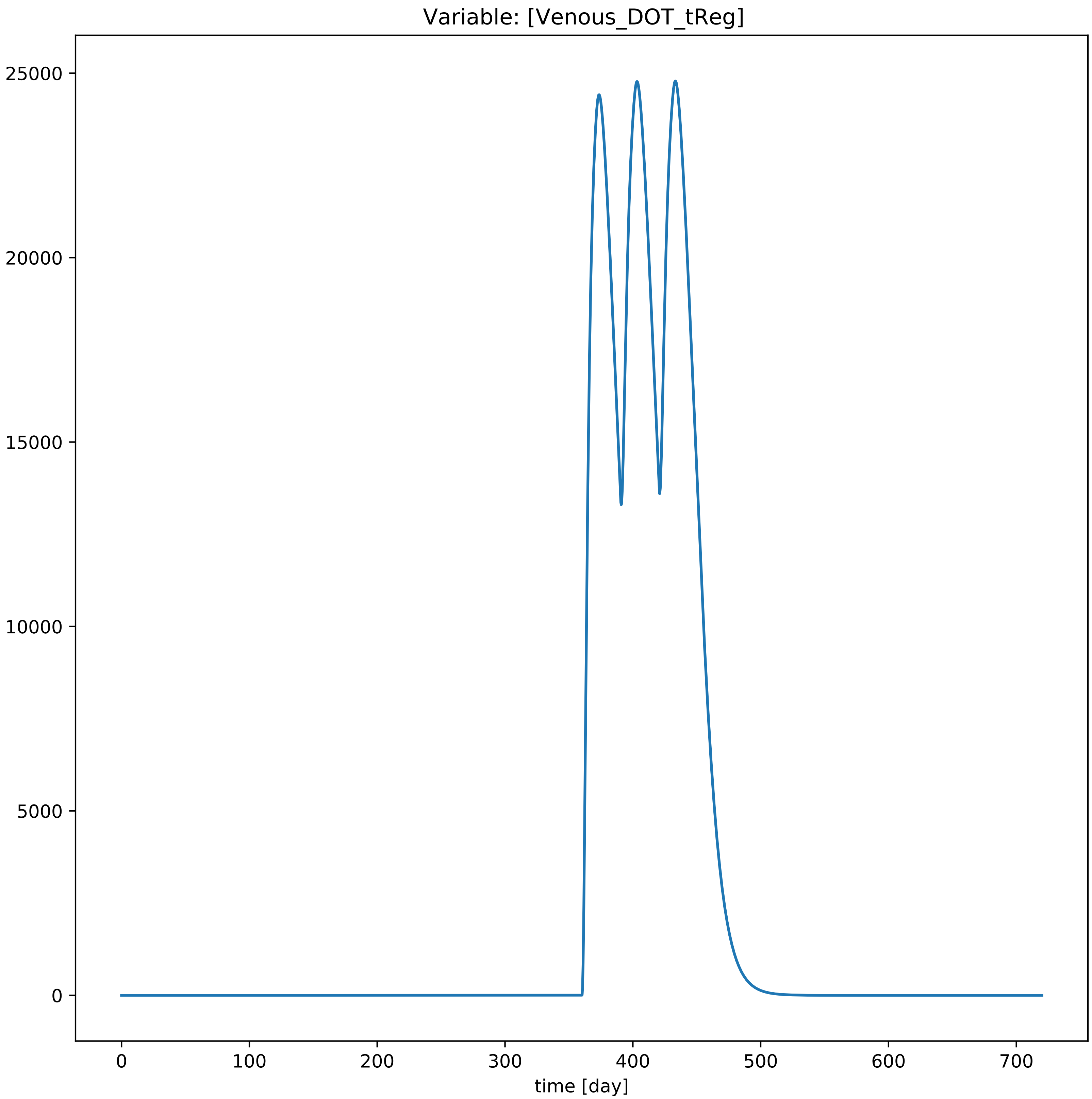

Variable: [Venous_DOT_tReg]
25000
20000
15000
10000
5000
0
0
100
200
300
400
500
600
700
time [day]

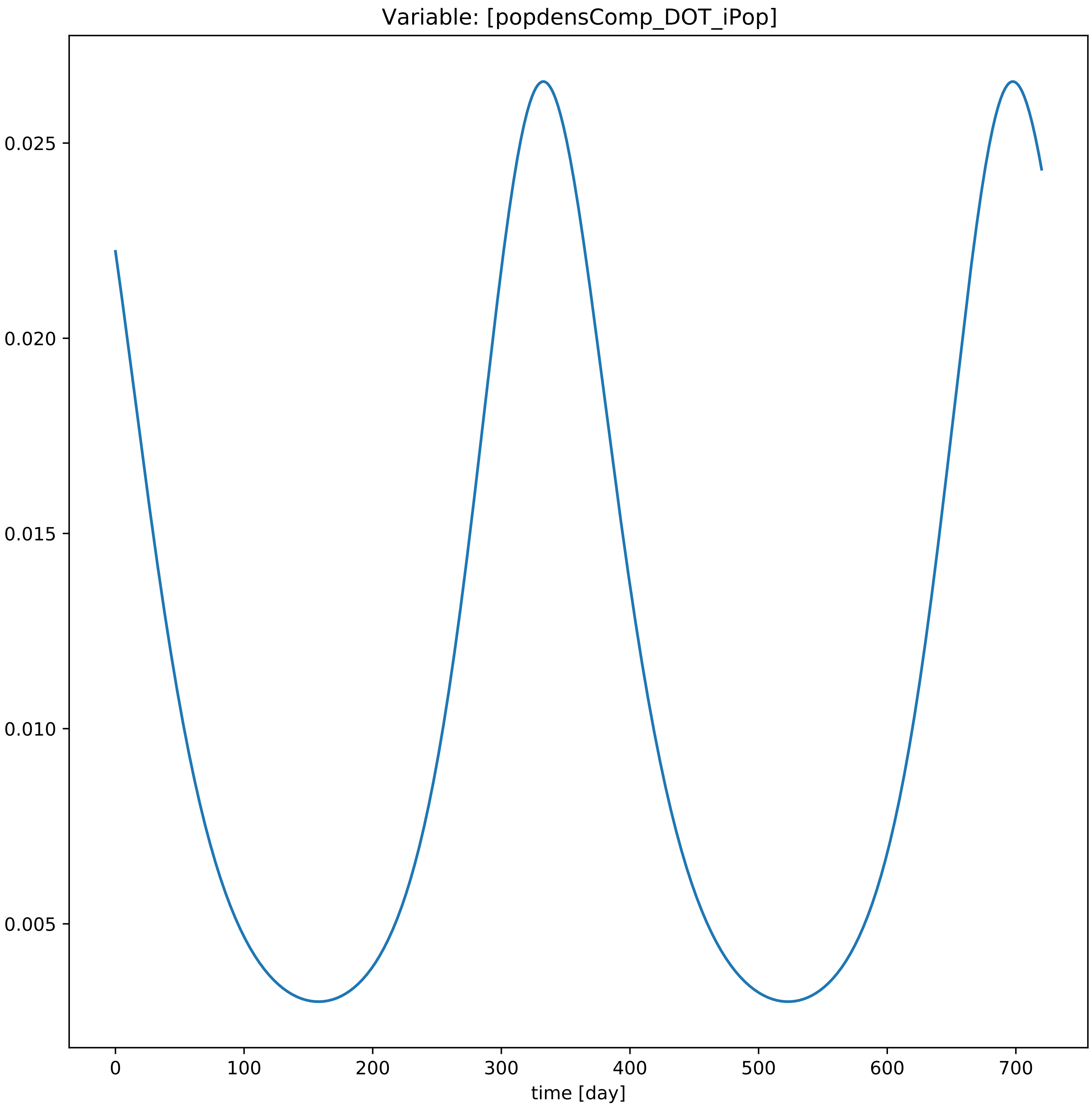
Variable: [popdensComp_DOT_iPop]
0.025
0.020
0.015
0.010
0.005
0
100
200
300
400
500
600
700
time [day]

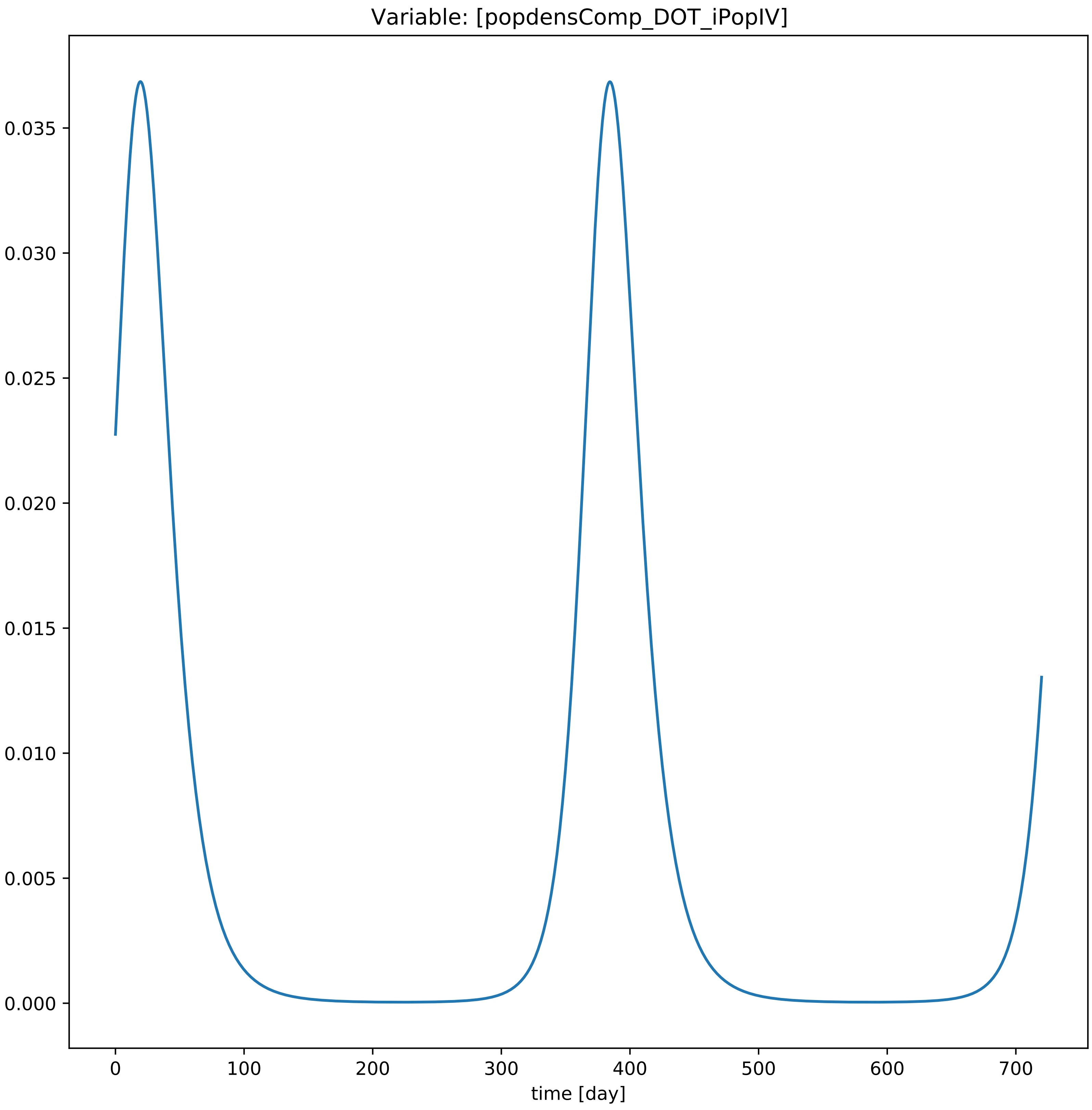
Variable: [popdensComp_DOT_iPopIV]
0.035
0.030
0.025
0.020
0.015
0.010
0.005
0.000
0
100
200
300
400
500
600
700
time [day]

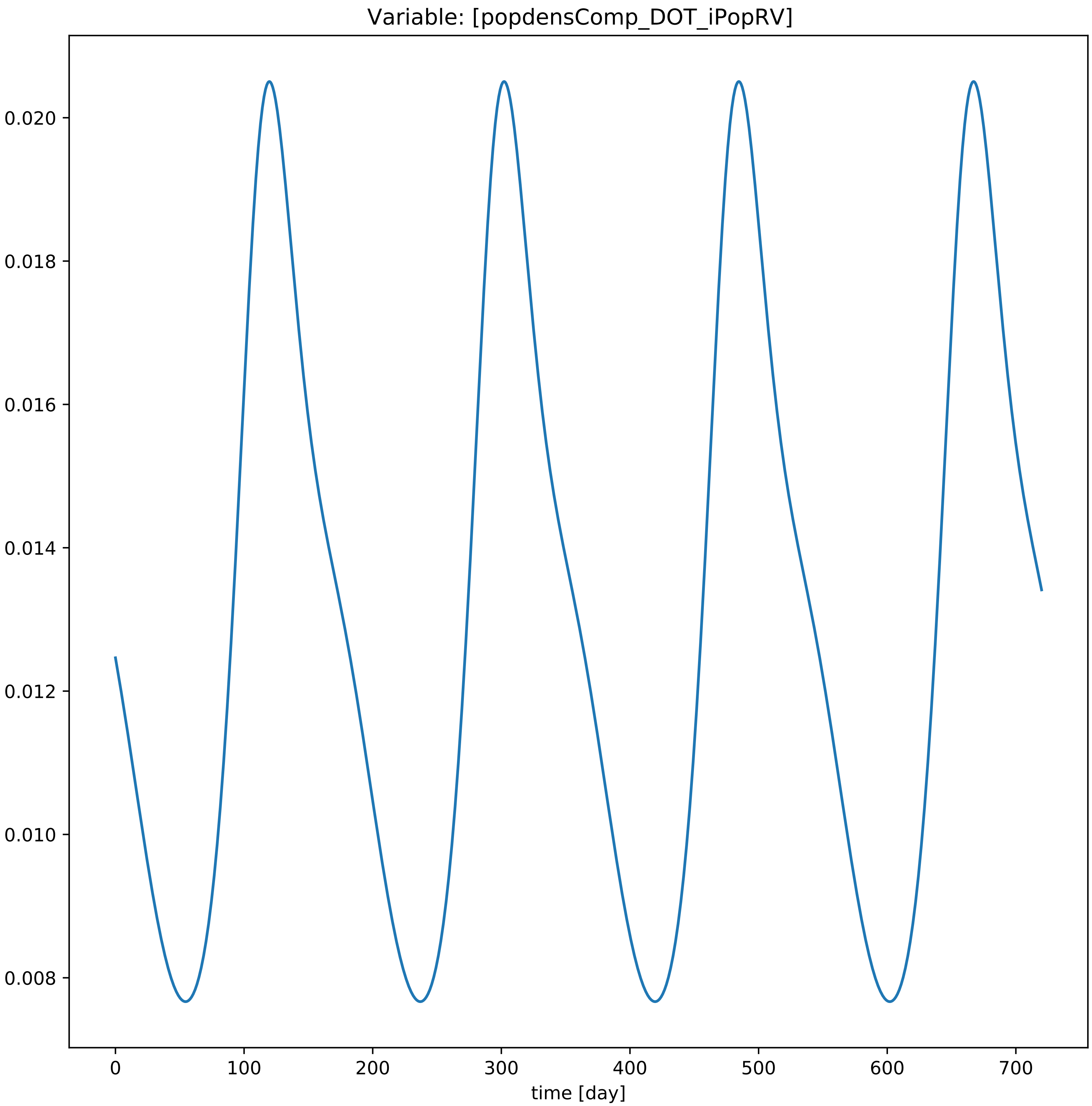

Variable: [popdensComp_DOT_iPopRV]
0.020
0.018
0.016
0.014
0.012
0.010
0.008
0
100
200
300
400
500
600
700
time [day]

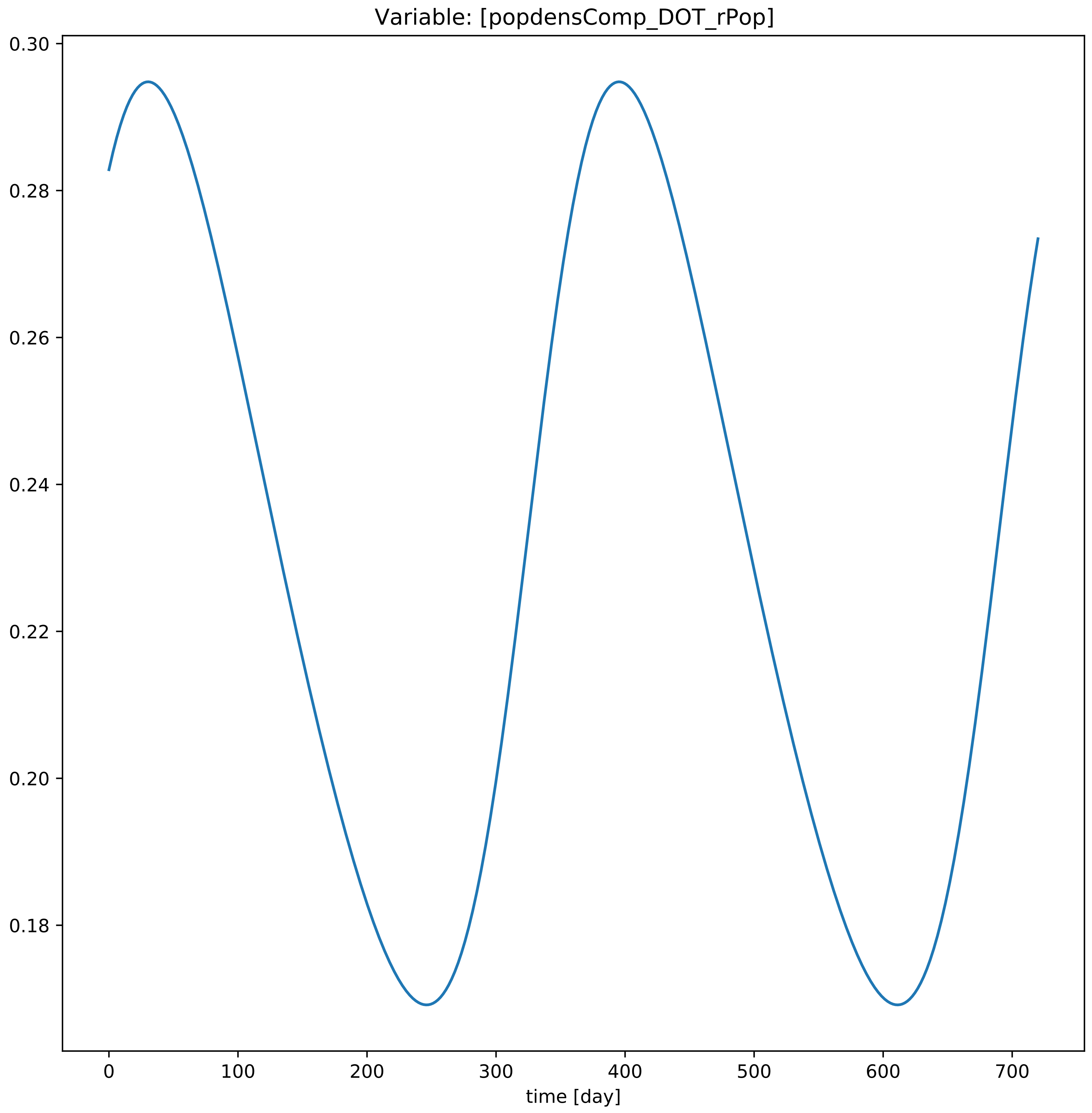

Variable: [popdensComp_DOT_rPop]
0.30
0.28
0.26
0.24
0.22
0.20
0.18
0
100
200
300
400
500
600
700
time [day]

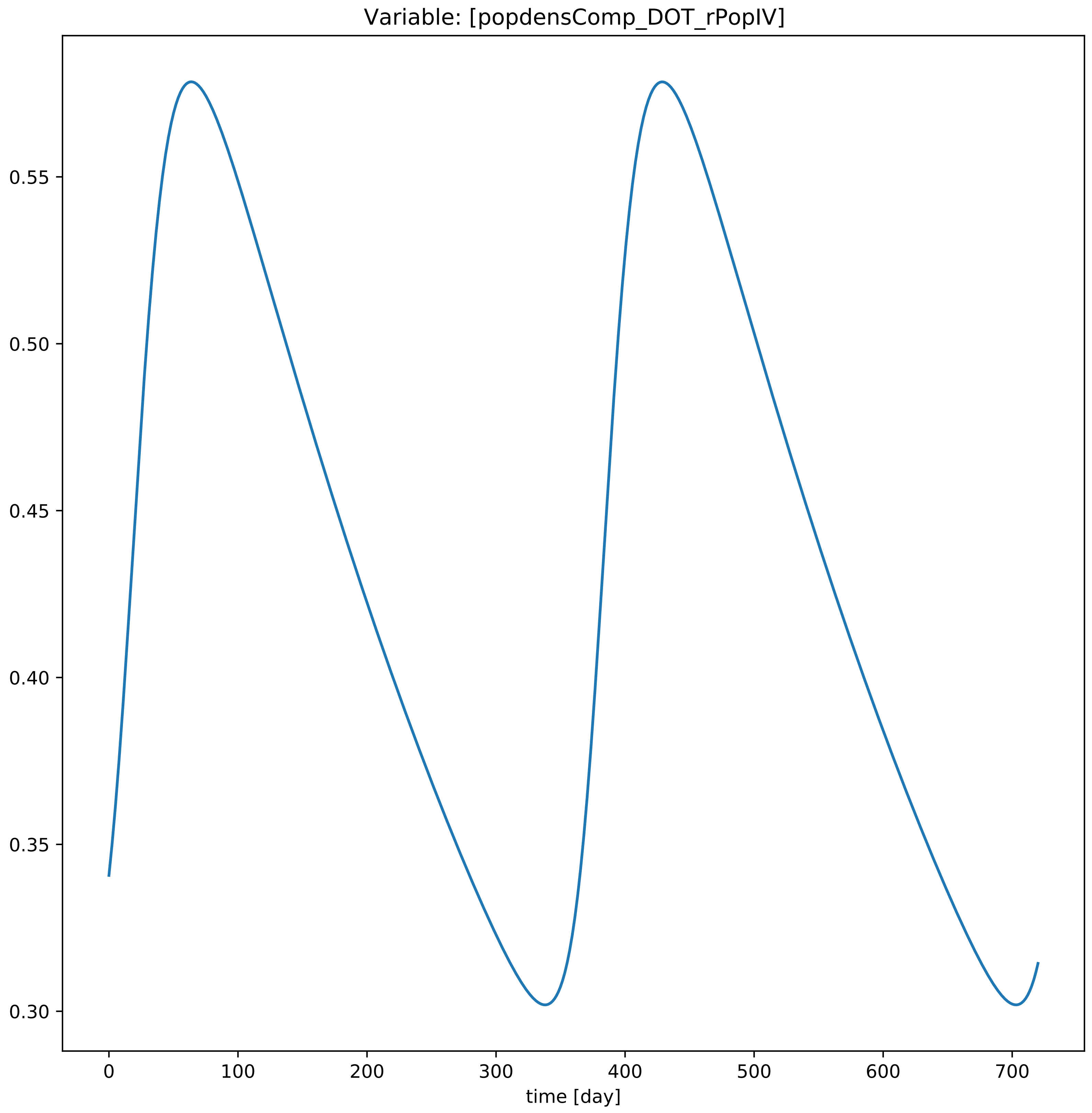

Variable: [popdensComp_DOT_rPopIV]
0.55
0.50
0.45
0.40
0.35
0.30
0
100
200
300
400
500
600
700
time [day]

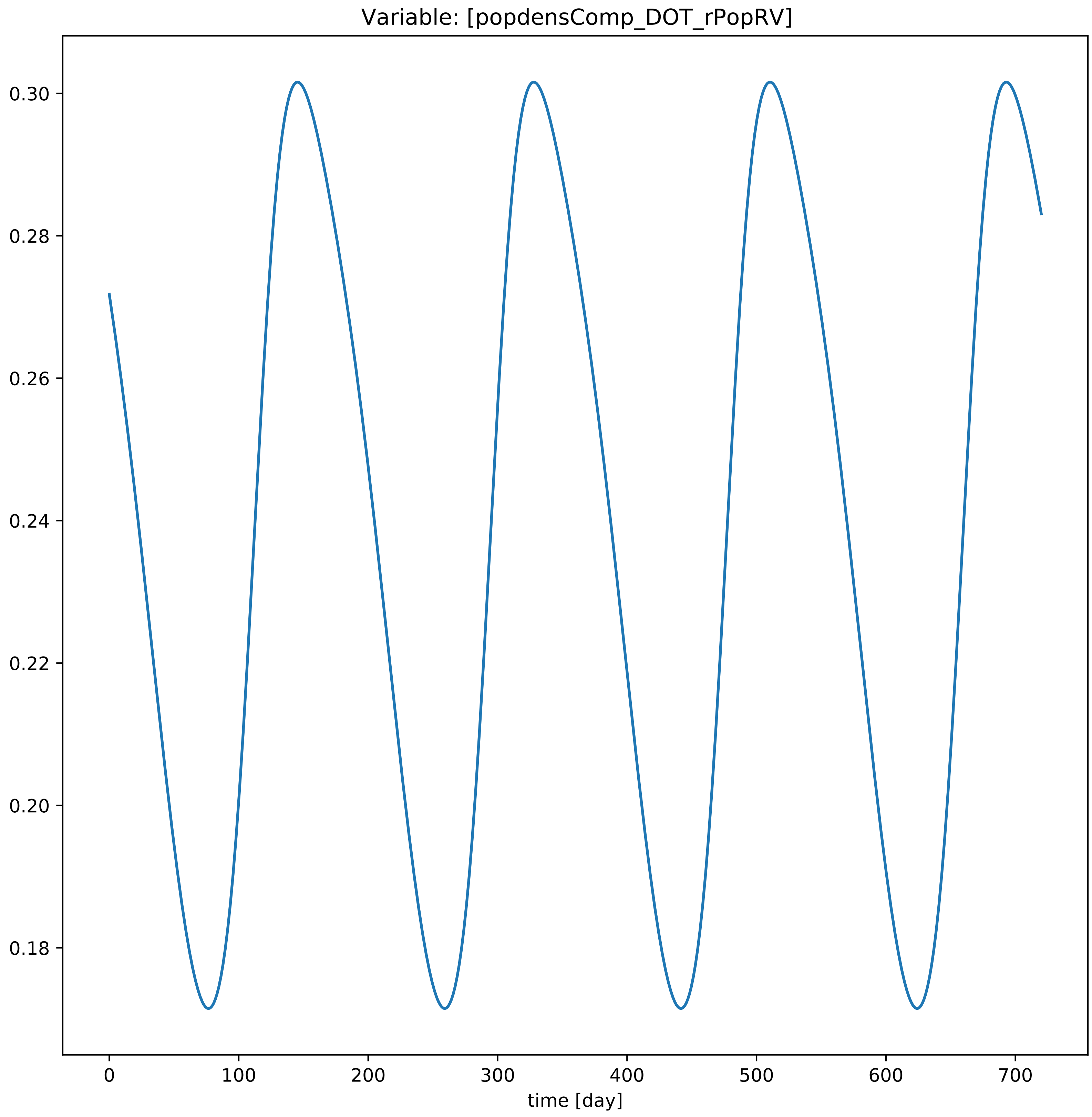

Variable: [popdensComp_DOT_rPopRV]
0.30
0.28
0.26
0.24
0.22
0.20
0.18
0
100
200
300
400
500
600
700
time [day]

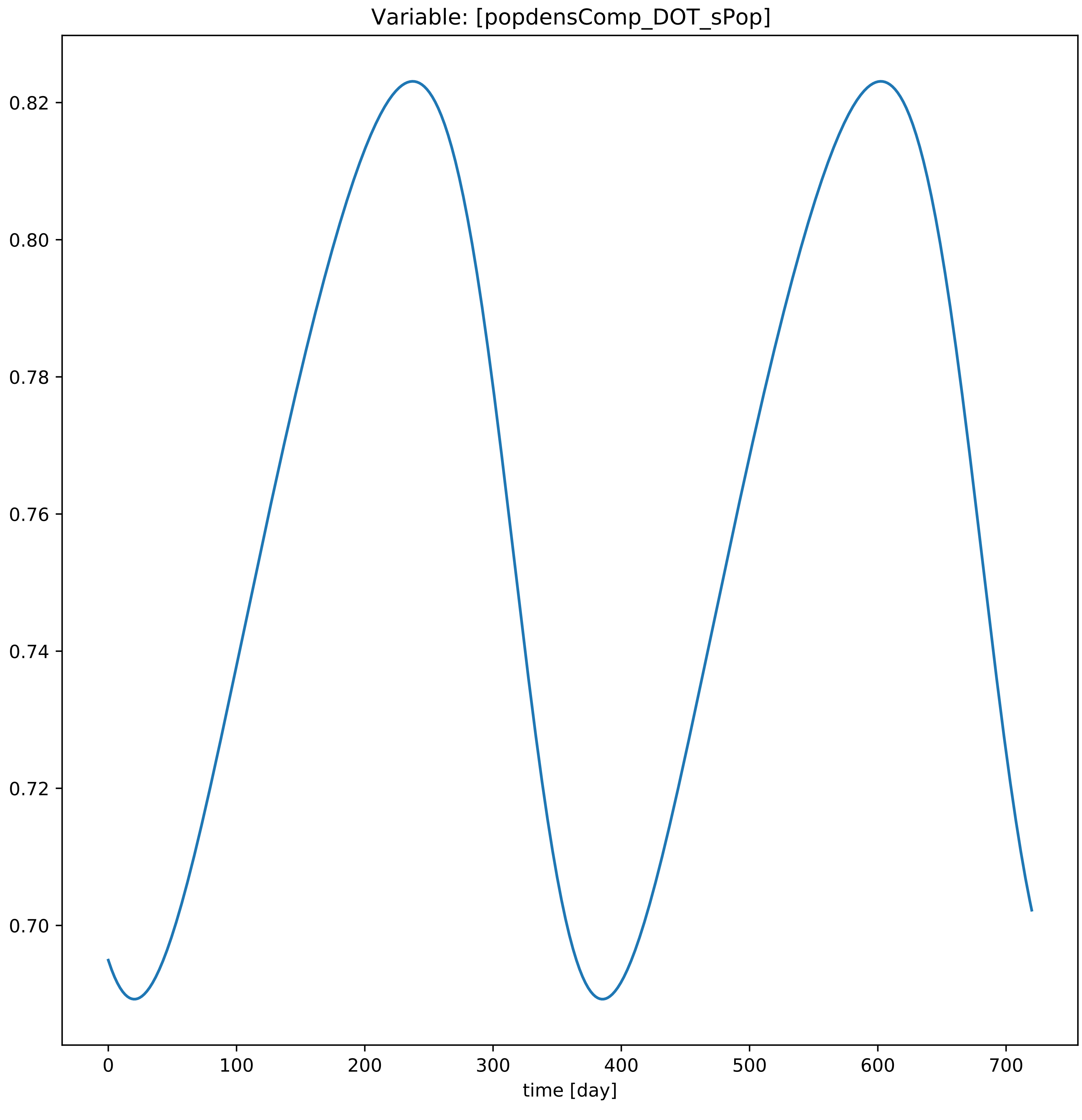

Variable: [popdensComp_DOT_sPop]
0.82
0.80
0.78
0.76
0.74
0.72
0.70
0
100
200
300
400
500
600
700
time [day]

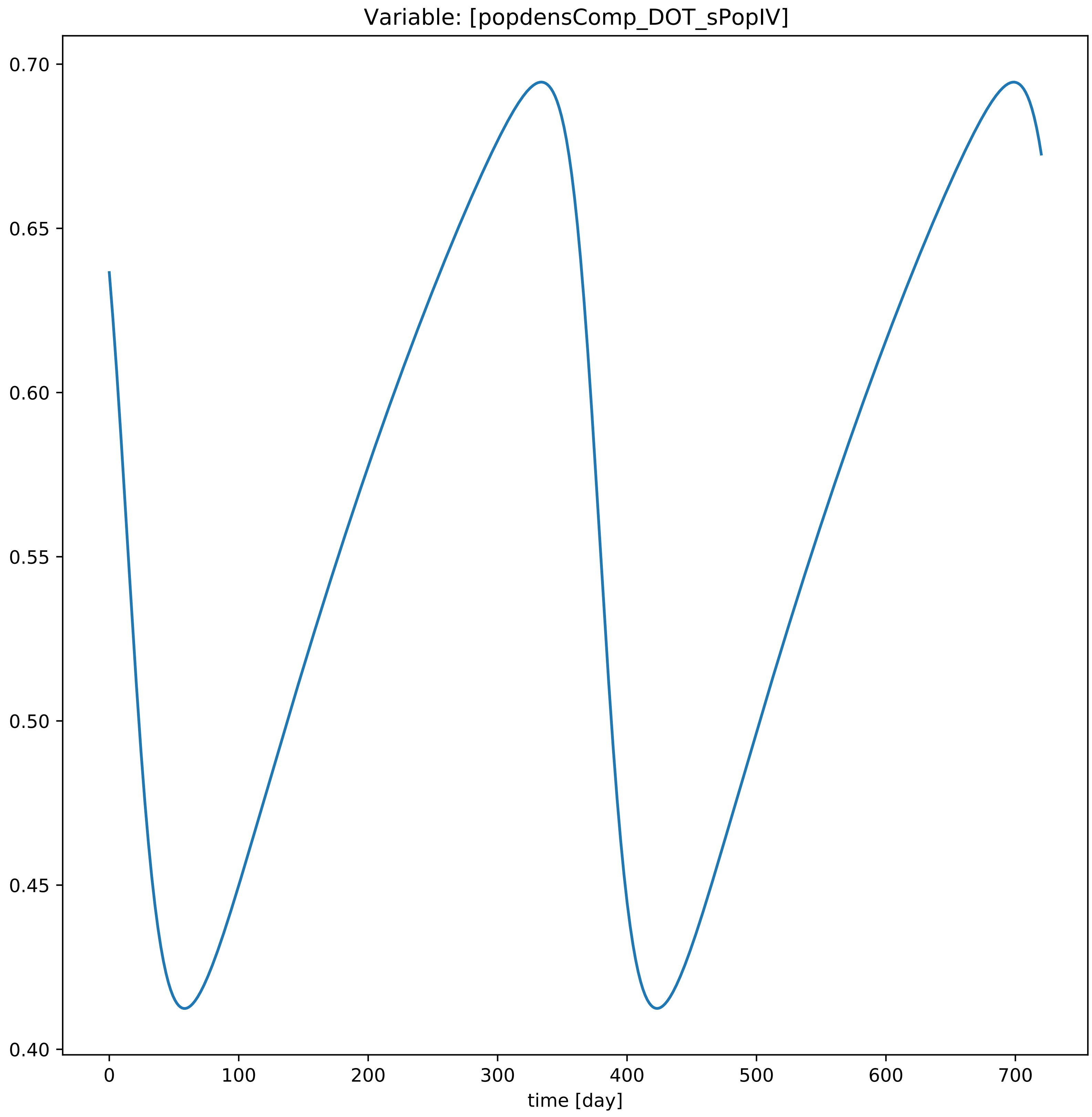

Variable: [popdensComp_DOT_sPopIV]
0.70
0.65
0.60
0.55
0.50
0.45
0.40
0
100
200
300
400
500
600
700
time [day]

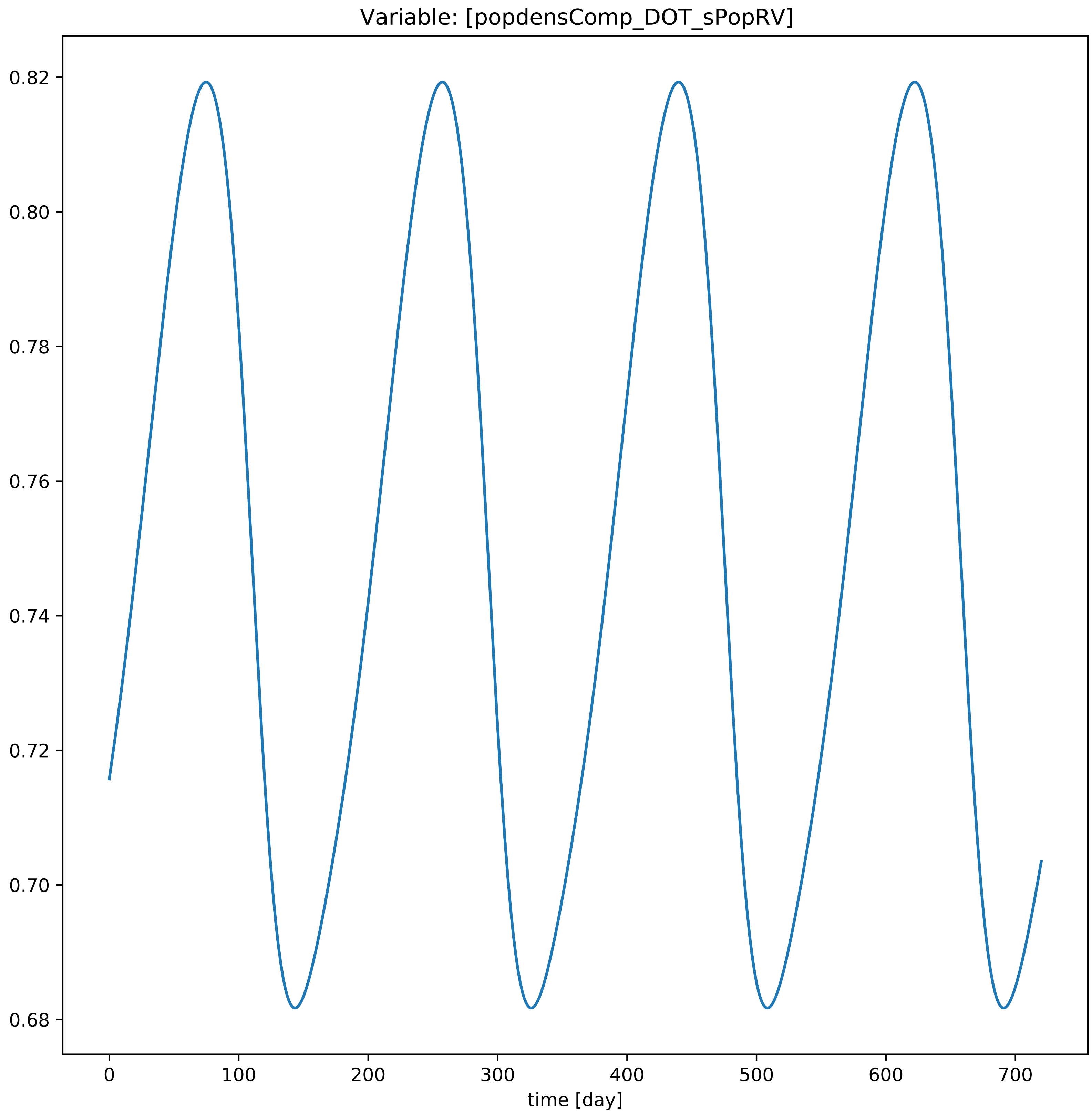

Variable: [popdensComp_DOT_sPopRV]
0.82
0.80
0.78
0.76
0.74
0.72
0.70
0.68
0
100
200
300
400
500
600
700
time [day]

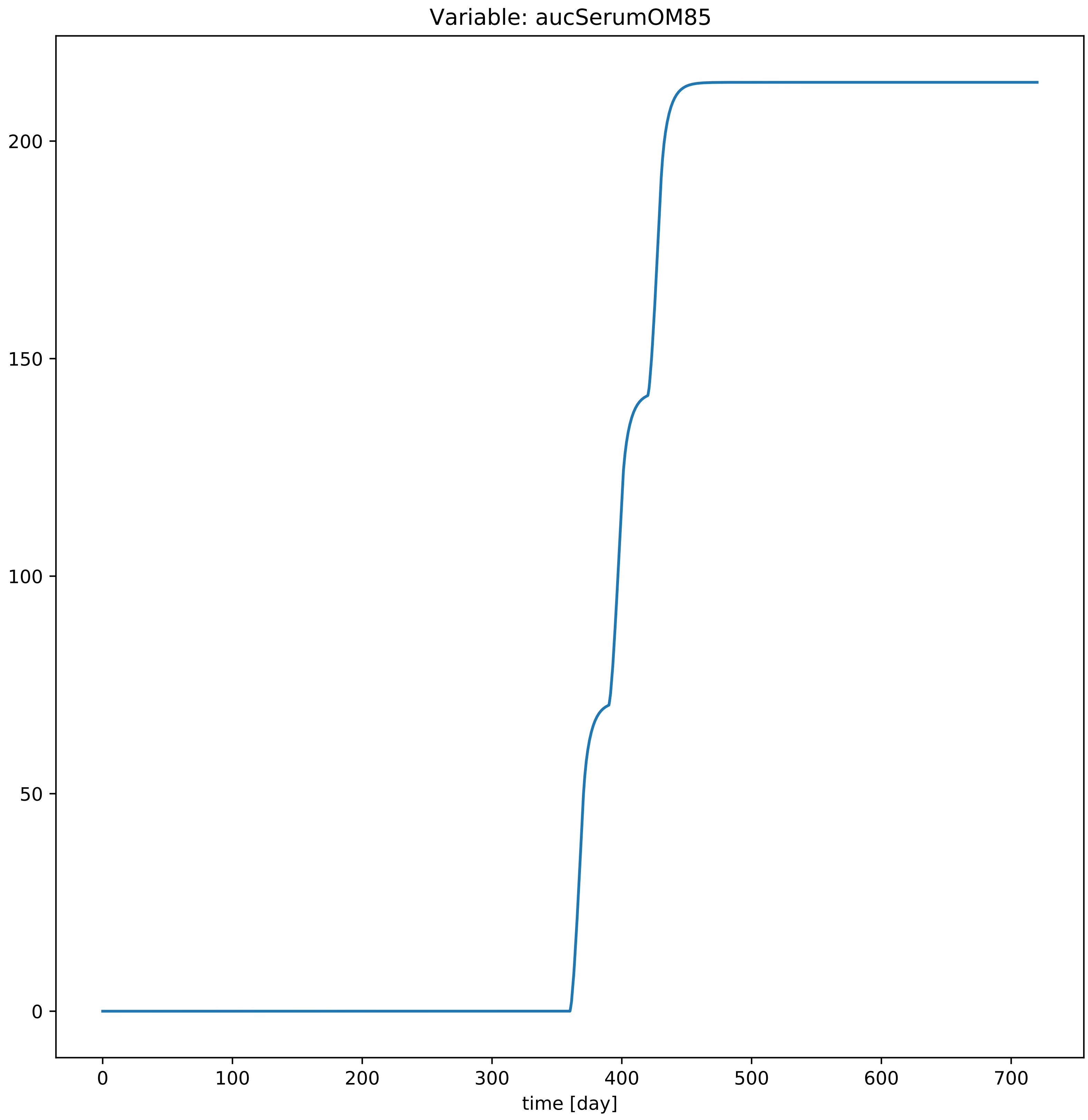

Variable: aucSerumOM85
200
150
100
50
0
0
100
200
300
400
500
600
700
time [day]

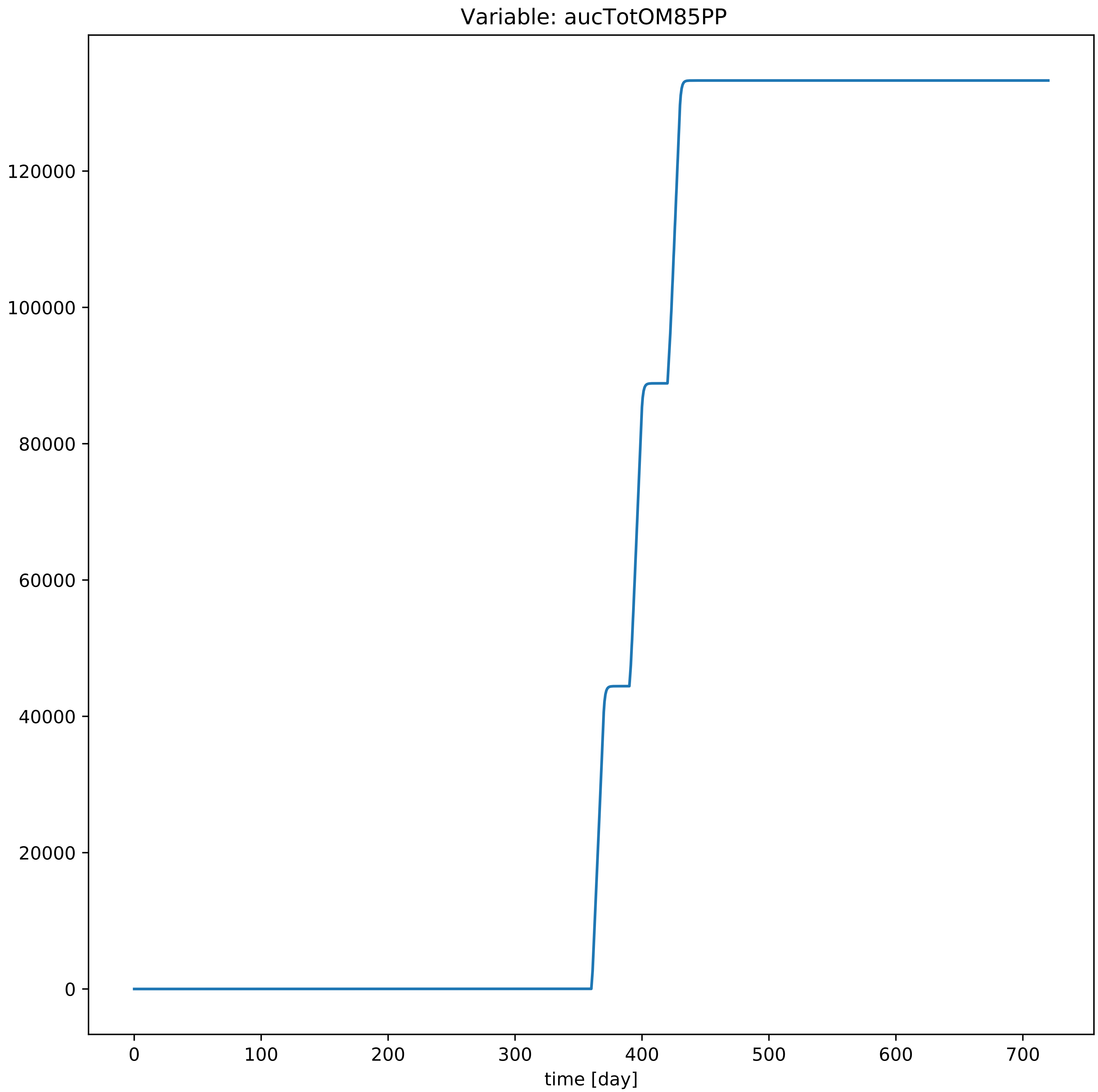

Variable: aucTotOM85PP
120000
100000
80000
60000
40000
20000
0
0
100
200
300
400
500
600
700
time [day]
